# *In vivo* methylation of OLA1 revealed by activity-based target profiling of NTMT1†
†Electronic supplementary information (ESI) available. See DOI: 10.1039/c9sc02550b


**DOI:** 10.1039/c9sc02550b

**Published:** 2019-08-09

**Authors:** Kaimin Jia, Gaochao Huang, Wei Wu, Ruben Shrestha, Bingbing Wu, Yulan Xiong, Ping Li

**Affiliations:** a Department of Chemistry , Kansas State University , Manhattan , Kansas 66506 , USA . Email: pli@k-state.edu; b Department of Anatomy and Physiology , Kansas State University , Manhattan , Kansas 66506 , USA

## Abstract

N-Terminal methyltransferase 1 (NTMT1) catalyzes the N-terminal methylation of proteins with a specific N-terminal motif after methionine removal. Aberrant N-terminal methylation has been implicated in several cancers and developmental diseases. Together with motif sequence and signal peptide analyses, activity-based substrate profiling of NTMT1 utilizing (*E*)-hex-2-en-5-ynyl-*S*-adenosyl-l-methionine (Hey-SAM) revealed 72 potential targets, which include several previously confirmed ones and many unknowns. Target validation using normal and NTMT1 knock-out (KO) HEK293FT cells generated by CRISPR-Cas9 demonstrated that Obg-like ATPase 1 (OLA1), a protein involved in many critical cellular functions, is methylated *in vivo* by NTMT1. Additionally, Hey-SAM synthesis achieved ≥98% yield for SAH conversion.

## Abstract 2

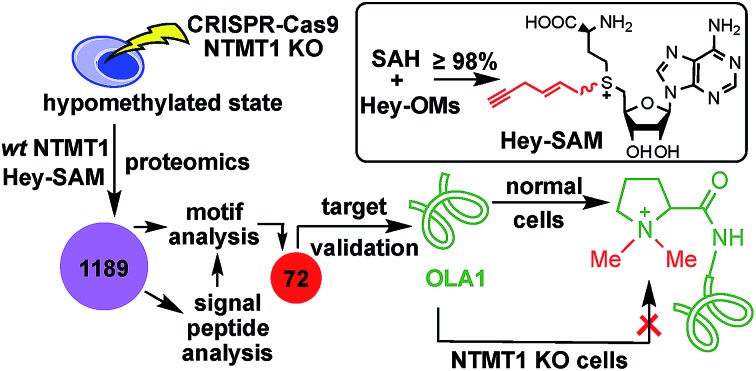
Target profiling of NTMT1 by Hey-SAM revealed that OLA1 undergoes N-terminal methylation catalyzed by NTMT1 *in vivo*.

## Introduction

Protein methylation, one of the most common post-translational modifications catalyzed by protein methyltransferases (PMTs),^1^ plays important roles in regulating epigenetics and cell signalling pathways.^2^,^3^ While most studies focus on protein arginine and lysine methyltransferases (PRMT and PKMT, respectively),^4^–^6^ the recent discovery of NTMT1 has attracted much attention due to its wide substrate spectrum toward non-histone proteins.^7^–^12^ Using *S*-adenosyl-l-methionine (SAM) as a methyl donor, NTMT1 methylates proteins with a specific N-terminal sequence of XPK after initial methionine removal.^11^,^12^ Subsequent study employing limited peptide arrays showed that NTMT1 has an expanded substrate specificity,^13^ suggesting that N-terminal methylation is a widespread post-translational modification.

N-terminal methylation has been established as a regulator of protein–DNA and protein–protein interactions for a number of proteins, such as RCC1,^11^ CENPA/B,^7^,^9^,^14^ DDB2,^8^ PARP3,^10^ and MYL9,^15^ playing important roles in cell mitotic progression, DNA damage repair, and regulation of protein function.^16^ Dysregulation of NTMT1 has been implicated in various cancers and developmental diseases.^17^ For example, NTMT1 is downregulated in patients with breast cancer and its loss promotes the growth and metastasis of breast cancer cells, suggesting that NTMT1 is a tumor suppressor.^18^ Conversely, NTMT1 is upregulated in colon cancer and has been proposed to function as a tumor promoter and oncogene.^19^ NTMT1-knockout (KO) mice exhibited the phenotype of premature aging.^20^ All of this indicates that understanding the signal transduction involving NTMT1 would be important for developing new therapeutics for cancer treatment.

Due to the potential wide presence of NTMT1-catalyzed methylation and the fact that dysregulation of NTMT1 occurs in a tissue-specific manner, we decided to develop a substrate profiling method that would allow us to study NTMT1 in a disease-specific manner. Activity-based substrate profiling using click chemistry has emerged as a powerful tool to identify PMT targets,^21^–^26^ and usually involves an engineered mutant–cofactor pair for target profiling. Here, we report a modified profiling method that employs wild-type (wt) NTMT1 and Hey-SAM for target identification.

## Results and discussion

**Fig. 1:**
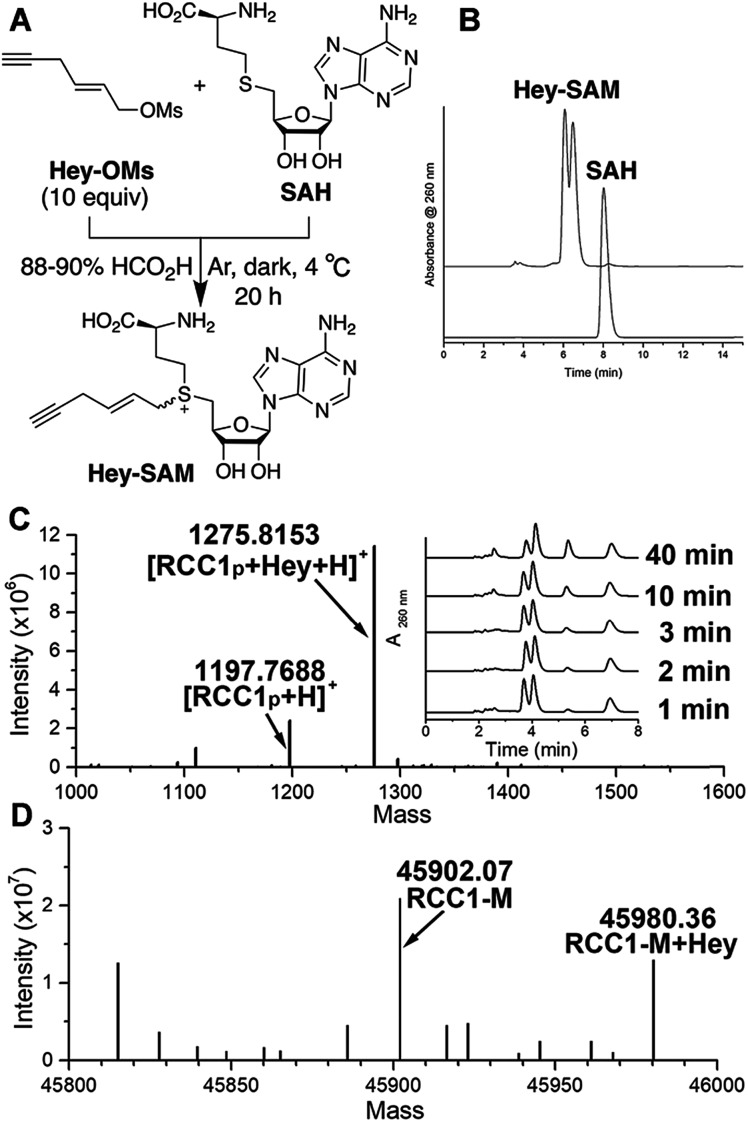
(A) Schematic illustration of Hey-SAM synthesis. (B) Hey-SAM synthesis monitored at 260 nm using HPLC. (C and D) MS analysis of Hey-SAM alkylation catalyzed by wt NTMT1 with RCC1_p_ (C) and methionine-removed RCC1 (D). The inset in (C) represents HPLC monitoring of the progress of the alkylation reaction. Peaks eluted at 3.9, 5.4, and 6.9 min are Hey-SAM, SAH, and the internal standard adenosine.

To identify a SAM analogue that can be accepted by wt NTMT1, we examined all SAM analogues reported so far.^27^ Among these analogues, Hey-SAM (Fig. 1A) is especially intriguing as (1) it has a high reactivity due to the presence of a sulfonium-β-sp^2^ carbon;^28^–^30^ (2) it cannot be accepted by many wt PRMTs and PKMTs due to its bulky size,^24^,^25^ thus eliminating non-specific labelling by these endogenous PMTs; and (3) it could be accepted by wt NTMT1 due to the large cofactor pocket in the enzyme structure.^31^,^32^ Hey-SAM was previously prepared from *S*-adenosyl-l-homocysteine (SAH) and (*E*)-hex-2-en-5-ynyl bromide,^33^ but the yield was only 35%.^33^ Thus, a modified approach was developed to use SAH and a highly reactive Hey-mesylate (Hey-OM) (Fig. 1A and Scheme S1†).^34^ Almost complete SAH conversion was achieved in 20 h. After repeated ether extractions to remove excess Hey-OMs, the aqueous mixture was analysed by HPLC (Fig. 1B). Epimers of Hey-SAM were obtained in ≥98% yield and were therefore directly used for further studies without HPLC purification.

Since RCC1 (Fig. S1†) is a well-known substrate of NTMT1 *in vitro* and *in vivo*,^11^ it was selected as a control for target profiling of NTMT1 described here. Chemically synthesized RCC1_p_ (Fig. S2†), representing the first 10 N-terminal residues of RCC1 after initial methionine removal, was selected as the peptide equivalent of RCC1 protein. As revealed by mass spectrometry (MS), Hey-SAM can be accepted by wt NTMT1 to catalyze monoalkylations with both RCC1_p_ (Fig. 1C and S2†) and recombinant RCC1 purified from *Escherichia coli* (Fig. 1D and S3†), with the concurrent release of SAH (Fig. 1C inset). The alkylation was enzyme-catalyzed as the absence of wt NTMT1 stops the reaction (Fig. S3†). The modification site was mapped to the N-terminus of RCC1 by tandem MS (Fig. S3†). It is of note that only the fast-eluting epimer is accepted by NTMT1 as a SAM surrogate (Fig. 1C inset).

**Fig. 2:**
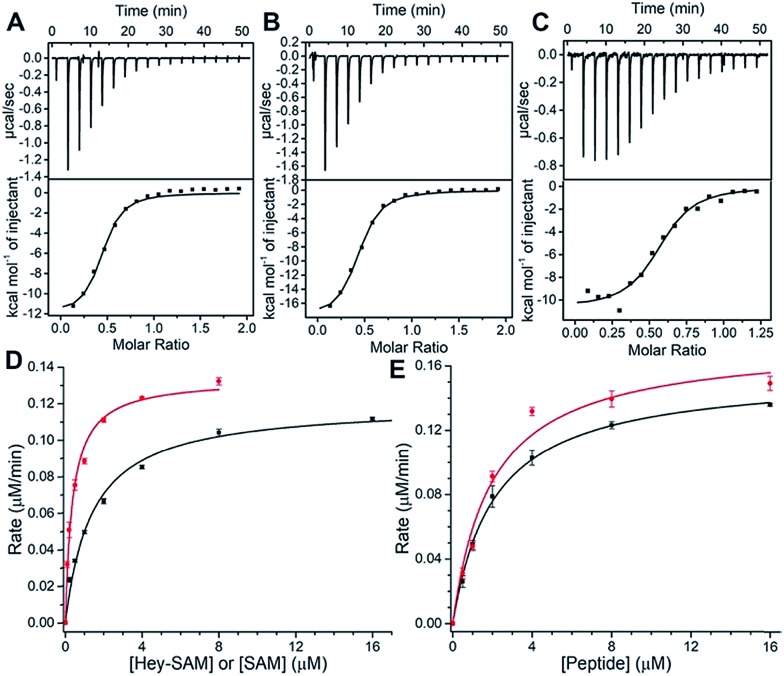
(A–C) Affinity measurements of Hey-SAM (A), SAM (B), and RCC1 peptide (C) with wt NTMT1 by ITC. (D and E) Determination of kinetic parameters of cofactors (D) and RCC1 peptide (E) with wt NTMT1. (D) The red and black solid lines represent SAM and Hey-SAM, respectively, in the presence of saturated RCC1 peptide; (E) the red and black lines represent RCC1 peptide in the presence of saturated SAM and Hey-SAM, respectively.

To evaluate substrate properties of Hey-SAM with wt NTMT1 and compare them with those of the native cofactor SAM, isothermal titration calorimetry (ITC) and steady-state kinetic assays were performed (Fig. 2 and Table 1). Dissociation constants (*K*_d_) for SAM, Hey-SAM and RCC1_p_ were determined to be similar, suggesting that their binding modes with wt NTMT1 are likely to be identical. Steady-state kinetic parameters were determined by monitoring SAH release using HPLC (Fig. 1C inset). While the Michaelis constant (*K*_M_) of Hey-SAM is 4 times higher than that of SAM, their turnover values (*k*_cat_) are almost the same. Furthermore, *K*_M_ and *k*_cat_ of RCC1_p_ in the presence of saturated SAM or Hey-SAM are comparable, indicating that the cofactors have marginal effects on the binding affinity and catalytic efficiency of the peptide substrate. Taken together, it suggests that Hey-SAM is an excellent SAM surrogate for wt NTMT1.

**Table 1:** Thermodynamic and kinetic parameters of cofactors and RCC1_p_ with wt NTMT1

	*K*_M_ (μM)	*k*_cat_ (min^–1^)	*K*_d_ (μM)
SAM	0.38 ± 0.03	0.45 ± 0.01	1.43 ± 0.33
Hey-SAM	1.40 ± 0.09	0.40 ± 0.01	1.29 ± 0.17
RCC1_p_	1.96 ± 0.24/SAM	0.58 ± 0.02/SAM	1.22 ± 0.02
	2.09 ± 0.18/Hey-SAM	0.52 ± 0.01/Hey-SAM	

After demonstrating that Hey-SAM and diazo biotin azide (DBA, for click chemistry) could efficiently label purified RCC1 (Fig. S4†), for a proof-of-concept study, they were utilized to label and pull-down the overexpressed RCC1 from *E. coli* lysates. SDS-PAGE and western blotting (WB) showed that RCC1 was pulled-down with high purity (Fig. S5†). No RCC1 was detected in the controls either in the absence of wt NTMT1 or using SAM to replace Hey-SAM. Quantification of the pull-down RCC1 by WB gave a normalized recovery yield of 10%, indicating that Hey-SAM is an effective SAM surrogate for NTMT1 target profiling.

**Fig. 3:**
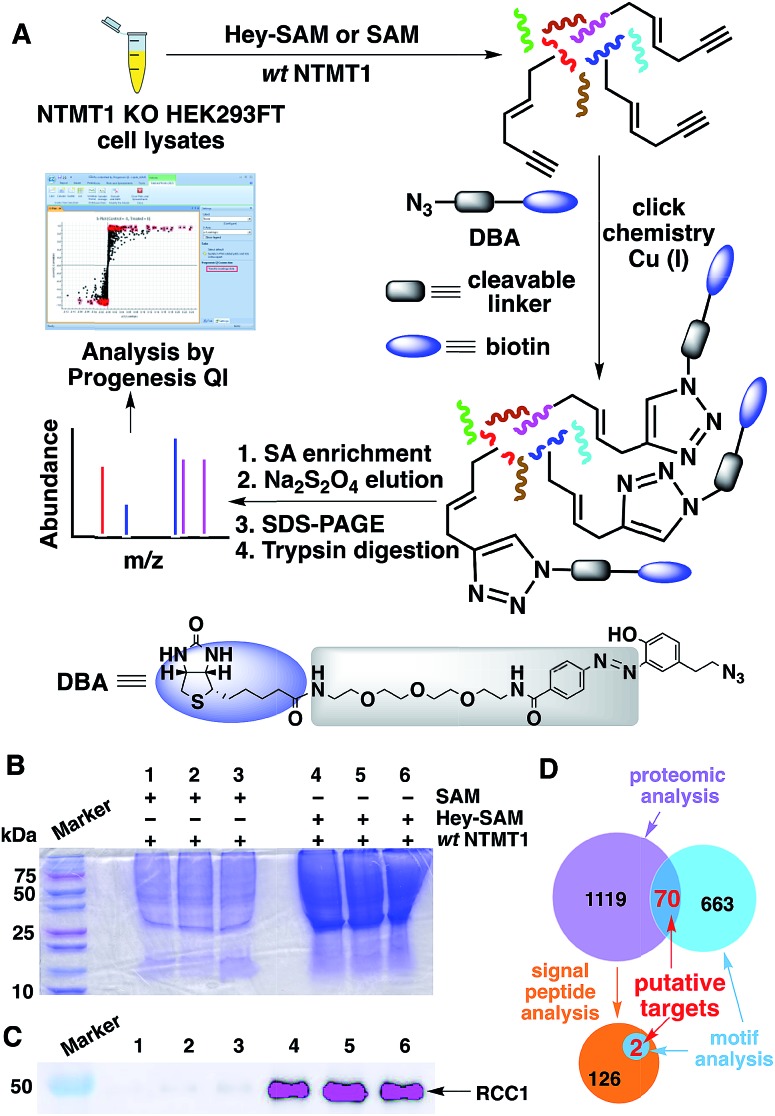
(A) Flowchart of the activity-based target profiling of NTMT1. (B) SDS-PAGE of pull-down proteins. (C) WB detection of RCC1 in pull-down proteins using anti-RCC1 antibody. (D) Identification of putative NTMT1 targets by proteomics and analyses of motif sequences and signal peptides.

Next, we carried out target identification using Hey-SAM with lysates from human embryonic kidney (HEK) 293FT cells following the procedure shown in Fig. 3A. To produce NTMT1-specific hypomethylated cells, CRISPR-Cas9 was used to knockout NTMT1,^35^ which was confirmed by WB and DNA sequencing (Fig. S6†). It should be noted that selection of the control in our experiments was not trivial. All known PKMTs except DOT1L contain a conserved SET-domain,^6^ and cannot accept Hey-SAM as a cofactor surrogate unless they are engineered.^25^ This is consistent with the reported experimental observation that the background labelling by Hey-SAM using HEK293T cell lysates was negligible, judging from the SDS-PAGE.^24^ All nine PRMTs identified so far contain a conserved core region of ∼310 amino acids where the cofactor binds.^6^,^36^ It has been reported that bulky cofactor analogues cannot be accepted by wt PRMT1.^26^ Therefore, the possibility of Hey-SAM being processed by PKMTs and PRMTs, which represent most PMTs, is rather low. In this respect, either SAM in the presence of NTMT1 or Hey-SAM in the absence of NTMT1 could be selected as the control. If Hey-SAM was accepted by a PMT in addition to NTMT1, using SAM in the presence of NTMT1 as the control would reveal a target list reflecting a broader activity of Hey-SAM rather than NTMT1-specific activity. However, this technical flaw could be corrected by the motif analysis described below. If a protein substrate was shared by both NTMT1 and another PMT, and Hey-SAM was also accepted by that PMT, using Hey-SAM in the absence of NTMT1 as the control would cause an indistinguishable max fold change between the control and treated samples (discussed below), resulting in the loss of the target protein. To avoid this, we decided to select SAM in the presence of NTMT1 as the control. Consistent with our expectations, SDS-PAGE of pull-down proteins showed much stronger Coomassie staining signals for samples labelled with Hey-SAM than for the controls labelled with SAM (Fig. 3B). As a quality control, RCC1 was pulled-down and detected by WB only in the samples treated with Hey-SAM (Fig. 3C), demonstrating the validity of our profiling protocol.

To uncover NTMT1 targets, proteins identified by MS using Progenesis QI (Waters) should have (1) a *p* value cut-off of ≤0.05; and (2) a max fold change value (Hey-SAM treated sample/SAM treated sample) of ≥2. This treatment gave 1189 candidate proteins. To further narrow down the targets, motif analysis was carried out for the first four N-terminal residues based on the limited peptide arrays published previously.^13^,^31^ Following the expanded N-terminal sequence consensus (Table S1†), a motif search in a human protein database returned 733 hits, in which 70 proteins were also predicted by proteomic analysis (Fig. 3D and Table S2†).

Among these 70 putative NTMT1 targets, RCC1, SET protein, and 60S ribosomal protein L23a (RL23A) have been reported previously.^11^ Six proteins with high, medium, and low confidence scores (highlighted in yellow in Table S2†) were selected for target validation *in vitro*. Four were found to be methylated by NTMT1 at the peptide (PB1 and SPD2B) or protein (OLA1 and RS14) level (Fig. S7–S12†). It is worth noting that, while DDX60L has the same N-terminal sequence (MGSK) as PB1, it cannot undergo methylation by NTMT1. The 5^th^ residues of DDX60L and PB1 are negatively charged aspartate and positively charged arginine, respectively. This indicates that the residue at the 5^th^ position is also important for NTMT1 substrate recognition, which prefers a positively charged residue for enhanced interactions with the negatively charged environment.^31^

It is of note that targets identified by proteomics may undergo peptidase-mediated N-terminal truncations followed by NTMT1-catalyzed methylation. Therefore, signal peptide analysis was also performed with the targets identified by proteomics, yielding 128 proteins that may undergo truncation to remove a signal sequence (Fig. 3D). Further motif analysis of the resulting matured proteins gave two potential NTMT1 targets, FECH and LAMC2 (Fig. 3D and Table S2†). Target validation using their N-terminal peptide analogues revealed that only FECH can be methylated by NTMT1 *in vitro* (Fig. S13 and S14†).

Finally, we decided to validate the methylation of OLA1 *in vivo* as it regulates many critical cellular functions.^37^ OLA1 is a member of the P-loop GTPases with the preference to hydrolyze ATP over GTP.^38^ Its function is to regulate numerous cellular processes *via* protein–protein interactions.^39^–^48^ For example, OLA1 acts as a breast cancer suppressor by binding with eukaryotic initiation factor 2 to stop protein synthesis.^40^ It also serves as a suppressor of colon and ovarian cancers and a promoter of lung cancer *via* regulating interactions with glycogen synthase kinase 3 and protein phosphatase 1.^39^,^47^ Recently, OLA1 was identified to directly interact with breast-cancer-associated gene 1 protein (BRCA1) and BRCA1-associated RING domain protein 1 (BARD1) to co-regulate centrosome formation.^45^,^49^ Thus, N-terminal methylation of OLA1 may modulate the aforementioned protein–protein interactions and consequently affect OLA1 functions in a way similar to the recently discovered eukaryotic elongation factor 1A methylation by METTL13.^50^,^51^

**Fig. 4:**
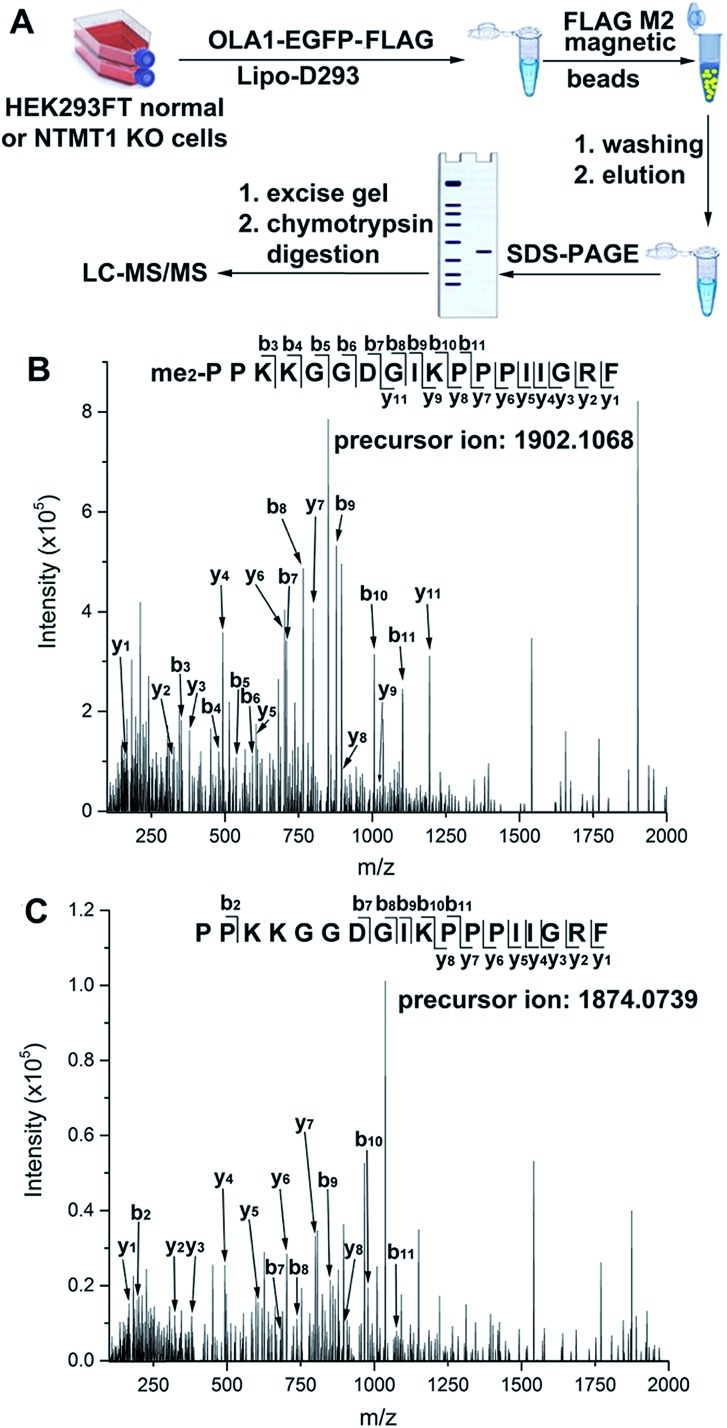
(A) Strategy to validate OLA1 N-terminal methylation *in vivo*. (B and C) Tandem MS spectra of dimethylated (B) and nonmethylated (C) peptide fragments obtained from normal and NTMT1 KO cells, respectively. This 18-mer peptide fragment corresponds to the first 18 N-terminal residues of OLA1 after methionine removal.

Normal and NTMT1 KO cells were transfected with *p*OLA1 containing dual C-terminal EGFP and FLAG tags. After the cell lysates were processed as shown in Fig. 4A, parent ions corresponding to N-terminal dimethylated and nonmethylated 18-mer peptides were observed in the normal and KO cells, respectively. Peptide mapping using tandem MS confirmed their identities (Fig. 4B and C), demonstrating that NTMT1 is responsible for OLA1 methylation *in vivo*. It is of note that all OLA1 was found to exist in the dimethylated format in normal cells, as the maximal degree of methylation for N-terminal proline is dimethylation. Since a positively charged residue at the 4^th^ position is critical for substrate recognition by NTMT1 (Fig. S15†),^31^,^32^ a K4Q-OLA1 mutant was constructed and confirmed not to be methylated by NTMT1 in normal cells (Fig. S16†). This indicates that the K4Q mutant could serve as a negative control for studying the function of OLA1 N-terminal methylation *in vivo*.

## Conclusions

We have synthesized Hey-SAM from SAH in ≥98% yield and used it with wt NTMT1 to perform target profiling. Seventy-two putative NTMT1 targets were discovered based on proteomics as well as motif sequence and signal peptide analyses, which include several known substrates and many unknowns. Target validation revealed that OLA1 is N-terminally methylated by NTMT1 *in vitro* and *in vivo*. While acetylation has been suggested for OLA1,^52^ to the best of our knowledge, the N-terminal methylation reported here represents the first confirmed post-translational modification for OLA1, a protein that has been demonstrated to directly interact with the BRCA1–BARD1 complex to co-regulate centrosome formation.^45^,^49^ Furthermore, this target profiling method can be adapted to a high-throughput screening format to identify NTMT1 inhibitors. Efforts to characterize the effect of N-terminal methylation on centrosome formation and to screen small-molecule inhibitors to NTMT1 are currently being undertaken in our laboratory.

## Conflicts of interest

There are no conflicts to declare.

## Supplementary Material

Supplementary information
