# Supplementary material for: In vivo methylation of OLA1 revealed by activity-based target profiling of NTMT1
Source: Chem Sci. 2019 Aug 9;10(35):8094–9. doi: 10.1039/c9sc02550b (PMC6889141; doi:10.1039/c9sc02550b)
Supplement: Supplementary file 1 [file SC-010-C9SC02550B-s001.pdf]

Electronic Supplementary Material (ESI) for Chemical Science.
This journal is © The Royal Society of Chemistry 2019

Supporting Information

In Vivo Methylation of OLA1 Revealed by Activity-Based Target Profiling of NTMT1

Kaimin Jia,[‡a] Gaochao Huang,[‡a] Wei Wu,[a] Ruben Shrestha,[a] Bingbing Wu,[a] Yulan Xiong,[b] and Ping Li[*a]

[a]Department of Chemistry, [b]Department of Anatomy and Physiology, Kansas State University, Manhattan, Kansas, United States, 66506

[‡]Denotes equal author contribution.
[*]To whom correspondence should be addressed: pli@k-state.edu

Table of Contents

1. General information…… S-2
2. Chemical synthesis of Hey-SAM…… S-2
3. Peptide synthesis…… S-4
4. Human cell culture…… S-4
5. Plasmid construction…… S-4
6. Protein expression and purification…… S-5
7. MS-based assay for RCC1 peptide and protein modifications…… S-5
8. Isothermal titration calorimetry…… S-6
9. Steady-state kinetics…… S-6
10. Peptide mapping of RCC1 purified from *E. coli*…… S-6
11. Click chemistry of labelled RCC1 and DBA…… S-7
12. Western blots…… S-7
13. Pull-down of overexpressed RCC1 from *E. coli* lysates…… S-8
14. Generation of NTMT1 KO cell line using CRISPR-Cas9…… S-8
15. Activity-based substrate profiling of NTMT1…… S-8
16. Motif and signal peptide analyses…… S-9
17. In vitro target validation…… S-9
18. In vivo methylation of OLA1…… S-9
19. References…… S-10
20. Scheme S1…… S-12
21. Figures S1-S16…… S-12
22. Tables S1-S4…… S-23
23. NMR spectra of synthesized compounds…… S-26
24. MS raw data of peptides and proteins…… S-53

1. General information

All chemical and biochemical reagents were purchased at the highest purity grade. Unless otherwise specified, all solvents were anhydrous, and all chemical syntheses were performed under argon atmosphere. Thin layer chromatography (TLC) was performed using 60 mesh silica gel plates and visualization was performed using short wavelength UV light (254 nm) and basic $KMnO_4$ staining. All samples for HPLC and mass spectrometry (MS) were filtered using 0.2 μm PTFE syringe or centrifugal filters to remove particles before injection. HPLC was performed with a Waters Breeze 2 system consisting of a 1525 pump and 2998 photodiode array detector. High-resolution MS (HRMS) was recorded on a Waters Xevo G2-XS QTof mass spectrometer. NMR spectra were recorded on a Varian 400 MHz or Bruker 600 MHz spectrometer. Chemical shifts of proton (^{1}H NMR) and carbon (^{13}C NMR) were reported in ppm relative to the peaks of residual solvents. Liquid chromatography coupled with tandem mass spectrometry (LC-MS/MS) was performed with a Waters Acquity H- or M-class UPLC connected to a Waters Xevo G2-XS QTof mass spectrometer, which were controlled by Waters MassLynx V4.1. Mobile phases of A and B for LC-MS were 0.1% formic acid in water and in acetonitrile, respectively. The expanded N-terminal sequences for motif analysis are listed in Table S1. Putative NTMT1 targets based on proteomics and motif analysis are listed in Table S2.

2. Chemical synthesis of Hey-SAM (Scheme S1 and Figure 1A)

*4-((tetrahydro-2H-pyran-2-yl)oxy)but-2-yn-1-ol **4**.*[S1-2] A solution of 3, 4-dihydro-2*H*-pyran (DHP, 16.8 g, 1.0 equiv.) in MeCN was added dropwise at room temperature to a suspension of **3** (86.0 g, 5.0 equiv.) and $CuSO_4•5H_2O$ (2.5 g, 0.05 equiv.) in MeCN. The mixture was stirred until DHP was completely consumed as determined by TLC. The reaction mixture was filtered by Celite and the filtrate was concentrated under a reduced pressure. The residue was purified by silica gel chromatography (hexane/EtOAc = 3/1) to give **4** as colorless oil (23.8 g, 70%).^{1}H NMR (400 MHz, $CDCl_3$) δ 4.80 (t, *J* = 3.4 Hz, 1H), 4.50–4.10 (m, 4H), 3.91–3.74 (m, 1H), 3.64–3.47 (m, 1H), 1.82–1.47 (m, 6H); ^{13}C NMR (101 MHz, $CDCl_3$) δ 96.86, 84.61, 81.32, 62.01, 54.39, 50.84, 30.18, 25.30, 18.95; HRMS: calcd. for $C_9H_{14}O_3Na^+$ $[M+Na]^+$: 193.0835, found: 193.0847.

*(E)-4-((tetrahydro-2H-pyran-2-yl)oxy)but-2-en-1-ol **5**.*[S3-4] To a solution of compound **4** (11.0 g, 1.0 equiv.) in THF (165 mL) was added dropwise at −78 °C a solution of 70 wt% sodium bis(2-methoxyethoxy)aluminium hydride (Red-Al) in toluene (37 g, 2.0 equiv.). The mixture was then kept stirring for 2 h at −78 °C and another 30 min at 0 °C. After the mixture was poured into ice, the precipitates formed were treated with aqueous 50% KOH (140 g, 19 equiv.) to obtain a clear solution. The aqueous layer was separated and then extracted twice with THF. The organic layer and extraction were combined, dried with $MgSO_4$, and concentrated under a reduced pressure. The residue was purified by silica gel chromatography (hexane/EtOAc = 3/1) to give **5** as colorless oil (8.2 g, 74%). ^{1}H NMR (400 MHz, $CDCl_3$) δ 6.05–5.65 (m, 2H), 4.64 (t, *J* = 3.6 Hz, 1H), 4.24 (dd, *J* = 12.5, 5.3 Hz, 1H), 4.15 (t, *J* = 5.2 Hz, 2H), 3.98 (dd, *J* = 12.6, 5.8 Hz, 1H), 3.89–3.82 (m, 1H), 3.54–3.46 (m, 1H), 1.89–1.43 (m, 6H); ^{13}C NMR (101 MHz, $CDCl_3$) δ 132.17, 127.47, 97.93, 67.06, 62.73, 62.16, 30.55, 25.40, 19.37; HRMS: calcd. for $C_9H_{16}O_3Na^+$ $[M+Na]^+$: 195.0992, Found: 195.1005.

*(E)-4-((tetrahydro-2H-pyran-2-yl)oxy)but-2-en-1-yl methanesulfonate **6**.*[S5] To a solution of compound **5** (11.4 g, 2.0 equiv.) and Et_3N (10.0 g, 1.5 equiv.) in CH_2Cl_2 (133 mL) was added methanesulfonyl chloride (9.86 g, 1.3 equiv.) at 0 °C. The mixture was kept stirring at 0 °C for 5 min and then quenched with saturated $NaHCO_3$ (aq). The organic layer was separated and concentrated under a reduced pressure. The residue was purified by silica gel chromatography (hexane/EtOAc = 3/1) to give **6** as colorless oil (15.6 g, 94%). ^{1}H NMR (400 MHz, $CDCl_3$) δ 6.02 (dt, *J* = 15.6, 5.1 Hz, 1H), 5.97–5.81 (m, 1H), 4.82–4.69 (m, 2H), 4.68–4.60 (m, 1H), 4.32–4.21 (m, 1H), 4.02 (dd, *J* = 13.8, 5.4 Hz, 1H), 3.90–3.80 (m, 1H), 3.56–3.47 (m, 1H), 3.01 (s, 3H), 1.87–1.51 (m, 6H); ^{13}C NMR (101 MHz, $CDCl_3$) δ 133.96, 123.71, 98.18,

69.74, 66.15, 62.17, 38.02, 30.45, 25.31, 19.31; HRMS: calcd. for $C_{10}H_{18}O_5SNa^+$ $[M+Na]^+$: 273.0767, Found: 273.0776.

(E)-trimethyl(6-((tetrahydro-2H-pyran-2-yl)oxy)hex-4-en-1-yn-1-yl)silane 7.[S6-7] To a flame-dried flask containing NaI (30.0 g, 2.0 equiv.), CuI (19.0 g, 1.0 equiv.), and K_2CO_3 (27.6 g, 2.0 equiv.) was added compound **6** (25 g, 1.0 equiv.), anhydrous DMF (200 mL), and trimethylsilylacetylene (12.1 g, 1.2 equiv.). After the reaction mixture was stirred at room temperature overnight, it was diluted with CH_2Cl_2 (600 mL) and then centrifuged. The solvent/supernatant was concentrated under high vacuum using an oil pump and liquid nitrogen trap. The residue was purified by silica gel chromatography eluted with 100% CH_2Cl_2 to give **7** as colorless oil (6.0 g, 24%). ^{1}H NMR (400 MHz, $CDCl_3$) δ 5.97–5.78 (m, 1H), 5.69 (dt, *J* = 15.4, 5.3 Hz, 1H), 4.63 (t, *J* = 3.7 Hz, 1H), 4.22 (dd, *J* = 12.4, 5.5 Hz, 1H), 3.97 (dd, *J* = 12.4, 6.4 Hz, 1H), 3.90–3.82 (m, 1H), 3.54–3.45 (m, 1H), 3.00 (d, *J* = 5.5 Hz, 1H), 1.88–1.79 (m, 1H), 1.75–1.69 (m, 1H), 1.62–1.49 (m, 4H), 0.15 (s, 9H); ^{13}C NMR (101 MHz, $CDCl_3$) δ 128.30, 127.36, 103.72, 98.02, 86.90, 67.26, 62.34, 30.73, 25.58, 23.04, 19.62, 0.21; HRMS: calcd for $C_{14}H_{24}O_2SiH^+$ $[M+H]^+$: 253.1618, Found: 253.1627.

(E)-6-(trimethylsilyl)hex-2-en-5-yn-1-ol ***8****.*[S6] To a solution of compound **7** (6.0 g, 1.0 equiv.) in MeOH (20 mL) was added pyridinium p-toluenesulfonate (PPTS, 0.3 g, 0.05 equiv.). The mixture was heated to reflux until compound **7** was completely consumed as determined by TLC. The reaction was quenched with saturated $NaHCO_3$ (aq). The crude product was extracted by CH_2Cl_2. The extractions were combined, dried with anhydrous $MgSO_4$, and concentrated to dryness under a reduced pressure. The residue was purified by silica gel chromatography (EtOAc/CH_2Cl_2 = 1/9) to give **8** as colorless oil (3.5 g, 87%). ^{1}H NMR (400 MHz, $CDCl_3$) δ 5.91 (dtt, *J* = 15.0, 5.6, 1.7 Hz, 1H), 5.68 (dtt, *J* = 15.0, 5.1, 1.3 Hz, 1H), 4.15 (s, 2H), 3.01 (dt, *J* = 5.4, 1.6 Hz, 2H), 1.44 (br, 1H), 0.16 (s, 9H); ^{13}C NMR (101 MHz, $CDCl_3$) δ 130.95, 126.01, 103.64, 87.00, 63.14, 22.89, 0.18; HRMS: calcd. for $C_9H_{17}OSiH^+$ $[M+H]^+$:169.1043, Found: 169.1035.

(E)-hex-2-en-5-yn-1-ol ***9****.* To a solution of compound **8** (1.30 g, 1.0 equiv.) in THF (15 mL) was added TBAF (1M solution in THF, 9.3 mL, 1.2 equiv.) at 0 °C. The mixture was kept stirring until compound **8** was completely consumed as determined by TLC. The reaction was quenched with saturated NH_4Cl (aq). After the THF was removed under reduce pressure, the crude product was extracted by CH_2Cl_2. The extractions were combined, dried with anhydrous $MgSO_4$, and concentrated to dryness under a reduced pressure. The residue was purified by silica gel chromatography (EtOAc/CH_2Cl_2 = 1/9) to give **9** as colorless oil (0.7 g, 90%). ^{1}H NMR (400 MHz, $CDCl_3$) δ 5.94 (dtt, *J* = 15.0, 5.6, 1.8 Hz, 1H), 5.69 (dtt, *J* = 15.2, 5.4, 1.4 Hz, 1H), 4.14 (d, *J* = 5.6 Hz, 2H), 2.97 (ddt, *J* = 6.8, 2.8, 1.3 Hz, 2H), 2.11 (t, *J* = 2.7 Hz, 1H), 1.60 (br, 1H); ^{13}C NMR (101 MHz, $CDCl_3$) δ 131.14, 125.50, 81.31, 70.56, 62.87, 21.38; HRMS: calcd. for $C_6H_9OH^+$ $[M+H]^+$: 97.0648, Found: 97.0640.

(E)-hex-2-en-5-yn-1-yl methanesulfonate (Hey-OMs) ***2****.* The reaction and silica gel chromatography were performed in the dark in the same way as **6** using **9** (0.67 g, 1.0 equiv.), Et_3N (1.40 g, 2.0 equiv.), and methanesulfonyl chloride (1.20 g, 1.5 equiv.) in CH_2Cl_2 (7 mL) to yield **2** as colorless oil (3.5 g, 95%). ^{1}H NMR (400 MHz, $CDCl_3$) δ 6.12–5.74 (m, 2H), 4.72 (d, *J* = 6.6 Hz, 2H), 3.06–2.95 (m, 5H), 2.15 (t, *J* = 2.6 Hz, 1H); ^{13}C NMR (101 MHz, $CDCl_3$) δ 131.66, 124.30, 71.44, 69.73, 38.24, 21.43; HRMS: calcd. for $C_7H_{10}O_3SNa^+$ $[M+Na]^+$: 197.0243, Found: 197.0238.

((S)-3-amino-3-carboxypropyl)(((2S,3S,4R,5R)-5-(6-amino-9H-purin-9-yl)-3,4 dihydroxytetra-hydro-furan-2-yl)methyl)((E)-hex-2-en-5-yn-1-yl)sulfonium methanesulfonate (Hey-SAM) ***1****.*[S8] To a solution of *S*-adenosyl-*L*-homocysteine (SAH, 10 mg, 1.0 equiv.) in 88-90% HCOOH (20 μL) was added freshly prepared compound **2** (22.6 mg, 5.0 equiv.) at 0 °C. The mixture was kept stirring in the dark at 0 °C under Ar protection for 4 h followed by the addition of a second batch of **2** (22.6 mg, 5.0 equiv.). After the mixture was kept stirring for another 16 h under the same condition, water (1.0 mL) was added and then

extracted with CH_2Cl_2 (3.0 mL) for three times. The aqueous layer was analyzed by HPLC using an X-Bridge Shield RP18 column (5.0 µm, 4.6 × 250 mm, Waters) eluted with water containing 0.03% TFA at the flow rate of 1.0 mL/min. A typical HPLC profile is shown in Figure 1B. Since the purity of Hey-SAM was ≥98%, it was used for study without further purification.

Partial separation of the two epimers were performed with a Luna C18(2) column (5 µm, 100 Å, 10 × 250 mm, Phenomenex) eluted with water containing 0.03% TFA at the flow rate of 3.0 mL/min. The fractions were pooled and lyophilized to yield two samples of epimer mixtures, in which ratios of fast- and slow-eluting epimers were at 7:3 and 4:6, respectively, based on NMR analysis.

*Fast- and slow-eluting epimers of Hey-SAM at **7:3 ratio**.* ^{1}H NMR (600 MHz, D_2O) δ 8.41–8.39 (m, 2H), 6.11 (m, 1H), 6.06 (dt, J = 15.4, 5.1 Hz, 0.7H), 5.91 (dt, J = 15.2, 5.2 Hz, 0.3H), 5.77 (dt, J = 15.1, 7.1Hz, 0.7H), 5.67 (dt, J =15.0, 7.5 Hz, 0.3H), 4.76 (t, J = 6.6 Hz, 1H), 4.60–4.56 (m, 1H), 4.51–4.47 (m, 1H), 4.13 (d, J = 7.6 Hz, 1.4H), 4.09 (d, J = 7.7 Hz, 0.6H), 3.97 (m, 0.3H), 3.92 (m, 0.7H), 3.85–3.81 (m, 1.4H), 3.79–3.76 (m, 0.6H), 3.55–3.47 (m, 1H), 3.46–3.37 (m, 1H), 3.02(d, J = 3.0 Hz, 1.4H), 2.94 (d, J = 4.8 Hz, 0.6H), 2.49 (s, 0.7H), 2.45 (s, 0.3H), 2.37–2.27 (m, 2H); ^{13}C NMR (151 MHz, D_2O) δ 171.92, 151.27, 149.19, 145.72, 144.83, 140.59, 120.65, 116.93, 91.43, 81.80, 79.89, 74.31, 73.95, 73.62, 52.76, 43.63, 42.43, 36.75, 26.32, 22.33; HRMS: calcd. for $C_{20}H_{27}N_6O_5S^+$ $[M+H]^+$: 463.1758, Found: 463.1776.

*Fast- and slow-eluting epimers of Hey-SAM at **4:6 ratio**.* ^{1}H NMR (600 MHz, D_2O) δ 8.41–8.39 (m, 2H), 6.11 (m, 1H), 6.06 (dt, J = 15.4, 5.1 Hz, 0.4H), 5.91(dt, J = 15.2, 5.2 Hz, 0.6H), 5.77 (dt, J = 15.1, 7.1Hz, 0.4H), 5.67 (dt, J =15.0, 7.5 Hz, 0.6H), 4.76 (t, J = 6.6 Hz, 1H), 4.60–4.56 (m, 1H), 4.51–4.47 (m, 1H), 4.13 (d, J = 7.6 Hz, 1.4H), 4.09 (d, J = 7.7 Hz, 0.6H), 3.97 (m, 0.6H), 3.92 (m, 0.4H), 3.85–3.81 (m, 1.4H), 3.79–3.76 (m, 0.6H), 3.55–3.47 (m, 1H), 3.46–3.37 (m, 1H), 3.02 (d, J = 3.0 Hz, 1.4H), 2.94(d, J = 4.8 Hz, 0.6H), 2.49 (s, 0.4H), 2.45 (s, 0.6H), 2.37–2.27 (m, 2H); ^{13}C NMR (151 MHz, D_2O) δ 171.96, 151.31, 149.25, 145.78, 144.74, 140.63, 120.59, 116.68, 91.34, 81.72, 79.92, 74.33, 74.13, 73.49, 52.68, 42.88,-42.03, 36.51, 26.23, 22.29.

3. Peptide synthesis

Peptide analogs of N-terminal RCC1 [SPKRIAKRRS($CONH_2$)], PB1 [GSKRRRATSP($CONH_2$)], SPD2B [PPRRSIVEVK($CONH_2$)], DDX60L [GSKDHAVFFR($CONH_2$)], H2A1B [SGRGKQGGKA($CONH_2$)], FECH [GAKPQVQPQK($CONH_2$)], LAMC2 [TSRREV(COOH)], OLA1 [PPKKGGDGIK($CONH_2$)], and K4Q-OLA1 [PPQKGGDGIK($CONH_2$)] were synthesized on a Rink amid or 2-chlorotrityl chloride resin (Peptides International) using standard Fmoc chemistry.[S9] Peptides were purified by semi-preparative HPLC, and their purity and identities were confirmed by analytical HPLC and HRMS, respectively. Conditions of semi-preparative and analytical HPLC are summarized in Table S3.

4. Human cell culture

Normal and NTMT1 KO HEK293FT cells were cultured in Dulbecco's modified Eagle's medium (DMEM, Corning, 10-013-CV, containing 4.5 g/L glucose, L-glutamine and sodium pyruvate) supplemented with 10% fetal bovine serum (FBS, Gibco, 16140089) and 1% penicillin-streptomycin (Gibco, 30-002-CI) in a humidified atmosphere containing 5% CO_2 at 37 °C.

5. Plasmid construction

Sequences of primers, restriction sites, tags, and vectors used to construct plasmids are summarized in Table S4. Plasmids were constructed following a standard protocol.[S10] The gene corresponding to human NTMT1 was synthesized by GenScript. Human RCC1 gene was amplified from *p*ST50Tr-hRCC1 (a gift from Prof. Song Tan at Penn State)[S11]. Genes corresponding to RS14 and OLA1 were amplified

from *c*DNA of HEK293FT cell line. Mammalian expression vector *p*EGFP-n1 was modified to introduce a FLAG tag at the C terminus of EGFP, yielding a new vector *p*EGFP-n1-FLAG. Plasmids to express OLA1 and K4Q-OLA1 in mammalian cells were constructed using *p*EGFP-n1-FLAG. To generate an NTMT1 KO cell line using CRISPR-Cas9, guide sequences were designed according to an online CRISPR Design Tool (http://tools.genome-engineering.org).[S12] Gene insertions were verified by DNA sequencing.

6. Protein expression and purification

Buffers used for protein purification are listed here. For purification of NTMT1, RCC1 and RS14, buffer A consisted of 300 mM NaCl and 5 mM imidazole in 25 mM HEPES (pH 7.4). Buffers B and C were the same as the buffer A except that the imidazole concentrations were 30 and 250 mM, respectively. Buffer D consisted 150 mM NaCl and 50 mM KCl in 25 mM HEPES (pH 7.4). For OLA1 purification,[S13] buffer A consisted of 700 mM NaCl, 5 mM $MgCl_2$ and 10% glycerol in 50 mM Tris (pH 7.9). Buffer B consisted of 100 mM NaCl, 5 mM $MgCl_2$, 5% glycerol, and 40 mM imidazole in 50 mM Tris (pH 7.9). Buffer C was the same as the buffer B except that the imidazole concentration was 250 mM. Buffer D consisted of 100 mM NaCl in 50 mM Tris (pH 7.4).

All proteins were overexpressed in *Escherichia coli* BL21(DE3) (Lucigen) except that RCC1 was overexpressed in *E. coli* C41(DE3) (Lucigen). Bacteria were grown in 4L of Luria Bertani (LB) media in the presence of 50 μg/mL kanamycin. When A_{600} reached 0.6–0.8, isopropyl-1-thio-β-D-galactopyranoside (IPTG) was added to a final concentration of 0.4 mM to induce protein expression. Bacteria were grown at 18 °C for additional 18 h and then harvested by centrifugation at 5,000 *g* for 20 min at 4°C. The cell pellets were collected and stored at −80 °C until further use.

All the following purification steps were carried out at 4 °C. The cell pellets were re-suspended in buffer A and lysed by sonication for 15 min (5-second on and 3-second off) at 40% amplitude. The cell debris was removed by centrifugation at 18,000 *g* for 45 min. The supernatant was decanted and incubated with Ni-NTA resin (Qiagen) that had been pre-equilibrated with buffer A for 30 min. The resin was washed with 30 column volumes (CV) of buffer B and then eluted with 5CV of buffer C. Fractions containing the targeted protein were collected and exchanged into buffer D using a HiPrep™ 26/10 desalting column (GE Healthcare). Purified proteins were concentrated to 5–20 mg/ml, flash-freezed in liquid N_2, and stored in aliquots at −80 °C until further use. Protein purity was assessed by 12% tris-tricine SDS-PAGE (Figure S1), and their concentrations were determined by bicinchoninic acid assays (BCA) using a BSA calibration curve.[S14]

7. MS-based assay for RCC1 peptide and protein modifications

RCC1 peptide ($RCC1_p$) concentration was determined at 205 nm using a calculated extinction coefficient of 29,070 M^{-1} cm^{-1}. Modifications of RCC1 at peptide and protein levels by *wt* NTMT1 were investigated using MS in the presence of cofactors. Specifically, 20 μM $RCC1_p$ in 10 mM ammonium bicarbonate (ABC) or RCC1 in 25 mM HEPES (pH 7.4) containing 150 mM NaCl and 50 mM KCl, 200 μM SAM or Hey-SAM, and 3 μM *wt* NTMT1 were incubated at 37 °C for 2 h. Negative controls were performed in the absence of *wt* NTMT1. The $RCC1_p$ modification reaction was quenched with an equal volume of water containing 0.1% formic acid and then analyzed by LC-MS using a BEH C18 analytical column (1.7 μm, 130 Å, 2.1 mm × 50 mm, Waters) eluted at a flow rate of 0.4 mL/min. The gradient of B increased from 0 to 90% in 2.5 min. The RCC1 protein modification reaction was not quenched and directly analyzed by intact protein MS, which was performed on a BEH C4 analytical column (1.7 μm, 300 Å, 2.1 mm × 50 mm, Waters) eluted at a flow rate of 0.4 mL/min. The gradient was: 0–1 min, 5% B; 1–3.5 min, 5–100% B; and 3.5–4.7 min, 100% B. Elution from 2–4.7 min was injected into the mass spectrometer. The data were processed by Waters MassLynx and BiopharmaLynx. Results are summarized in Figures 1C-D, S2, and S3A-E.

8. Isothermal titration calorimetry (ITC)

ITC measurements were performed with MicroCal iTC200 (GE Healthcare) at 25 °C. Purified *wt* NTMT1 was diluted to 50 μM using the buffer consisting of 150 mM NaCl and 50 mM KCl in 25 mM HEPES (pH 7.4). Cofactors or RCC1 peptide substrate was dissolved and diluted to 300–500 μM using the same buffer. Measurements were carried out with 18 injections of 2 μL with an interval time of 180 s and a reference power of 7 μcal/s. Experiments were done in triplicate and data were analyzed by Origin software. The results are shown in Figures 2A-C.

9. Steady-state kinetics

SAH release monitored by HPLC was used to determine steady-state kinetics. Adenosine was added as an internal standard to quantify the SAH formation. Experiments were performed by varying concentrations of one substrate in the presence of the other substrate at saturation. Specifically, concentrations of SAM and Hey-SAM varied from 0.1 to 8 μM and from 0.2 to 16 μM, respectively, in the presence of 40 μM $RCC1_p$. Concentration of $RCC1_p$ varied from 0.5 to 16 μM in the presence of 20 μM SAM or Hey-SAM.

Experiments were carried out in the following way. In a final volume of 400 μL, 0.3 μM *wt* NTMT1 was incubated with a cofactor and $RCC1_p$ at 37 °C in the buffer consisting of 50 mM KCl in 25 mM Tris (pH 7.5). Aliquots of 40 μL were withdrawn at selected time intervals (1 to 90 min) and the reactions were quenched with 40 μL of 0.4% TFA solution containing 4.0 μM adenosine. The reaction mixtures were then centrifuged at 18,000 *g* for 2 min. The supernatants were removed and 40 μL were injected into an XBridge Shield RP 18 column (3.5 μm, 130 Å, 4.6 × 150 mm, Waters). The column was eluted at 1 mL/min with an isocratic gradient of 1% methanol in water supplemented with 0.01% TFA. Measurements were done in triplicate. Concentrations of released SAH were determined by A_{260} ratios of the peak areas corresponding to SAH and adenosine. The data obtained were fitted into the Michaelis-Menten equation ($v = \frac{V_{max}[S]}{K_M+[S]}$) using SigmaPlot. Results are shown in Figures 2D-E and summarized in Table 1.

10. Peptide mapping of RCC1 purified from *E. coli*

To map the site of RCC1 modified by Hey-SAM in the presence of *wt* NTMT1, the reaction mixture was separated by SDS-PAGE. The protein band corresponding to RCC1 was cut out and treated with in-gel trypsin digestion following a standard protocol.[S15] Briefly, the RCC1 gel band was cut into 1.0 mm^3 pieces, reduced with 100 mM dithiothreitol (DTT), and then alkylated with 55 mM iodoacetamide. Digestion was implemented at 37 °C for 18 h with 20 ng/μL sequencing-grade trypsin (Promega) in 10 mM ABC containing 10% (vol/vol) acetonitrile. The peptides were extracted with gentle shaking at 37 °C for 1 h using a water acetonitrile mixture (1:1, vol/vol) containing 5% formic acid. The peptide extraction was concentrated by SpeedVac and analyzed by LC-MS/MS.

For LC−MS/MS analysis, the digestion products were re-dissolved in water containing 5% formic acid and then separated on a BEH C18 column (1.7 μm, 130 Å, 2.1 mm × 50 mm, Waters) eluted at 0.2 mL/min. The gradient of mobile phase B increased from 0 to 50% in 50 min. The mass spectrometer was operated in an MS^E mode with a ramp collision energy ranging from 20 to 45 V. Data were analyzed by BiopharmaLynx (Waters), for which the searching parameters were set at peptide mass tolerance of 10 ppm, MS^E mass tolerance of 30 ppm, and no missed cleavages allowed. The fixed modification of cysteine carbamidomethylation and variable modification of N-terminal chain by Hey were also used in the data searching. A precursor ion corresponding to the Hey-alkylated SPK tripeptide (m/z: 409.2451) was detected. Further fragmentation of this precursor ion gave y_2 and y_3 ions, confirming the N-terminus of methionine-removed RCC1 was modified by Hey (Figures S3F).

11. Click chemistry of labelled RCC1 and DBA

In a final volume of 200 µl, 20 µM purified RCC1 was incubated overnight at 4 °C with 50 µM Hey-SAM and 3 µM *wt* NTMT1 in the buffer consisting of 150 mM NaCl and 50 mM KCl in 25 mM HEPES (pH 7.4). Negative controls were obtained either using SAM to substitute Hey-SAM or in the absence of *wt* NTMT1. Proteins were then precipitated out using 1.0 mL cold methanol and the resultant samples were centrifuged at 14,100 *g* for 2 min. After the supernatant was removed, protein pellets were washed with 1.0 mL cold methanol, air-dried for 15 min, and re-dissolved in 200 µL buffer E consisting of 150 mM NaCl and 1% SDS in 25 mM HEPES (pH 7.4). A total volume of 22 µL click chemistry cocktail [one volume of 5 mM diazo biotin azide (DBA) and ten volumes of premixed solution consisting of 1 mM tris(benzyltriazolylmethyl)amine (TBTA), 10 mM $CuSO_4$, and 10 mM tris(2-carboxyethyl)phosphine (TCEP) with the pH adjusted to 7.0 using 1 M NaOH] was added into the samples and the click reaction was performed in the dark for 2 h at room temperature.[S16] The samples were mixed with 1.0 mL methanol and kept overnight at −80 °C. The precipitated proteins were washed with 1.0 mL cold methanol and re-dissolved in 100 µL buffer E. Presence of RCC1 and biotin were confirmed by western blot (WB) as described below in Section 12 using rabbit anti-RCC1 antibody (Abcam) and streptavidin-HRP (SA-HRP, Abcam), respectively. Results are shown in Figures S4A-B.

To further confirm the purified RCC1 had been N-terminally biotinylated using click chemistry, we decided to perform LC-MS/MS analysis. Gel shift assay was employed to enrich the biotinylated RCC1, as only a fraction of recombinant RCC1 purified from *E. coli* could serve as the active NTMT1 substrate. It is of note that RCC1 produced from *E. coli* exists in three formats: full-length, N-terminal methionine removed, and N-terminal methionine and serine removed. Among them, only the RCC1 with the methionine removed is active and can be alkylated by *wt* NTMT1. The amount of active RCC1 varies between 30 and 50% of the total protein. To eliminate the interference of the inactive formats of RCC1 in MS, a gel shift assay using streptavidin (SA) was performed. Briefly, 10 µL of the above re-dissolved protein samples after click chemistry were mixed with 10 µL of 2 × Laemmli buffer and heated at 100 °C for 5 min. After cooling down to room temperature, the samples were incubated with 15 µL of 1 mg/ml SA solution (NEB) at room temperature for 30 min.[S17] Protein mixtures were then separated by SDS-PAGE. Native SA exists as a mixture of tetramer and dimer at 52.8 and 26.4 KDa, respectively (Lane 3 in Figure S4A). After it was added to the controls and reaction (Lanes 7-9 in Figure S4A), only the reaction sample formed two new bands at 75 and 150 KDa (arrows in Figure S4A), corresponding to RCC1+2SA and 2RCC1+4SA, respectively. These two new bands were cut out, treated with trypsin, and then analyzed by LC-MS/MS as described above in Section 10. A precursor ion corresponding to the biotinylated SPK tripeptide (m/z: 1120.5615) was detected. Further fragmentation of the precursor ion gave b_1, y_2, and y_4 ions, confirming the N-terminus of methionine-removed RCC1 was modified by a biotin tag (Figures S4C).

12. Western blots

Equal amounts of cell lysates or protein samples were separated in a 12% SDS-PAGE and then transferred to a supported nitrocellulose membrane (Bio-Rad). Immunoblotting was performed overnight at 4 °C with a rabbit anti-RCC1 antibody (Abcam), rabbit anti-OLA1 antibody (Bethyl Laboratories), rabbit anti-NTMT1 antibody (Abcam), rabbit anti-actin antibody (Sigma), or streptavidin-HRP (Abcam) at 1:1000-1:3000 dilutions. Membrane was washed with TBS buffer (pH 7.4) containing 0.1% Tween 20 (TBS-T). If the primary antibodies did not conjugate with HRP, then they would be recognized by an HRP-conjugated goat anti-rabbit antibody (Abcam, 1:5000 dilutions) by gentle shaking for 1 h at room temperature. After washing membrane with TBS-T buffer, bands were detected using SuperSignal West Pico PLUS chemiluminescent substrate (Thermo Fisher Scientific) following manufacturer's protocol. The bands were visualized using Amersham Imager 600 (GE Healthcare).

13. Pull-down of overexpressed RCC1 from *E. coli* lysates

E. coli C41(DE3) cells transformed with *p*RCC1 were grown and harvested as described above in Section 6. They were re-suspended in a buffer consisting of 300 mM NaCl in 25 mM HEPES (pH 7.4) and sonicated for 15 min (5-second on and 3-second off) at 40% amplitude. After centrifugation at 18,000 *g* for 45 min, 1 mL supernatant was withdrawn and incubated with 50 μM Hey-SAM and 3 μM *wt* NTMT1 overnight at 4 °C. Negative controls were performed either using SAM to substitute Hey-SAM or in the absence of *wt* NTMT1. Proteins were precipitated out using 4.0 mL cold methanol, air-dried for 15 min, and re-dissolved in 4.0 mL buffer E (Section 11). Click chemistry was performed with 440 μL click chemistry cocktail (Section 11) in the dark for 2 h at room temperature.[S16] After proteins were precipitated out using 16.0 mL cold methanol and re-dissolved in 1.0 mL PBS (1×, pH 7.4) containing 1% SDS, 60 μL SA beads (GE Healthcare) prewashed with PBS (1×, pH 7.4) were added and incubated with the protein mixtures for 2 h at room temperature with gentle shaking. Samples were centrifuged at 4,000 *g* for 2 min and the unbound proteins were pipetted out. The SA beads were then washed sequentially with PBS (1×, pH 7.4) containing 1% SDS, PBS (1×, pH 7.4) containing 4 M urea, and 100 mM ABC, each for three times. Finally, the biotin labelled proteins were cleaved and eluted from the beads using 60 μL 25 mM $Na_2S_2O_4$ in 20 mM ABC. The elution was mixed with 2 × Laemmli buffer and analyzed by SDS-PAGE (Figure S5A). Presence of RCC1 was detected and quantified by WB as described above in Section 12 using rabbit anti-RCC1 (Abcam) and goat anti-rabbit antibodies (Abcam) as the primary and secondary antibodies, respectively (Figure S5B). To calculate the recovery yield, a standard curve of signal versus protein amount was generated using a series dilution of purified RCC1 from *E. coli* at a known concentration (Figure S5C).

14. Generation of NTMT1 KO cell line using CRISPR-Cas9

NTMT1 knockout (KO) HEK293FT cell line was generated according to the published protocol.[S12] Briefly, plasmids of PX459-NTMT1$_{sgRNA1}$ and PX459-NTMT1$_{sgRNA2}$ (1 μg each) were transfected into HEK293FT cells pre-seeded in a 6-well plate using LipoD293 (SignaGen Laboratories) and Opti-MEM™ I reduced serum medium (ThermoFisher) when cell confluency reached to 50-70%. After 36 h, the transfected cells were treated with 3 μg/mL puromycin for one week. Cells were then dissociated with TrypLE™ express enzyme (ThermoFisher), counted, and serially diluted in DMEM supplemented with 20% FBS to a final cell density of 50-100 cells per 15 mL medium to reduce the possibility of having multiple cells in one well. The diluted cells were plated to two 96-well plates and cultured for additional two weeks. After passaging into a 24-well plate, cells were screened by WBs (Figure S6). For the cells without detection of NTMT1, their genomic DNAs were extracted with a tissue DNA kit (Omega, D3396-01), and the NTMT1 exon 1 was amplified and sequenced. The KO cell lines confirmed by DNA sequencing were frozen in liquid N_2.

15. Activity-based substrate profiling of NTMT1

NTMT1 KO cells were cultured as described in Section 4. At 70%–90% confluence stage, cells (4 of 175 cm^2 flasks) were harvested and lysed by sonication for 8 min (5-second on and 3-second off) at 40% amplitude in 9 mL cold buffer consisting of 50 mM KCl, 10% glycerol, and 1× protease inhibitor (Pierce) in 25 mM HEPES (pH 7.4). Cell lysates were centrifuged at 15,000 *g* for 15 min at 4 °C to remove cell debris. The supernatant was incubated with 50 μM Hey-SAM or SAM (negative control) in the presence of 3 μM *wt* NTMT1 for 4 h at 37 °C. Four-time volumes of cold methanol were added into each sample and kept at −80 °C overnight to precipitate proteins. The samples were centrifuged at 7,197 *g* for 30 min and the supernatant was removed. The protein pellets were air-dried for 15 min and re-dissolved in 4.0 mL buffer E (Section 11). Click chemistry and subsequent treatment were performed in the same way as

described above in Section 13. As a positive control, presence of RCC1 was detected by WB (Figure 3C) as described above in Section 12.

Lanes of samples treated with Hey-SAM and SAM (negative control) were excised and digested with trypsin following the procedures described above in Section 10. For LC-MS/MS analysis, protein digestion products were re-dissolved in water containing 1% formic acid, filtered, and injected into Acquity M-class UPLC coupled with Xevo G2-XS QTof high-resolution mass spectrometer (Waters). The injected peptides were first passed through a Symmetry trap column (C18, 100 Å, 5 µm, 300 µm × 50 mm, Waters), and then separated on an HSS T3 column (C18, 100 Å, 1.8 µm, 300 µm × 100 mm, Waters), which were eluted at 8 µL/min. The gradient of mobile phase B for separation increased from 1% to 40% in 45 min. The MS was obtained under a positive polarity and sensitivity mode. The tandem MS was recorded under an MS^E mode with a ramp collision energy ranging from 20 to 45 V. Raw MS data were analyzed by Progenesis QI for Proteomics (Waters) to give protein candidates, for which they had to pass (1) the Anova p value ≤ 0.05 and (2) max fold change (Hey-SAM treated sample/SAM treated sample) ≥ 2. Experiments were done in triplicate.

16. Motif and signal peptide analyses

NTMT1 was previously reported to methylate proteins with an N-terminal sequence of Xaa (Ala/Pro/Ser)-Pro-Lys after methionine removal.[S18] Additional studies revealed that the 2nd residue Xaa can also be replaced with other residues except for hydrophobic Leu/Ile/Trp and negatively charged Asp/Glu residues.[S19-20] *In vitro* peptide methylation assays indicated that the proline at the 3rd position could be substituted with asparagine, glycine, alanine, methionine, serine, or threonine to some extent.[S19] The 4th residue was demonstrated to be only limited to a positively charged lysine or arginine, as it is critical for the formation of hydrogen bonds with the substrate binding pocket in NTMT1.[S20-21] Based on these expanded N-terminal sequence motifs (Table S1), a search of the human protein database using https://www.genome.jp/tools/motif/MOTIF2.html returned 733 potential NTMT1 targets.

Protein targets identified by Progenesis QI were further analyzed using UniProt online tools (https://www.uniprot.org/uploadlists/) to find proteins that may undergo N-terminal truncation. Signal and transit peptides should be selected for PTM processing in the UniProt. After the proteins were identified and signal sequences were manually removed, the resulting matured proteins were then subject to motif analysis as described above.

17. *In vitro* target validation

As described above in Section 7, MS-based methylation assays were carried out with the N-terminal peptide analogs of PB1, SPD2B, DDX60L, H2A1B, FECH, and LAMC2, as well as the recombinant proteins of RS14 and OLA1. Peptide data and intact protein mass were analyzed by MassLynx (Waters) and BiopharmaLynx (Waters), respectively. Results are shown in Figures S7-15.

18. *In vivo* methylation of OLA1

Normal and NTMT1 KO cells were cultured as described in Section 4. At 70-90% confluence stage, cells were transfected with 20 µg *p*OLA1-EGFP-FLAG or *p*K4Q-OLA1-EGFP-FLAG using LipoD293 (SignaGen Laboratories). After 36 h, cells were harvested, re-suspended in 3 mL PBS (1×, pH 7.4), and lysed by sonication for 4 min (5-second on and 3-second off) at 40% amplitude. The cell debris was removed by centrifugation at 15,000 *g* for 15 min and the supernatant was incubated with 30 µL FLAG-M2 magnetic beads (Sigma) for 4 h at 4 °C. After washing the magnetic beads with PBS buffer (1×, pH 7.4) containing 0.2% Triton X-100 for three times, the FLAG-tagged protein was eluted with 60 µL of 200 mM glycine (pH 2.5). The pH of the elution was adjusted to ~7 using 1 M Tris (pH 7.9). The eluted protein was first reduced by 6 µL buffer consisting of 10% SDS and 20 mM TCEP in 500 mM Tris (pH 7.9) for 10 min at 37 °C and then alkylated with 100 mM Tris buffer (pH 7.9) containing 100 mM

iodoacetamide for 15 min at 37 °C.[S22] The resulting sample was concentrated to ~ 10 μL by SpeedVac, mixed with 10 μL of 2 × Laemmli buffer, and heated at 100 °C for 5 min before it was loaded onto a gel for SDS-PAGE. After gel staining and de-staining, the band corresponding to OLA1-EGFP-FLAG or K4Q-OLA1-EGFP-FLAG (~73kD) was excised, cut into small pieces, and washed with a mixture of 100 mM ABC and acetonitrile (1:1, vol/vol) at 37 °C to completely de-stain the gel. After shrunk and dehydrated with acetonitrile, the gel pieces were incubated with chymotrypsin [Promega, 200 ng diluted in 20 μL buffer consisting of 10 mM $CaCl_2$ in100 mM Tris (pH 7.9)] for 18 h at 25 °C. The digested peptides were extracted sequentially with 100 μL of 5% formic acid in water, a mixture of acetonitrile and water (1:1, vol/vol) containing 5% formic acid, and pure acetonitrile. The extracts were combined and concentrated to dryness by SpeedVac. The residue was processed and analyzed by LC-MS/MS in the same way as described in Section 15 except that the gradient of mobile phase B increased from 1% to 40% in 40 min. The data obtained were mapped to the primary structure of OLA1 or K4Q-OLA1 by BiopharmaLynx (Waters). Peptide fragmentation patterns were analyzed manually and results are shown in Figures 4B-C and S16.

19. References

S1. Khan, A. T.; Choudhury, L. H.; Ghosh, S. Cupric sulfate pentahydrate (CuSO4· 5H2O): A mild and efficient catalyst for tetrahydropyranylation/depyranylation of alcohols and phenols. *Tetrahedron Lett.* **2004**, *45*, 7891-7894.

S2. Nicolaus, N.; Strauss, S.; Neudörfl, J.-M.; Prokop, A.; Schmalz, H.-G. A [2+ 2+ 2]-cycloaddition approach toward 6-oxa-allocolchicinoids with apoptosis-inducing activity. *Org. Lett.* **2009**, *11*, 341-344.

S3. Caldwell, J. J.; Cameron, I. D.; Christie, S. D.; Hay, A. M.; Johnstone, C.; Kerr, W. J.; Murray, A. Total synthesis of Japanese Hop ether using an efficient intramolecular Pauson-Khand reaction. *Synthesis* **2005**, 3293-3296.

S4. Pena, J.; Moro, R. F.; Basabe, P.; Marcos, I. S.; Diez, D. Solvent free l-proline-catalysed domino Knoevenagel/6π-electrocyclization for the synthesis of highly functionalised 2*H*-pyrans. *RSC Adv.* **2012**, *2*, 8041-8049.

S5. Tonn, C.; Palazón, J.; Ruiz-Pérez, C.; Rodrlguez, M.; Martín, V. Enantiomeric synthesis of polysubstituted furanes by stereoselective intramolecular bromoetherification. *Tetrahedron Lett.* **1988**, *29*, 3149-3152.

S6. Jecs, E.; Diver, S. T. Toward the synthesis of amphidinolide P: Optimization of a model ene–yne metathesis fragment coupling. *Tetrahedron Lett.* **2014**, *55*, 4933-4937.

S7. Trost, B. M.; Pinkerton, A. B. A three-component coupling approach to cyclopentanoids. *J. Org. Chem.* **2001**, *66*, 7714-7722.

S8. Bothwell, I. R.; Luo, M. Large-scale, protection-free synthesis of Se-adenosyl-L-selenomethionine analogues and their application as cofactor surrogates of methyltransferases. *Org. Lett.* **2014**, *16*, 3056-3059.

S9. Coin, I.; Beyermann, M.; Bienert, M. Solid-phase peptide synthesis: From standard procedures to the synthesis of difficult sequences. *Nat. Protoc.* **2007**, *2*, 3247-3256.

S10. Green, M. R.; Sambrook, J., Molecular cloning. *A Laboratory Manual 4th* **2012**.

S11. Makde, R. D.; Tan, S. Strategies for crystallizing a chromatin protein in complex with the nucleosome core particle. *Anal. Biochem.* **2013**, *442*, 138-145.

S12. Ran, F. A.; Hsu, P. D.; Wright, J.; Agarwala, V.; Scott, D. A.; Zhang, F. Genome engineering using the CRISPR-Cas9 system. *Nat. Protoc.* **2013**, *8*, 2281-2308.

S13. Koller-Eichhorn, R.; Marquardt, T.; Gail, R.; Wittinghofer, A.; Kostrewa, D.; Kutay, U.; Kambach, C. Human OLA1 defines an ATPase subfamily in the Obg family of GTP-binding proteins. *J.Biol. Chem.* **2007,** *282*, 19928-19937.

S14. Smith, P. K.; Krohn, R. I.; Hermanson, G.; Mallia, A.; Gartner, F.; Provenzano, M.; Fujimoto, E.; Goeke, N.; Olson, B.; Klenk, D. Measurement of protein using bicinchoninic acid. *Anal. Biochem.* **1985**, *150*, 76-85.

S15. Shevchenko, A.; Tomas, H.; Havli, J.; Olsen, J. V.; Mann, M. In-gel digestion for mass spectrometric characterization of proteins and proteomes. *Nat. Protoc.* **2006**, *1*, 2856-2860.

S16. Islam, K.; Chen, Y. L.; Wu, H.; Bothwell, I. R.; Blum, G. J.; Zeng, H.; Dong, A. P.; Zheng, W. H.; Min, J. R.; Deng, H. T.; Luo, M. K. Defining efficient enzyme-cofactor pairs for bioorthogonal profiling of protein methylation. *P. Natl. Acad. Sci. USA* **2013,** *110*, 16778-16783.

S17. Sorenson, A. E.; Askin, S. P.; Schaeffer, P. M. In-gel detection of biotin–protein conjugates with a green fluorescent streptavidin probe. *Anal. Methods* **2015**, *7*, 2087-2092.

S18. Tooley, C. E. S.; Petkowski, J. J.; Muratore-Schroeder, T. L.; Balsbaugh, J. L.; Shabanowitz, J.; Sabat, M.; Minor, W.; Hunt, D. F.; Macara, I. G. NRMT is an α-N-methyltransferase that methylates RCC1 and retinoblastoma protein. *Nature* **2010**, *466*, 1125-1128.

S19. Petkowski, J. J.; Schaner Tooley, C. E.; Anderson, L. C.; Shumilin, I. A.; Balsbaugh, J. L.; Shabanowitz, J.; Hunt, D. F.; Minor, W.; Macara, I. G. Substrate specificity of mammalian N-terminal α-amino methyltransferase NRMT. *Biochemistry* **2012**, *51*, 5942-5950.

S20. Dong, C.; Mao, Y.; Tempel, W.; Qin, S.; Li, L.; Loppnau, P.; Huang, R.; Min, J. Structural basis for substrate recognition by the human N-terminal methyltransferase 1. *Gene. Dev.* **2015**, *29*, 2343-2348.

S21. Wu, R.; Yue, Y.; Zheng, X.; Li, H. Molecular basis for histone N-terminal methylation by NRMT1. *Gene. Dev.* **2015,** *29*, 2337-2342.

S22. Speicher, K.; Kolbas, O.; Harper, S.; Speicher, D. Systematic analysis of peptide recoveries from in-gel digestions for protein identifications in proteome studies. *J. Biomol. Tech.* **2000,** *11*, 74-86.

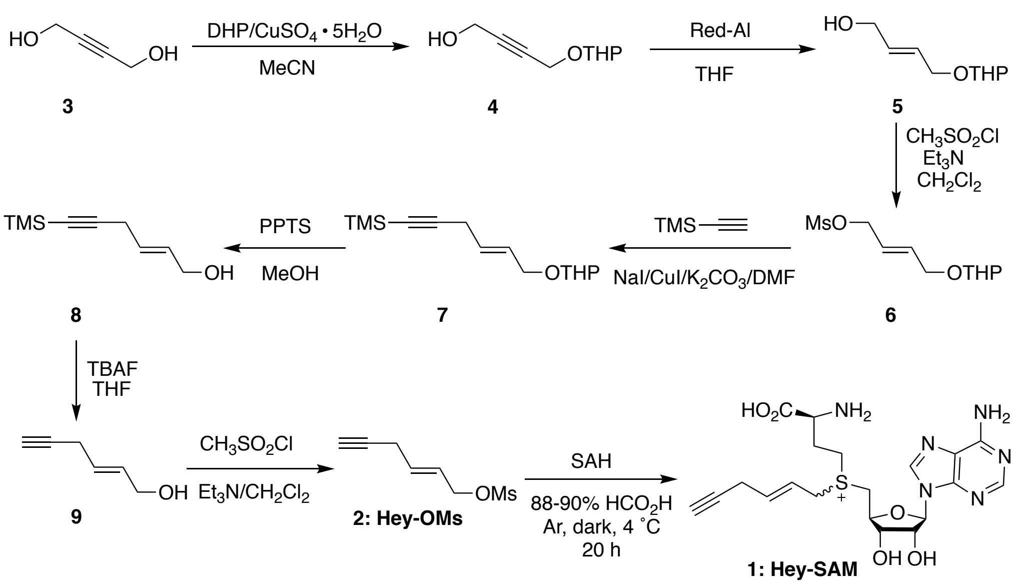

Scheme S1. Chemical synthesis of Hey-SAM **1**

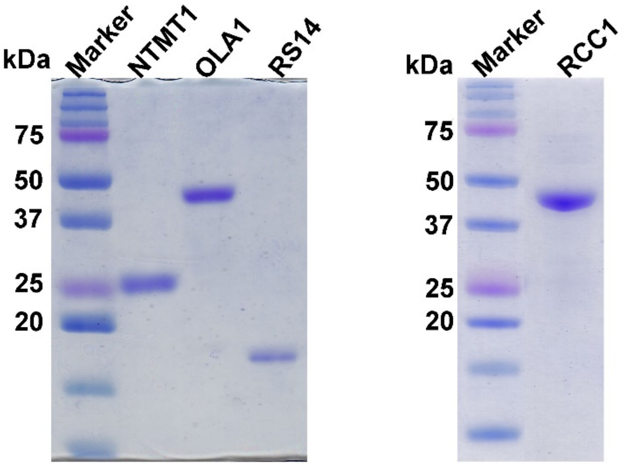

Figure S1. Tris-tricine SDS-PAGE of recombinant proteins from *E. coli*. The calculated MWs for NTMT1, OLA1, RS14, and RCC1 are 27.7, 45.7, 17.7, and 45.9 KDa, respectively.

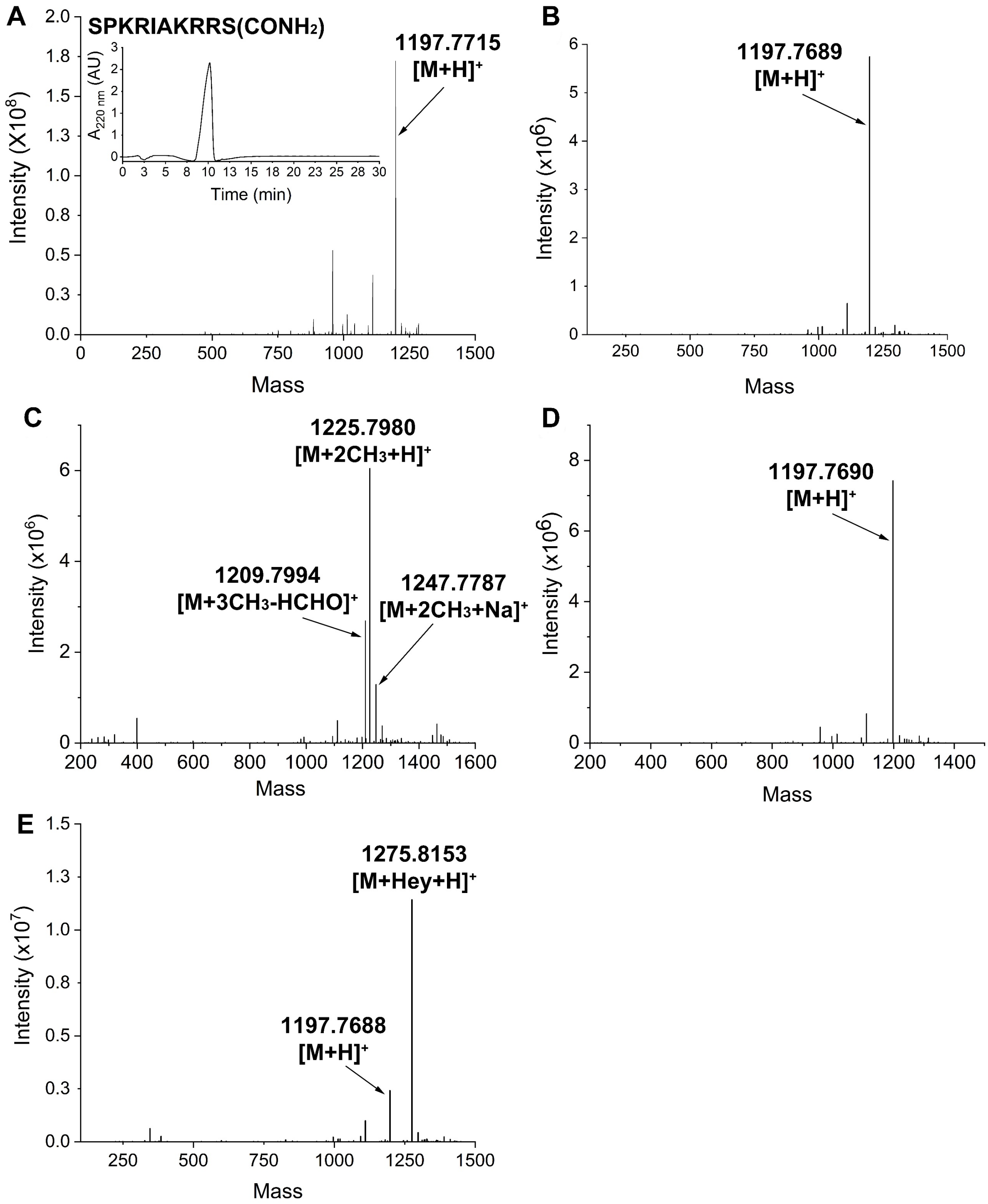

Figure S2. MS analysis of $RCC1_p$ modification by *wt* NTMT1 using SAM or Hey-SAM as the cofactor. All reactions were carried out with 20 μM $RCC1_p$ and 200 μM cofactor for 2 h at 37 °C. (A) MS and HPLC (inset) analyses of the synthesized $RCC1_p$. The calculated exact mass for $[M+H]^+$ is 1197.7656 Da. (B) MS analysis of the reaction between $RCC1_p$ and SAM in the absence of the enzyme. (C) MS analysis of the reaction between $RCC1_p$ and SAM catalyzed by *wt* NTMT1. The calculated exact masses for $[M+2CH_3+H]^+$, $[M+2CH_3+Na]^+$, and $[M+3CH_3–HCHO]^+$ are 1225.7969, 1247.7789, and 1209.8020 Da. (D) MS analysis of the reaction between $RCC1_p$ and Hey-SAM in the absence of the enzyme. (E) MS analysis of the reaction between $RCC1_p$ and Hey-SAM catalyzed by *wt* NTMT1. The calculated exact mass for monoalkylated ion, $[M+Hey+H]^+$, is 1275.8126 Da.

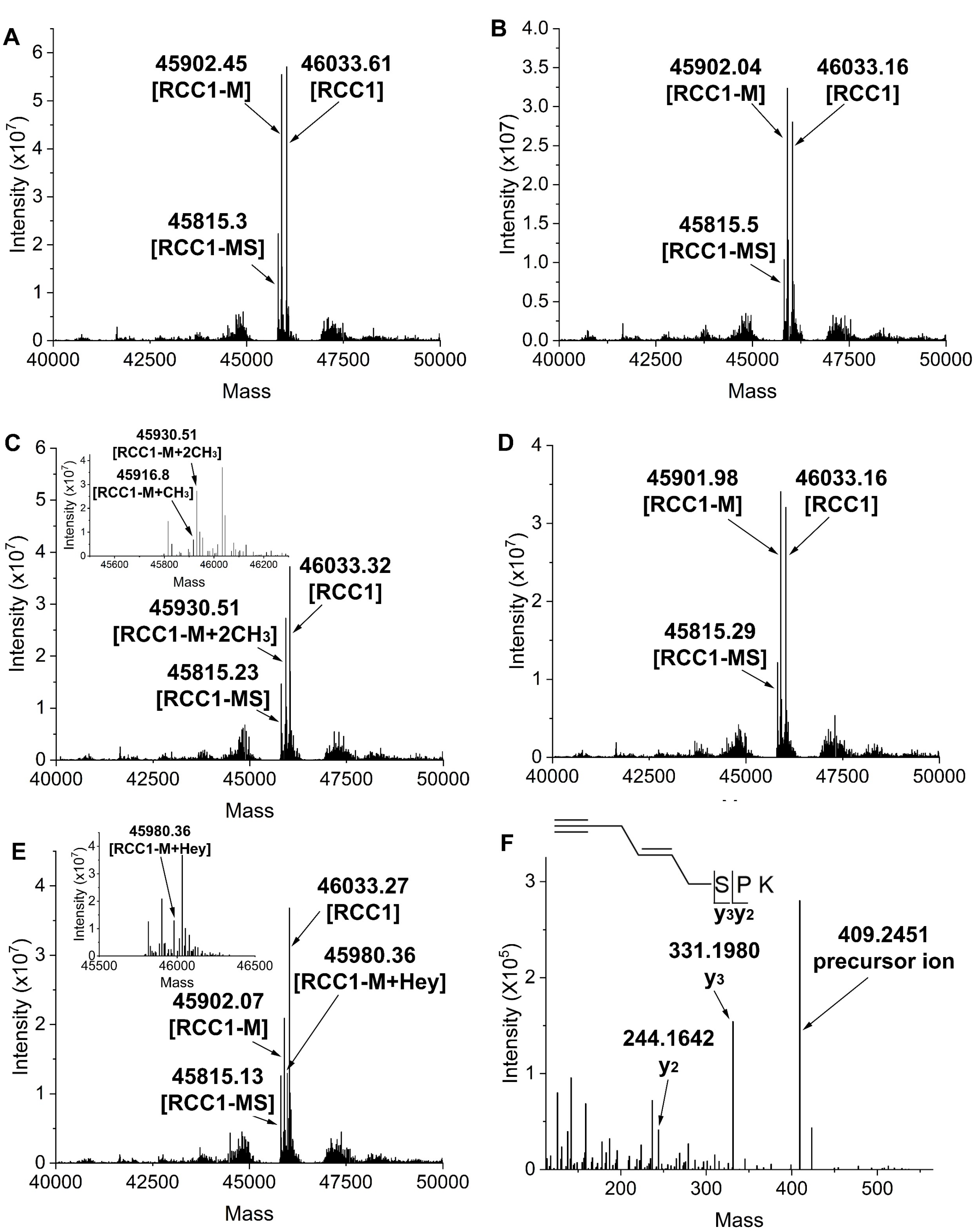

Figure S3. Intact protein MS analysis of RCC1 modification by *wt* NTMT1 using SAM or Hey-SAM as the cofactor. All reactions were carried out with 20 μM RCC1 protein and 200 μM cofactor for 2 h at 37 °C. Calculated MWs of full-length, M removed, and MS removed RCC1 are 46034.14, 45902.95, and 45815.87 Da, respectively. Only the RCC1 with the M removed is the active substrate of *wt* NTMT1. The insets in (C) and (E) are expansions of MWs. (A) RCC1 purified from *E. coli* C41(DE3) strain. (B) RCC1 and SAM in the absence of *wt* NTMT1. (C) RCC1 and SAM in the presence of 3 μM *wt* NTMT1. Calculated MWs of mono- ([RCC1–M+CH_3]) and dimethylated RCC1 ([RCC1–M+2CH_3]) are 45916.98 and 45931.00 Da, respectively. (D) RCC1 and Hey-SAM in the absence of *wt* NTMT1. (E) RCC1 and Hey-SAM in the presence of 3 μM *wt* NTMT1. The calculated MW of monoalkylated RCC1 ([RCC1–M+Hey]) is 45981.06 Da. (F) Tandem MS spectrum of mapping the modification site on RCC1.

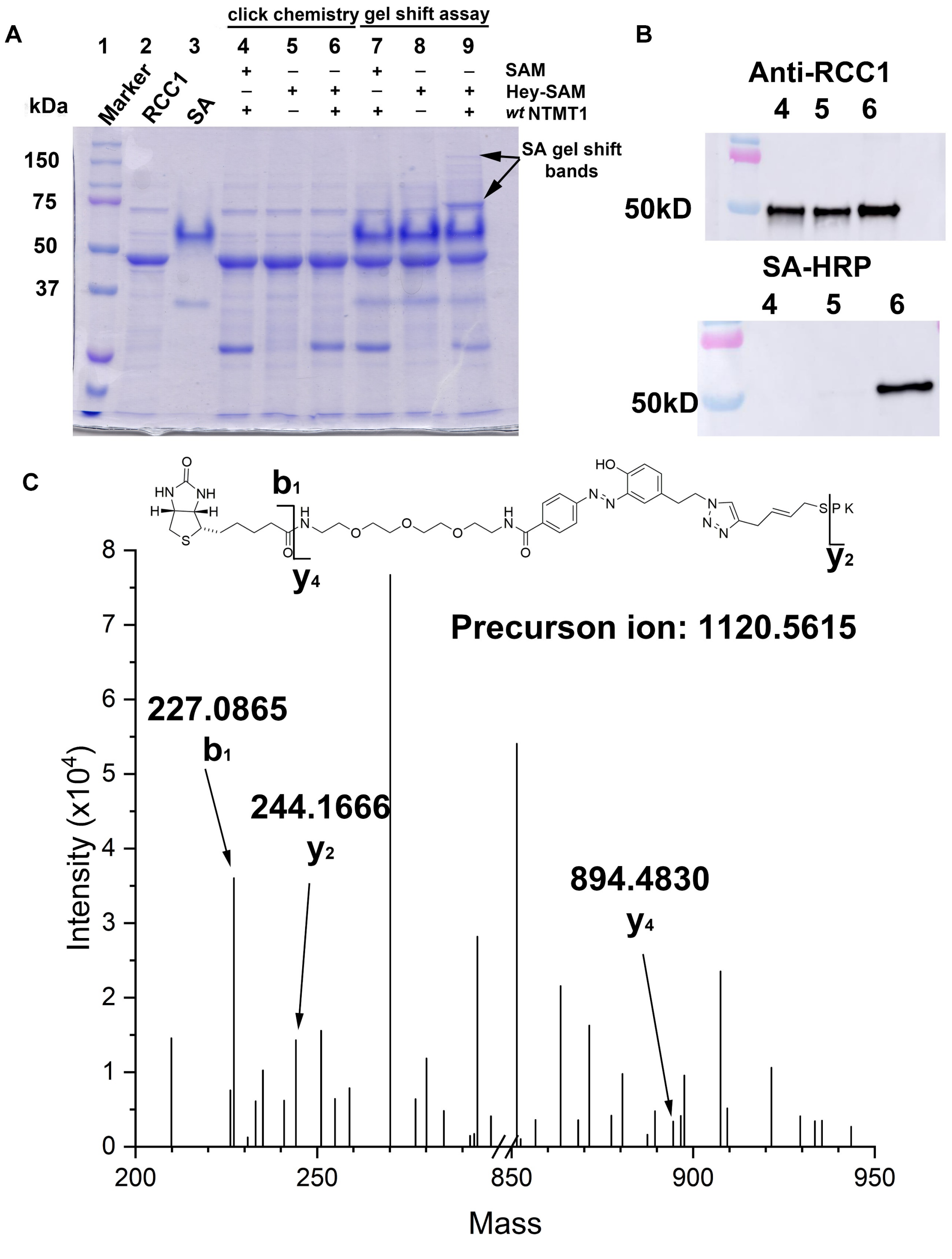

Figure S4. Click chemistry between DBA and purified RCC1 pre-labeled with different cofactors. (A) SDS-PAGE gel. Lanes 1, 2, and 3 are MW markers, purified RCC1, and purchased SA, respectively. Non-denatured SA exists as a mixture of dimer (26.4 KDa) and tetramer (52.8 KDa). Lanes 4, 5, and 6 are click chemistry of purified RCC1 pre-labeled with SAM (control), no NTMT1 (control), and Hey-SAM (Lane 6) using DBA, respectively. Lanes 7-9 are gel shift assays of click reactions shown in Lanes 4-6 with non-denatured SA. (B) WBs to detect RCC1 by rabbit anti-RCC1 antibody (top) and the presence of biotin using SA-HRP in Lanes 4-6 shown in A. (C) Tandem MS spectrum of mapping the biotin tag to the N-terminal serine of RCC1. The exact mass of the calculated parent tripeptide ion ($[M+H]^+$) is 1120.5608 Da.

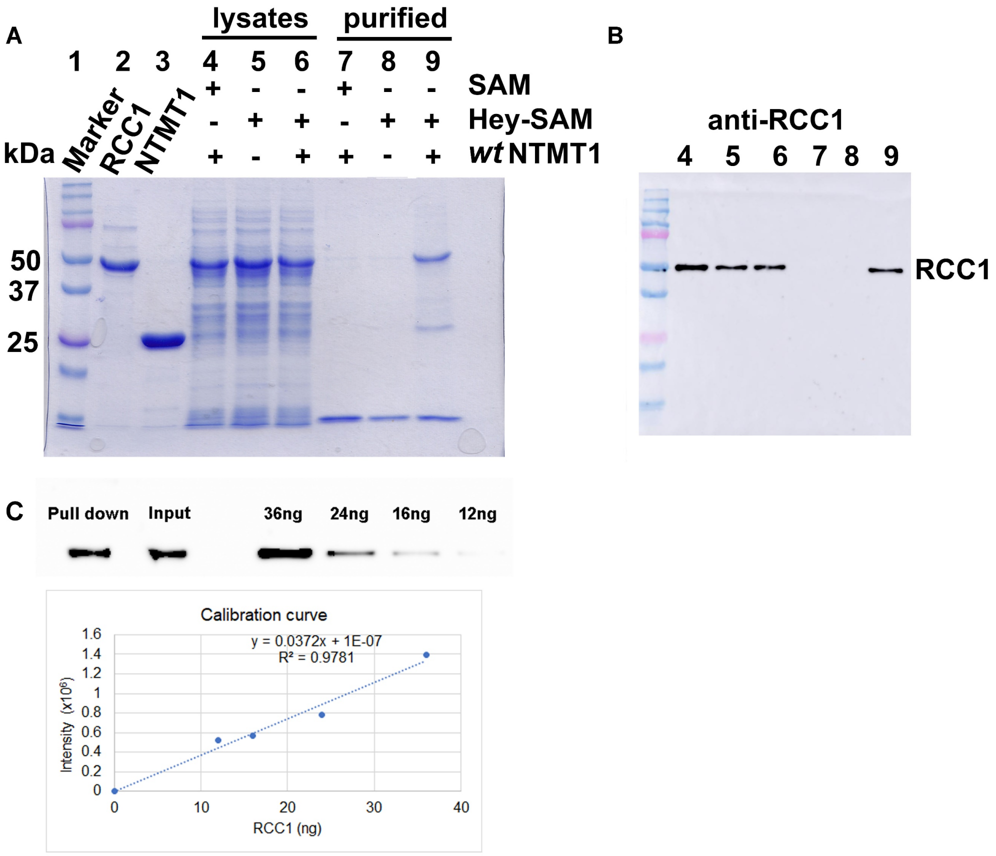

Figure S5. Proof-of-concept to pull-down overexpressed RCC1 from *E. coli* lysates. (A) SDS-PAGE gel. Lanes 1, 2, and 3 are MW markers, RCC1, and NTMT1, respectively. Lanes 4-6 are lysates labelled with SAM or Hey-SAM followed by click chemistry with DBA. Lanes 7-9 are proteins pulled-down from the treated lysates in Lanes 4-6 using SA beads. (B) Detection of RCC1 by WB using rabbit anti-RCC1 antibody. (C) WB standard curve and recovery yield calculated from WB.

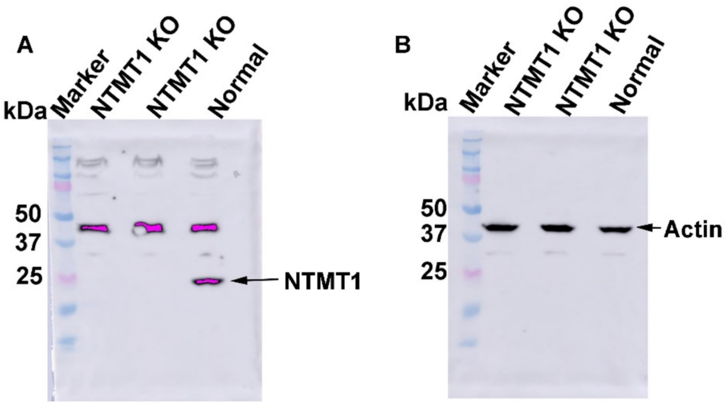

Figure S6. Generation of NTMT1 KO cells using CRISPR-Cas9. (A) WB detection using rabbit anti-NTMT1 antibody. (B) WB detection using rabbit anti-actin antibody. Actin served as a control.

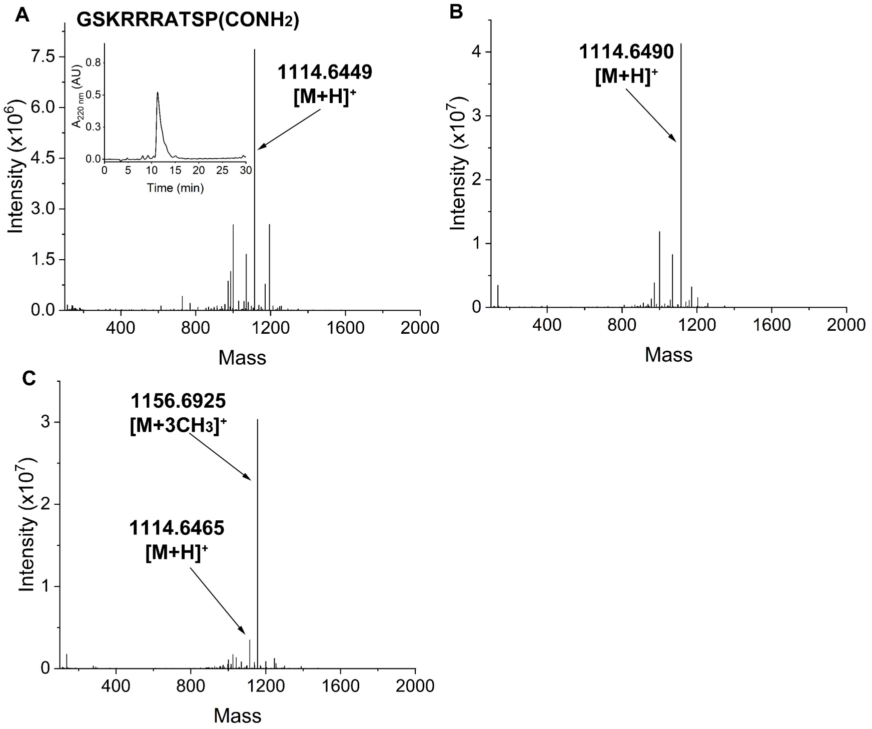

Figure S7. MS analysis of PB1 peptide methylation. (A) MS and HPLC (inset) analyses of the synthesized PB1 peptide. The calculated exact mass for $[M+H]^+$ is 1114.6557 Da. (B) MS analysis of the reaction between 20 μM PB1 peptide and 200 μM SAM in the absence of *wt* NTMT1. (C) MS analysis of the reaction between 20 μM PB1 peptide and 200 μM SAM catalyzed by 3 μM *wt* NTMT1. The calculated exact mass for trimethylated product ($[M+3CH_3]^+$) is 1156.7027 Da.

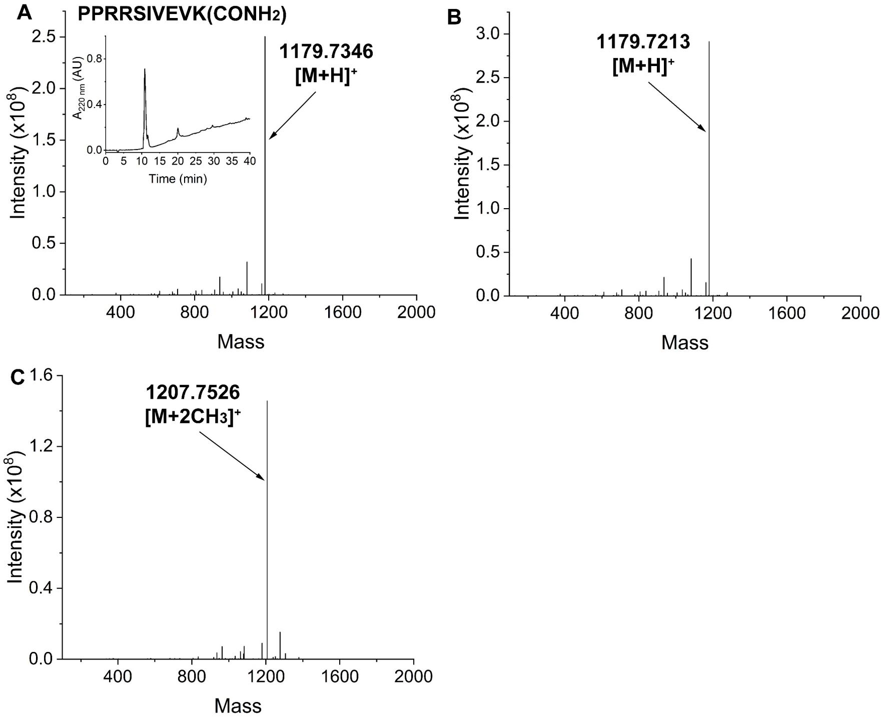

Figure S8. MS analysis of SPD2B peptide methylation. (A) MS and HPLC (inset) analyses of the synthesized SPD2B peptide. The calculated exact mass for $[M+H]^+$ is 1179.7326 Da. (B) MS analysis of the reaction between 20 μM SPD2B peptide and 200 μM SAM in the absence of *wt* NTMT1. (C) MS analysis of the reaction between 20 μM SPD2B peptide and 200 μM SAM catalyzed by 3 μM *wt* NTMT1. The calculated exact mass for dimethylated product ($[M+2CH_3]^+$) is 1207.7639 Da.

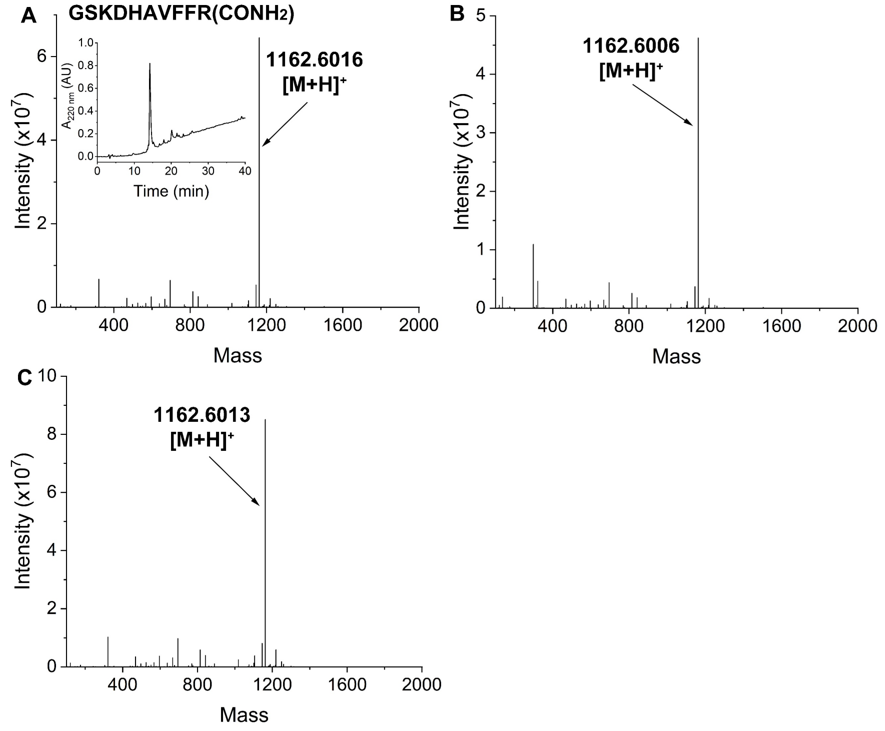

Figure S9. MS analysis of DDX60L peptide methylation. (A) MS and HPLC (inset) analyses of the synthesized DDX60L peptide. The calculated exact mass for $[M+H]^+$ is 1162.6122 Da. (B) MS analysis of the reaction between 20 μM DDX60L peptide and 200 μM SAM in the absence of *wt* NTMT1. (C) MS analysis of the reaction between 20 μM DDX60L peptide and 200 μM SAM catalyzed by 3 μM *wt* NTMT1. The calculated exact mass for trimethylated product ($[M+3CH_3]^+$) is 1204.6591 Da, which was not detected in MS.

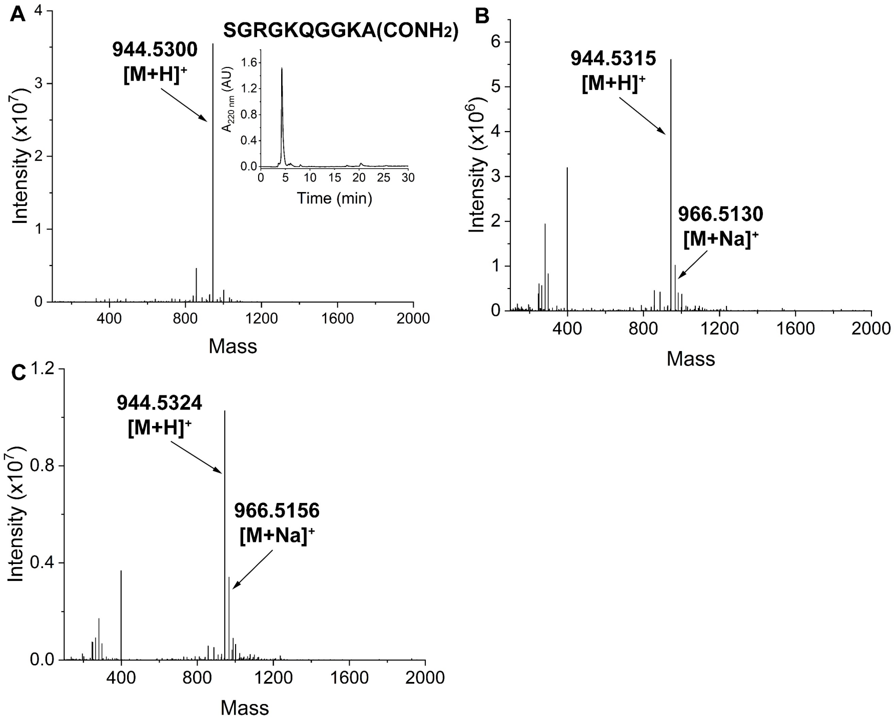

Figure S10. MS analysis of H2A1B peptide methylation. (A) MS and HPLC (inset) analyses of the synthesized H2A1B peptide. The calculated exact mass for $[M+H]^+$ is 944.5390 Da. (B) MS analysis of the reaction between 20 μM H2A1B peptide and 200 μM SAM in the absence of *wt* NTMT1. (C) MS analysis of the reaction between 20 μM H2A1B peptide and 200 μM SAM catalyzed by 3 μM *wt* NTMT1. The calculated exact mass for trimethylated product ($[M+3CH_3]^+$) is 986.5859 Da, which was not detected in MS.

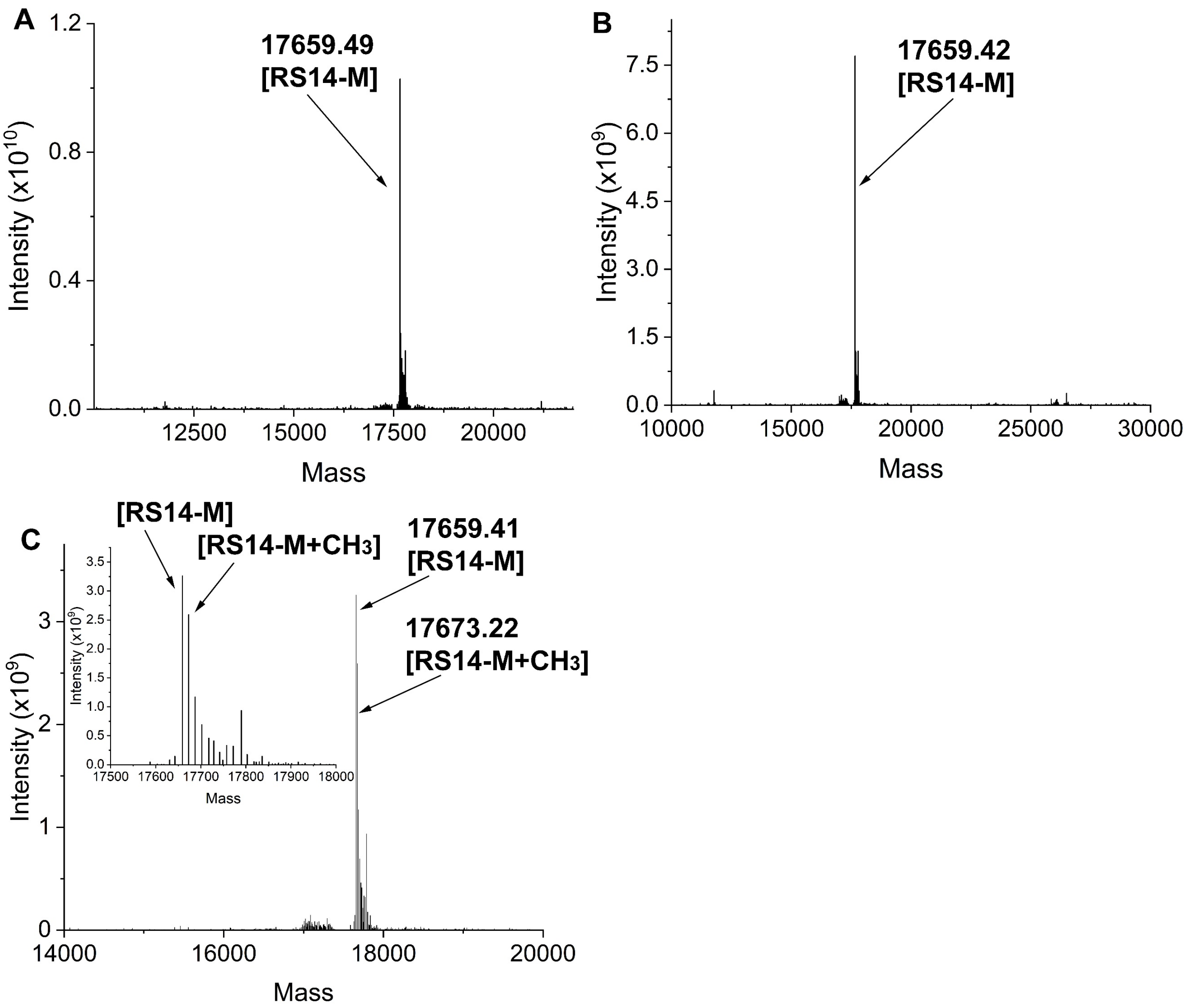

Figure S11. Intact protein MS analysis of RS14 methylation. (A) RS14 purified from *E. coli* BL21(DE3) strain. The calculated MW of RS14 with the initial methionine removal is 17661.2 Da. (B) 20 μM RS14 incubated with 200 μM SAM in the absence of *wt* NTMT1. (C) 20 μM RS14 incubated with 200 μM SAM in the presence of 3 μM *wt* NTMT1. The inset shows the expansion of MWs. The calculated MW of monomethylated product ([RS14–M+CH_3]) is 17675.2 Da.

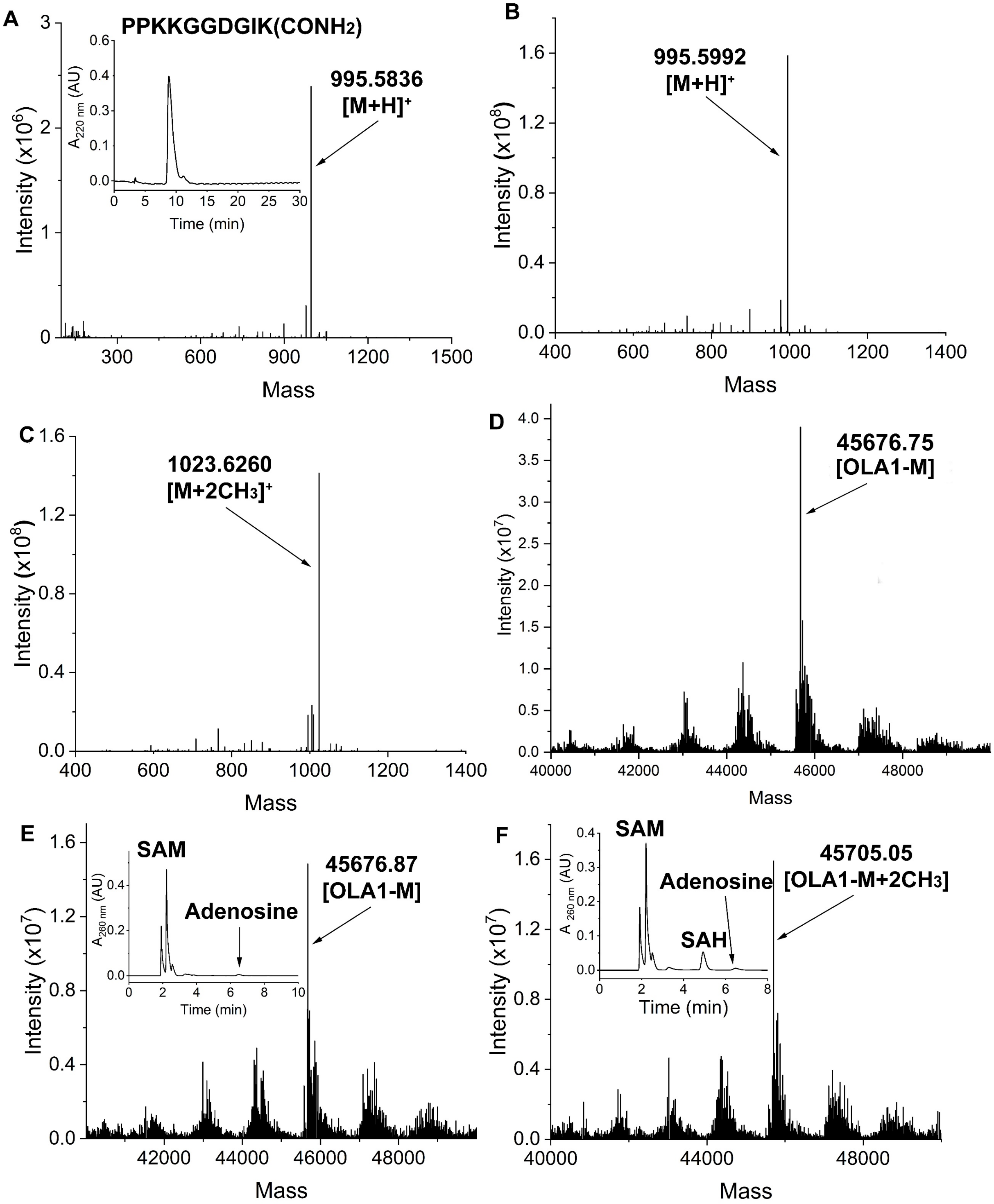

Figure S12. MS analysis of OLA1 methylation *in vitro*. (A) MS and HPLC (inset) analyses of the synthesized OLA1 peptide. The calculated exact mass for $[M+H]^+$ is 995.6002 Da. (B) MS analysis of the reaction between 20 μM OLA1 peptide and 200 μM SAM in the absence of *wt* NTMT1. (C) MS analysis of the reaction between 20 μM OLA1 peptide and 200 μM SAM catalyzed by 1 μM *wt* NTMT1. The calculated exact mass for dimethylated product ($[M+2CH_3]^+$) is 1023.6315 Da. (D) Intact protein mass of OLA1 purified from *E. coli* BL21(DE3) strain. The calculated MW of OLA1 with the initial methionine removal is 45677.5 Da. (E) Intact protein mass of OLA1 (20 μM) after incubating with 200 μM SAM in the absence of *wt* NTMT1. (F) Intact protein mass of OLA1 (20 μM) after incubating with 200 μM SAM in the presence of 1 μM NTMT1. The calculated MW of dimethylated product ($[OLA1–M+2CH_3]$) is 45706.56 Da. The insets in (E) and (F) show SAH release monitored by HPLC at 260 nm.

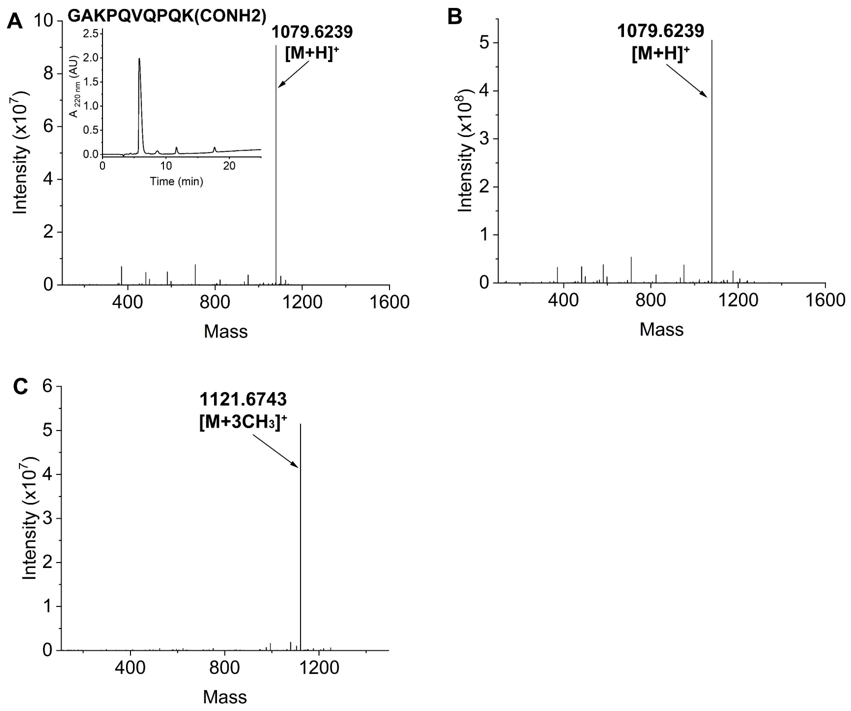

Figure S13. MS analysis of peptide methylation. (A) MS and HPLC (inset) analyses of the synthesized FECH peptide. The calculated exact mass for $[M+H]^+$ is 1079.6326 Da. (B) MS analysis of the reaction between 20 μM FECH peptide and 200 μM SAM in the absence of *wt* NTMT1. (C) MS analysis of the reaction between 20 μM FECH peptide and 200 μM SAM catalyzed by 3 μM *wt* NTMT1. The calculated exact mass for trimethylated product ($[M+3CH_3]^+$) is 1121.6795 Da.

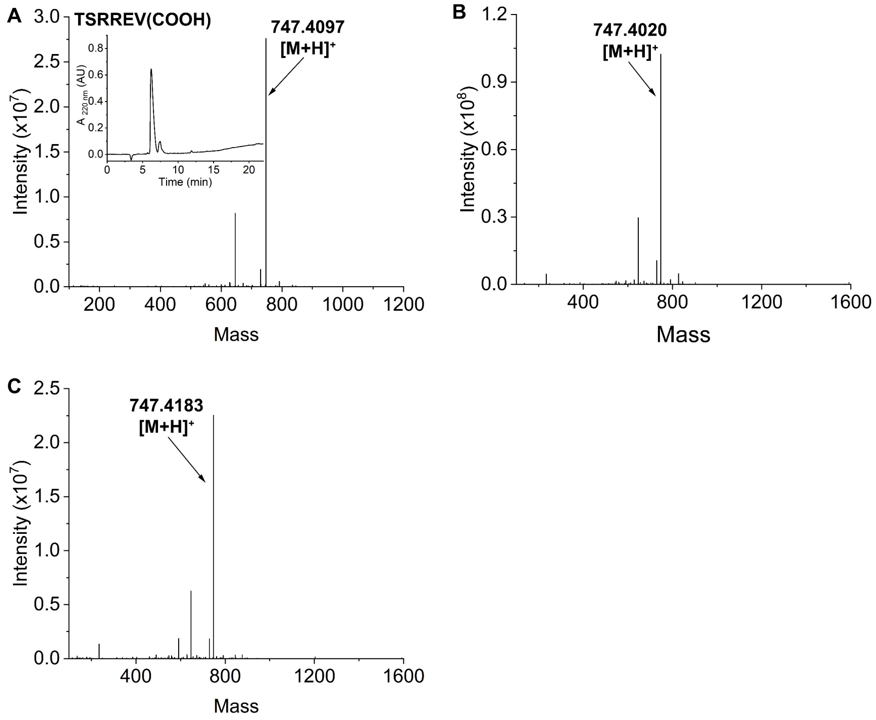

Figure S14. MS analysis of LAMC2 peptide methylation. (A) MS and HPLC (inset) analyses of the synthesized LAMC2 peptide. The calculated exact mass for $[M+H]^+$ is 747.4113 Da. (B) MS analysis of the reaction between 20 μM LAMC2 peptide and 200 μM SAM in the absence of *wt* NTMT1. (C) MS analysis of the reaction between 20 μM LAMC2 peptide and 200 μM SAM catalyzed by 3 μM *wt* NTMT1. The calculated exact mass for trimethylated product ($[M+3CH_3]^+$) is 789.4583 Da, which was not detected in MS.

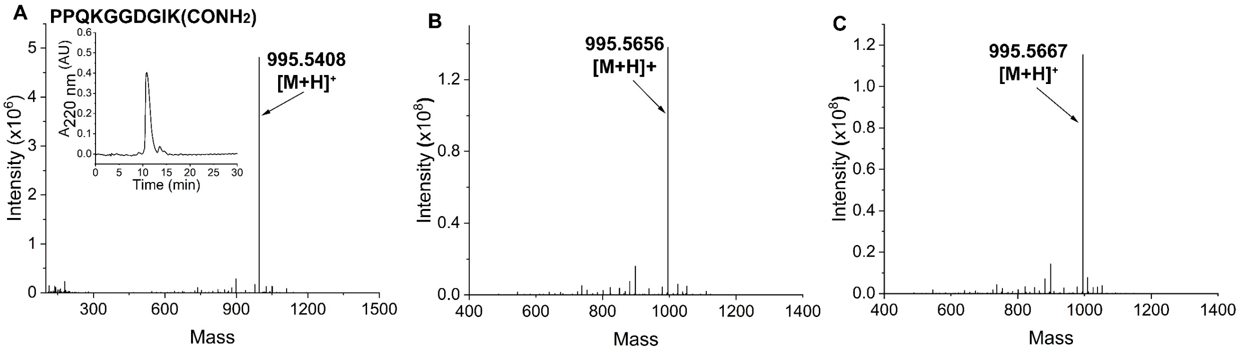

Figure S15. MS analysis of K4Q-OLA1 peptide methylation. (A) MS and HPLC (inset) analyses of the synthesized K4Q-OLA1 peptide. The calculated exact mass for $[M+H]^+$ is 995.5638 Da. (B) MS analysis of the reaction between 20 μM K4Q-OLA1 peptide and 200 μM SAM in the absence of *wt* NTMT1. (C) MS analysis of the reaction between 20 μM K4Q-OLA1 peptide and 200 μM SAM catalyzed by 1 μM *wt* NTMT1. The calculated exact mass for dimethylated product ($[M+2CH_3]^+$) is 1023.5951 Da, which was not detected in MS.

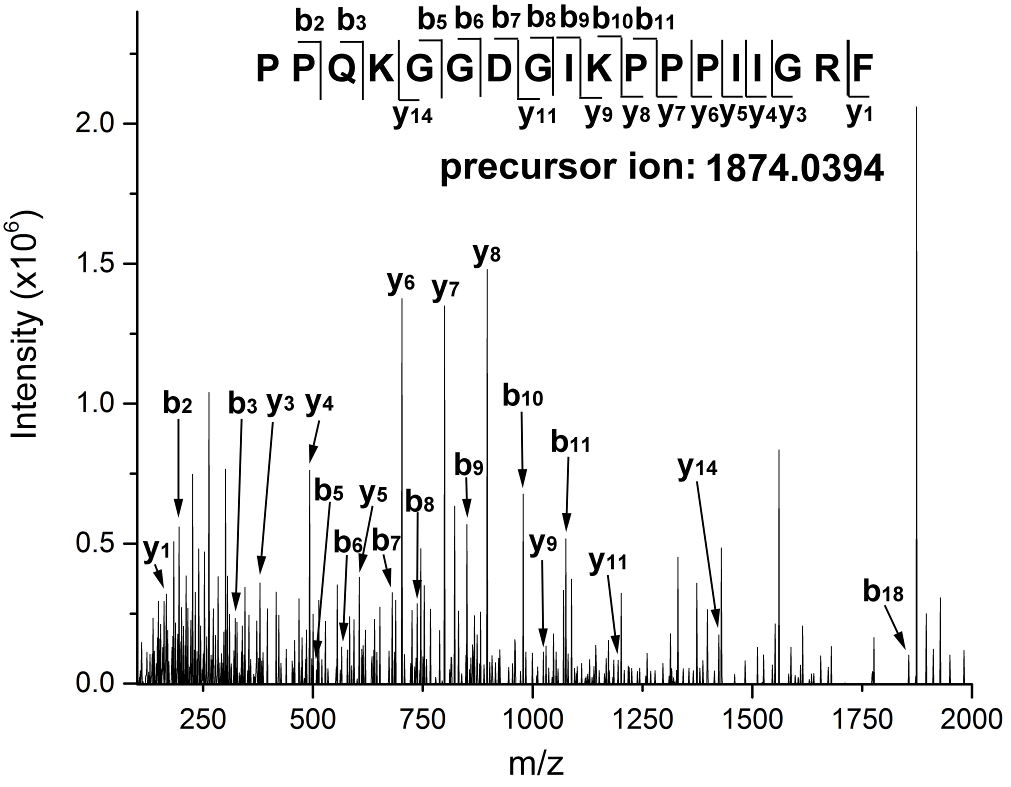

Figure S16. Tandem MS spectrum of nonmethylated peptide fragment obtained from normal HEK293FT cells. This 18-mer peptide fragment corresponds to the first 18 N-terminal residues of K4Q-OLA1 after methionine removal.

Table S1. Expanded N-terminal motifs for NTMT1 substrates

Residues	1	M								
	2	A	G	F	M	P	S	T	R	Y
	3	A	G	M	N	P	S	T		
	4	K	R							

Table S2. Potential protein targets of NTMT1*

UniProt ID	Protein name	Confidence score	Anova (*p*)	Max fold change
P08670	VIME	449.57	7.82E-06	2.88
P53396	ACLY	439.42	9.73E-05	2.95
Q9NR22	ANM8	289.60	1.34E-05	6.37
P54652	HSP72	246.86	0.000118	2.97
P22102	PUR2	173.66	0.000116	2.52
P13667	PDIA4	137.95	5.01E-05	2.40
P04181	OAT	115.58	8.47E-05	2.88
Q5H9U9	DDX60L	92.45	5.94E-05	4.12
Q05BV3	EMAL5	90.08	7.60E-05	3.16
P37802	TAGL2	75.66	0.000104	3.12
P62750	RL23A	70.68	4.02E-05	4.77
Q6GYQ0	RGPA1	68.95	5.95E-05	2.36
Q8N7X0	ADGB	64.13	0.002031	2.91
P15121	ALDR	59.92	2.11E-06	3.15
P42336	PK3CA	58.43	0.000209	4.44
P18754	RCC1	56.32	5.01E-05	3.46
Q9Y6M1	IF2B2	50.81	6.73E-05	2.85
P46776	RL27A	45.86	3.58E-05	3.28
P61158	ARP3	43.57	0.000311	2.70
P35232	PHB	43.24	0.001095	3.05
Q659A1	ICE2	42.68	0.000314	3.08
Q6UB35	C1TM	42.05	0.00011	2.43
O00160	MYO1F	38.42	1.53E-05	7.83
B4DIP2	B4DIP2	38.40	0.000237	2.79
Q9Y4E1	WAC2C	37.10	0.000257	8.23
Q641Q2	WAC2A	37.10	0.000257	8.23
Q8IYD8	FANCM	36.06	0.000281	3.22
Q9Y5S2	MRCKB	34.31	2.07E-05	3.44
P62263	RS14	34.10	0.000113	4.20
Q16576	RBBP7	32.22	0.000883	4.23
Q8NBS9	TXND5	29.80	0.000341	2.77
Q8TF47	ZFP90	29.54	0.000505	2.82
P04908	H2A1B	29.50	0.00172	2.57
P0C0S8	H2A1	29.50	0.00172	2.57
P20671	H2A1D	29.50	0.00172	2.57
Q16777	H2A2C	29.50	0.00172	2.57
Q6FI13	H2A2A	29.50	0.00172	2.57
Q7L7L0	H2A3	29.50	0.00172	2.57
Q93077	H2A1C	29.50	0.00172	2.57
Q96KK5	H2A1H	29.50	0.00172	2.57
Q99878	H2A1J	29.50	0.00172	2.57
Q9BTM1	H2AJ	29.50	0.00172	2.57
P16104	H2AX	29.50	0.00172	2.57
Q8IUE6	H2A2B	29.50	0.00172	2.57
Q96QV6	H2A1A	29.50	0.00172	2.57

Q13017	RHG05	26.46	0.000342	2.05
Q9Y2Z9	COQ6	26.40	7.67E-05	2.74
P62805	H4	26.25	0.000414	2.72
P10606	COX5B	26.03	0.000181	4.29
Q8IWG1	WDR63	23.87	0.000666	3.67
Q8NB90	AFG2H	23.41	7.21E-05	3.09
Q13753	LAMC2	23.13	2.40E-06	4.10
P46459	NSF	21.53	0.000219	2.44
Q01955	CO4A3	20.50	7.26E-06	8.67
Q2PPJ7	RGPA2	20.03	0.000238	3.61
Q5T5U3	RHG21	19.69	1.60E-05	3.01
Q9UQB3	CTND2	17.29	0.004354	2.48
Q9NTK5	OLA1	16.12	0.000527	3.57
Q12965	MYO1E	15.73	0.000128	2.90
Q86U86	PB1	15.47	4.33E-06	7.29
P22830	FECH	15.4	6.27E-05	2.97
Q9BQ39	DDX50	12.22	5.70E-05	2.94
Q86VW1	S22AG	11.67	1.39E-05	3.90
Q6ZRP0	PR23C	11.44	5.58E-06	5.67
Q01105	SET	11.44	8.35E-05	4.14
Q96L34	MARK4	11.40	0.000136	2.85
Q9Y3F4	STRAP	10.70	0.000349	2.99
Q6NSJ2	PHLB3	9.44	0.000226	2.32
Q96GM5	SMRD1	6.01	0.001129	3.81
P42679	MATK	5.76	5.39E-05	2.67
A1X283	SPD2B	5.37	0.000273	2.94
P26358	DNMT1	4.99	3.55E-05	6.33

*Proteins highlighted in magenta are obtained from signal peptide and motif sequence analyses. Proteins highlighted in green are NTMT1 substrates previously confirmed. Proteins highlighted in yellow and magenta were selected for target validation.

Table S3. HPLC conditions for peptide purification and characterization*

		Semi-preparative HPLC	Analytical HPLC
Column		Phenomenex Luna C18(2), 5 µm, 100 Å, 10 mm × 250 mm	Waters XBridge BEH Shield RP 18, 5 µm, 130 Å, 4.6 mm × 250 mm
Flow Rate (mL/min)		3.0	1.0
Mobile Phases		**A:** 0.1% TFA in H_2O; **B:** 0.1% TFA in MeOH	
Gradient (**B**)	RCC1	0-5-30 min: 5-5-30% (23.7 min)	0-40 min: 5-95% (10.2 min)
	PB1	N/A	0-30 min: 1-15% (11.2 min)
	SPD2B	0-40 min: 10-40% (18.3 min)	0-40 min: 10-90% (10.8 min)
	DDX60L	N/A	0-40 min: 10-90% (14.2 min)
	H2A1B	N/A	0-30 min: 1-15% (4.3 min)
	FECH	N/A	0-40 min: 5-90% (5.8 min)
	LAMC2	N/A	0-40 min: 5-90% (6.2 min)
	OLA1	0–25 min: 5–40% (11.9 min)	0-30 min: 5–15% (9.0 min)
	K4Q-OLA1	0–30–50 min: 5–15–50% (37.0 min)	0-30 min: 5–15% (10.8 min)

*Numbers shown in parentheses are HPLC retention times.

Table S4. Plasmid constructions

Plasmid	Primer sequence (restriction site bolded and underlined)		Vector	Tag
*p*NTMT1	NdeI_Fw	TAGCG**CATATG**ACGAGCGAGGTGATAG	*p*ET28-MHL (Addgene)	His-tag
	HindIII_Rv	TGACG**AAGCTT**CATCTCAGGGCAAAGC		
*p*RCC1	NdeI_Fw	TCGTTC**CATATG**TCACCCAAGCGCATAGC	*p*ET30a (+) (Novagen)	
	XhoI_Rv	AAACTA**CTCGAG**GCTCTGTTCTTTGTCCTTGAC		
*p*RS14	NcoI_Fw	TTTTTA**CCATGG**CACCTCGAAAGGGGAAGG	*p*ET28b (+) (Novagen)	
	HindIII_Rv	TTTTTT**AAGCTT**CAGACGGCGACCACGGCGACC C		
*p*OLA1	NdeI_Fw	TTGATA**CATATG**CCCCCTAAAAAGGGAGGTGATG	*p*ET30a (+) (Novagen)	
	XhoI_Rv	AAACAC**CTCGAG**TTTCTTCTTCGGTTGTTGAG		
*p*K4Q-OLA1	NdeI_Fw	TTGATA**CATATG**CCCCCTCAAAAGGGAGGTGATG	*p*ET30a (+) (Novagen)	
	XhoI_Rv	AAACAC**CTCGAG**TTTCTTCTTCGGTTGTTGAG		
*p*EGFP-n1-FLAG	BamHI_Fw	AAAATT**GGATCC**ACCGGTCGCCACCATGG	*p*EGFP-n1 (Clontech)	FLAG-tag
	NotI_Rv	AA**GCGGCCGC**CTACTTATCGTCATCGTCCTTGTA ATCCTTGTACAG CTCGTCCATGCC		
*p*OLA1-EGFP-FLAG	XhoI_Fw	TTGATA**CTCGAG**GCCACCATGCCCCCTAAAAAGG GAGGTGATG	*p*EGFP-n1-FLAG (this study)	
	BamHI_Rv	AAACAC**GGATCC**ACTTTCTTCTTCGGTTGTTGAG		
*p*K4Q-OLA1-EGFP-FLAG	XhoI_Fw	ATA**CTCGAG**GCCACCATGCCCCCTCAAAAGGGAG GTGATG		
	BamHI_Rv	AAACAC**GGATCC**ACTTTCTTCTTCGGTTGTTGAG		
PX459-NTMT1$_{sgRNA1}$	BbsI_Fw	**CACC**GCTGGAAACAAATCCCACCCA	PX459 (Addgene)	N/A
	BbsI_Rv	**AAAC**TGGGTGGGATTTGTTTCCAGC		
PX459-NTMT1$_{sgRNA2}$	BbsI_Fw	**CACC**GCATGCCGTCCACCGTGGGT		
	BbsI_Rv	**AAAC**ACCCACGGTGGACGGCATGC		
Seq-NTMT1$_{exon1}$	Forward	AAAACCCATATGCTCTGCACTCAGCAGGATGTGT GAC	for sequencing to confirm NTMT1 KO	

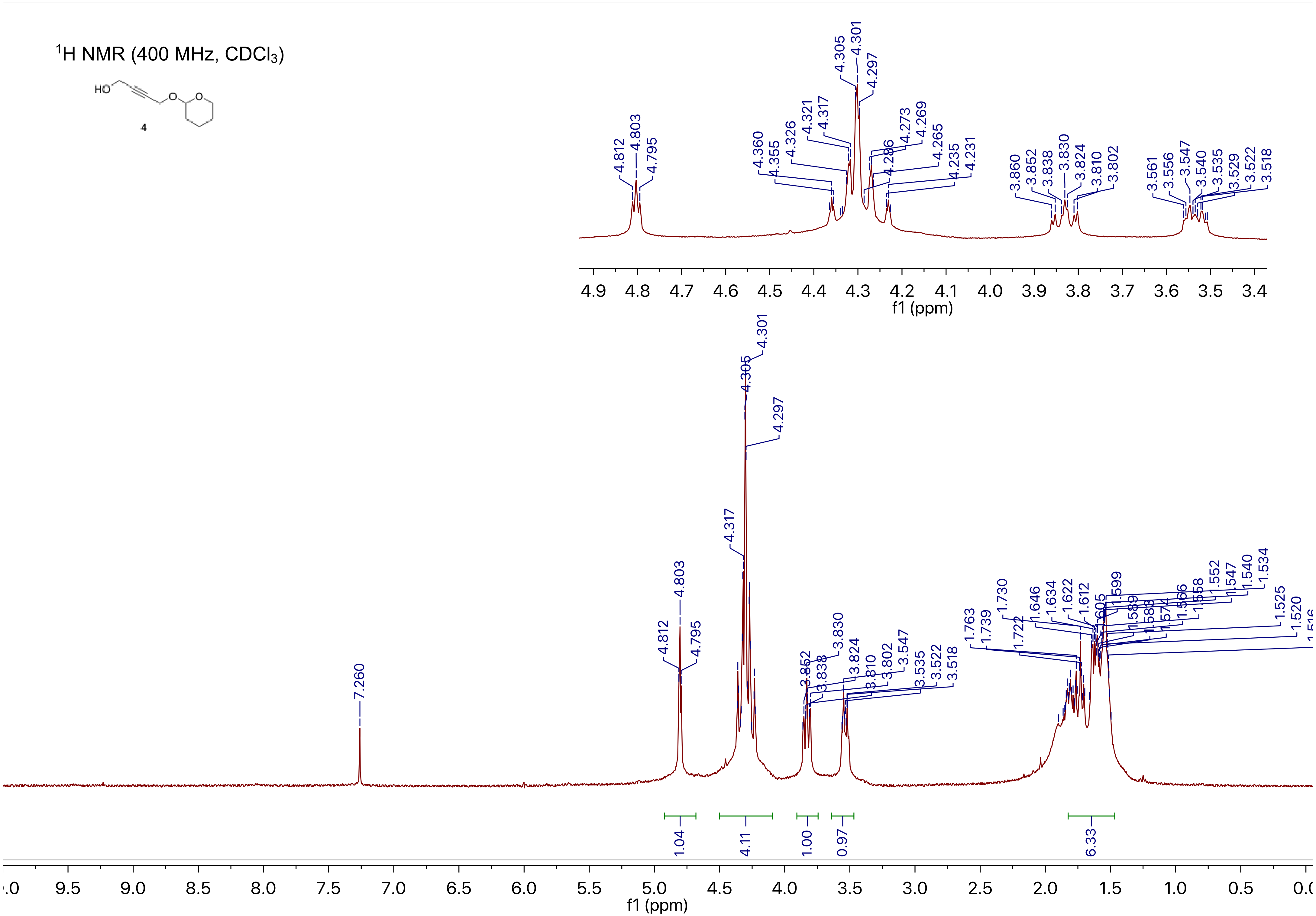

^{1}H NMR (400 MHz, CDCl$_3$)
HO
O
O
4
f1 (ppm)
7.260
4.812
4.803
4.795
4.317
4.305
4.301
4.297
3.852
3.838
3.830
3.824
3.810
3.802
3.547
3.535
3.522
3.518
1.04
4.11
1.00
0.97
6.33

^{1}H NMR, gCOSY (400, MHz, $CDCl_3$)

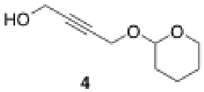

4

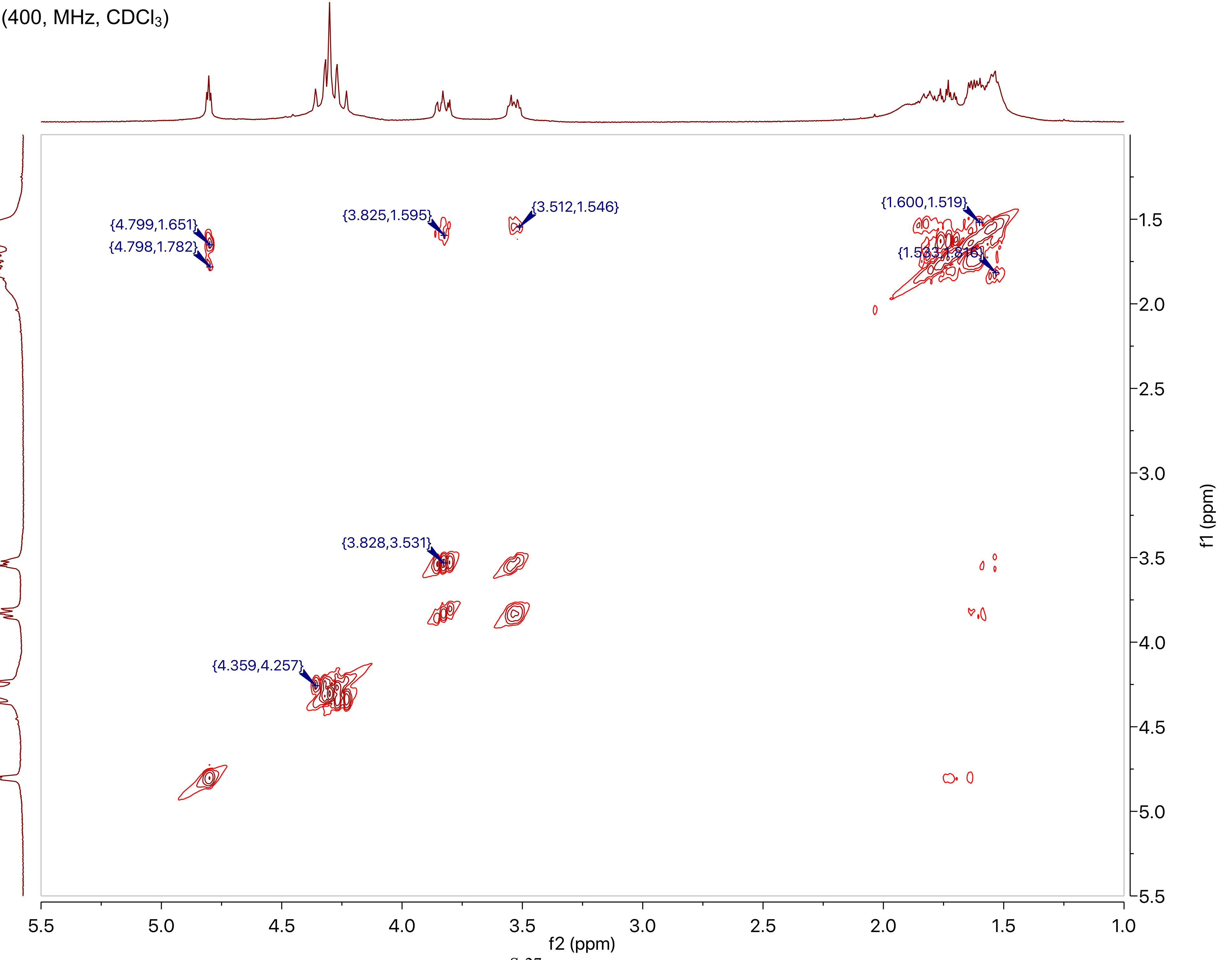

^{13}C NMR (101 MHz, $CDCl_3$)

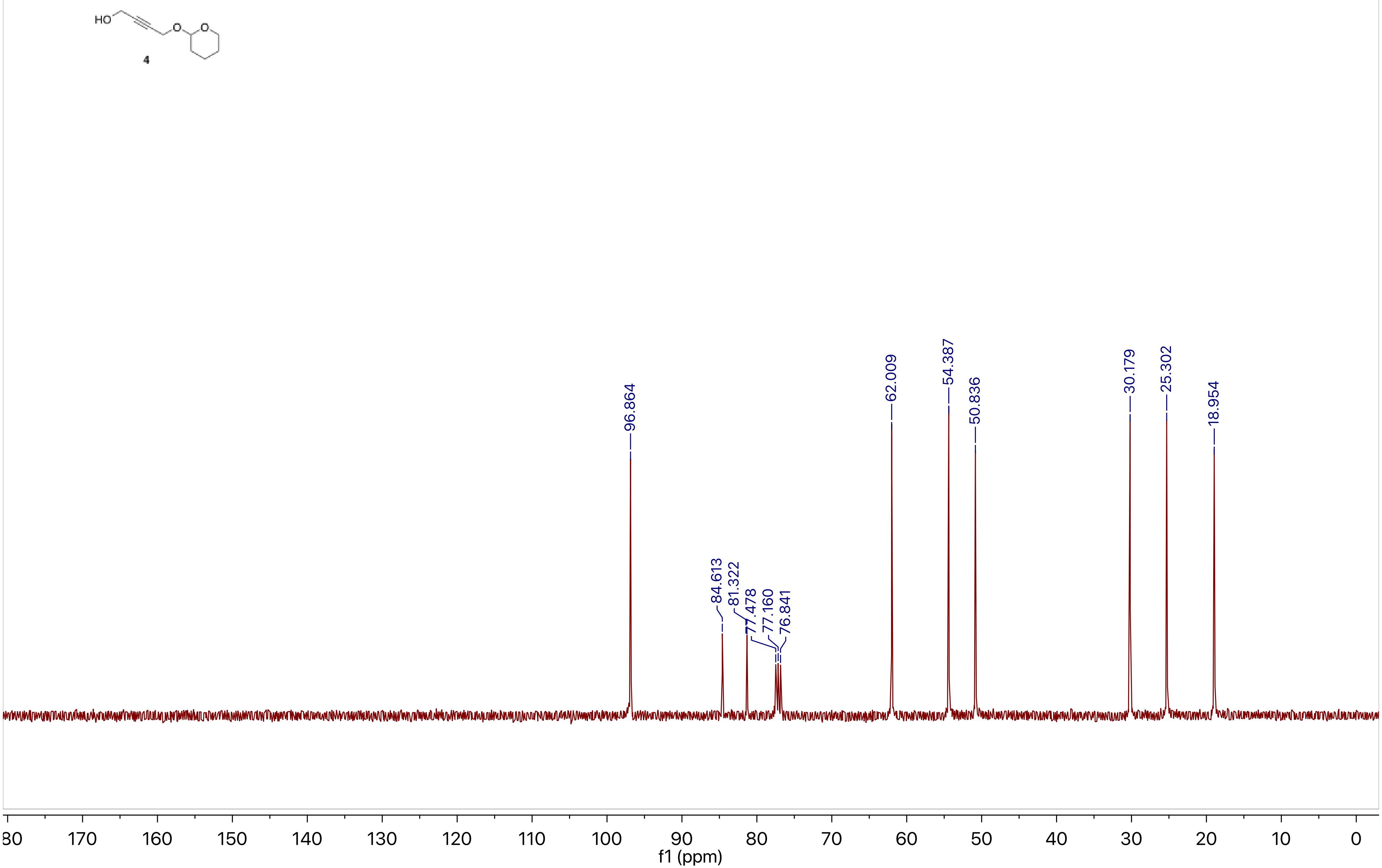

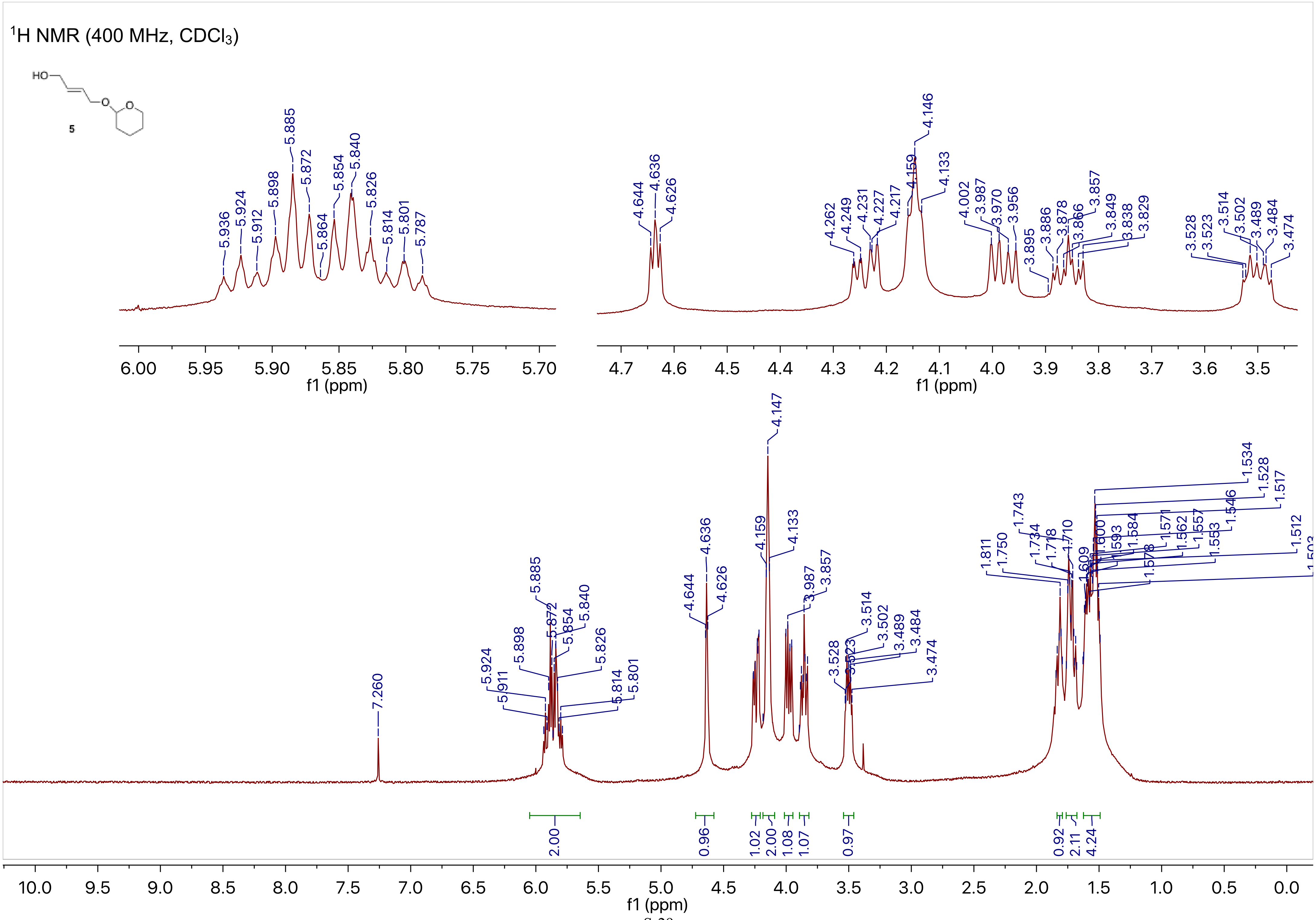
¹H NMR (400 MHz, CDCl₃)
HO
O
O
5
f1 (ppm)
f1 (ppm)
f1 (ppm)
2.00
0.96
1.02
2.00
1.08
1.07
0.97
0.92
2.11
4.24

^{1}H NMR, gCOSY (400, MHz, $CDCl_3$)

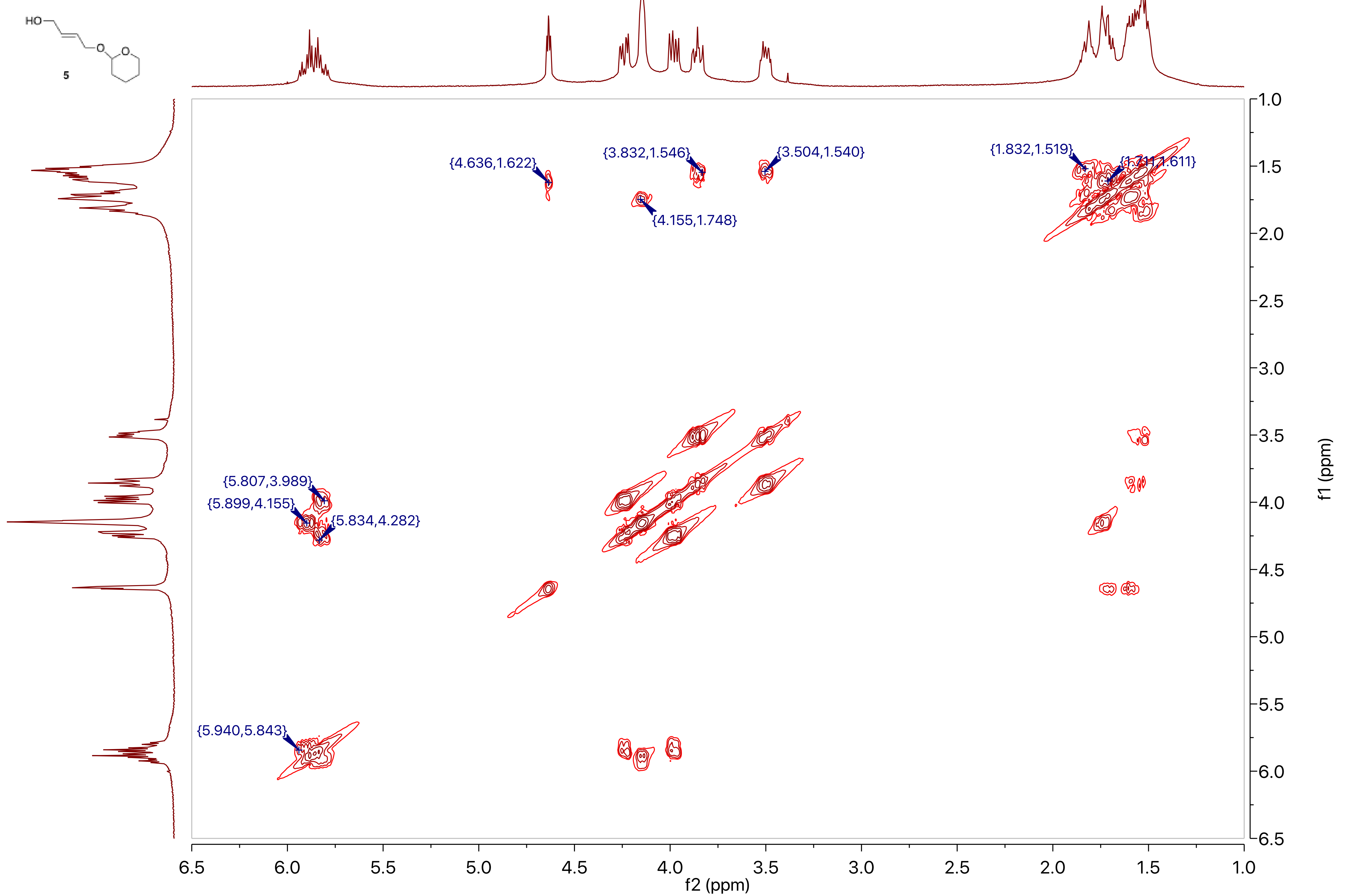

^{13}C NMR (101 MHz, $CDCl_3$)

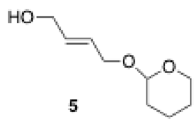

5

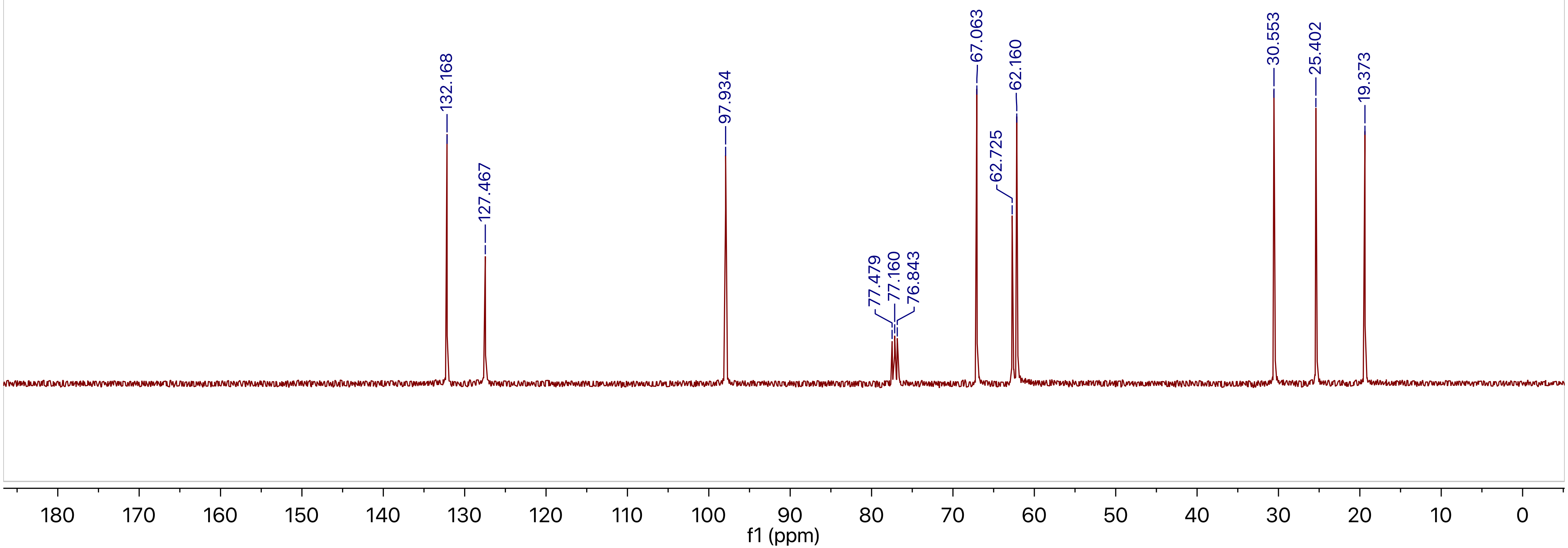

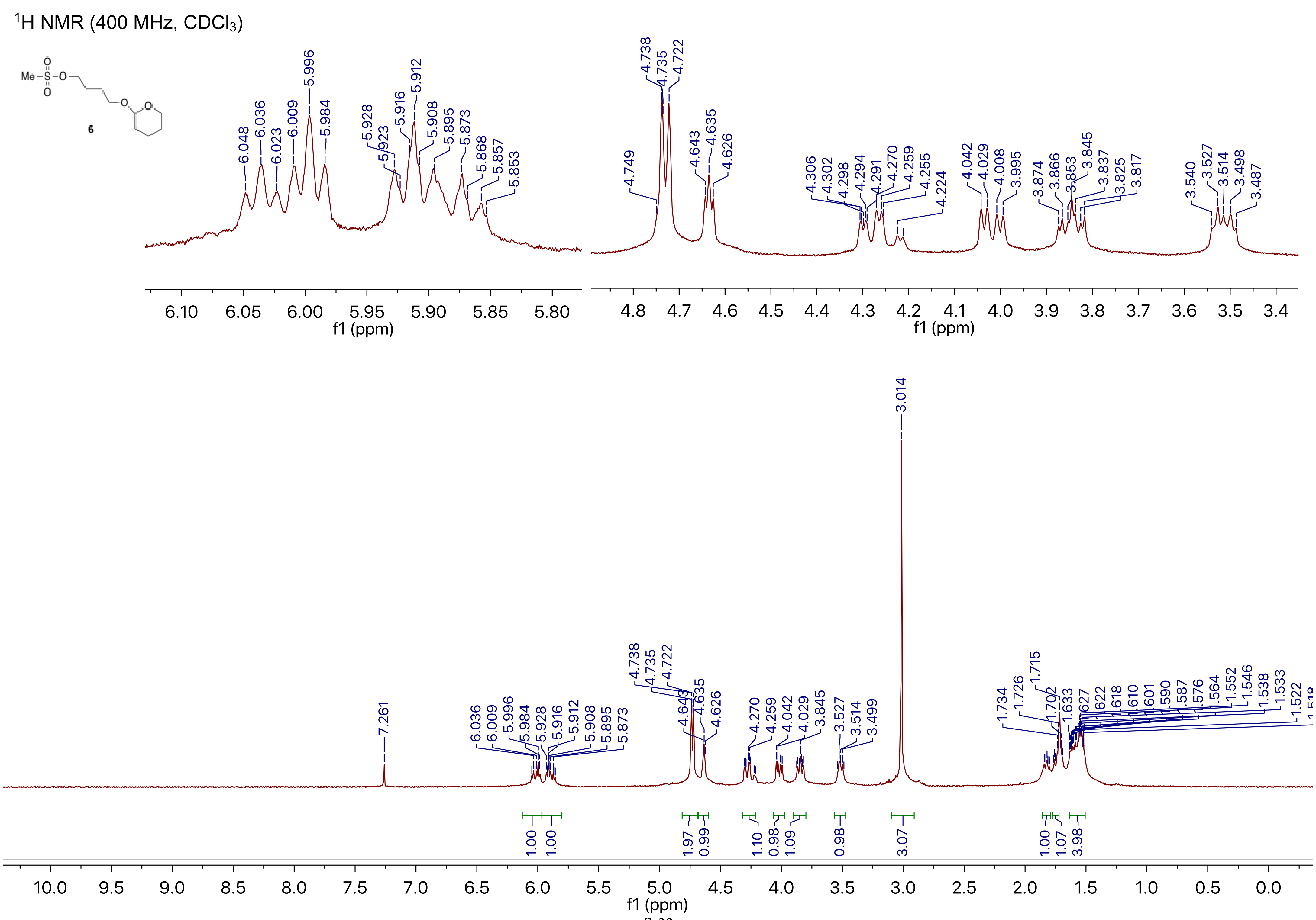
1H NMR (400 MHz, CDCl3)
6
f1 (ppm)

^{1}H NMR, gCOSY (400, MHz, $CDCl_3$)

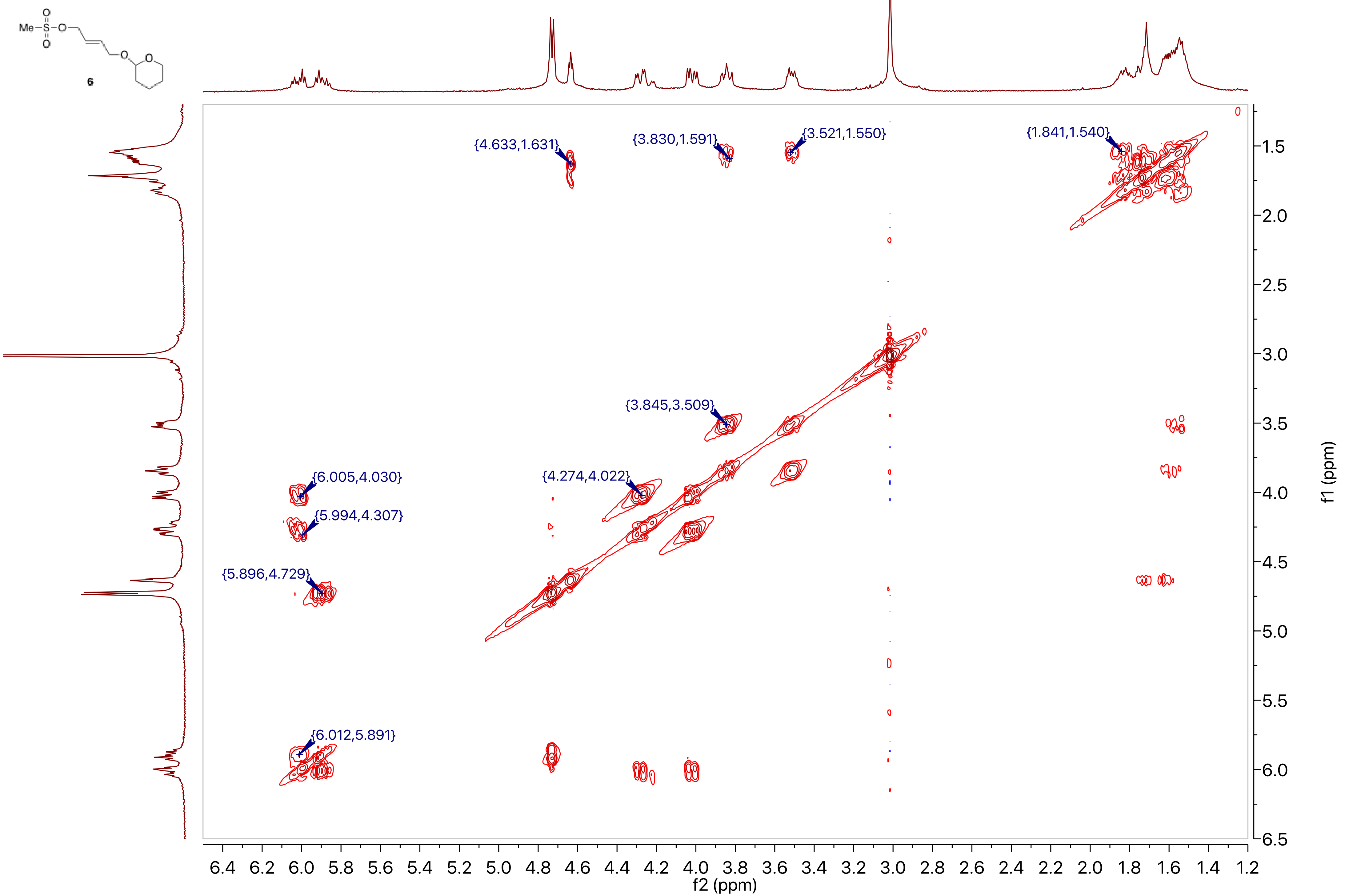

^{13}C NMR (101 MHz, $CDCl_3$)

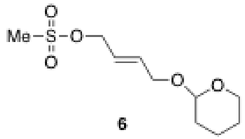

6

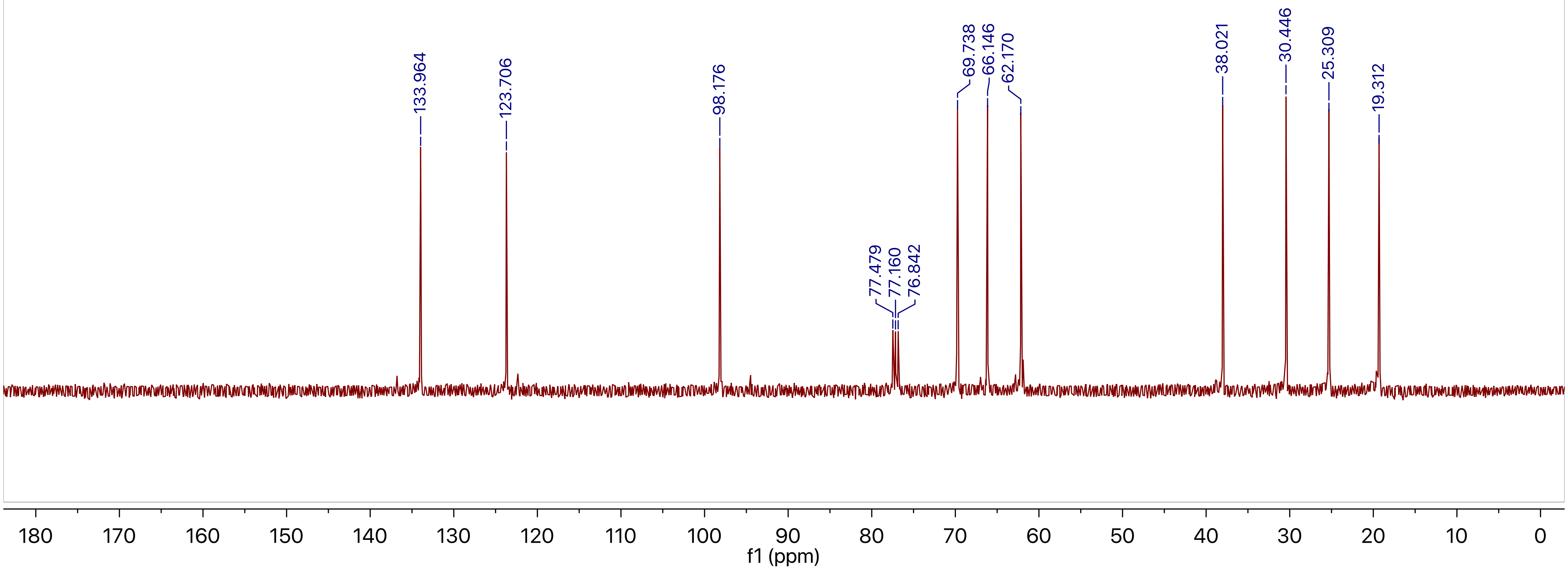

^{1}H NMR (400 MHz, $CDCl_3$)

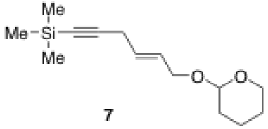

7

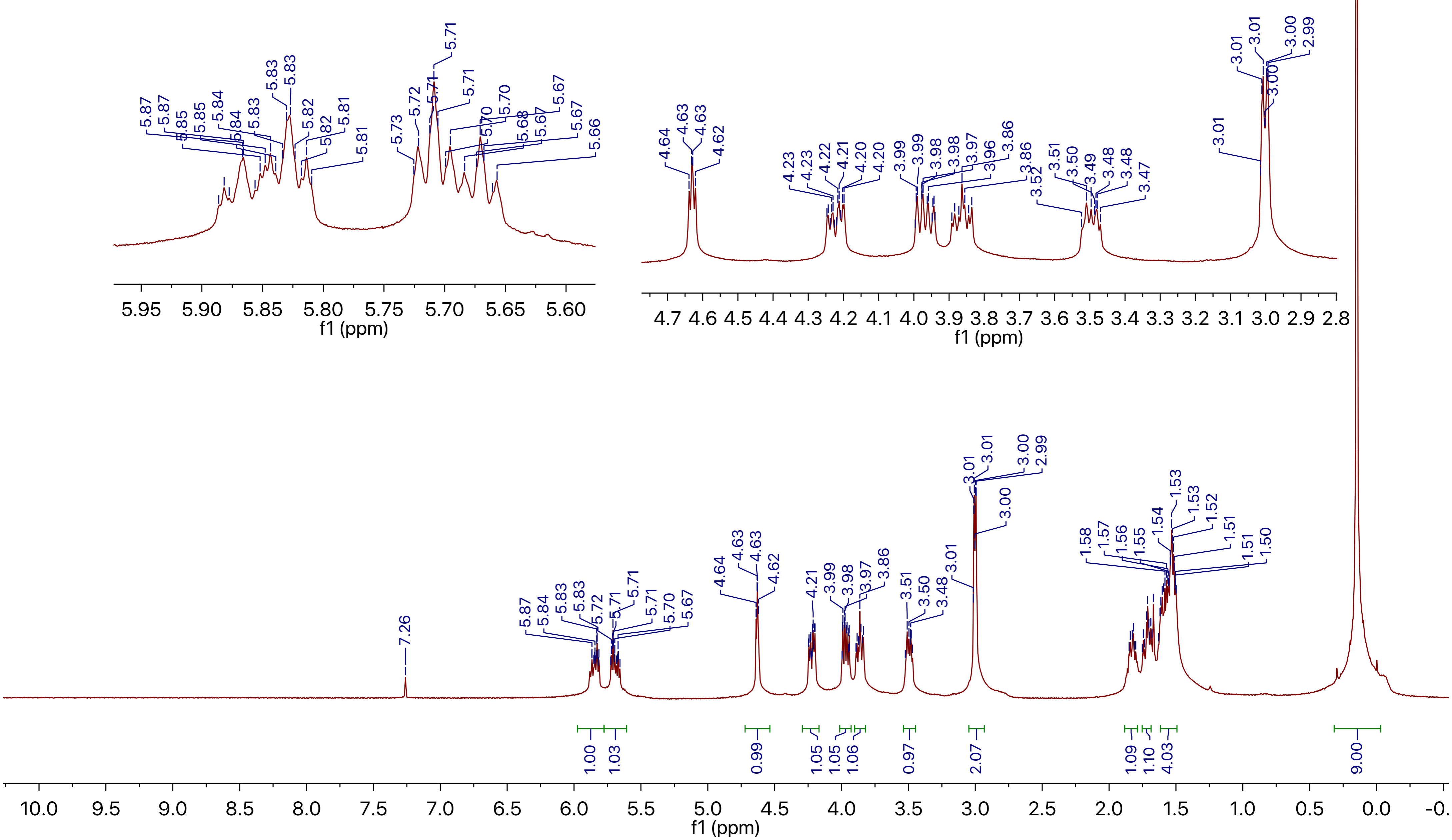

^{1}H NMR, gCOSY (400, MHz, $CDCl_3$)

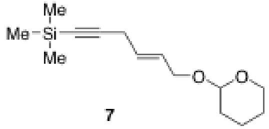

7

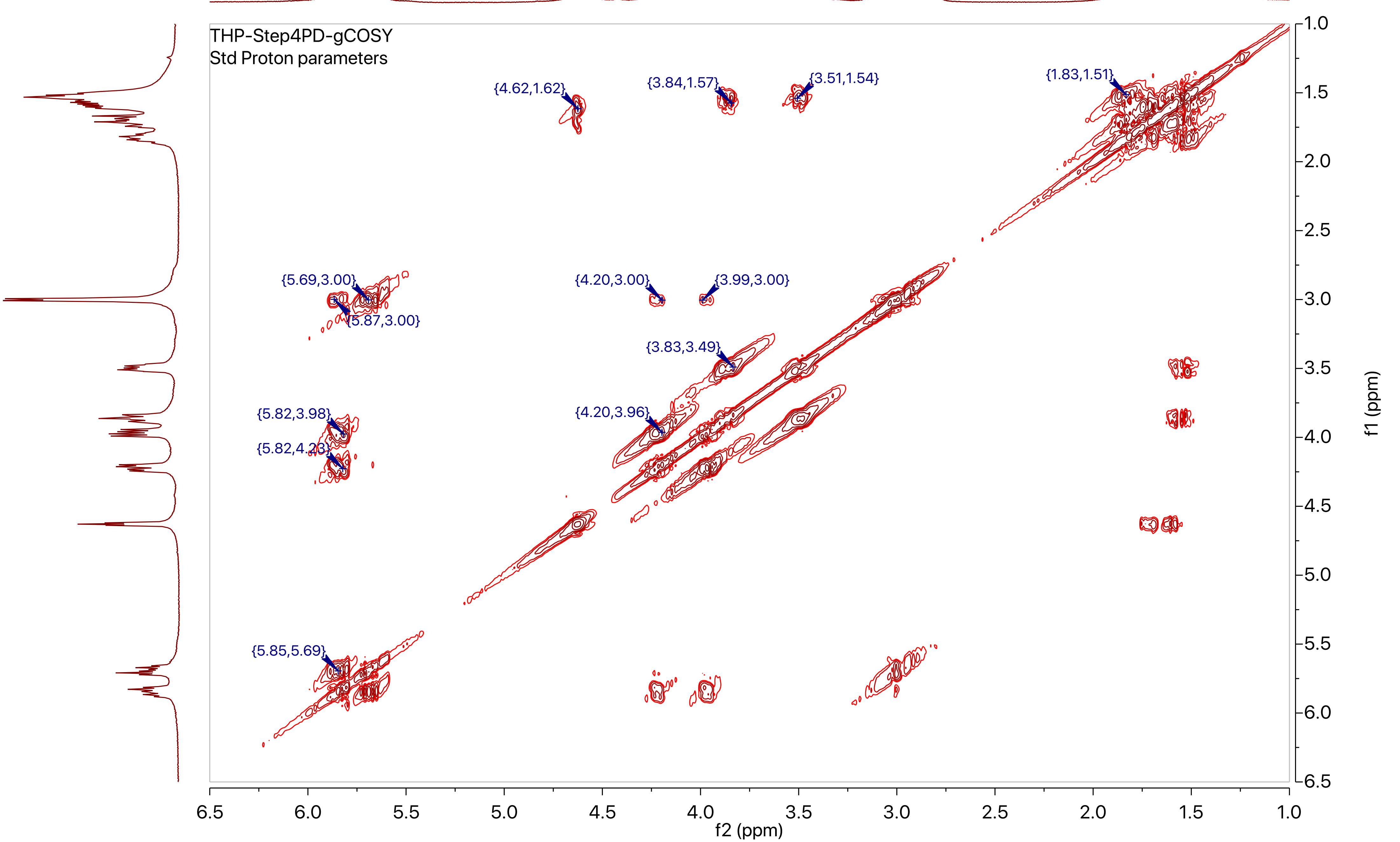

^{13}C NMR (101 MHz, $CDCl_3$)

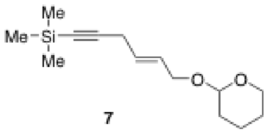

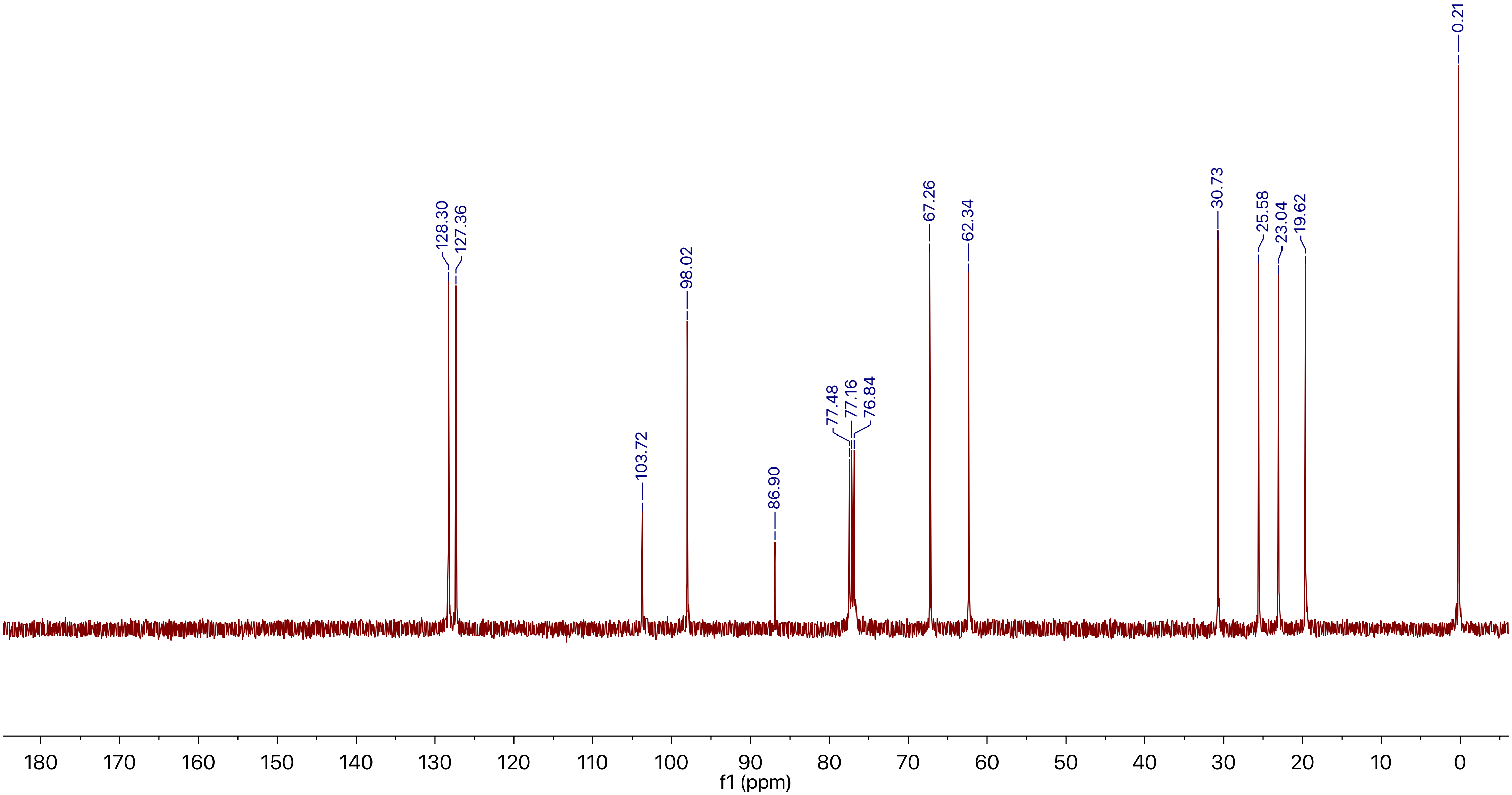

^1H NMR (400 MHz, $CDCl_3$)

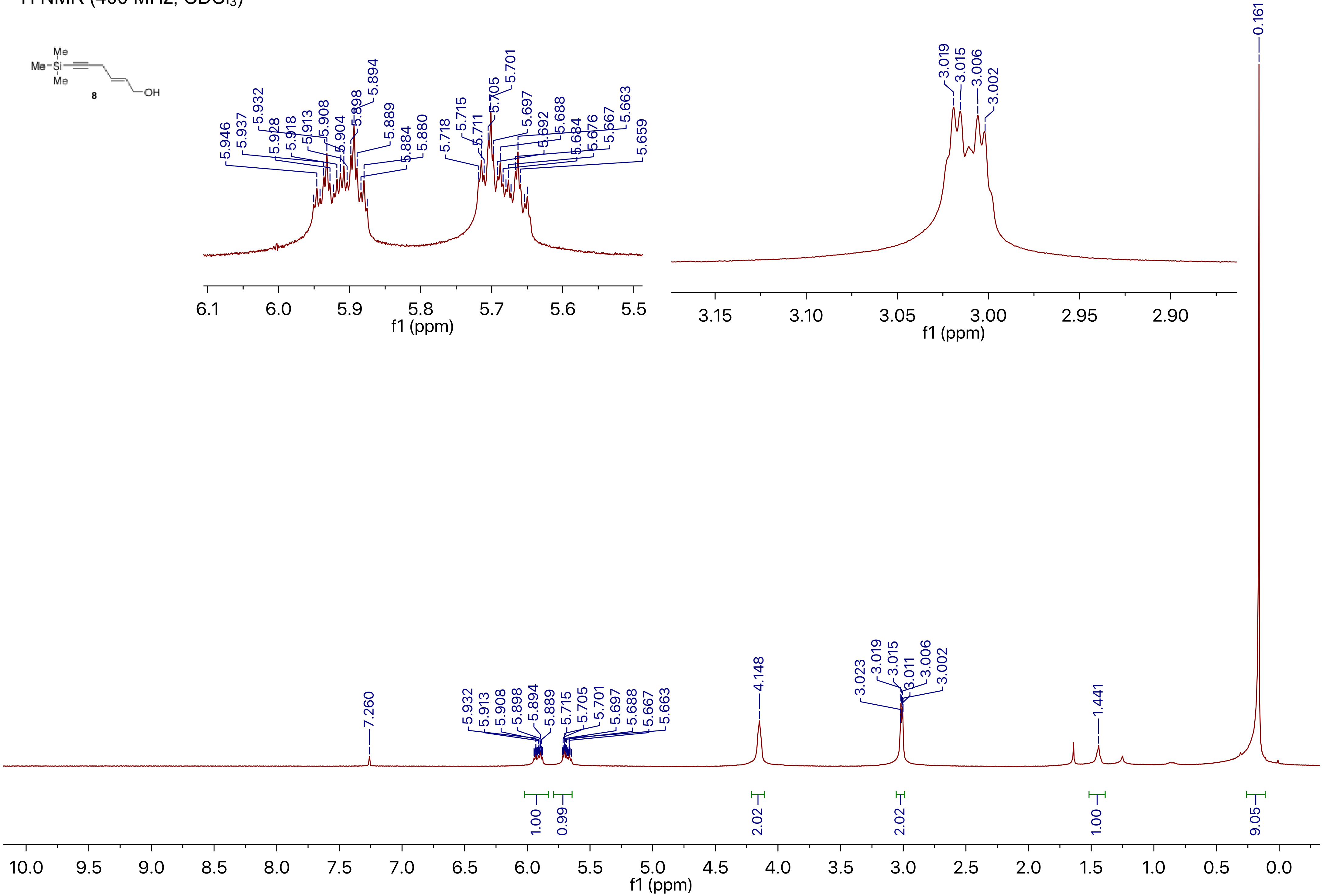

^{1}H NMR, gCOSY (400, MHz, $CDCl_3$)

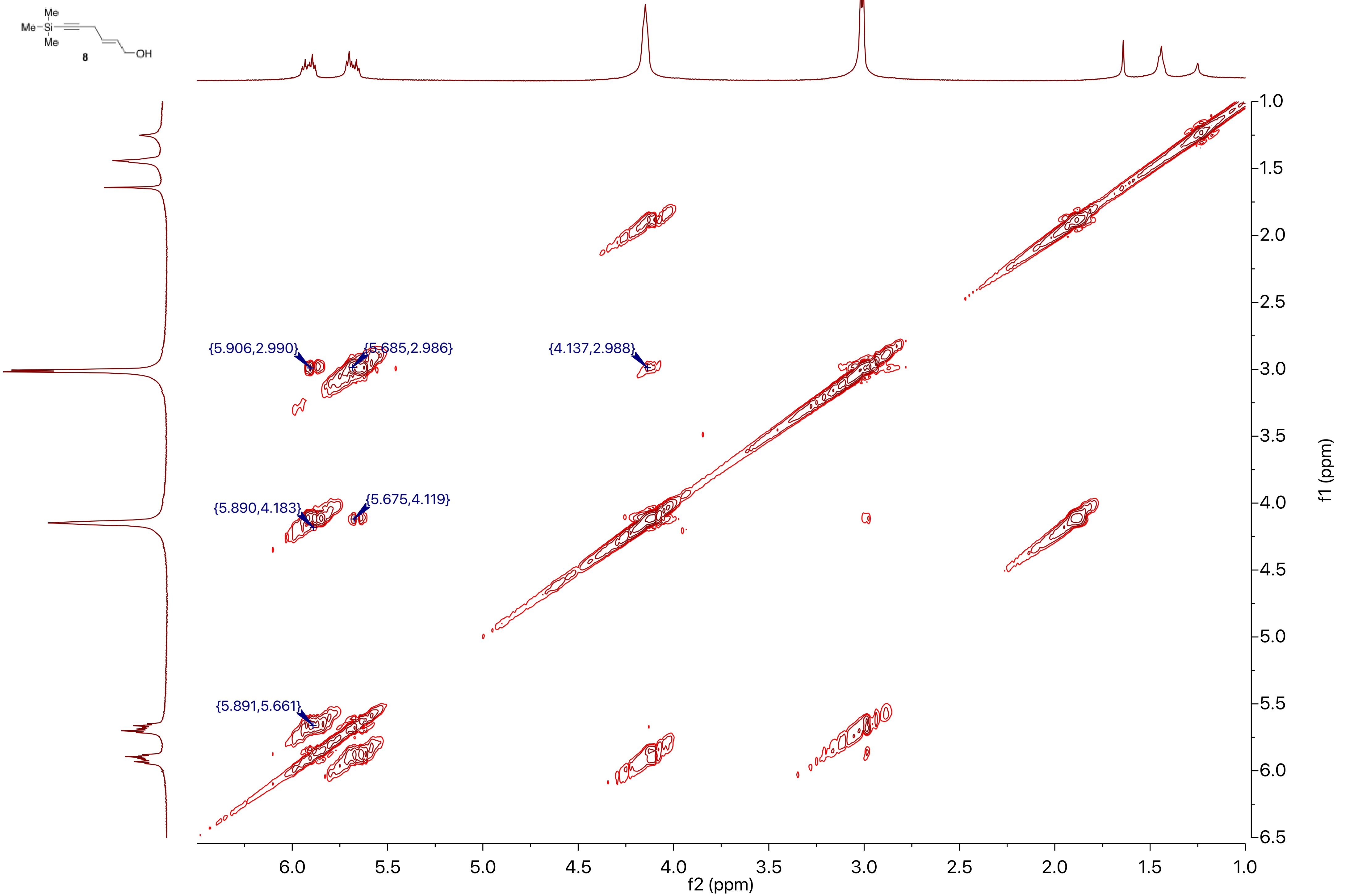

^{13}C NMR (101 MHz, $CDCl_3$)

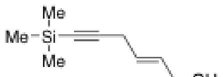

8

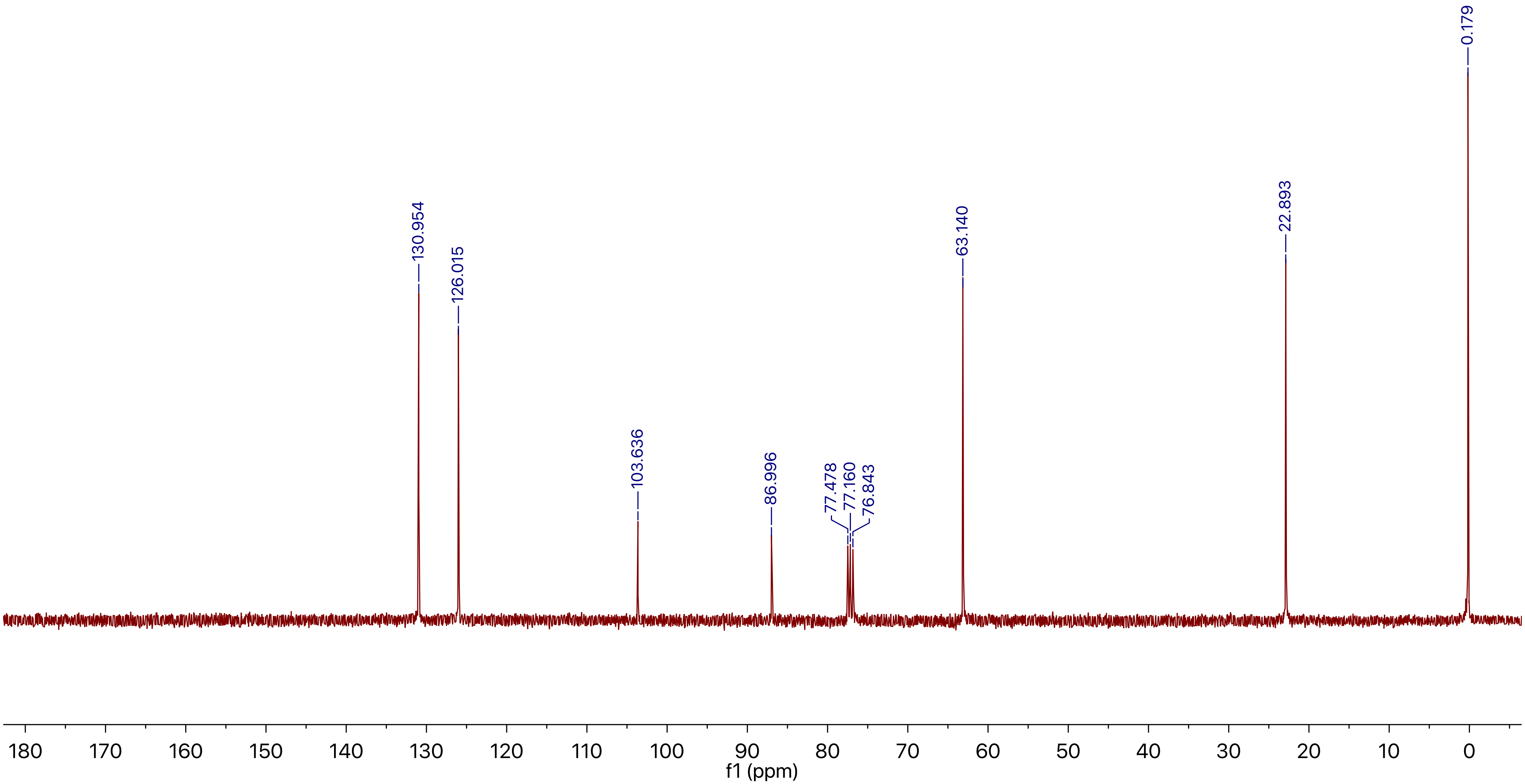

^{1}H NMR (400 MHz, $CDCl_3$)

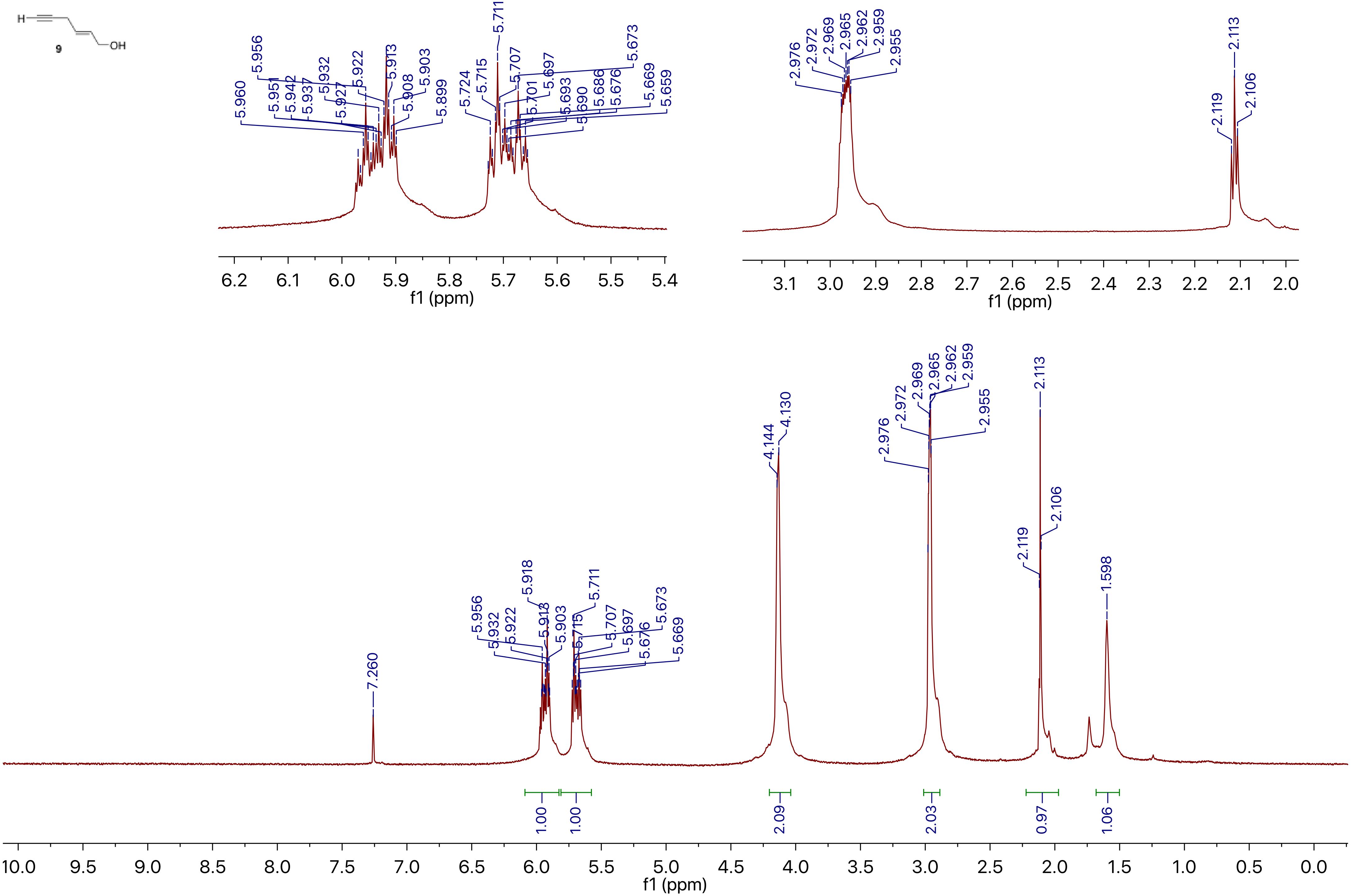

^{1}H NMR, gCOSY (400, MHz, $CDCl_3$)

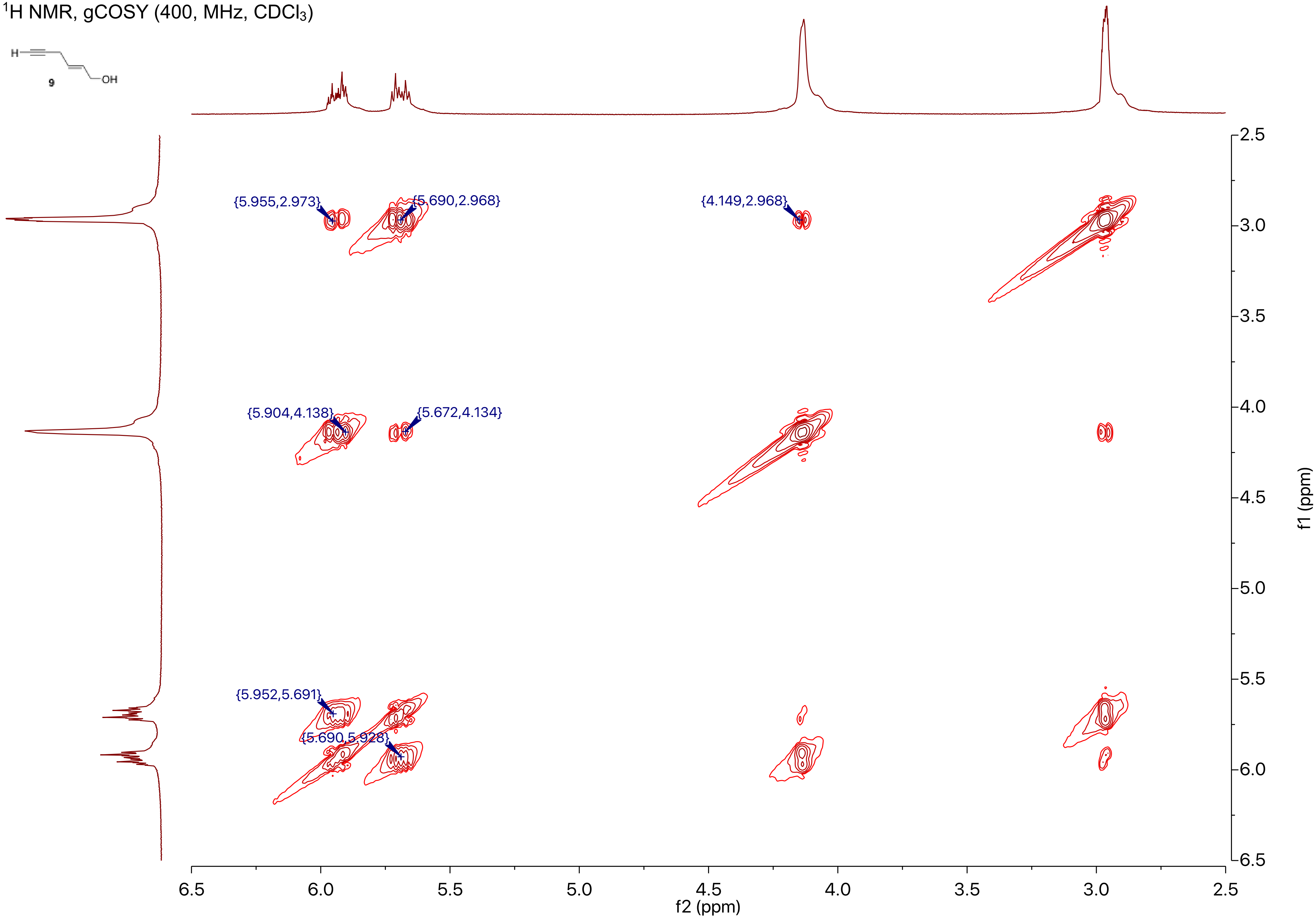

^{13}C NMR (101 MHz, $CDCl_3$)

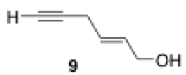

9

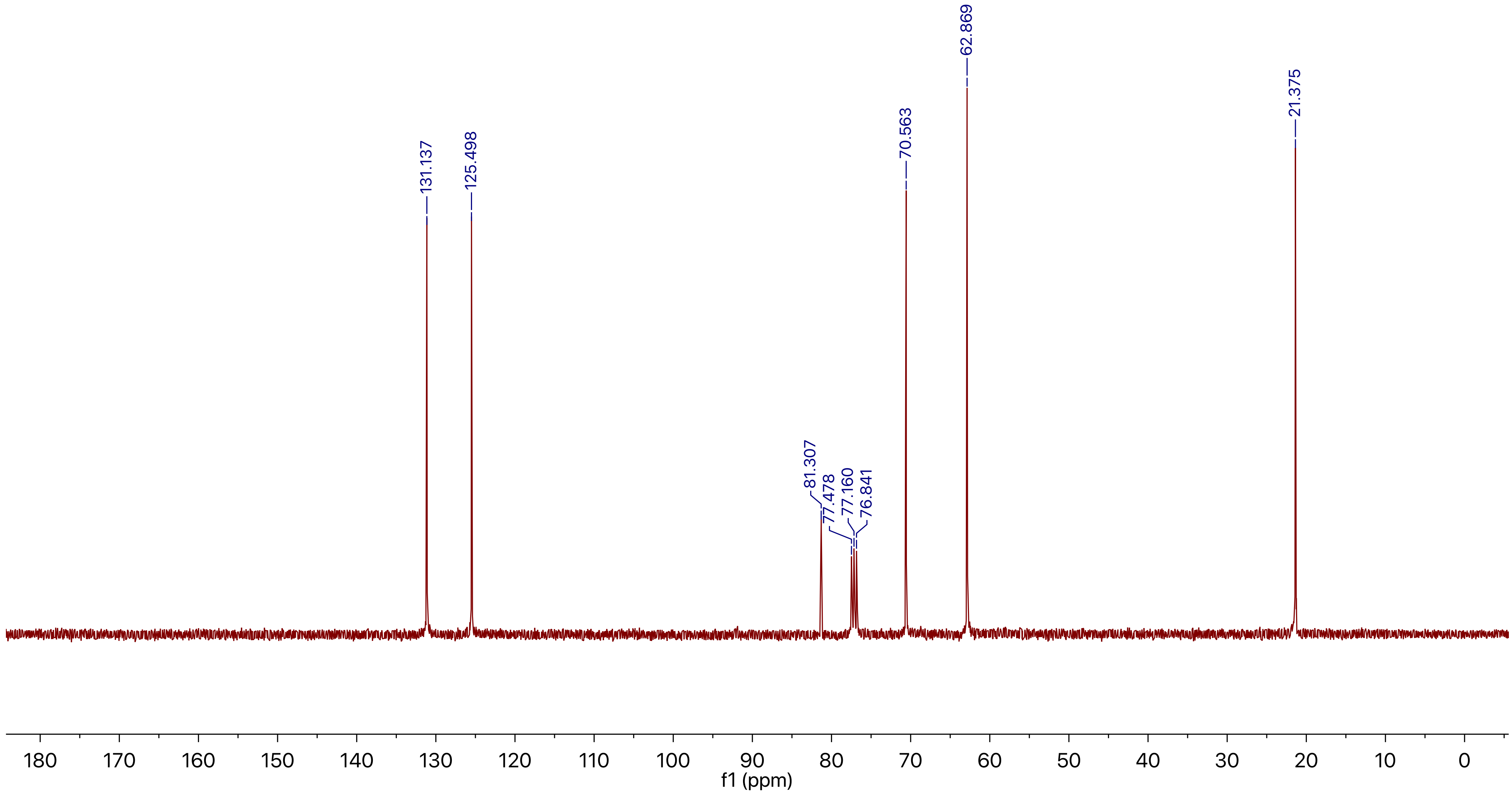

^{1}H NMR (400 MHz, $CDCl_3$)

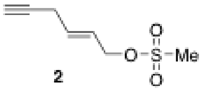

2

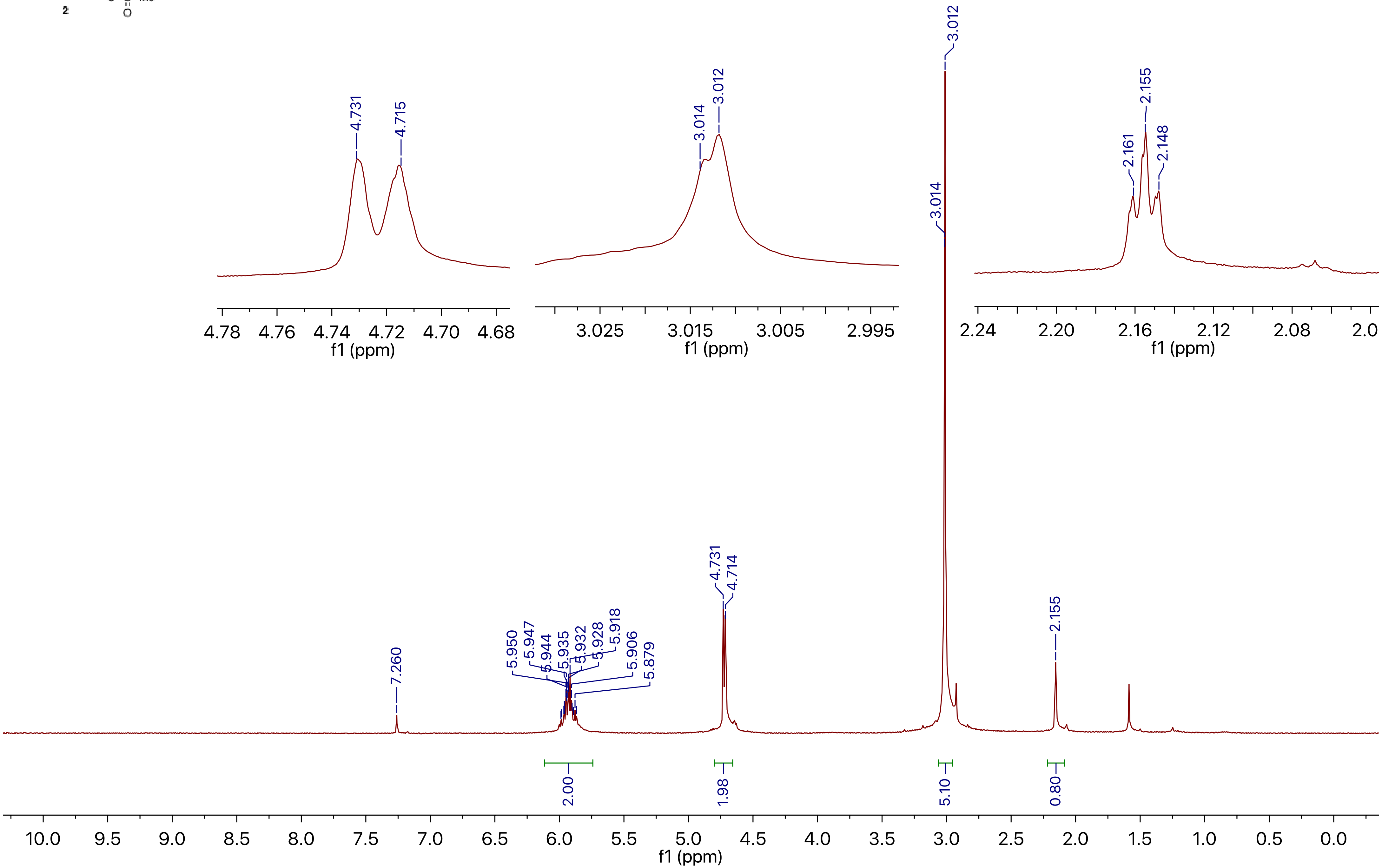

^{1}H NMR, gCOSY (400, MHz, CDCl$_3$)

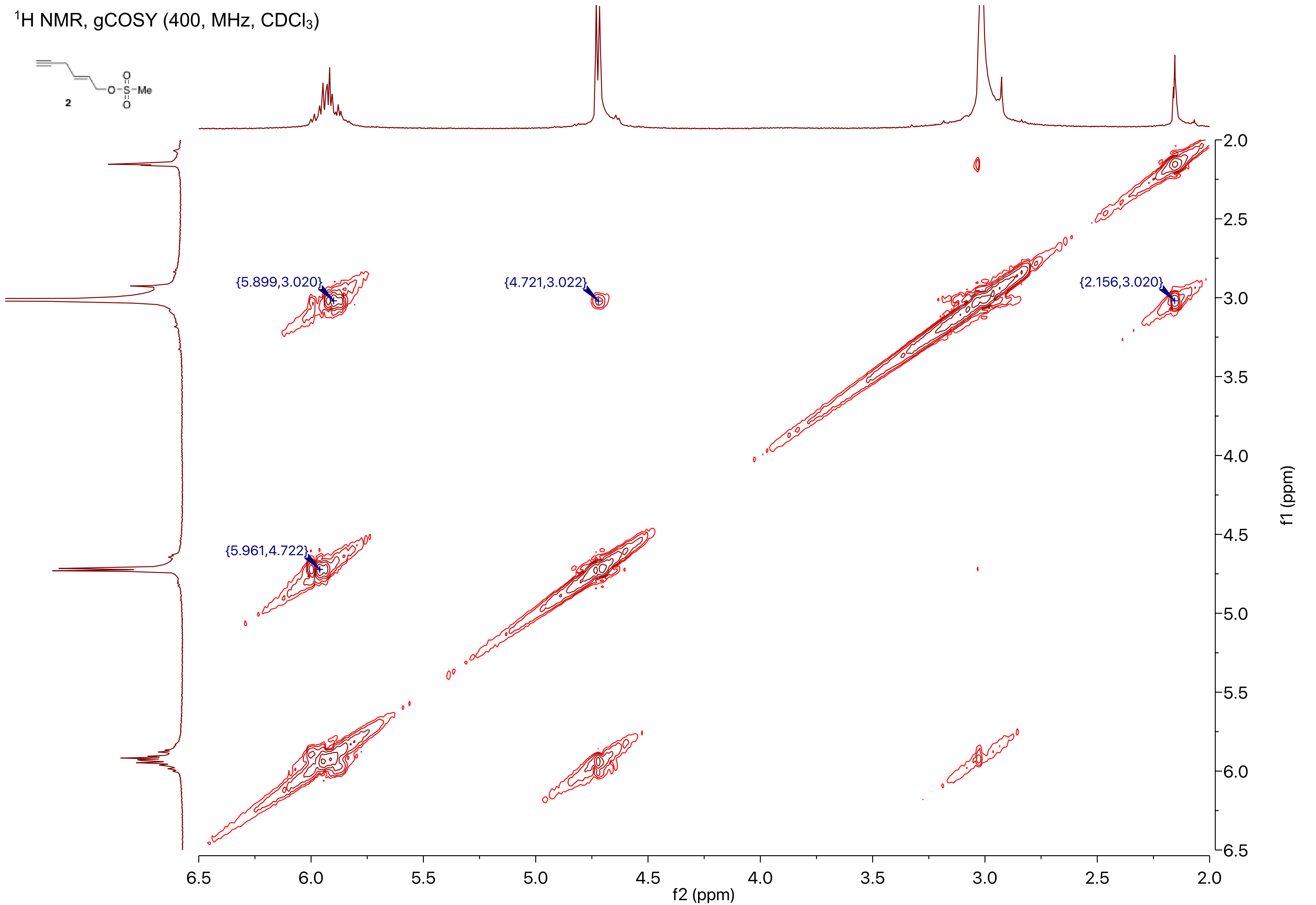

^{13}C NMR (101 MHz, $CDCl_3$)

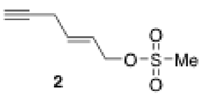

2

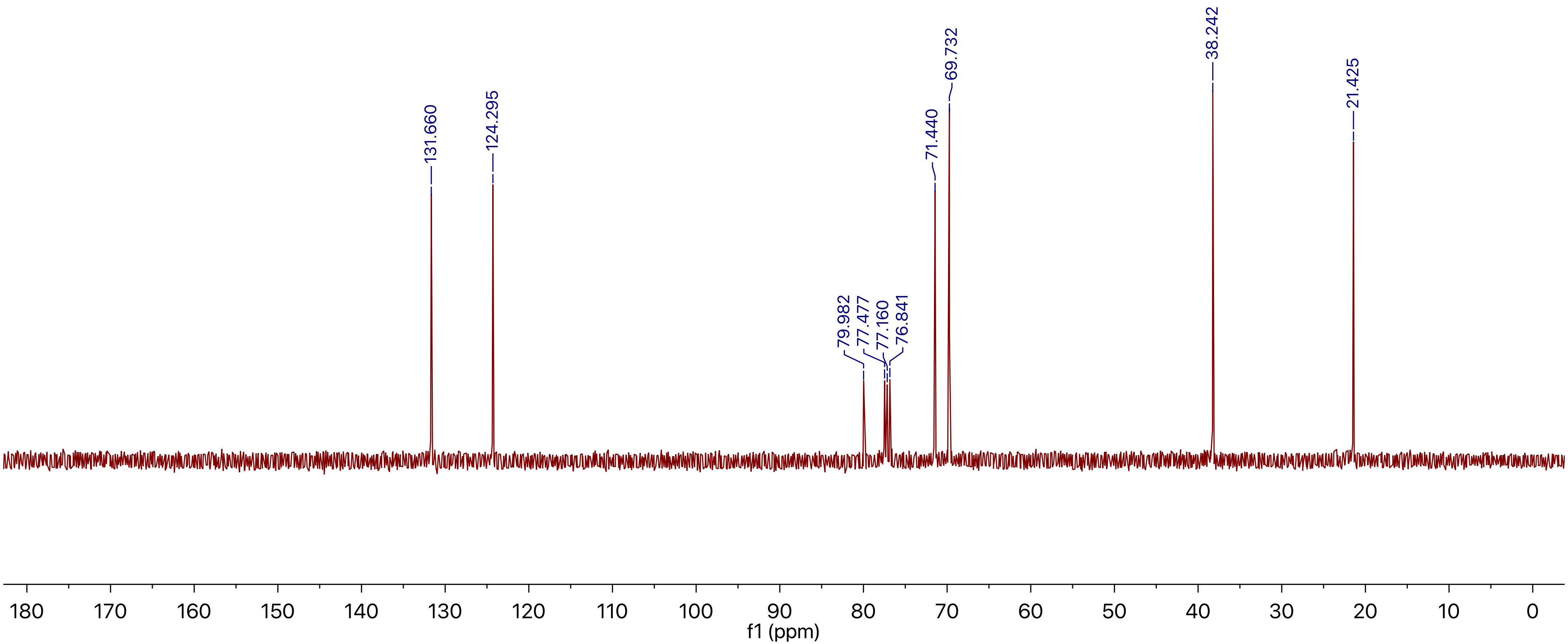

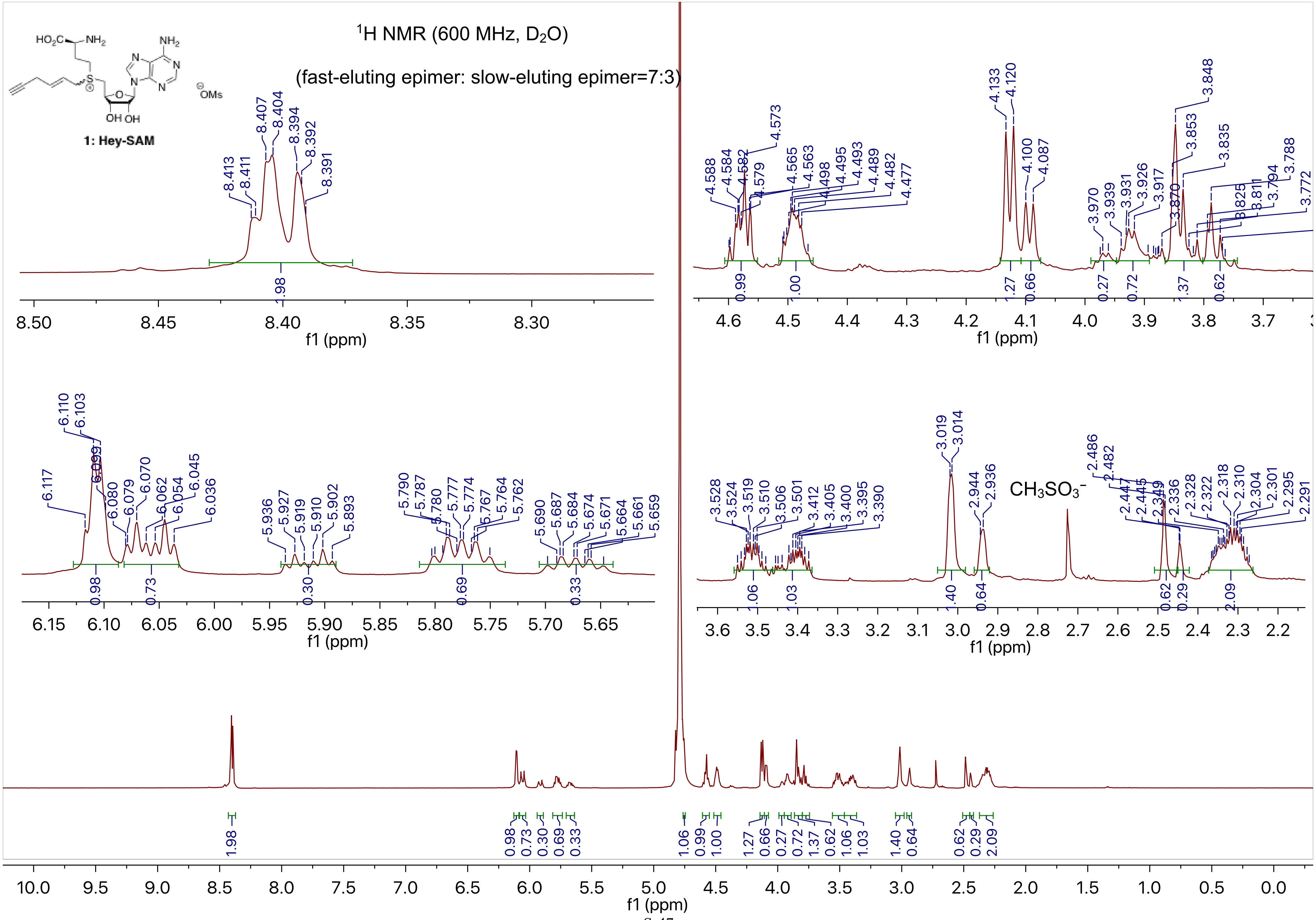

1H NMR (600 MHz, D2O)
(fast-eluting epimer: slow-eluting epimer=7:3)
1: Hey-SAM
OMs
CH3SO3−
f1 (ppm)

^{1}H NMR COSY (600 MHz, D_2O)

(fast-eluting epimer: slow-eluting epimer=7:3)

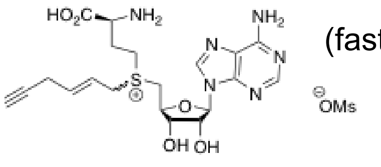

1: Hey-SAM

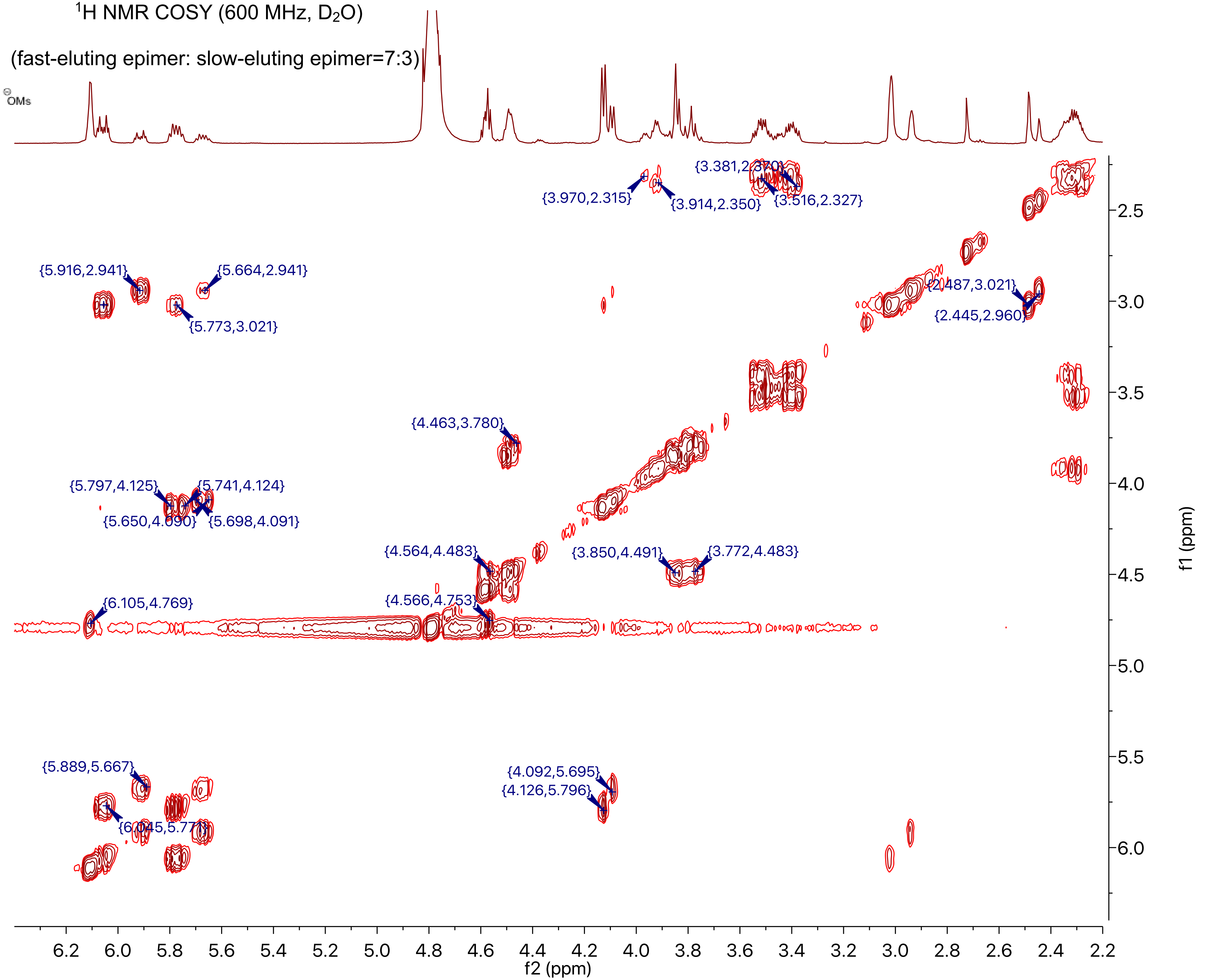

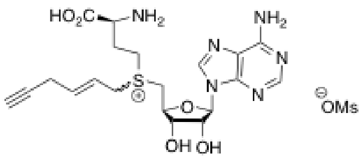

1: Hey-SAM

^{13}C NMR (151 MHz, D_2O) (fast-eluting epimer: slow-eluting epimer=7:3)

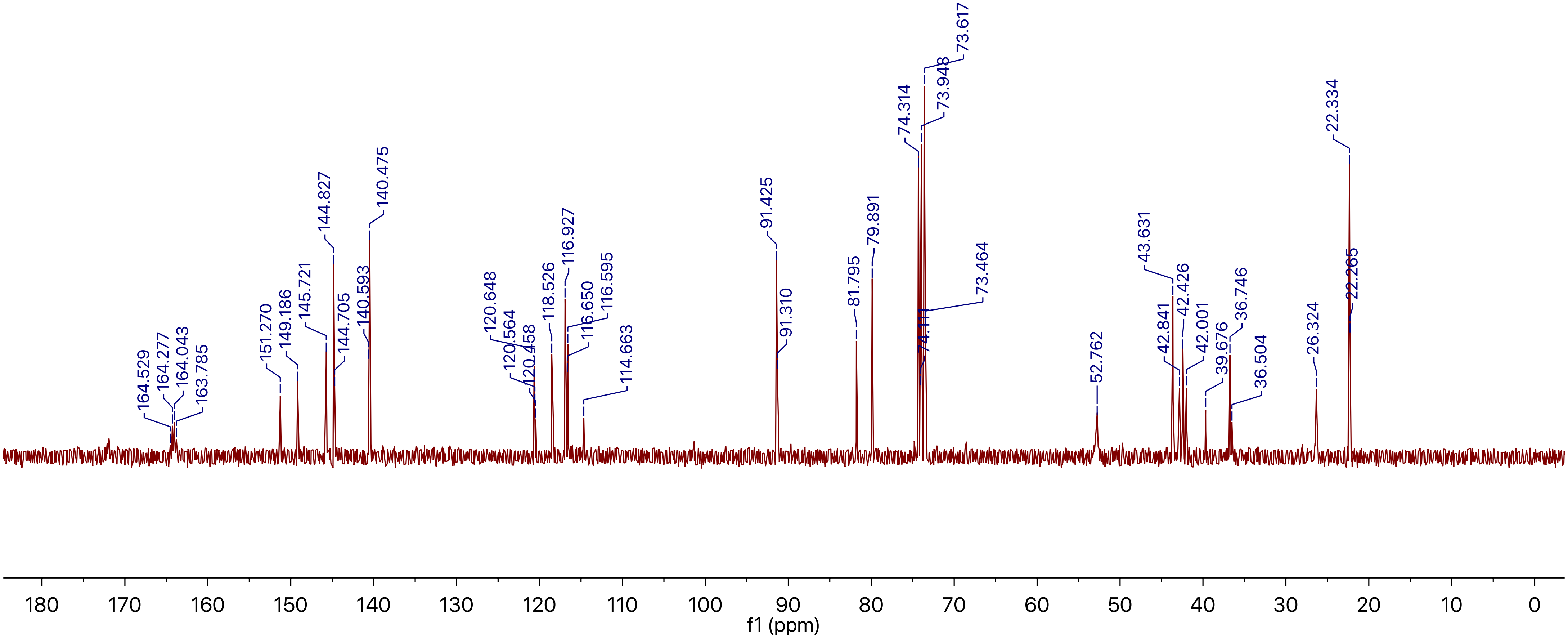

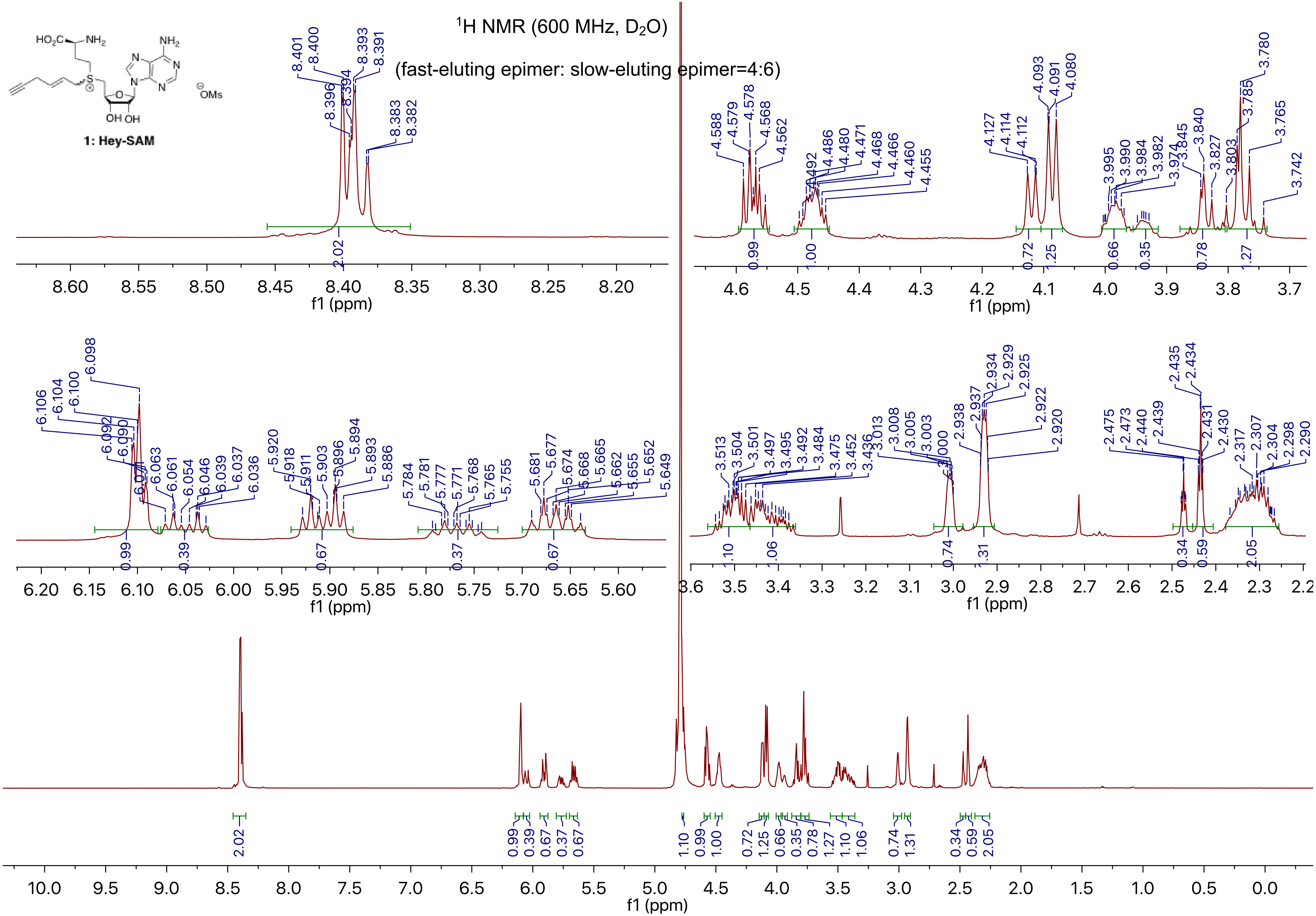

^{1}H NMR (600 MHz, D_2O)
(fast-eluting epimer: slow-eluting epimer=4:6)
1: Hey-SAM
8.401 8.400 8.396 8.394 8.393 8.391 8.383 8.382
2.02
8.60 8.55 8.50 8.45 8.40 8.35 8.30 8.25 8.20
f1 (ppm)
4.588 4.579 4.578 4.568 4.562 4.492 4.486 4.480 4.471 4.468 4.466 4.460 4.455
4.127 4.114 4.112 4.093 4.091 4.080
3.995 3.990 3.984 3.982 3.974 3.845 3.840 3.827 3.803 3.785 3.780 3.765 3.742
0.99 1.00 0.72 1.25 0.66 0.35 0.78 1.27
4.6 4.5 4.4 4.3 4.2 4.1 4.0 3.9 3.8 3.7
f1 (ppm)
6.106 6.104 6.100 6.098 6.092 6.090 6.071 6.063 6.061 6.054 6.046 6.039 6.037 6.036
5.920 5.918 5.911 5.903 5.896 5.894 5.893 5.886
5.784 5.781 5.777 5.771 5.768 5.765 5.755
5.681 5.677 5.674 5.668 5.665 5.662 5.655 5.652 5.649
0.99 0.39 0.67 0.37 0.67
6.20 6.15 6.10 6.05 6.00 5.95 5.90 5.85 5.80 5.75 5.70 5.65 5.60
f1 (ppm)
3.513 3.504 3.501 3.497 3.495 3.492 3.484 3.475 3.452 3.436 3.013 3.008 3.005 3.003 3.000
2.938 2.937 2.934 2.929 2.925 2.922 2.920
2.475 2.473 2.440 2.439 2.435 2.434 2.431 2.430 2.317 2.307 2.304 2.298 2.290
1.10 1.06 0.74 1.31 0.34 0.59 2.05
3.6 3.5 3.4 3.3 3.2 3.1 3.0 2.9 2.8 2.7 2.6 2.5 2.4 2.3 2.2
f1 (ppm)
2.02 0.99 0.39 0.67 0.37 0.67 1.10 0.99 1.00 0.72 1.25 0.66 0.35 0.78 1.27 1.10 1.06 0.74 1.31 0.34 0.59 2.05
10.0 9.5 9.0 8.5 8.0 7.5 7.0 6.5 6.0 5.5 5.0 4.5 4.0 3.5 3.0 2.5 2.0 1.5 1.0 0.5 0.0
f1 (ppm)

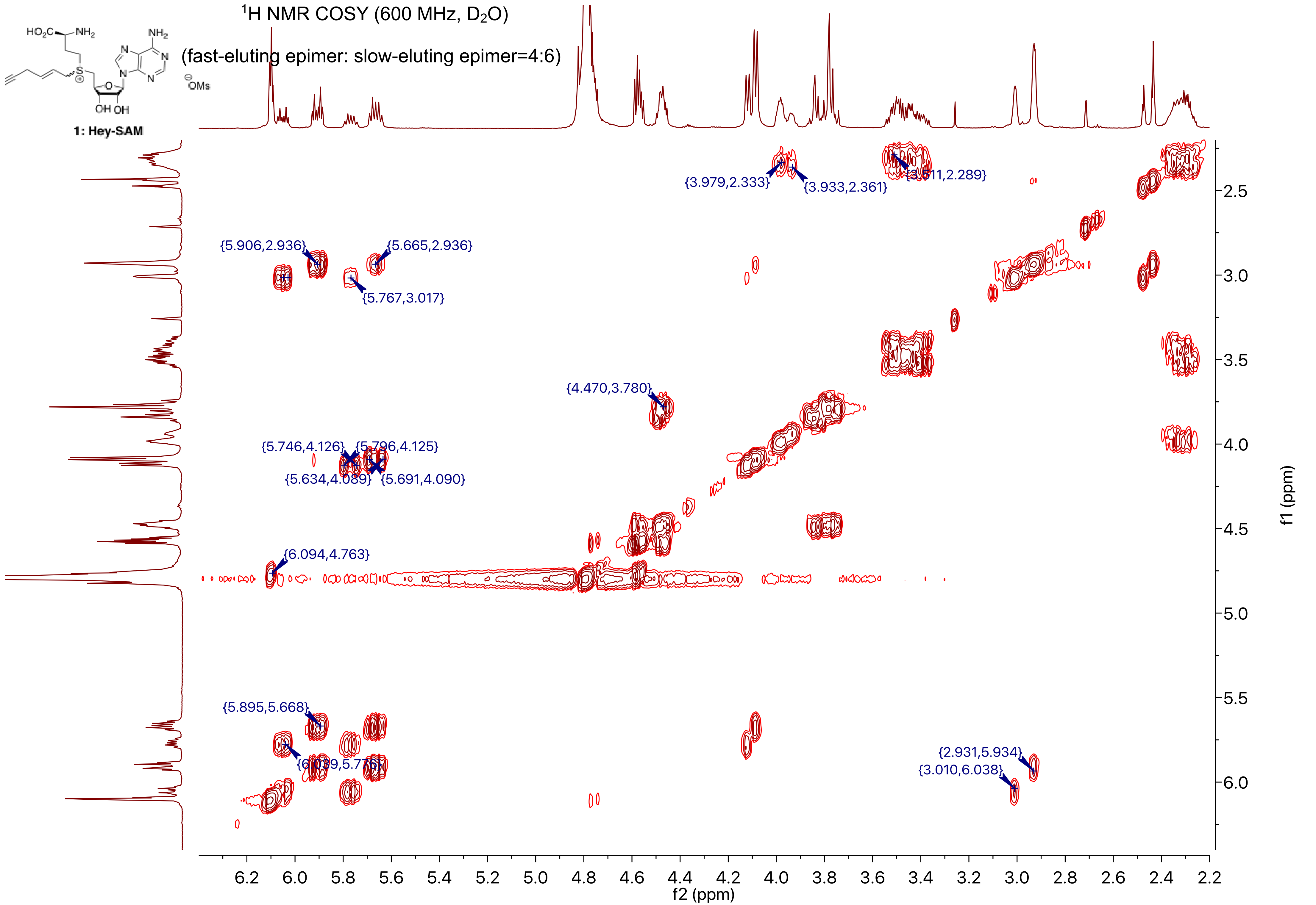

1H NMR COSY (600 MHz, D2O)
(fast-eluting epimer: slow-eluting epimer=4:6)
1: Hey-SAM
{3.979,2.333}
{3.933,2.361}
{3.511,2.289}
{5.906,2.936}
{5.665,2.936}
{5.767,3.017}
{4.470,3.780}
{5.746,4.126}
{5.796,4.125}
{5.634,4.089}
{5.691,4.090}
{6.094,4.763}
{5.895,5.668}
{6.039,5.776}
{2.931,5.934}
{3.010,6.038}
f2 (ppm)
f1 (ppm)

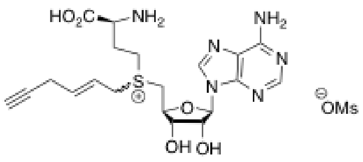

1: Hey-SAM

^{13}C NMR (151 MHz, D_2O) (fast-eluting epimer: slow-eluting epimer=4:6)

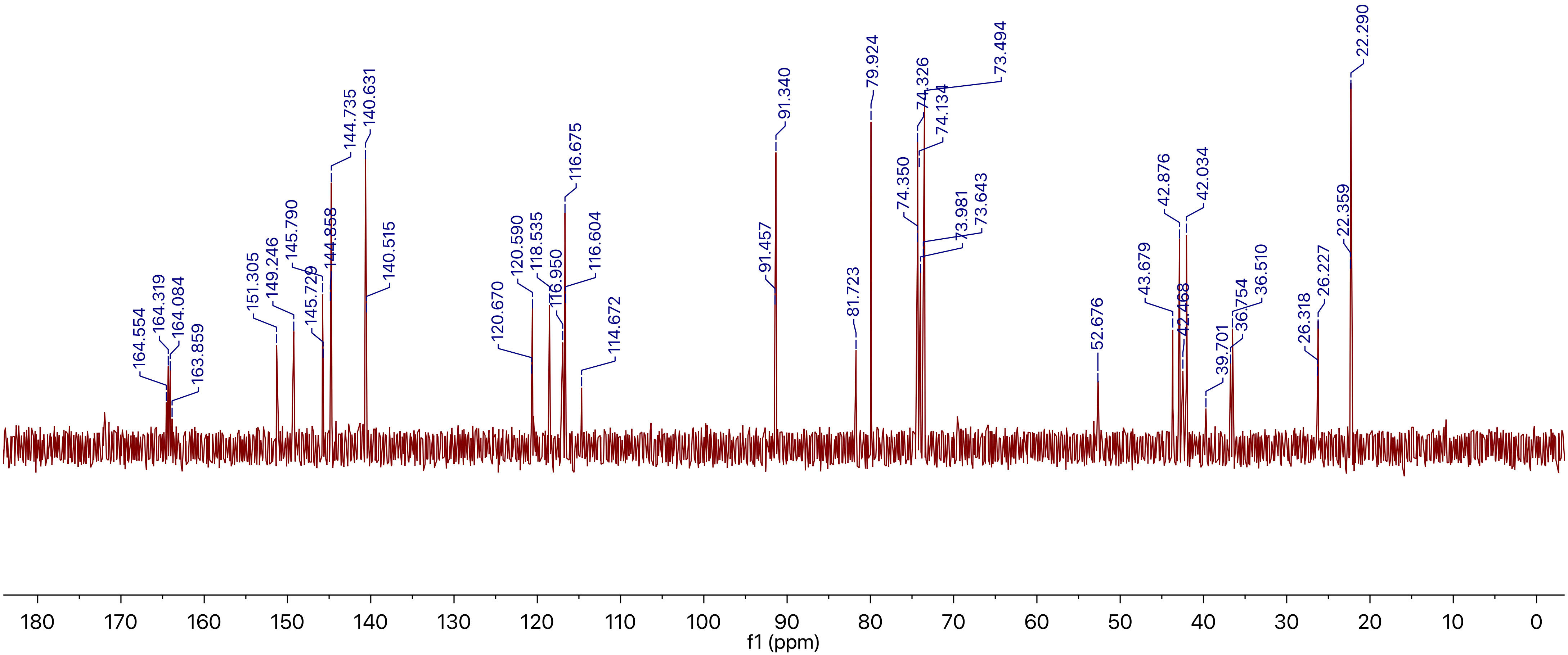

Figure S2A (TOF transformed)

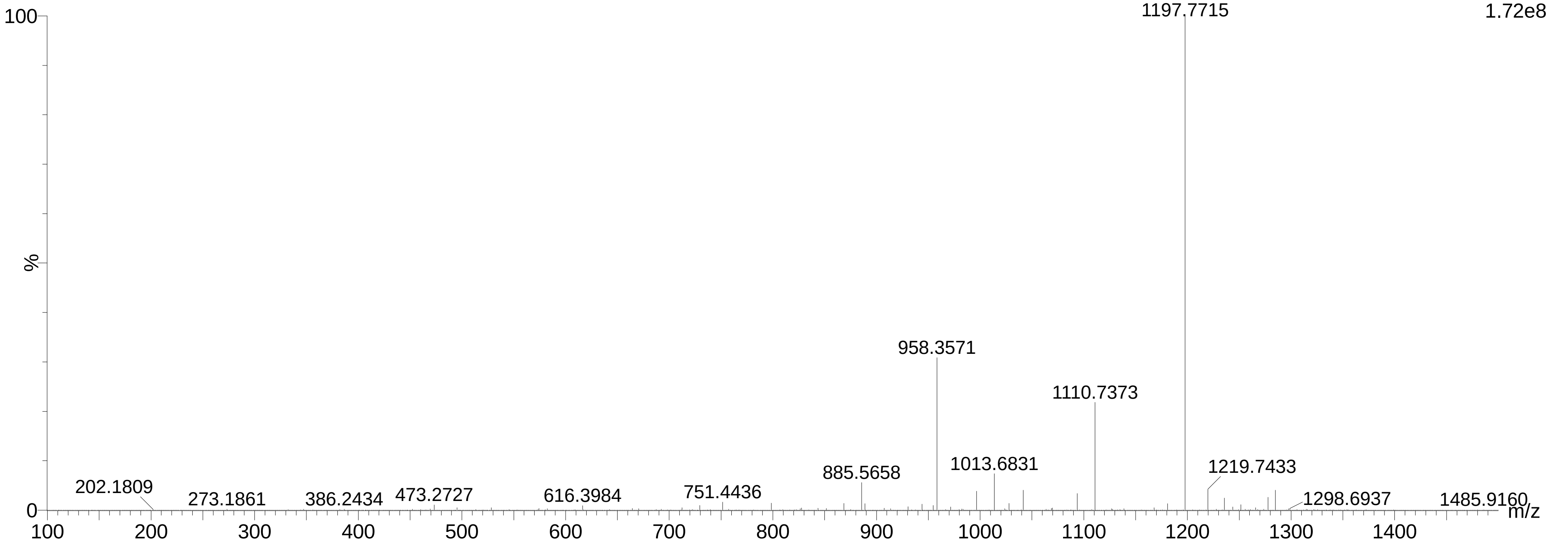

Figure S2A (Lock mass corrected)

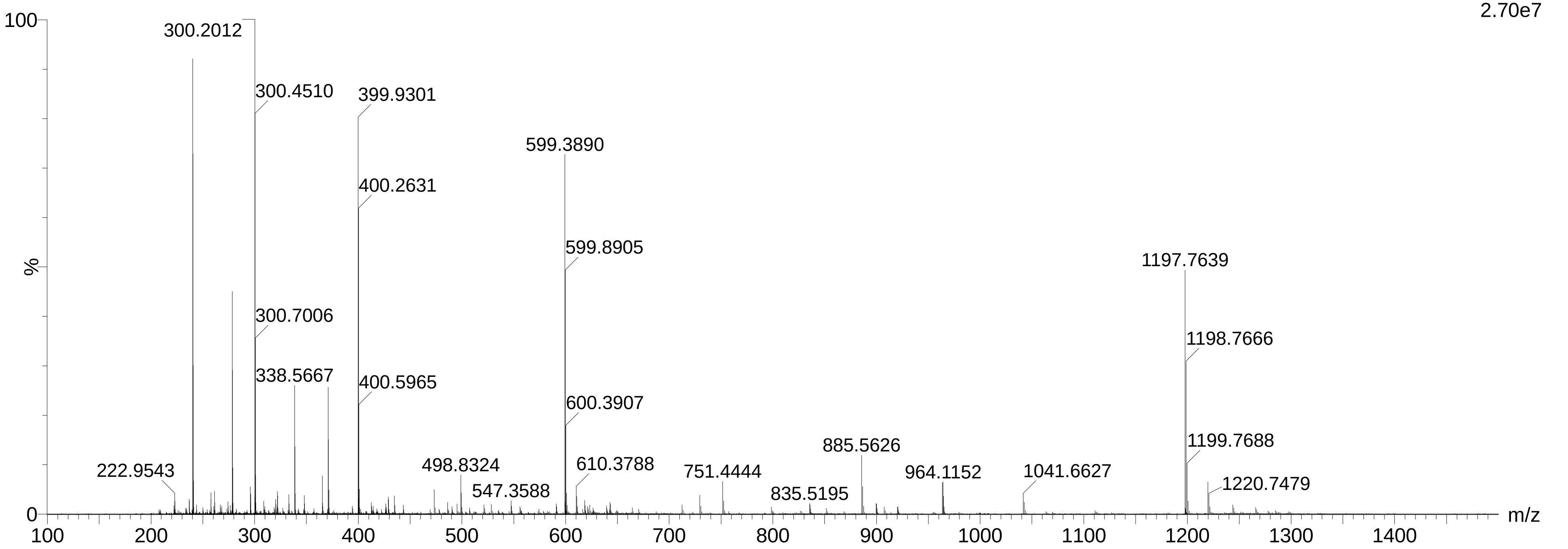

Figure S2B (TOF transformed)

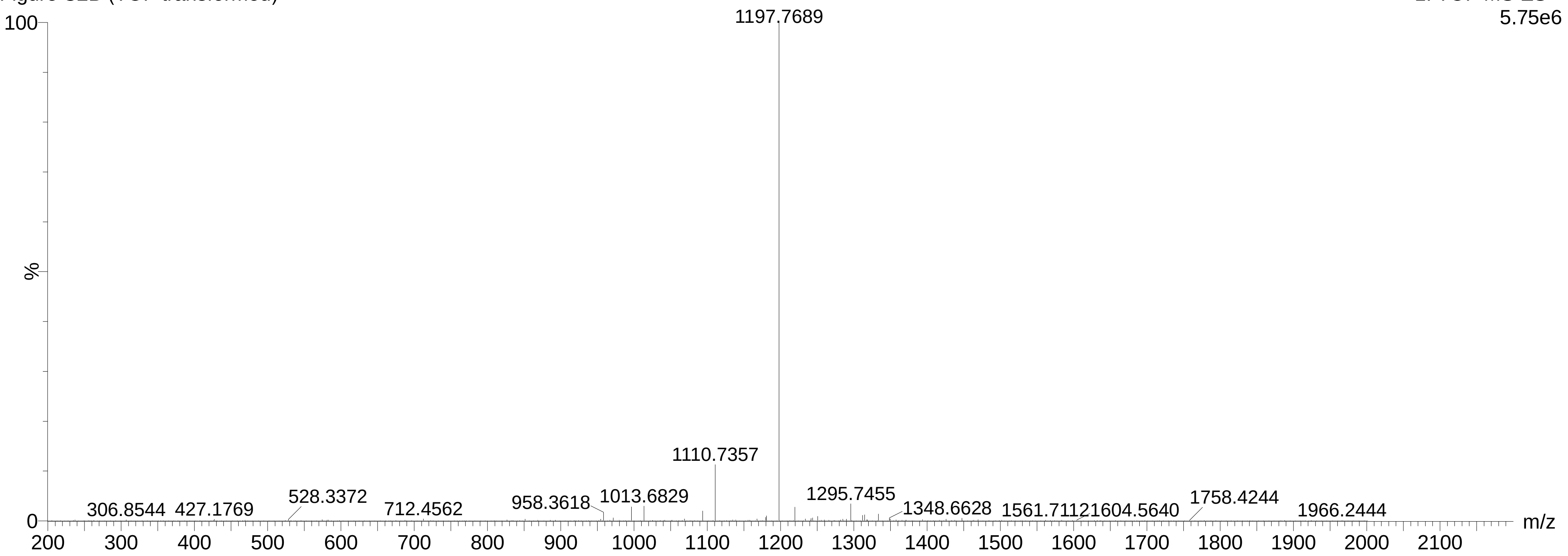

Figure S2B (Lock mass corrected)

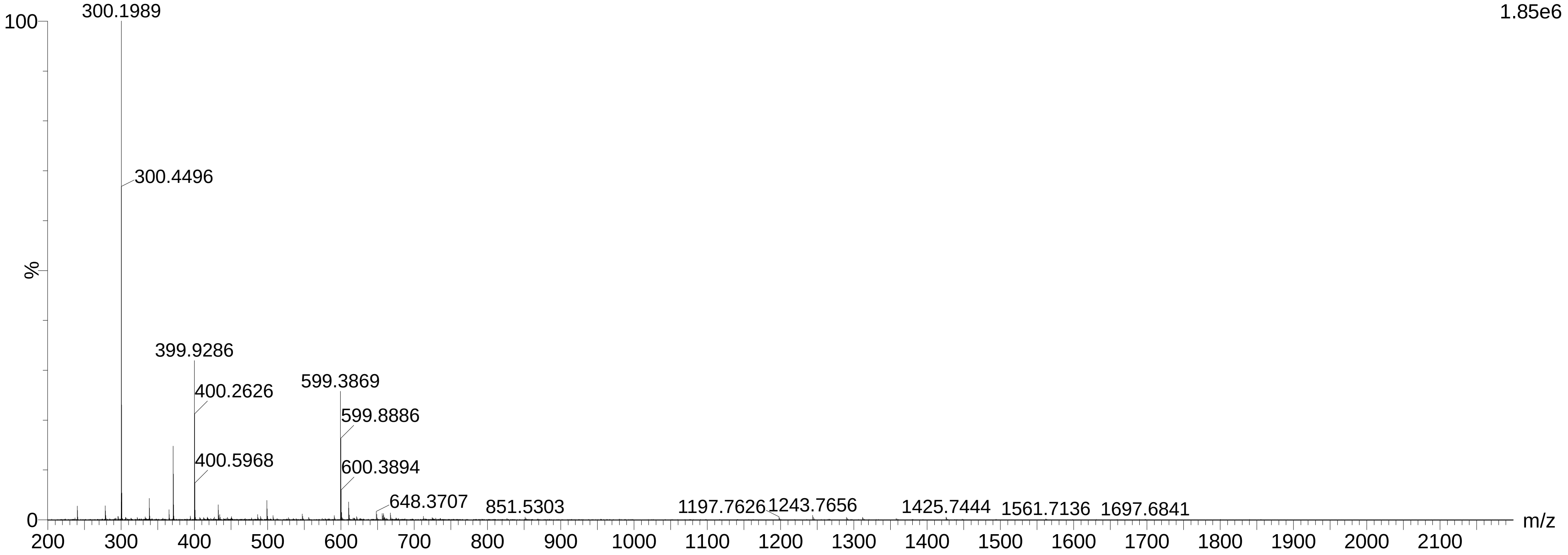

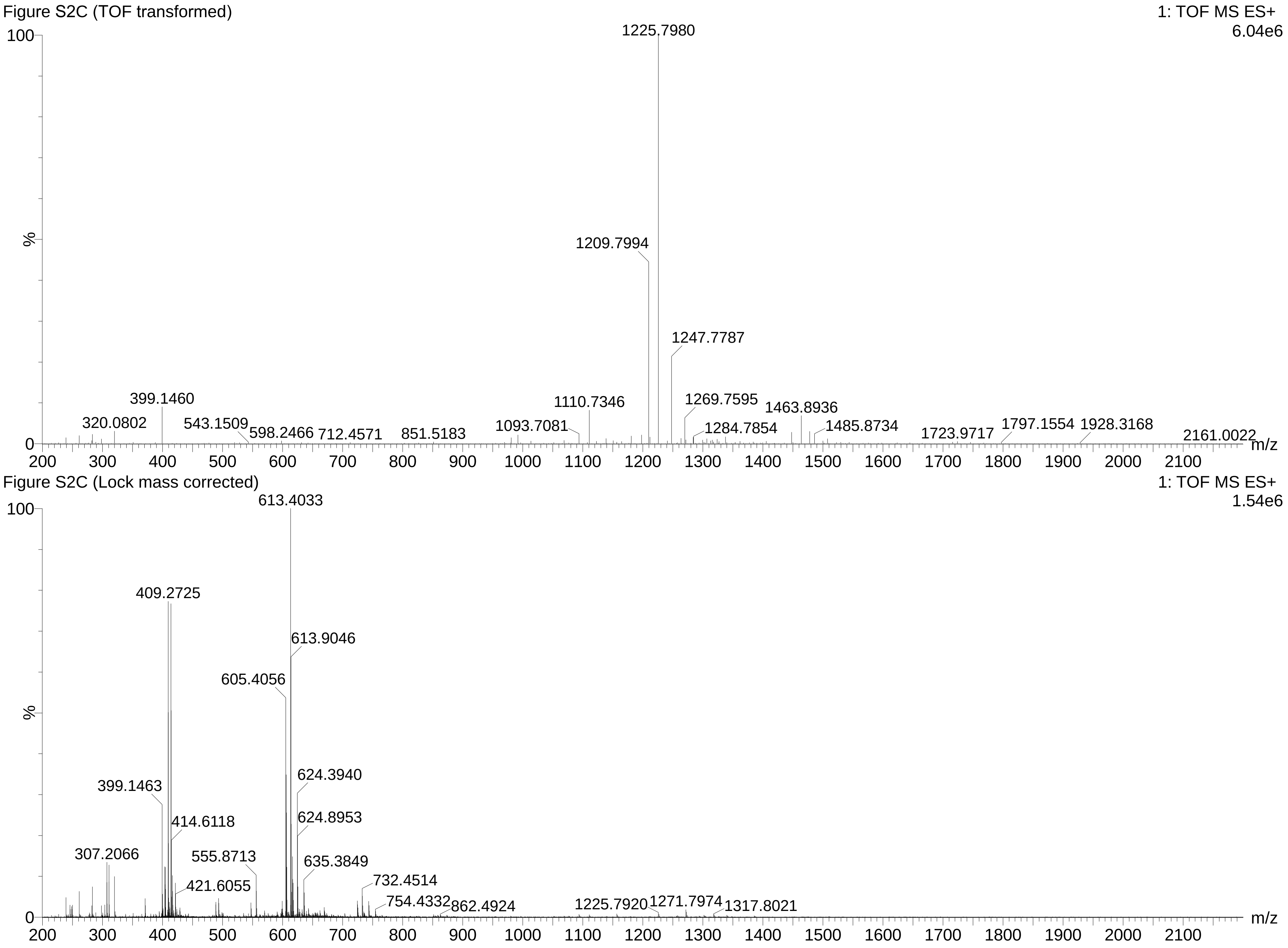

Figure S2C (TOF transformed)
1: TOF MS ES+
6.04e6
1225.7980
1209.7994
1247.7787
399.1460
320.0802
543.1509
598.2466
712.4571
851.5183
1093.7081
1110.7346
1269.7595
1284.7854
1463.8936
1485.8734
1723.9717
1797.1554
1928.3168
2161.0022
%
m/z
Figure S2C (Lock mass corrected)
1: TOF MS ES+
1.54e6
613.4033
409.2725
613.9046
605.4056
399.1463
624.3940
414.6118
624.8953
307.2066
555.8713
635.3849
421.6055
732.4514
754.4332
862.4924
1225.7920
1271.7974
1317.8021

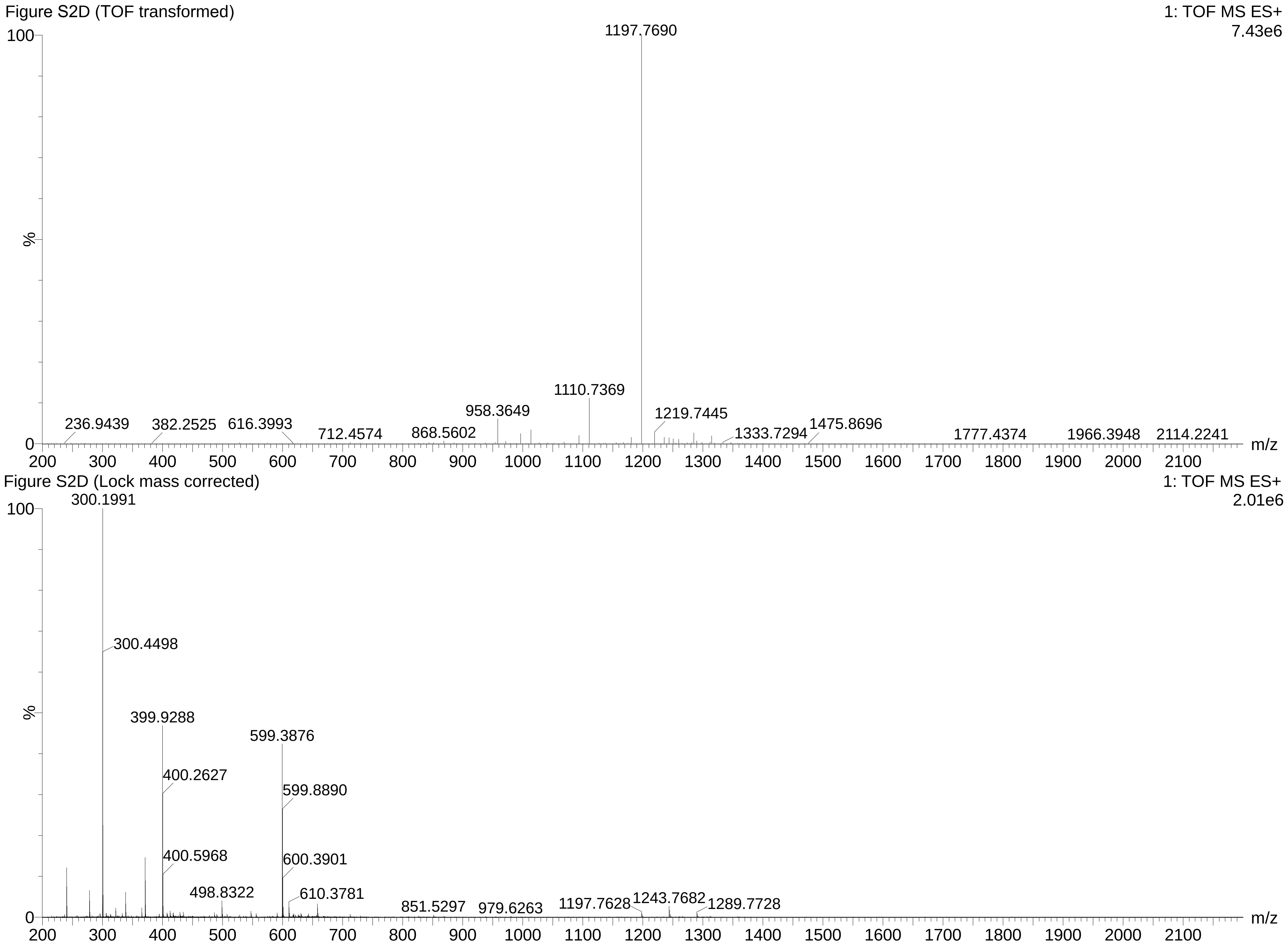
Figure S2D (TOF transformed)
1: TOF MS ES+
7.43e6
1197.7690
1110.7369
958.3649
1219.7445
236.9439
382.2525
616.3993
712.4574
868.5602
1333.7294
1475.8696
1777.4374
1966.3948
2114.2241
%
m/z
Figure S2D (Lock mass corrected)
1: TOF MS ES+
2.01e6
300.1991
300.4498
399.9288
599.3876
400.2627
599.8890
400.5968
600.3901
498.8322
610.3781
851.5297
979.6263
1197.7628
1243.7682
1289.7728
%
m/z

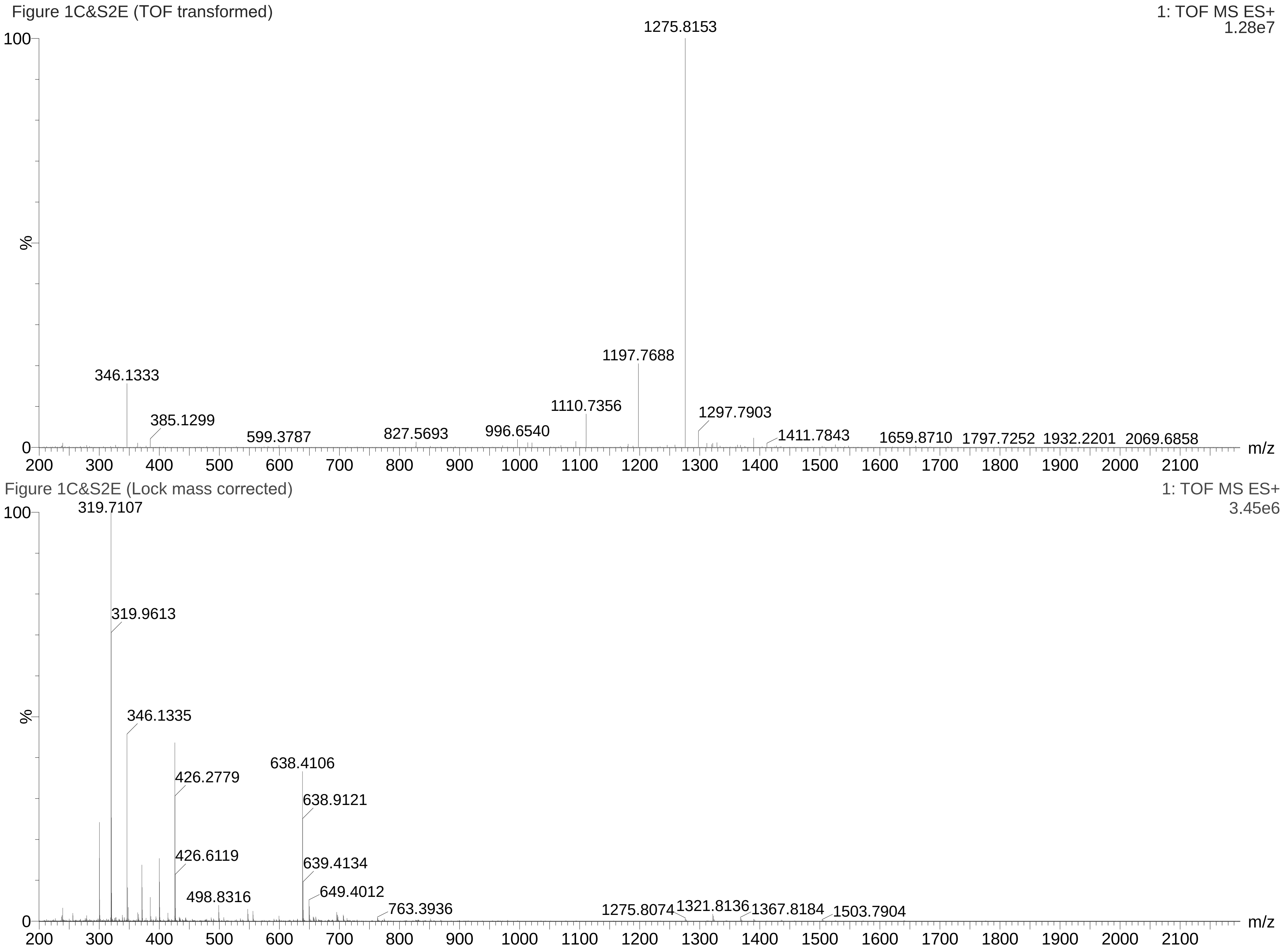
Figure 1C&S2E (TOF transformed)
1: TOF MS ES+
1.28e7
1275.8153
1197.7688
346.1333
1110.7356
385.1299
1297.7903
599.3787
827.5693
996.6540
1411.7843
1659.8710
1797.7252
1932.2201
2069.6858
%
m/z
Figure 1C&S2E (Lock mass corrected)
1: TOF MS ES+
3.45e6
319.7107
319.9613
346.1335
426.2779
638.4106
638.9121
426.6119
639.4134
649.4012
498.8316
763.3936
1275.8074
1321.8136
1367.8184
1503.7904
%
m/z

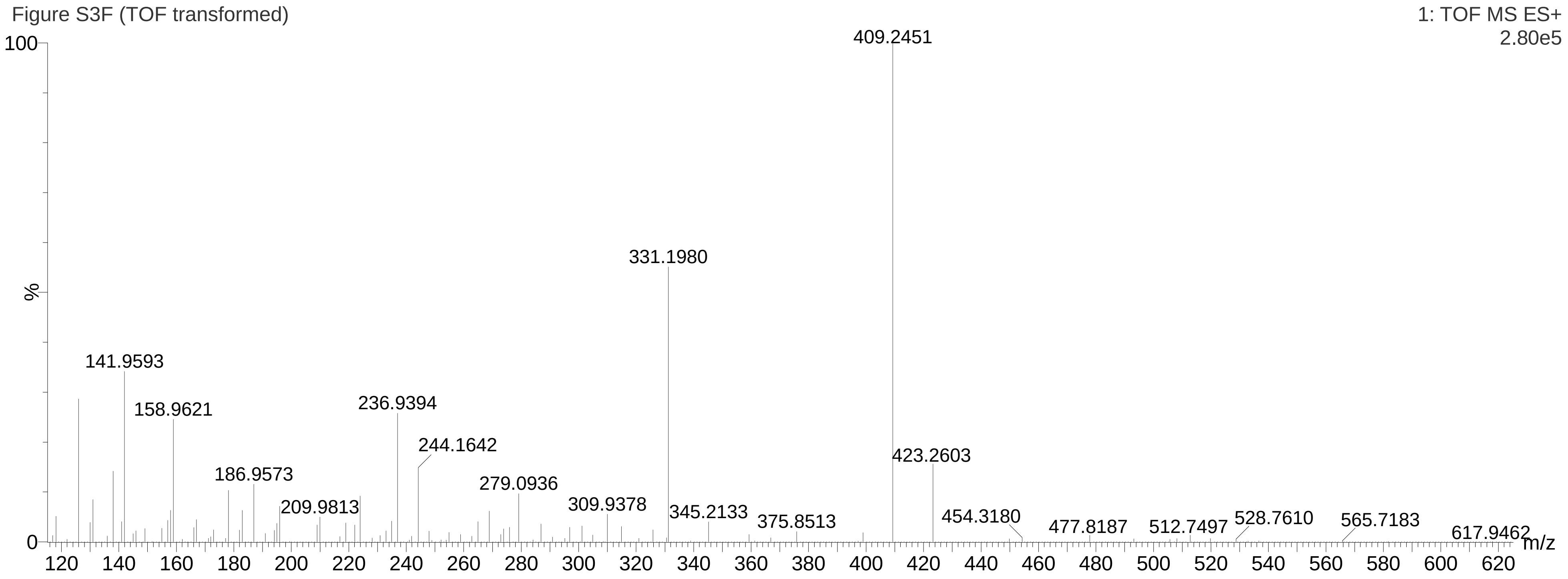
Figure S3F (TOF transformed)
1: TOF MS ES+
2.80e5
409.2451
331.1980
141.9593
158.9621
236.9394
244.1642
423.2603
186.9573
279.0936
209.9813
309.9378
345.2133
375.8513
454.3180
477.8187
512.7497
528.7610
565.7183
617.9462
100
%
0
m/z
120 140 160 180 200 220 240 260 280 300 320 340 360 380 400 420 440 460 480 500 520 540 560 580 600 620

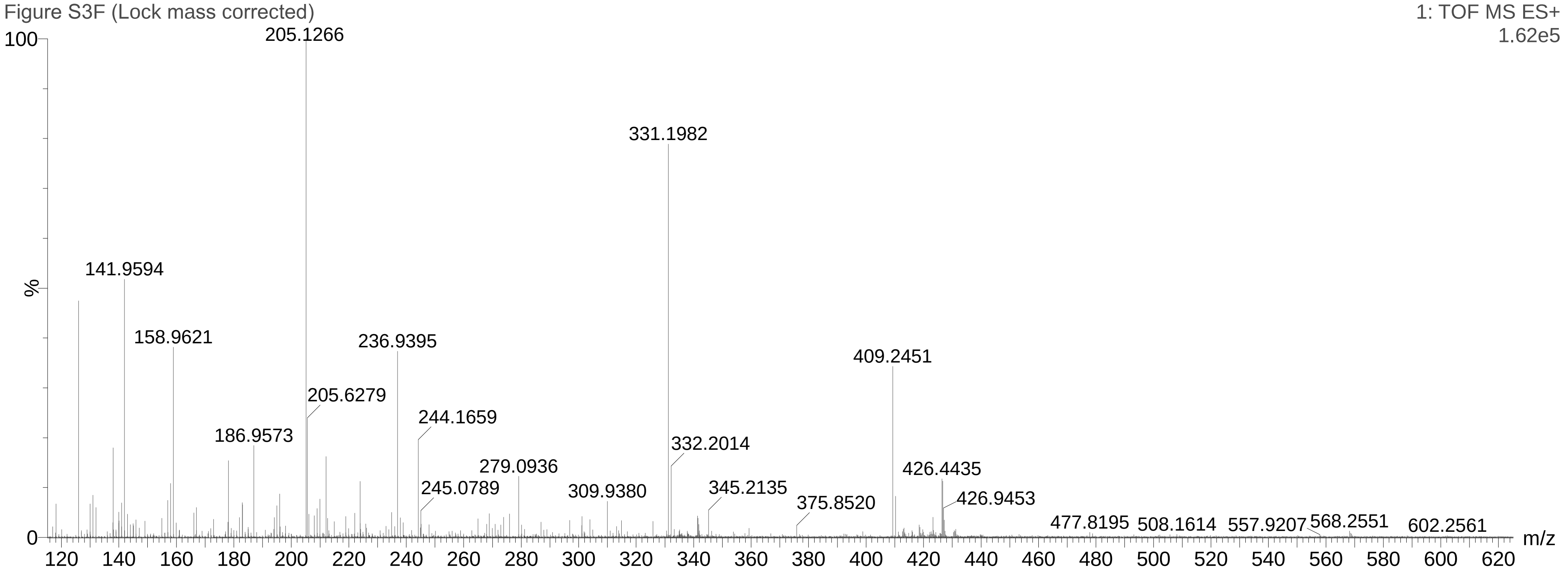
Figure S3F (Lock mass corrected)
1: TOF MS ES+
1.62e5
205.1266
331.1982
141.9594
158.9621
236.9395
409.2451
205.6279
244.1659
186.9573
332.2014
279.0936
426.4435
245.0789
309.9380
345.2135
426.9453
375.8520
477.8195
508.1614
557.9207
568.2551
602.2561
100
%
0
m/z
120 140 160 180 200 220 240 260 280 300 320 340 360 380 400 420 440 460 480 500 520 540 560 580 600 620

Figure S4C (Precursor, TOF transformed)

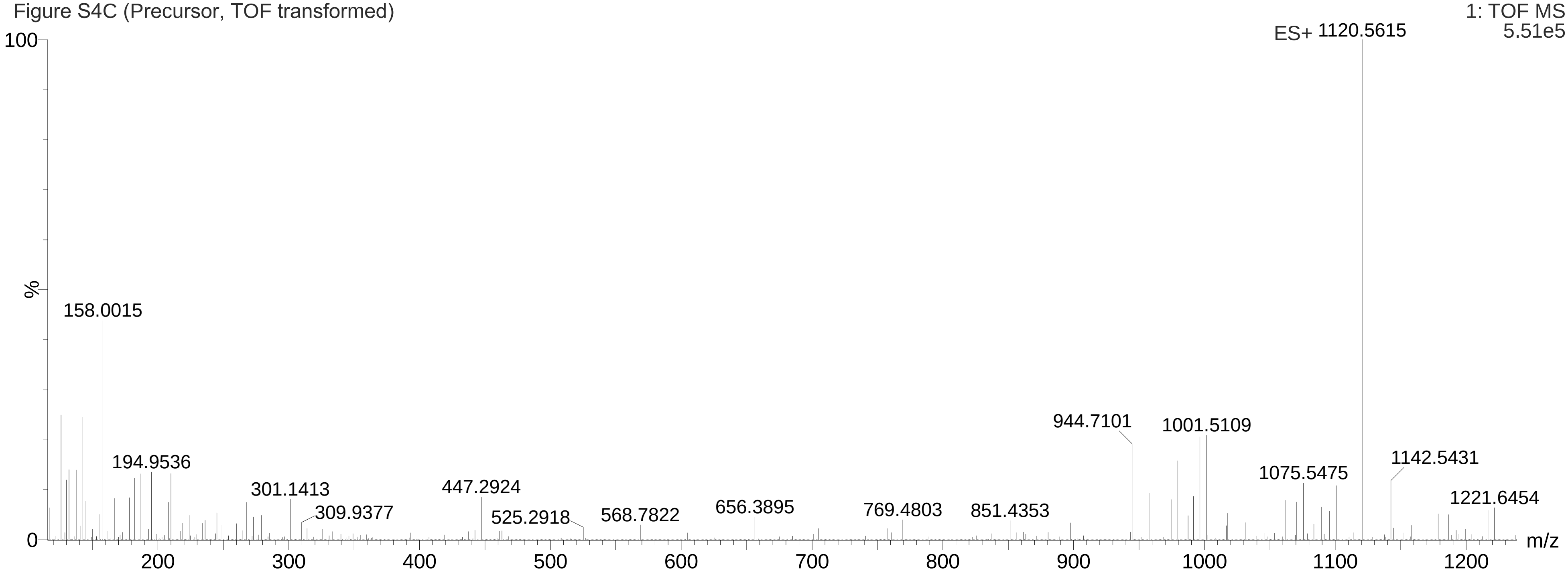

Figure S4C (Precursor, Lock mass corrected)

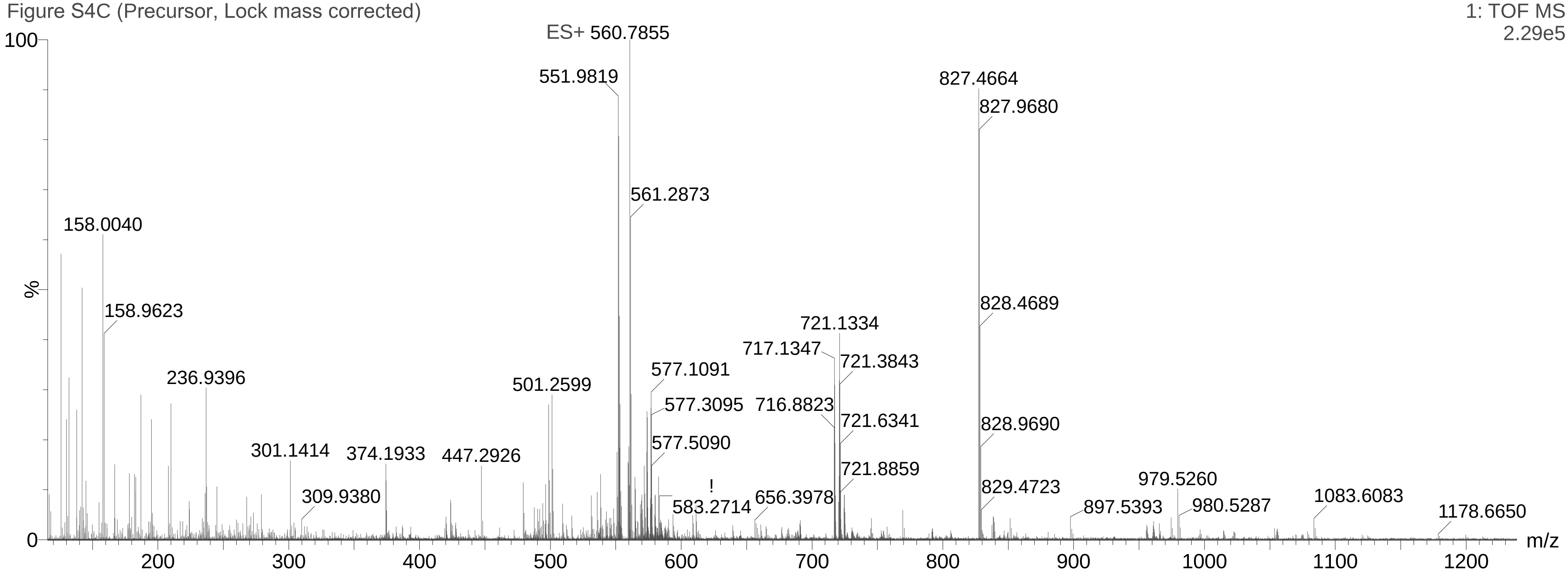

Figure S4C (TOF transformed)

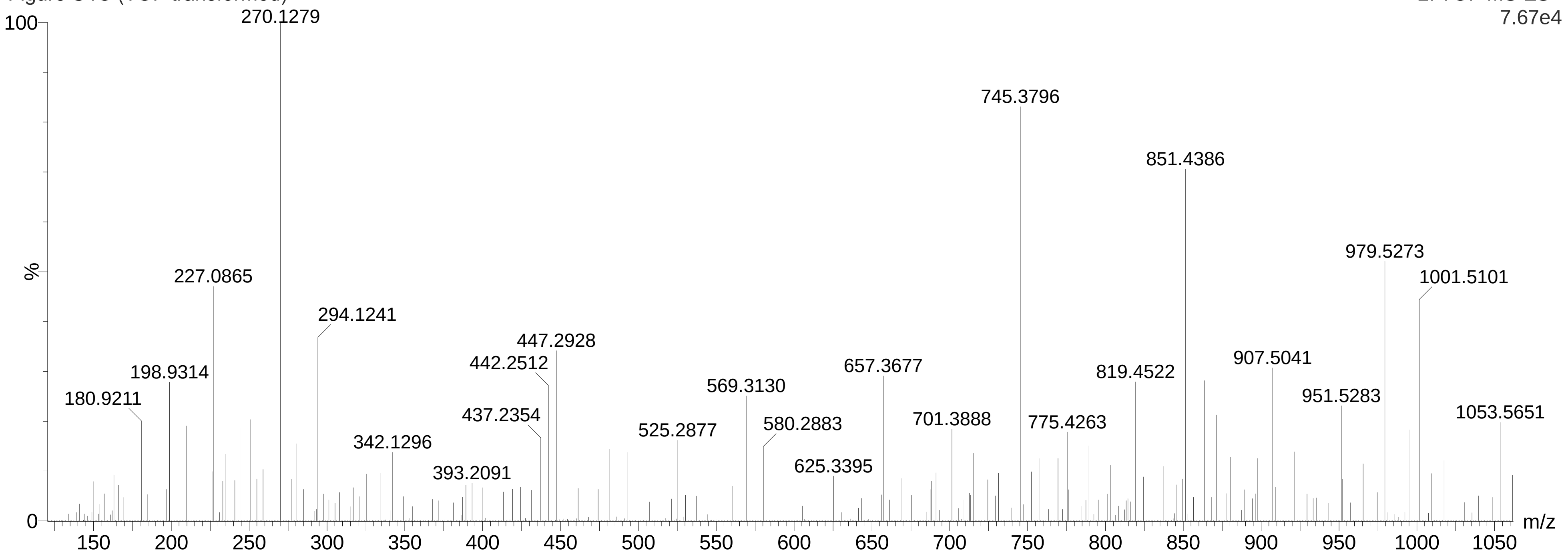

Figure S4C (Lock mass corrected)

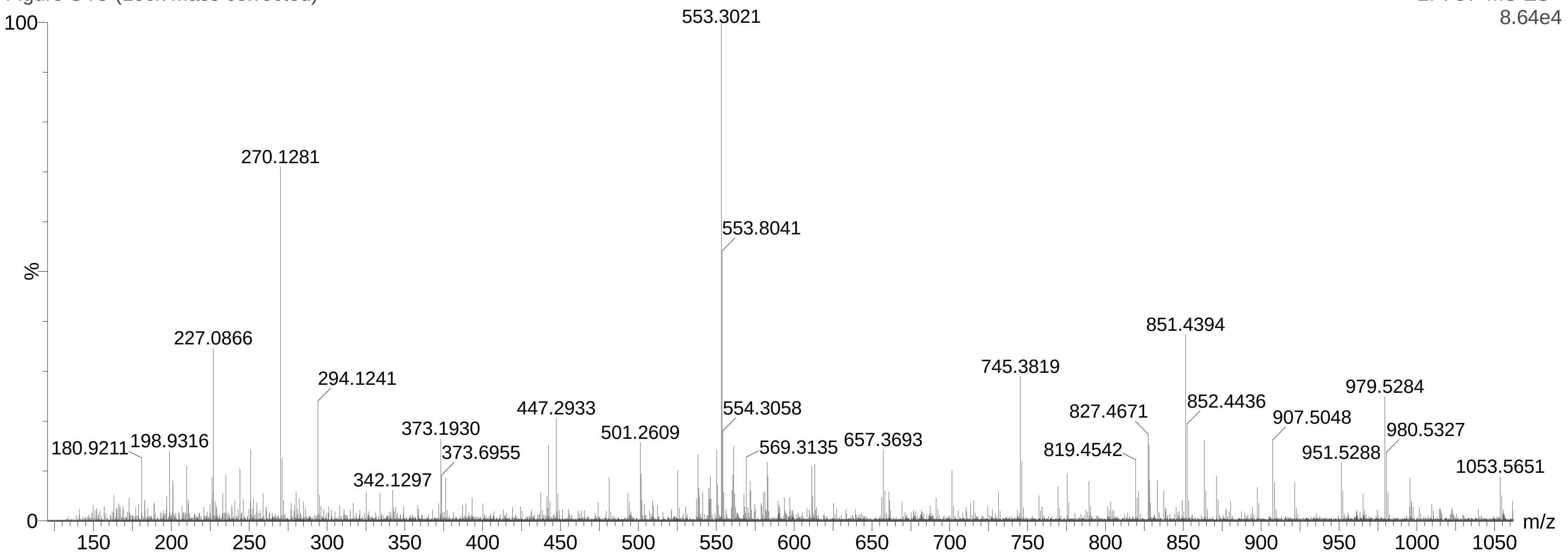

Figure S7A (TOF transformed)

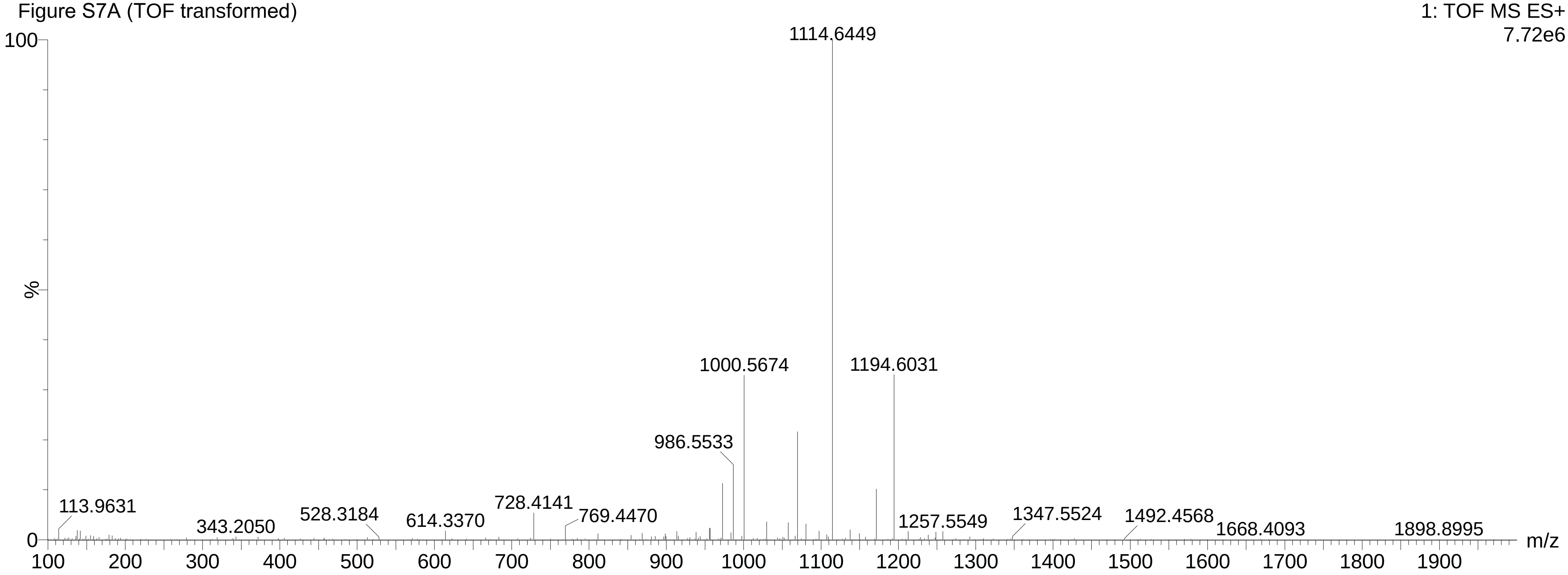

Figure S7A (Lock mass corrected)

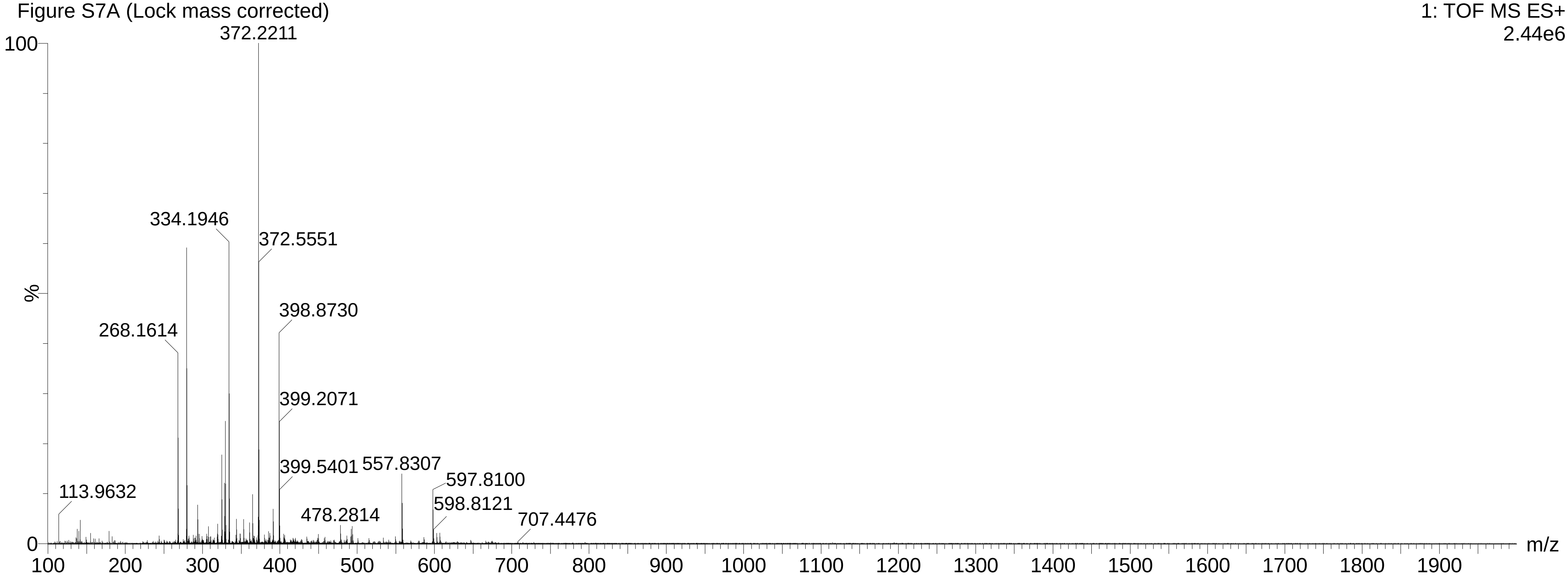

Figure S7B (TOF transformed)

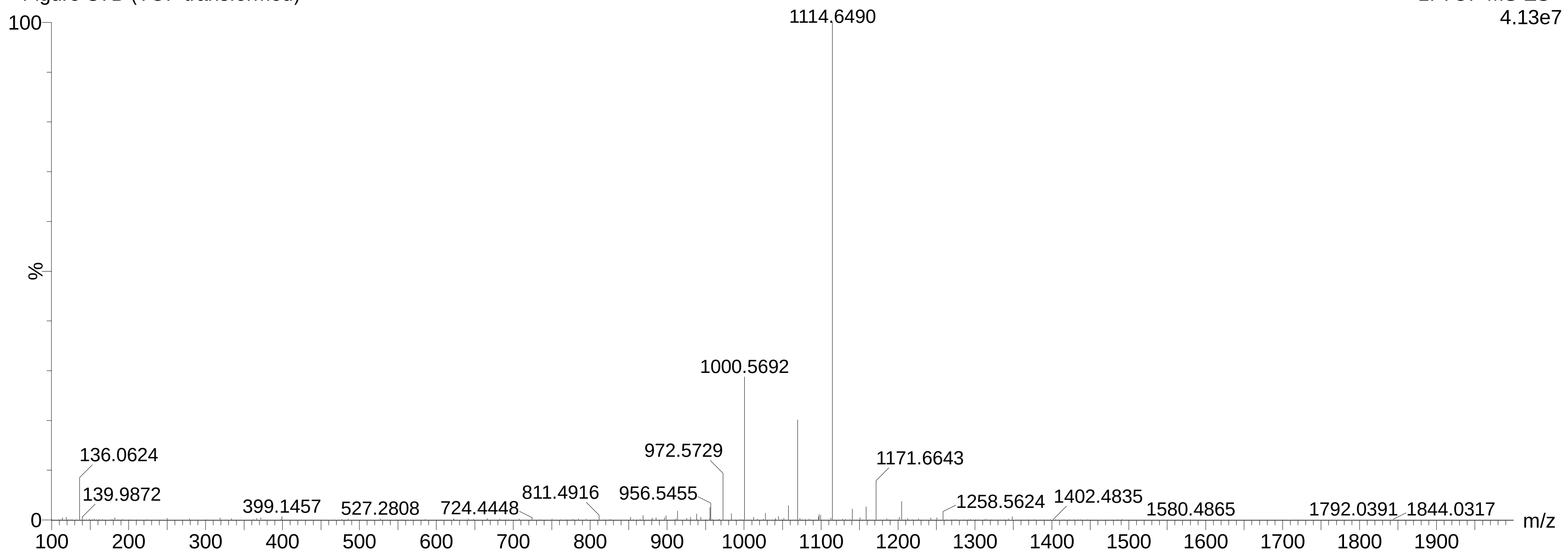

Figure S7B (Lock mass corrected)

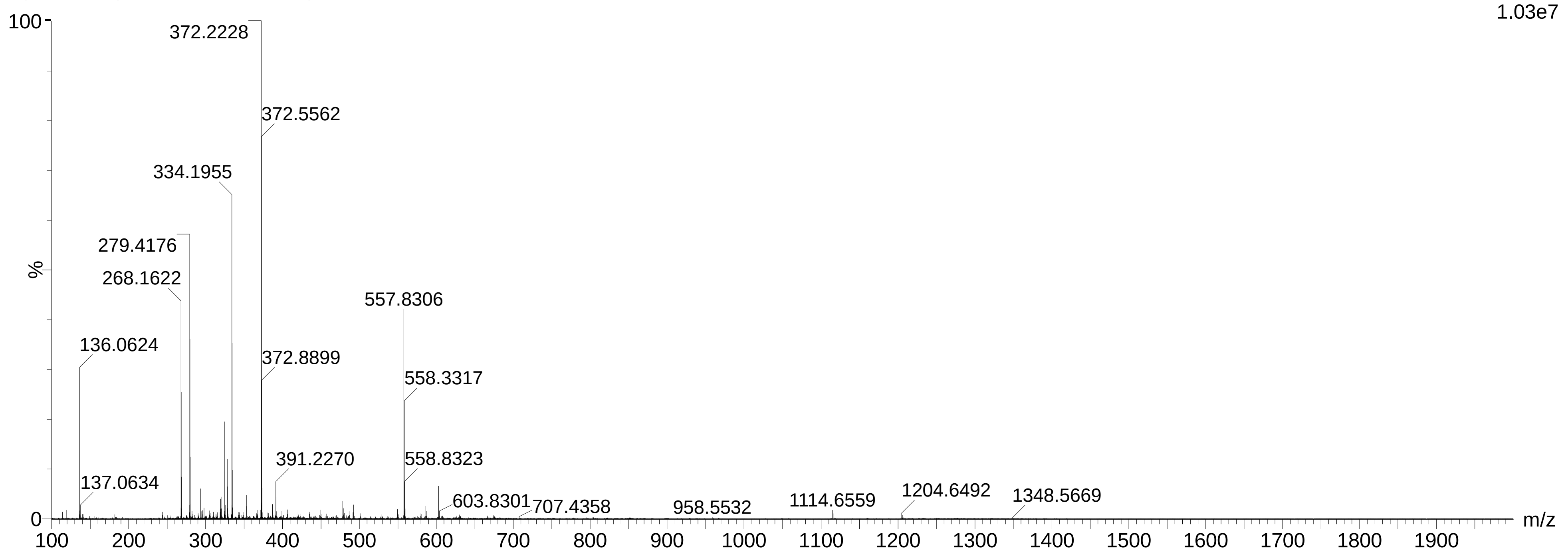

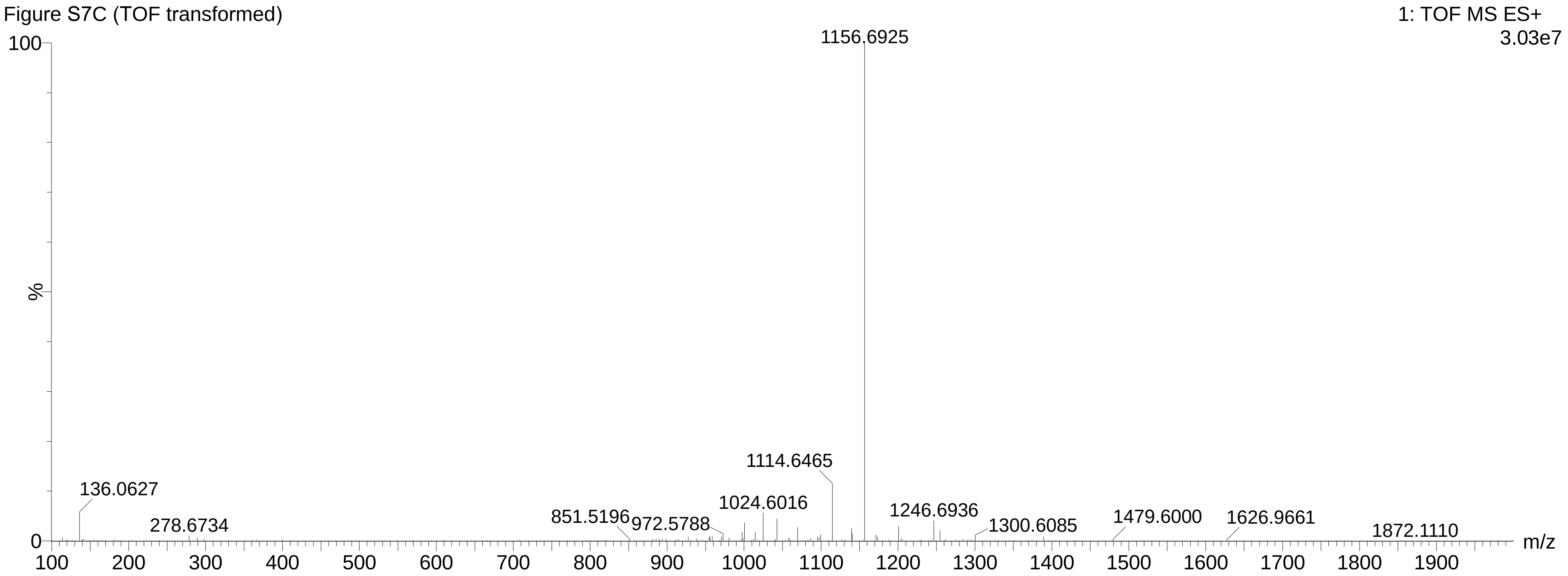
Figure S7C (TOF transformed)
1: TOF MS ES+
3.03e7
1156.6925
1114.6465
136.0627
1024.6016
1246.6936
851.5196
972.5788
278.6734
1300.6085
1479.6000
1626.9661
1872.1110
%
m/z

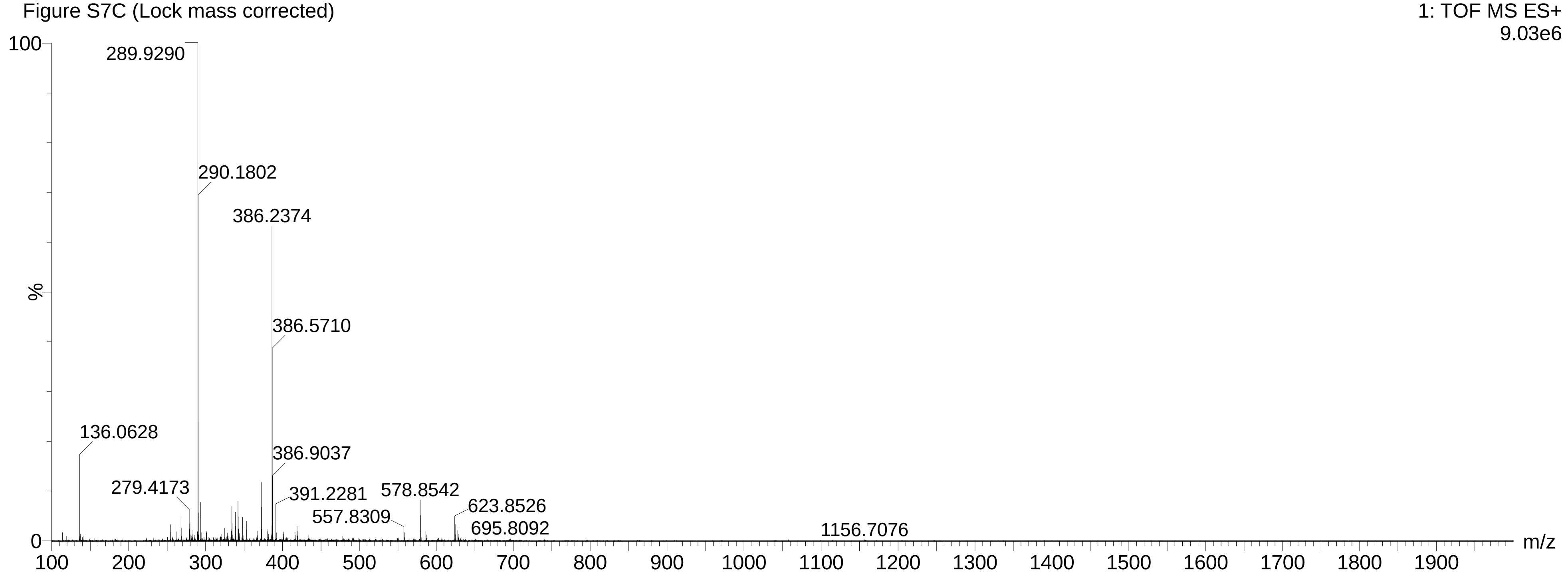
Figure S7C (Lock mass corrected)
1: TOF MS ES+
9.03e6
289.9290
290.1802
386.2374
386.5710
136.0628
386.9037
279.4173
391.2281
578.8542
623.8526
557.8309
695.8092
1156.7076
%
m/z

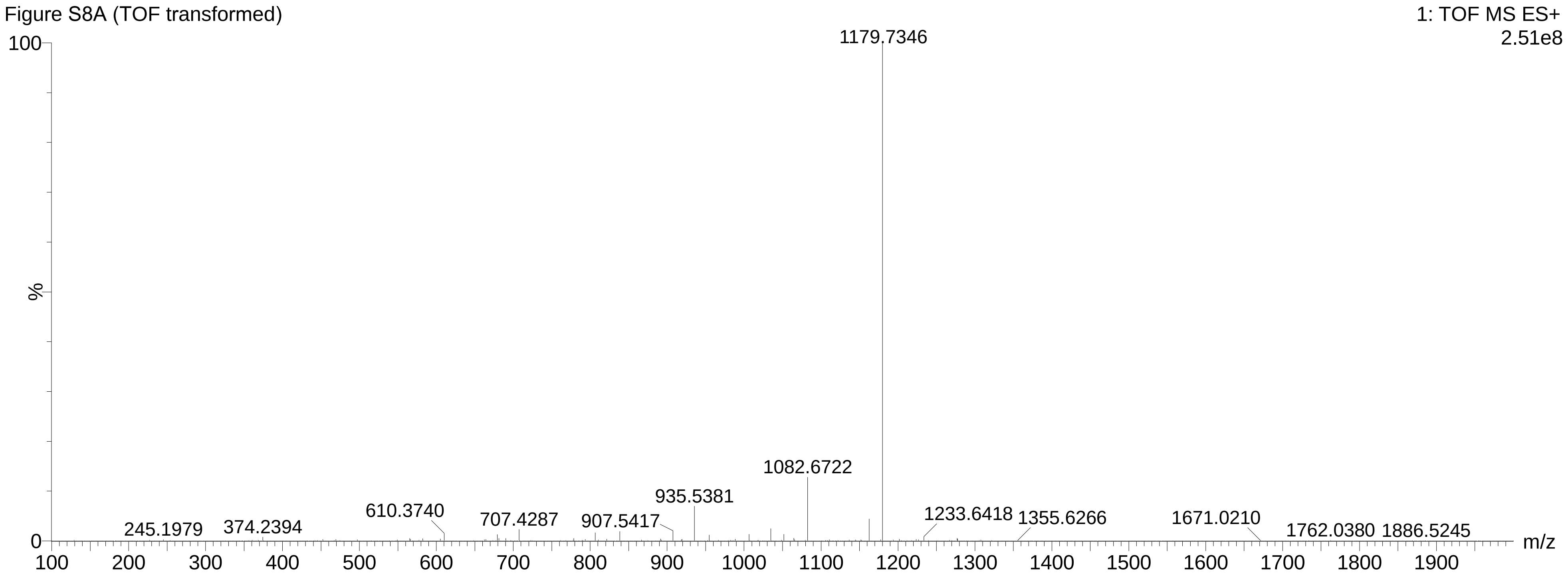

Figure S8A (TOF transformed)
1: TOF MS ES+
2.51e8
1179.7346
1082.6722
935.5381
907.5417
707.4287
610.3740
374.2394
245.1979
1233.6418
1355.6266
1671.0210
1762.0380
1886.5245
100
%
0
m/z
100 200 300 400 500 600 700 800 900 1000 1100 1200 1300 1400 1500 1600 1700 1800 1900

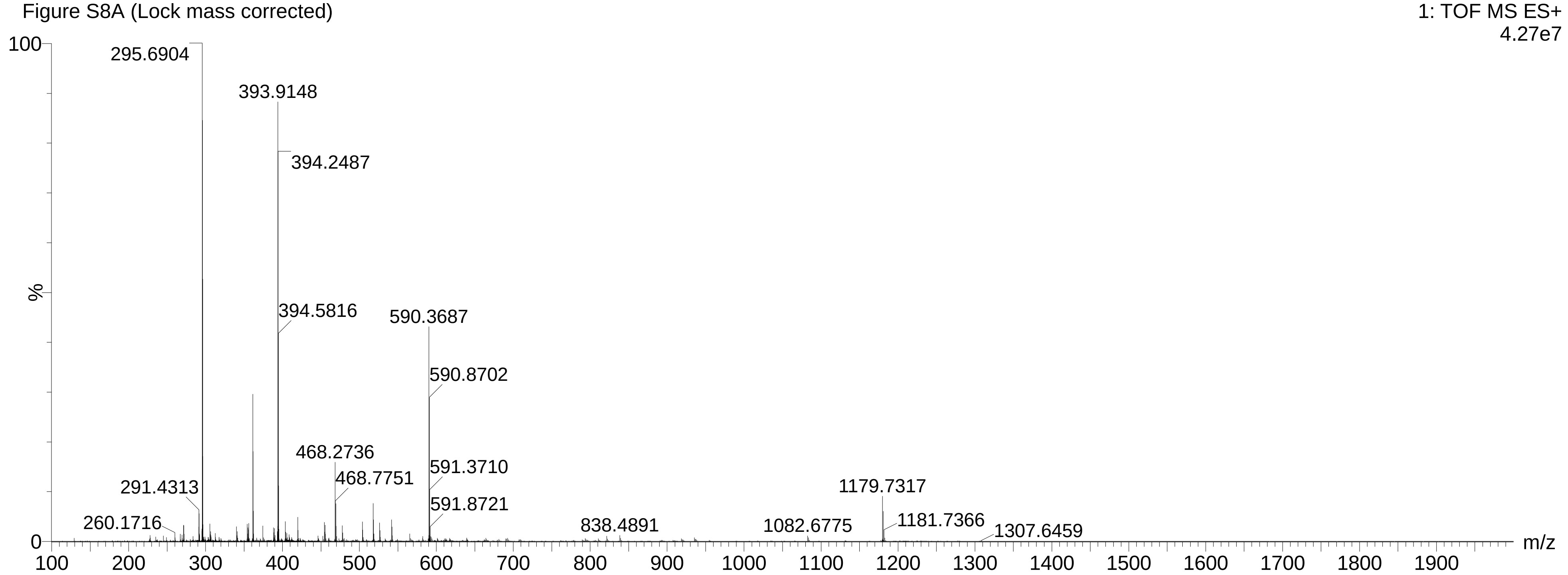

Figure S8A (Lock mass corrected)
1: TOF MS ES+
4.27e7
295.6904
393.9148
394.2487
394.5816
590.3687
590.8702
468.2736
468.7751
591.3710
591.8721
291.4313
260.1716
838.4891
1082.6775
1179.7317
1181.7366
1307.6459
100
%
0
m/z
100 200 300 400 500 600 700 800 900 1000 1100 1200 1300 1400 1500 1600 1700 1800 1900

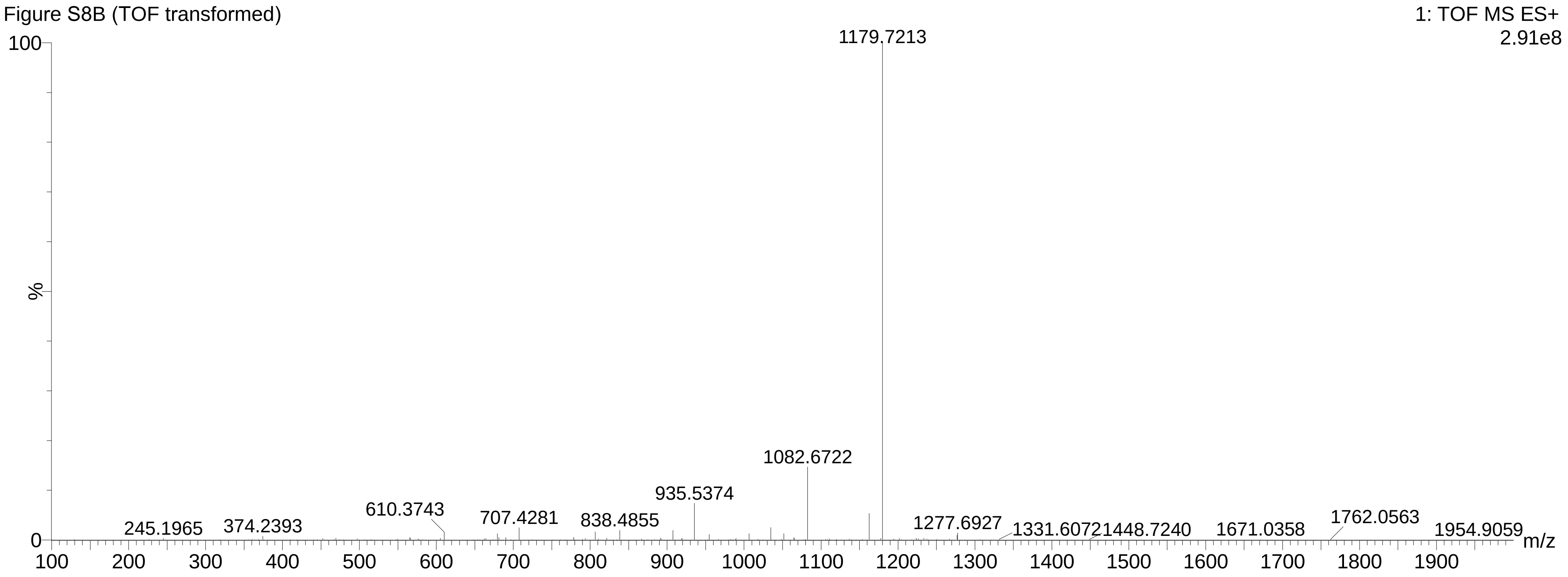

Figure S8B (TOF transformed)
1: TOF MS ES+
2.91e8
100
%
0
1179.7213
1082.6722
935.5374
610.3743
707.4281
838.4855
245.1965
374.2393
1277.6927
1331.6072
1448.7240
1671.0358
1762.0563
1954.9059
m/z
100 200 300 400 500 600 700 800 900 1000 1100 1200 1300 1400 1500 1600 1700 1800 1900

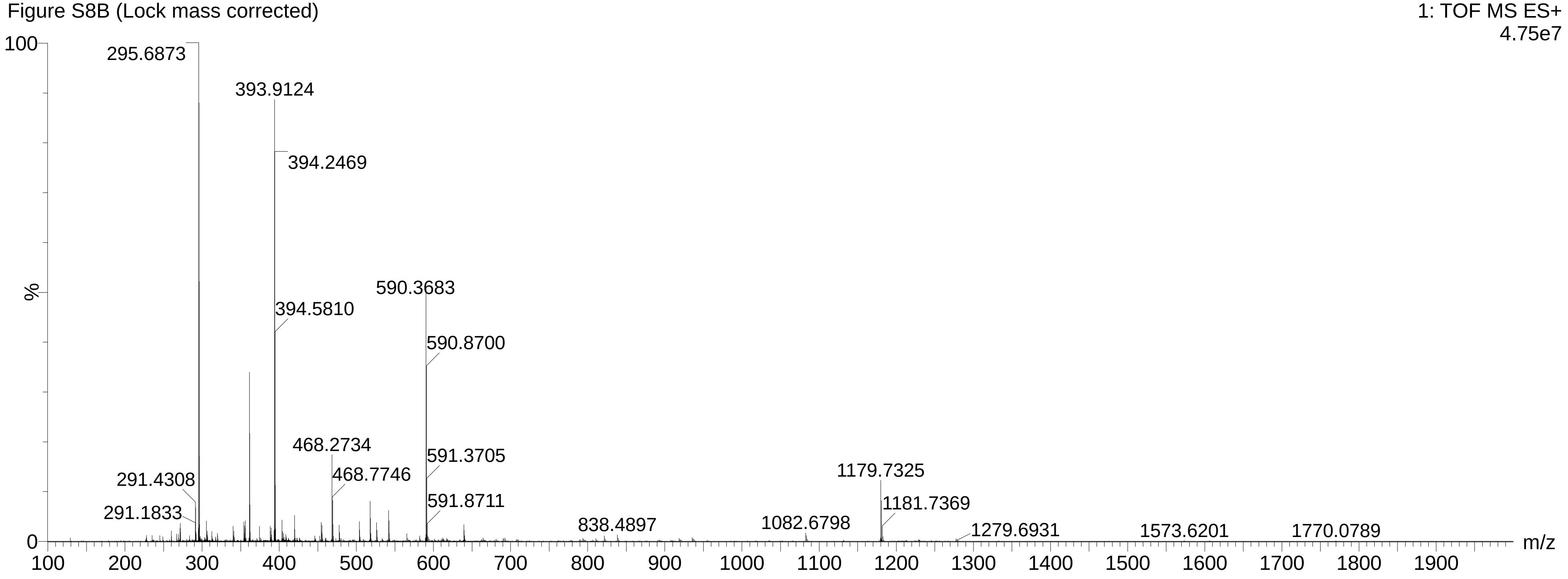

Figure S8B (Lock mass corrected)
1: TOF MS ES+
4.75e7
100
%
0
295.6873
393.9124
394.2469
394.5810
590.3683
590.8700
468.2734
468.7746
591.3705
591.8711
291.4308
291.1833
838.4897
1082.6798
1179.7325
1181.7369
1279.6931
1573.6201
1770.0789
m/z
100 200 300 400 500 600 700 800 900 1000 1100 1200 1300 1400 1500 1600 1700 1800 1900

Figure S8C (TOF transformed)

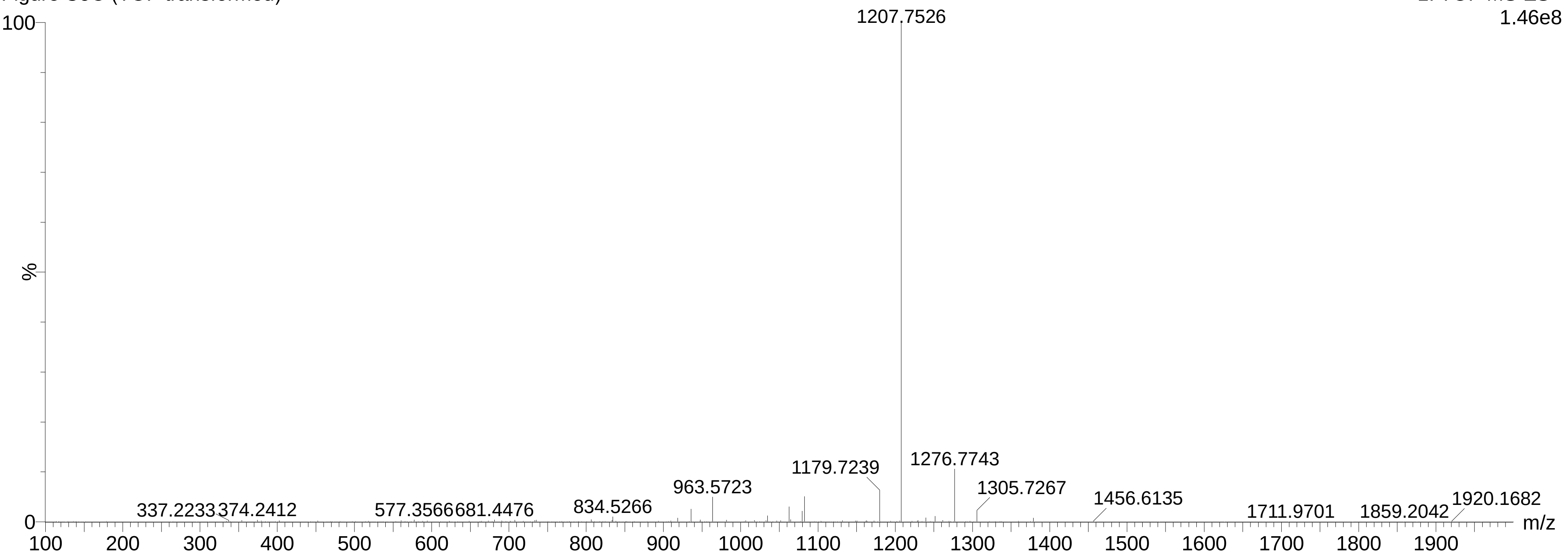

Figure S8C (Lock mass corrected)

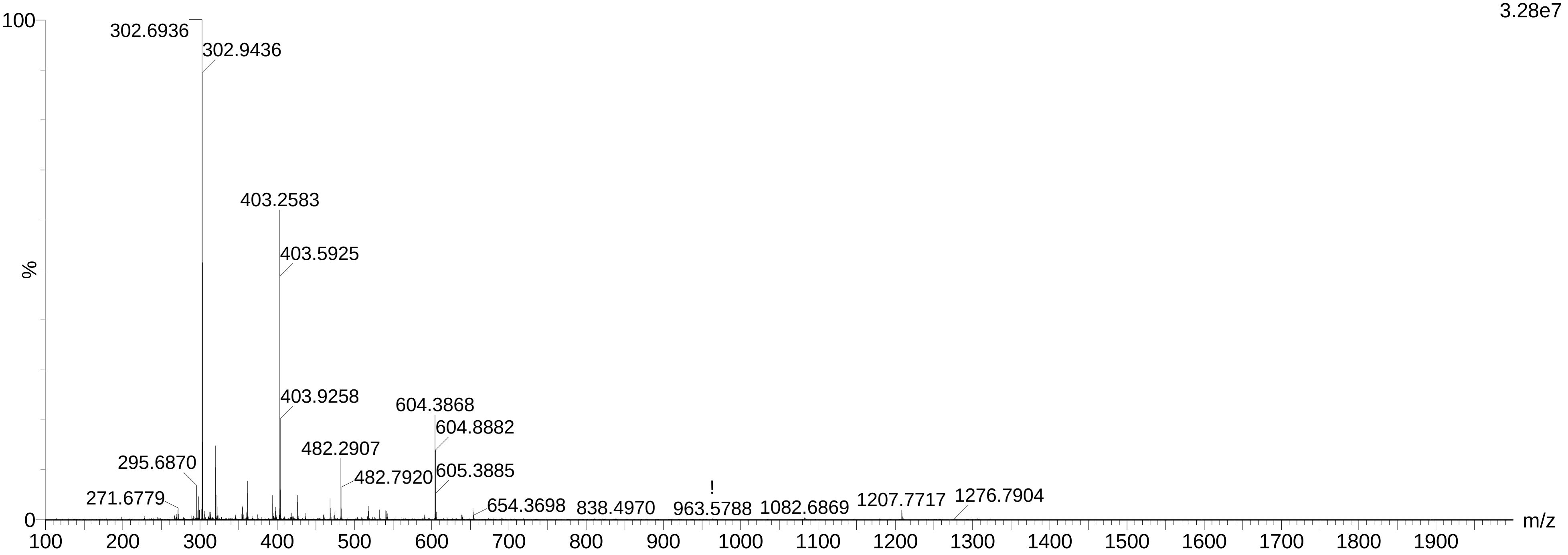

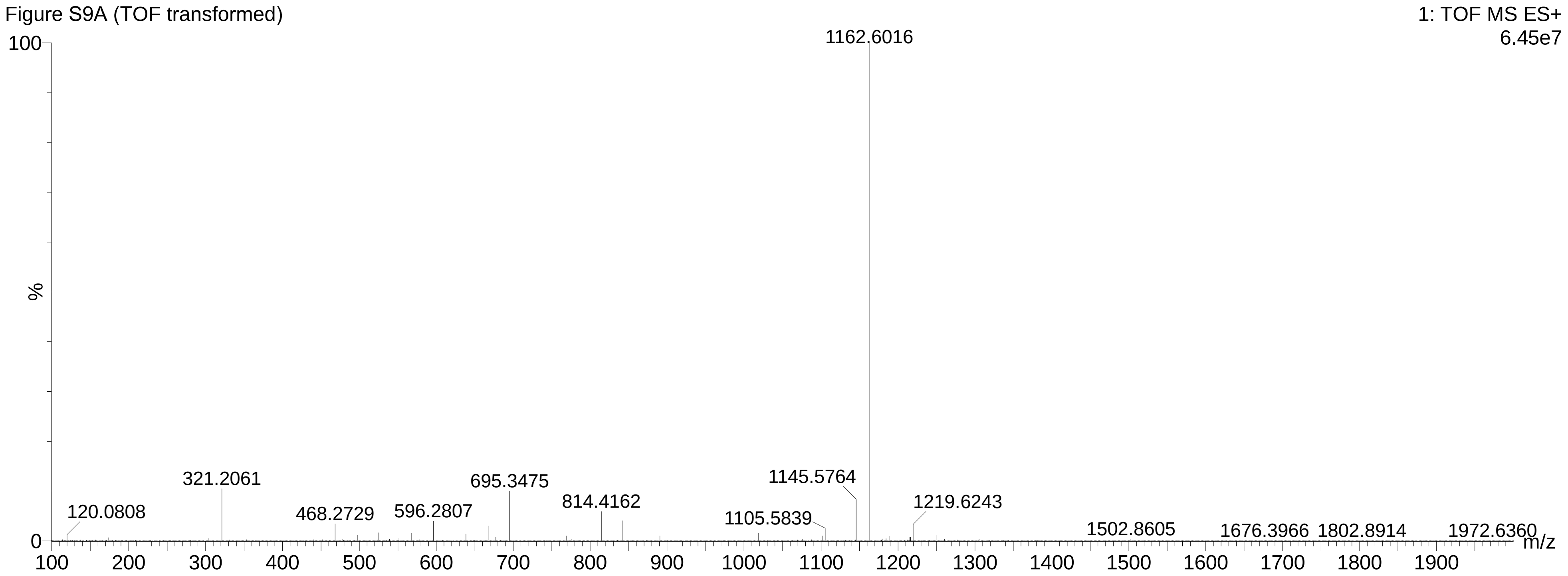
Figure S9A (TOF transformed)
1: TOF MS ES+
6.45e7
1162.6016
321.2061
695.3475
1145.5764
120.0808
468.2729
596.2807
814.4162
1219.6243
1105.5839
1502.8605
1676.3966
1802.8914
1972.6360
%
m/z

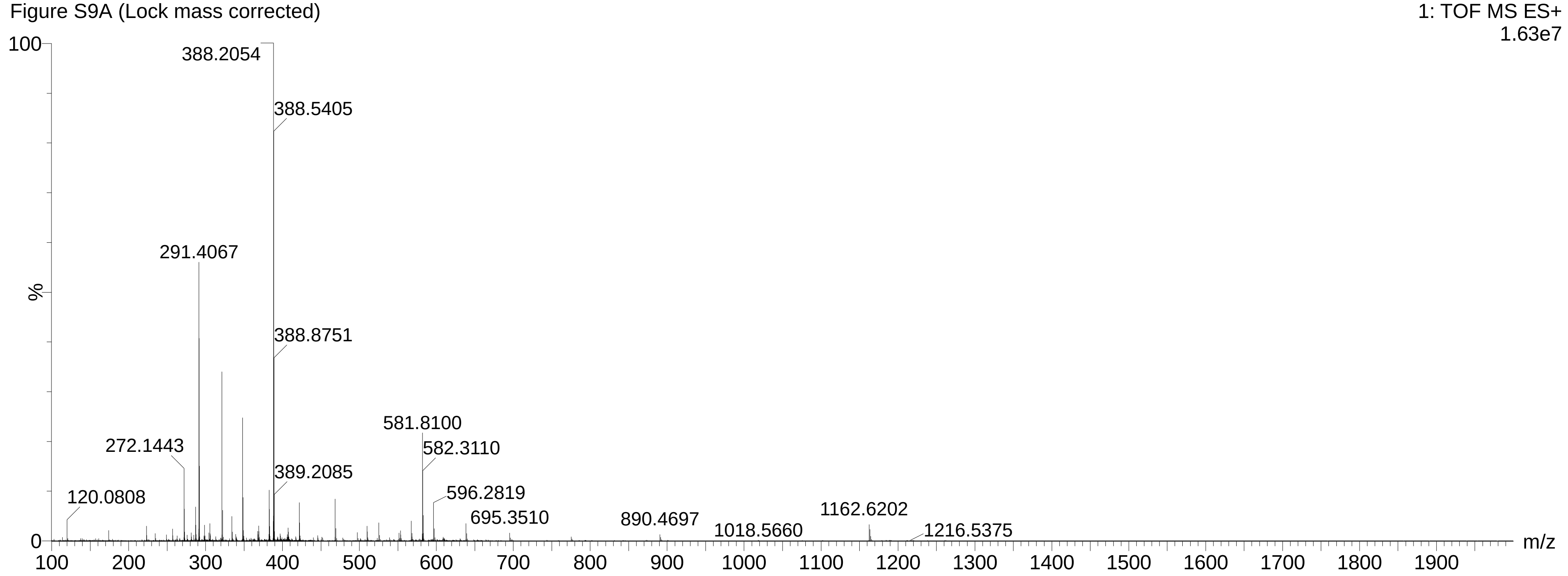
Figure S9A (Lock mass corrected)
1: TOF MS ES+
1.63e7
388.2054
388.5405
291.4067
388.8751
581.8100
582.3110
272.1443
389.2085
120.0808
596.2819
695.3510
890.4697
1162.6202
1018.5660
1216.5375
%
m/z

Figure S9B (TOF transformed)

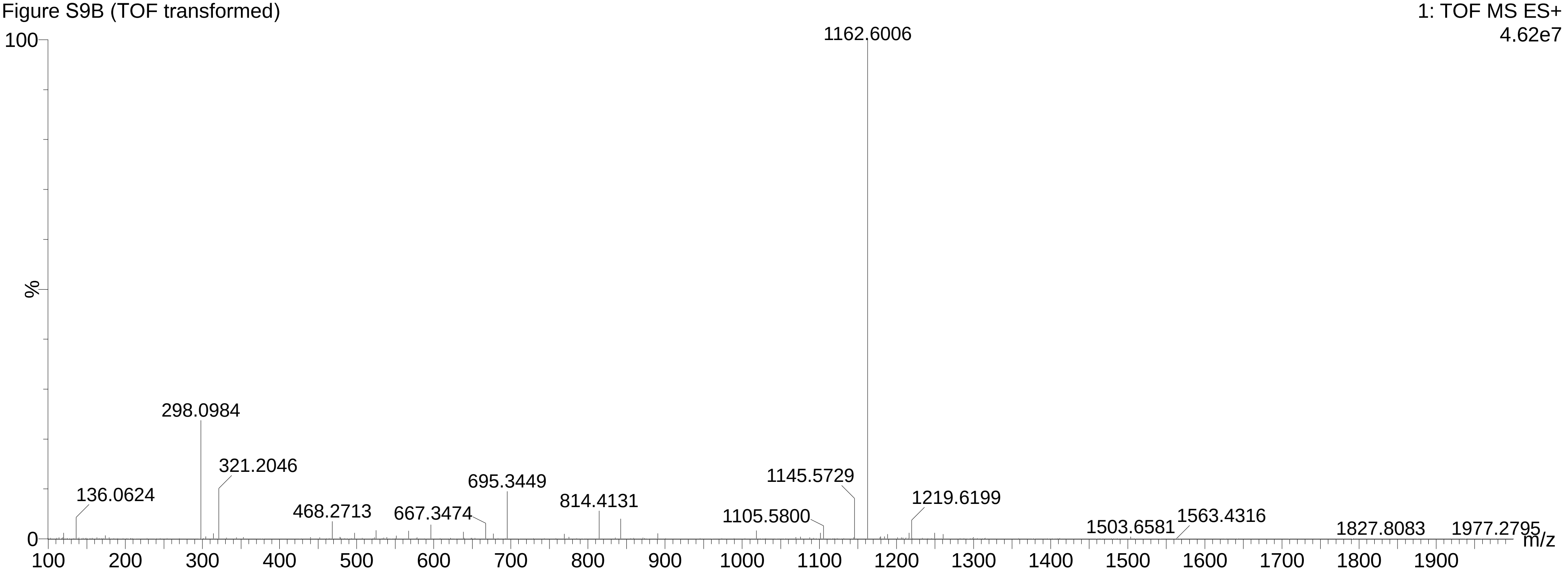

Figure S9B (Lock mass corrected)

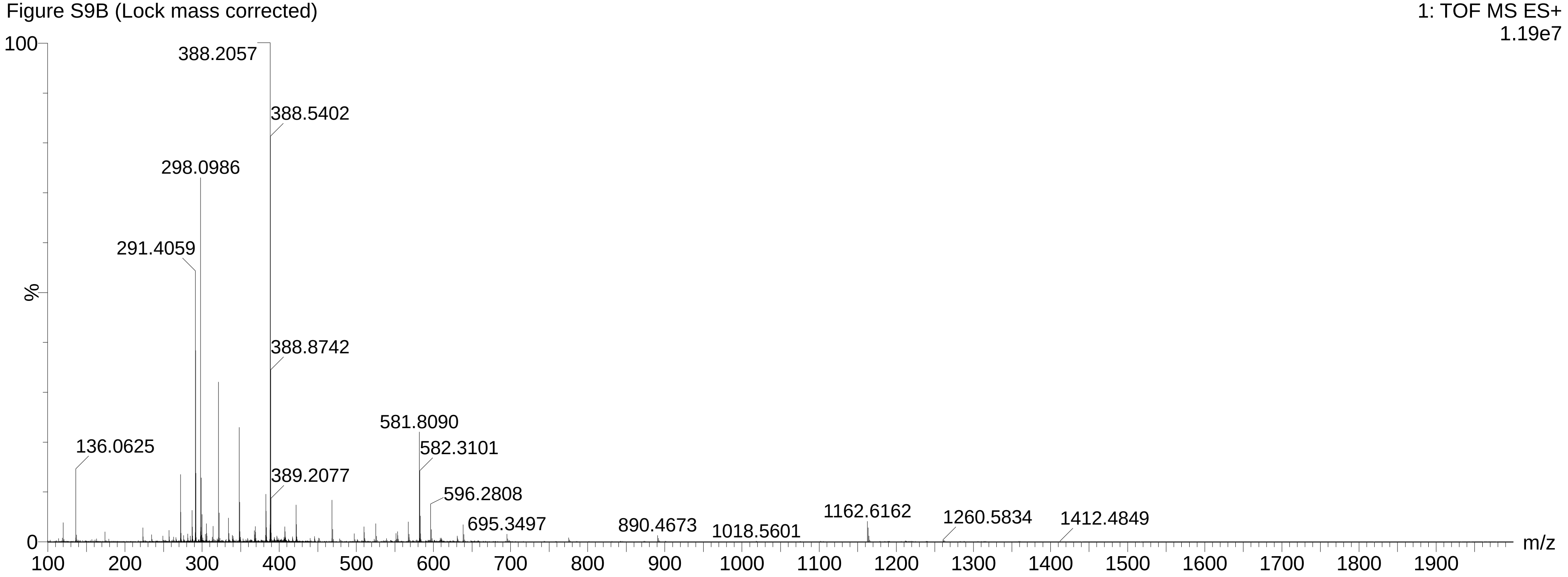

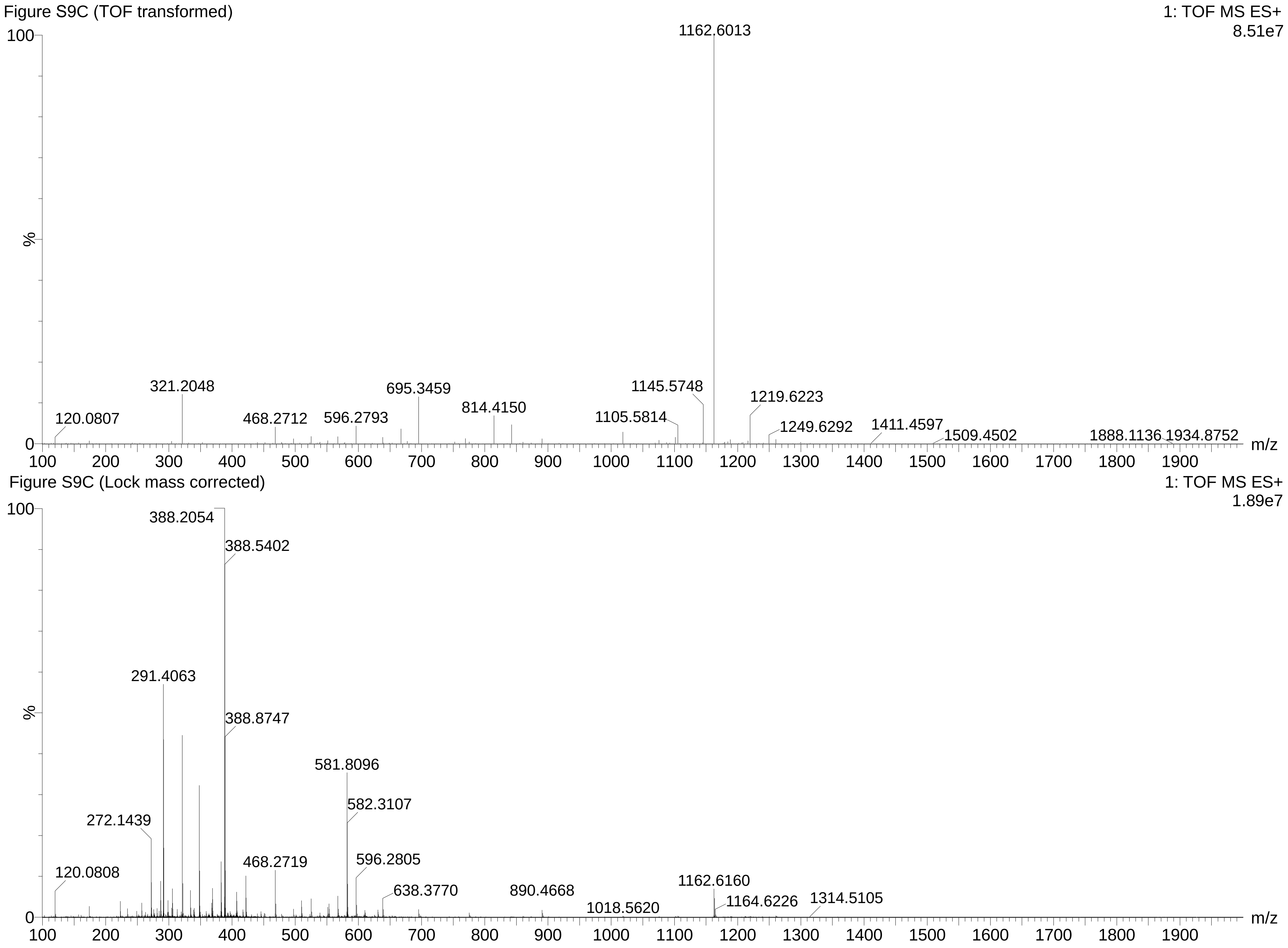

Figure S9C (TOF transformed)
1: TOF MS ES+
8.51e7
100
%
0
1162.6013
321.2048
695.3459
1145.5748
1219.6223
120.0807
468.2712
596.2793
814.4150
1105.5814
1249.6292
1411.4597
1509.4502
1888.1136
1934.8752
m/z
100
200
300
400
500
600
700
800
900
1000
1100
1200
1300
1400
1500
1600
1700
1800
1900
Figure S9C (Lock mass corrected)
1: TOF MS ES+
1.89e7
388.2054
388.5402
291.4063
388.8747
581.8096
582.3107
272.1439
468.2719
596.2805
120.0808
1162.6160
638.3770
890.4668
1164.6226
1314.5105
1018.5620

Figure S10A (TOF transformed)

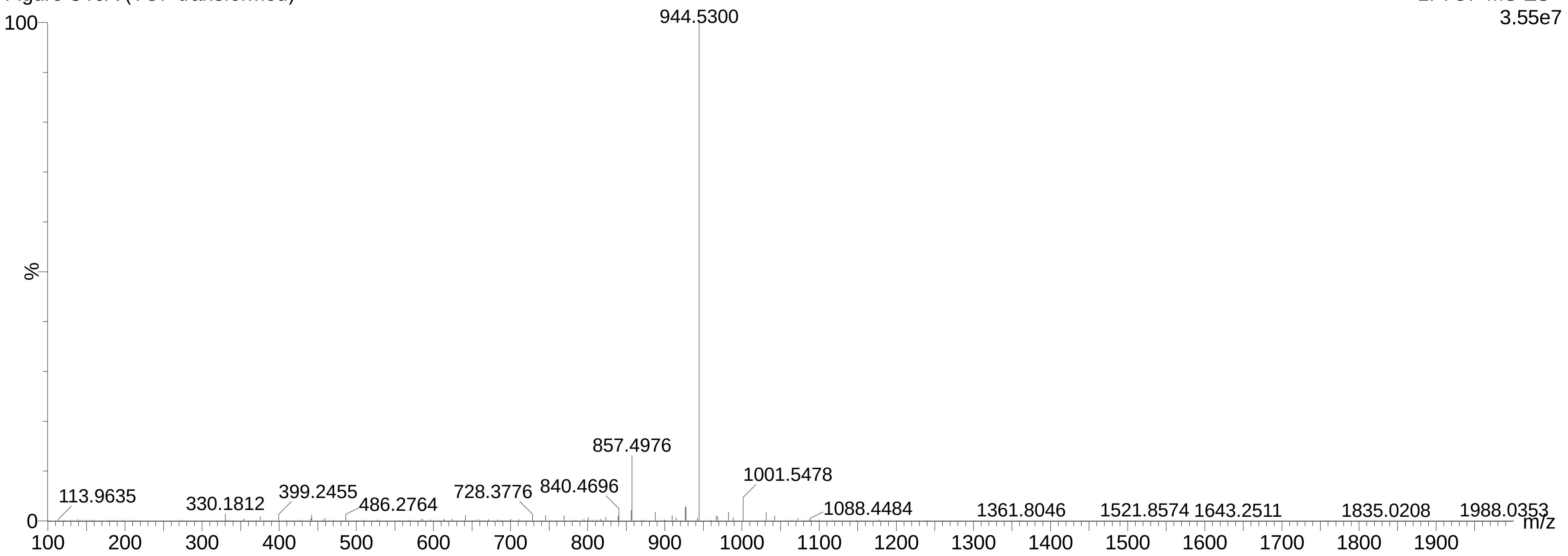

Figure S10A (Lock mass corrected)

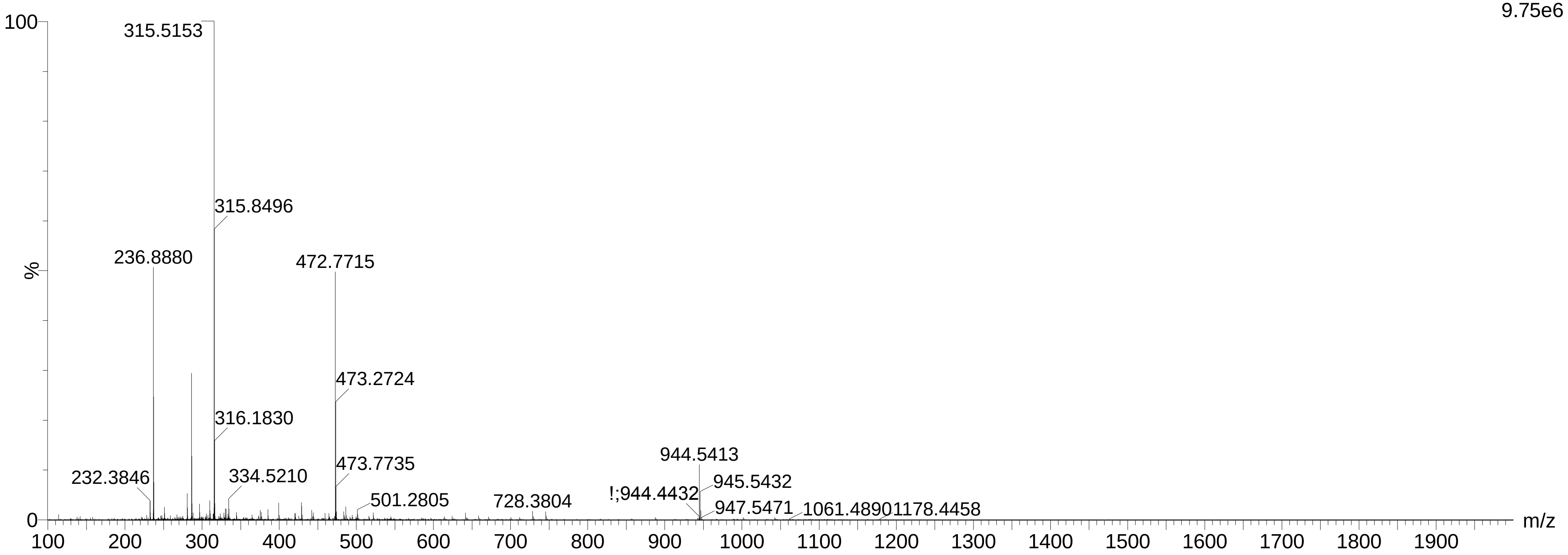

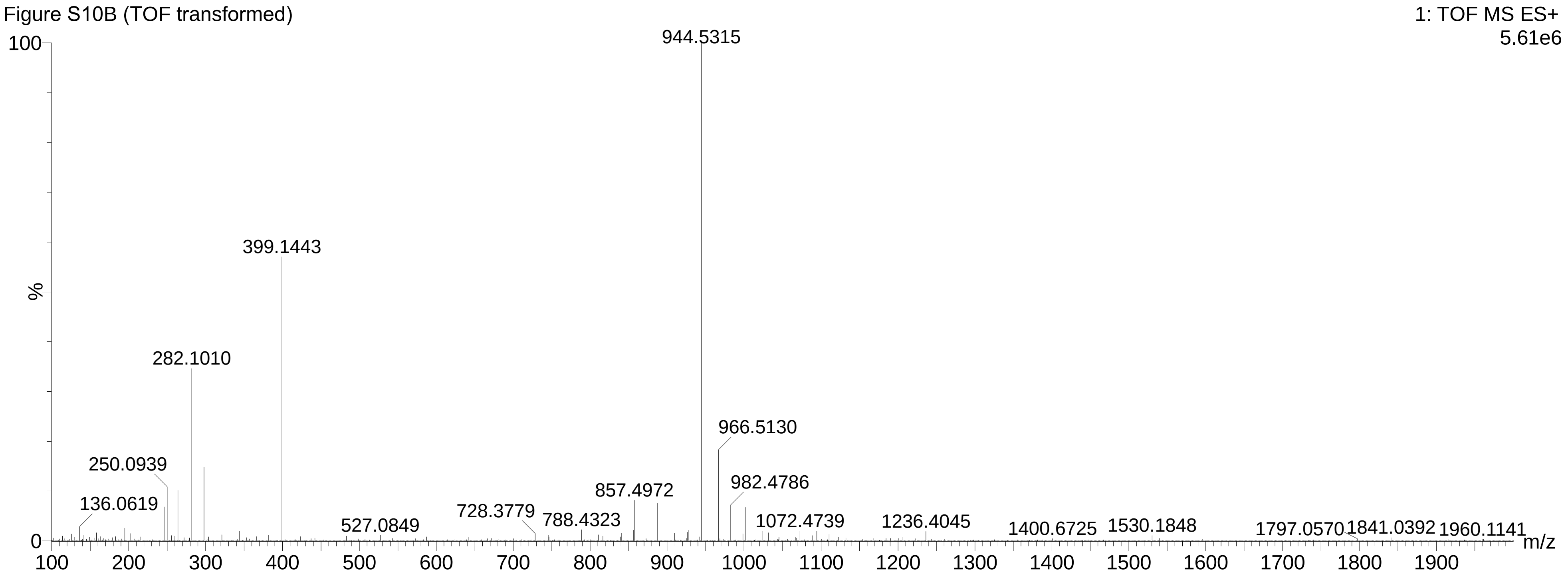
Figure S10B (TOF transformed)
1: TOF MS ES+
5.61e6
944.5315
399.1443
282.1010
966.5130
250.0939
982.4786
857.4972
136.0619
728.3779
788.4323
1072.4739
1236.4045
527.0849
1400.6725
1530.1848
1797.0570
1841.0392
1960.1141
100
%
0
m/z
100 200 300 400 500 600 700 800 900 1000 1100 1200 1300 1400 1500 1600 1700 1800 1900

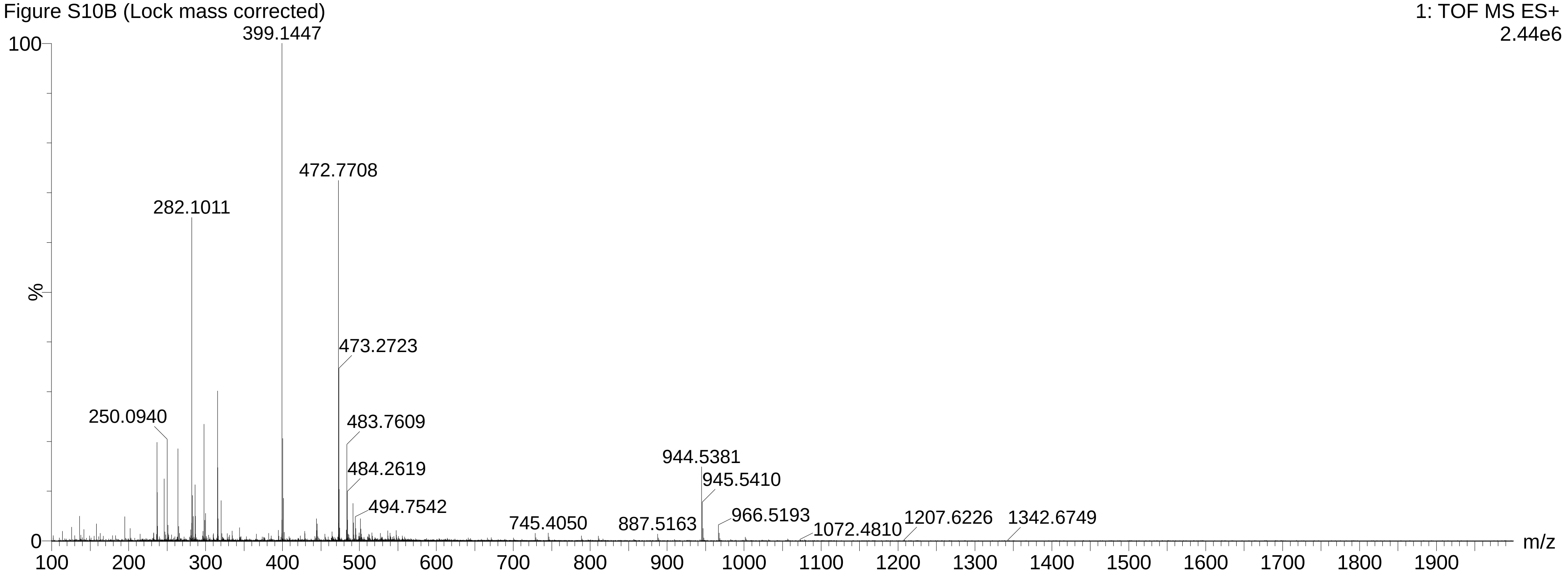
Figure S10B (Lock mass corrected)
1: TOF MS ES+
2.44e6
399.1447
472.7708
282.1011
473.2723
250.0940
483.7609
484.2619
494.7542
944.5381
945.5410
966.5193
745.4050
887.5163
1072.4810
1207.6226
1342.6749
100
%
0
m/z
100 200 300 400 500 600 700 800 900 1000 1100 1200 1300 1400 1500 1600 1700 1800 1900

Figure S10C (TOF transformed)

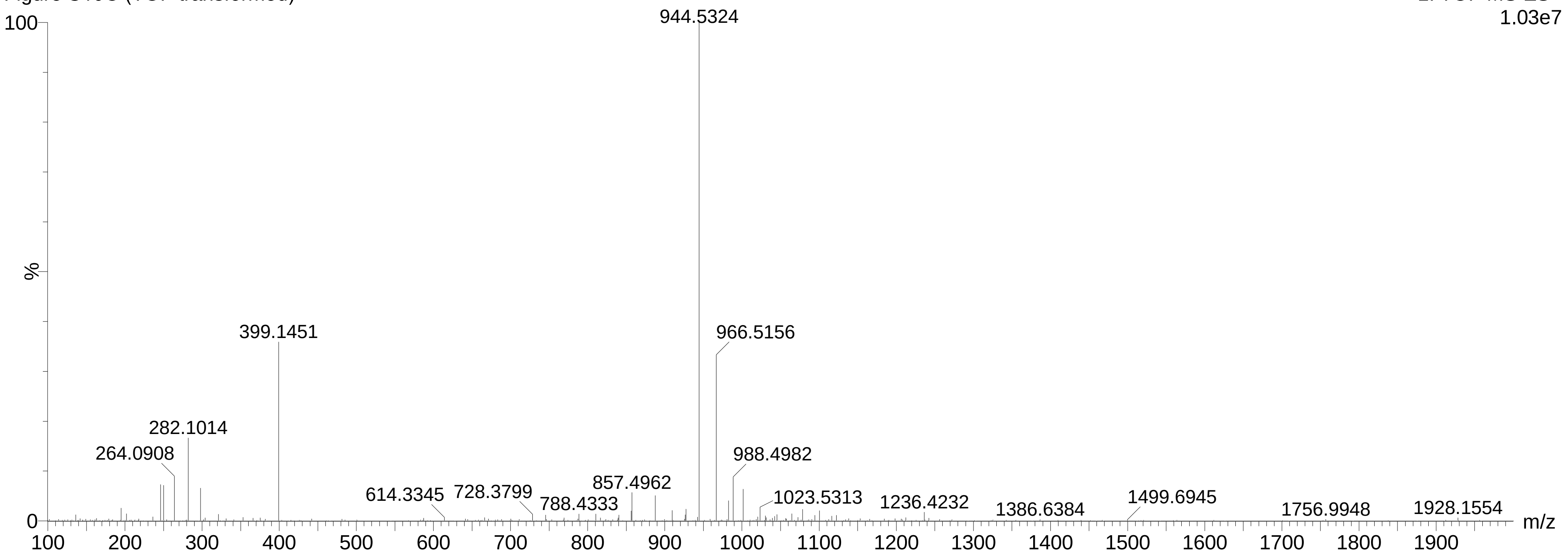

Figure S10C (Lock mass corrected)

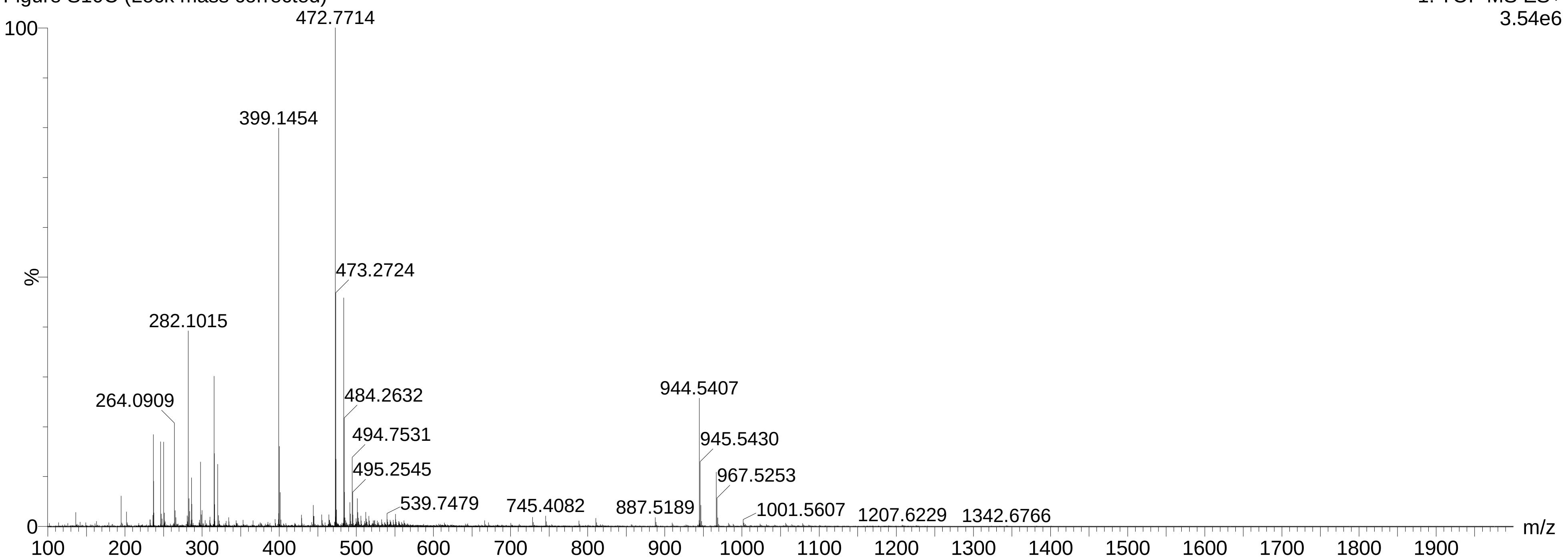

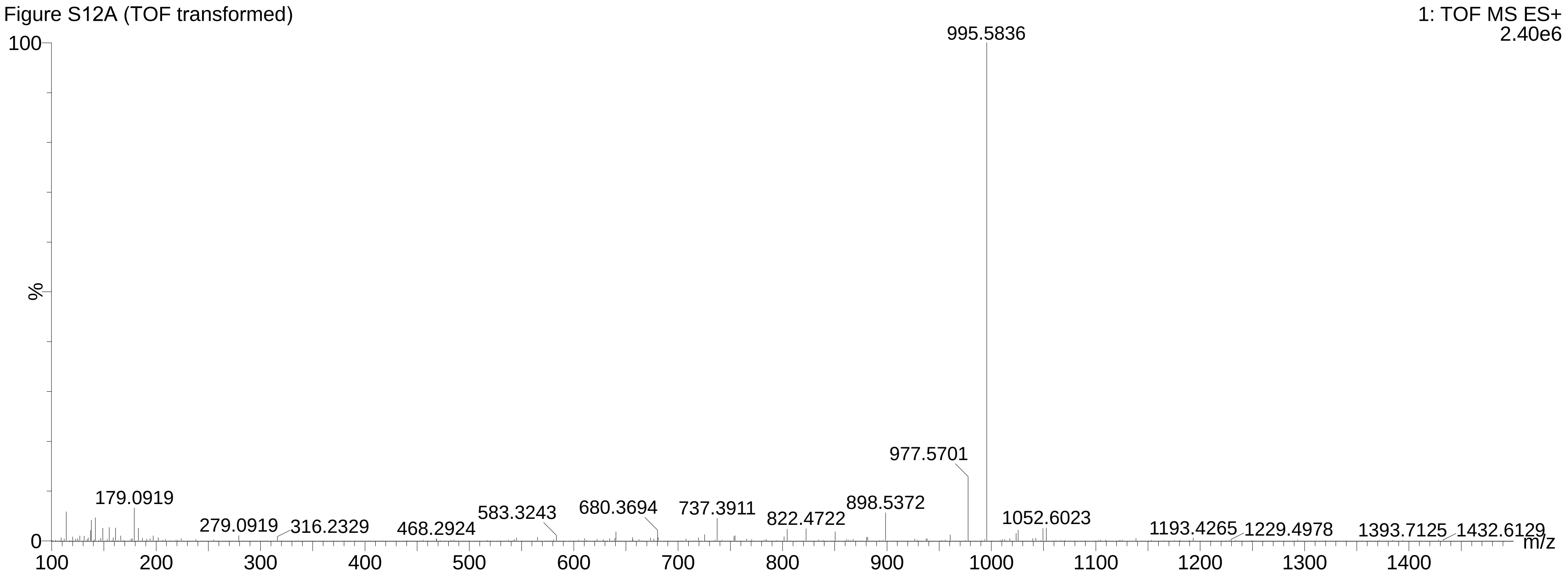
Figure S12A (TOF transformed)
1: TOF MS ES+
2.40e6
995.5836
977.5701
179.0919
279.0919
316.2329
468.2924
583.3243
680.3694
737.3911
822.4722
898.5372
1052.6023
1193.4265
1229.4978
1393.7125
1432.6129
100
%
0
m/z
100
200
300
400
500
600
700
800
900
1000
1100
1200
1300
1400

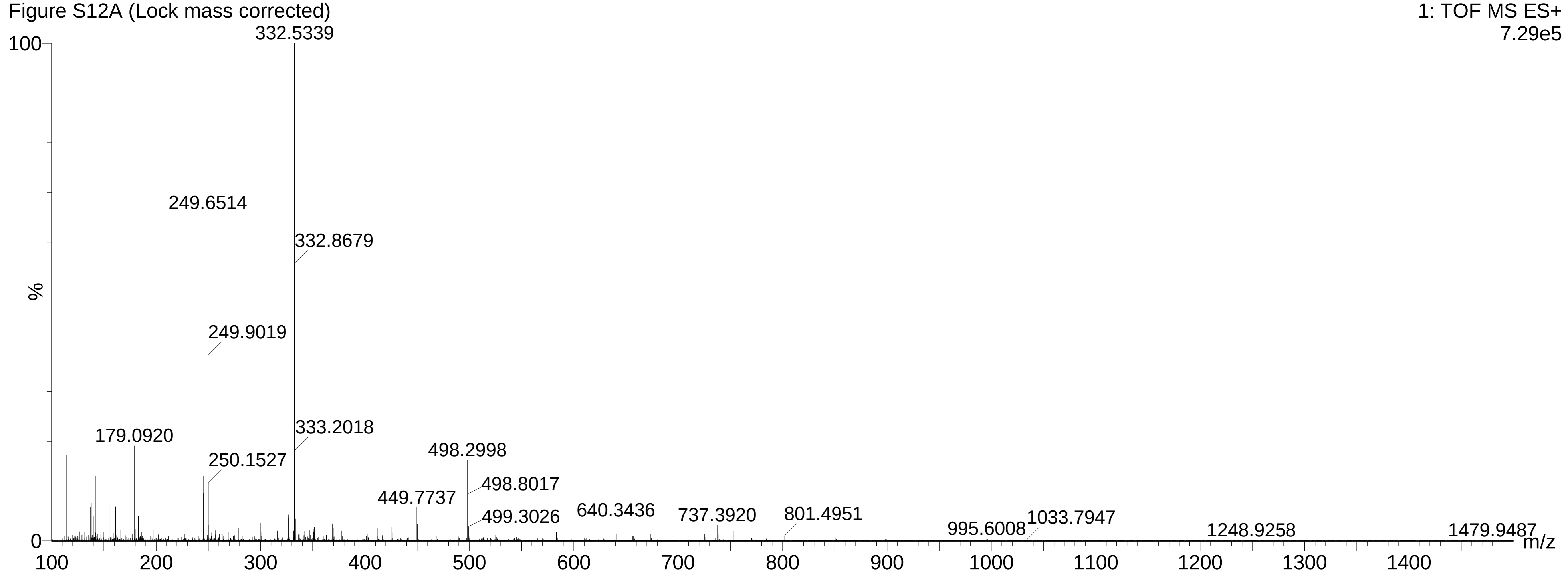
Figure S12A (Lock mass corrected)
1: TOF MS ES+
7.29e5
332.5339
249.6514
332.8679
249.9019
179.0920
333.2018
250.1527
498.2998
498.8017
449.7737
499.3026
640.3436
737.3920
801.4951
995.6008
1033.7947
1248.9258
1479.9487
100
%
0
m/z
100
200
300
400
500
600
700
800
900
1000
1100
1200
1300
1400

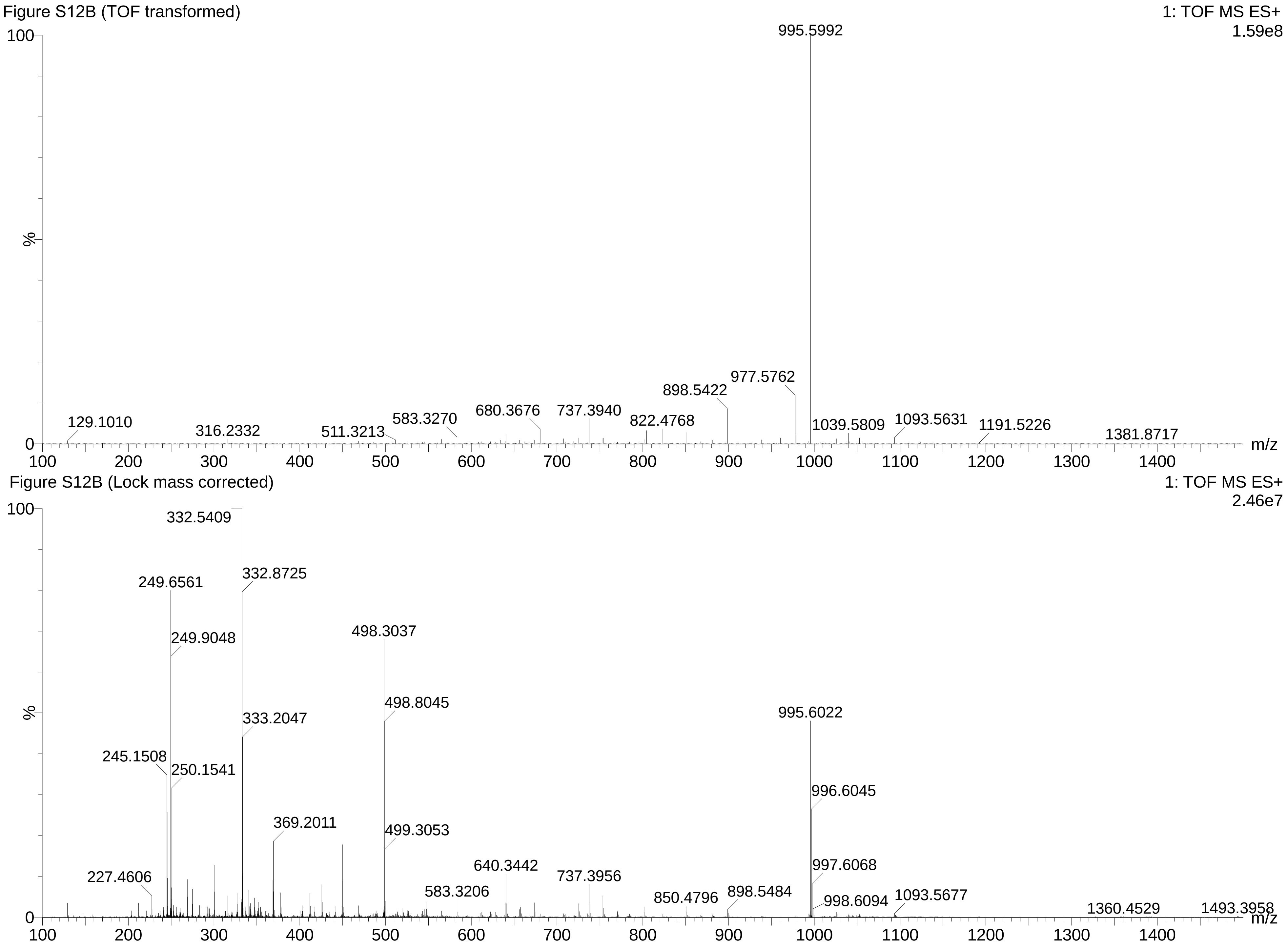
Figure S12B (TOF transformed)
1: TOF MS ES+
1.59e8
100
%
0
995.5992
977.5762
898.5422
129.1010
316.2332
511.3213
583.3270
680.3676
737.3940
822.4768
1039.5809
1093.5631
1191.5226
1381.8717
m/z
100 200 300 400 500 600 700 800 900 1000 1100 1200 1300 1400
Figure S12B (Lock mass corrected)
1: TOF MS ES+
2.46e7
100
%
0
332.5409
332.8725
249.6561
249.9048
498.3037
498.8045
333.2047
995.6022
245.1508
250.1541
996.6045
369.2011
499.3053
640.3442
997.6068
227.4606
737.3956
583.3206
850.4796
898.5484
998.6094
1093.5677
1360.4529
1493.3958
m/z
100 200 300 400 500 600 700 800 900 1000 1100 1200 1300 1400

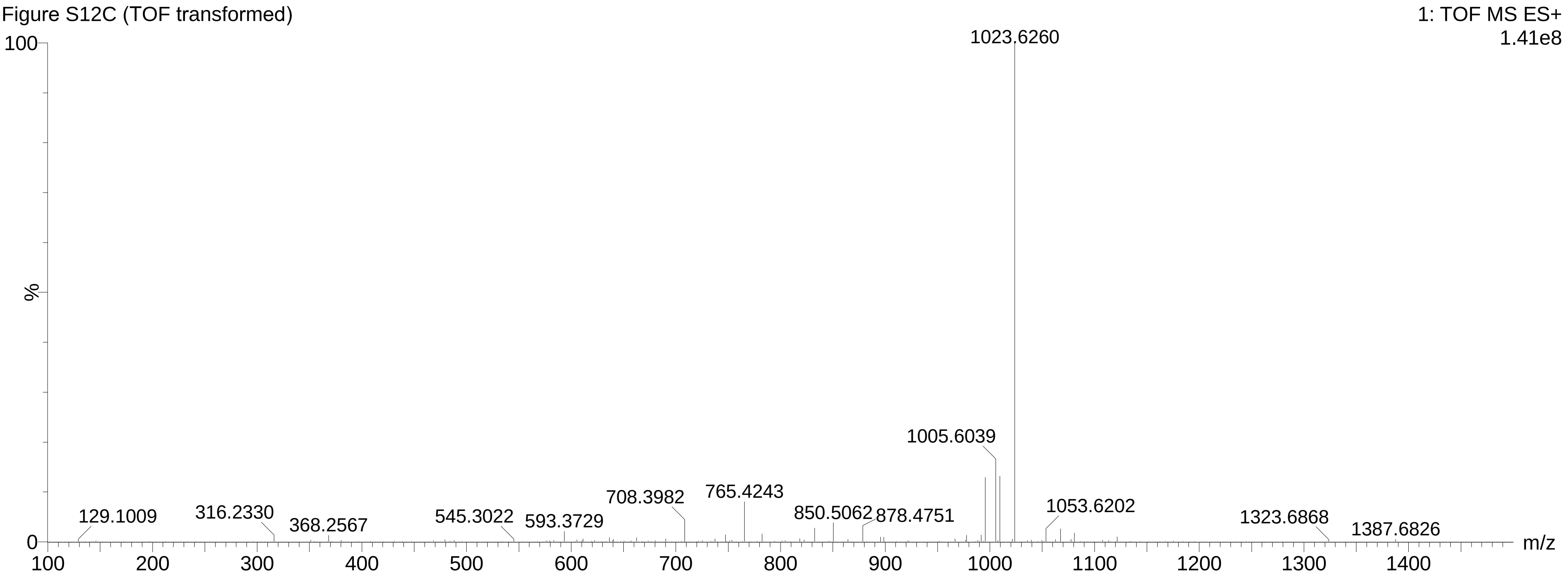
Figure S12C (TOF transformed)
1: TOF MS ES+
1.41e8
100
%
0
1023.6260
1005.6039
129.1009
316.2330
368.2567
545.3022
593.3729
708.3982
765.4243
850.5062
878.4751
1053.6202
1323.6868
1387.6826
m/z
100
200
300
400
500
600
700
800
900
1000
1100
1200
1300
1400

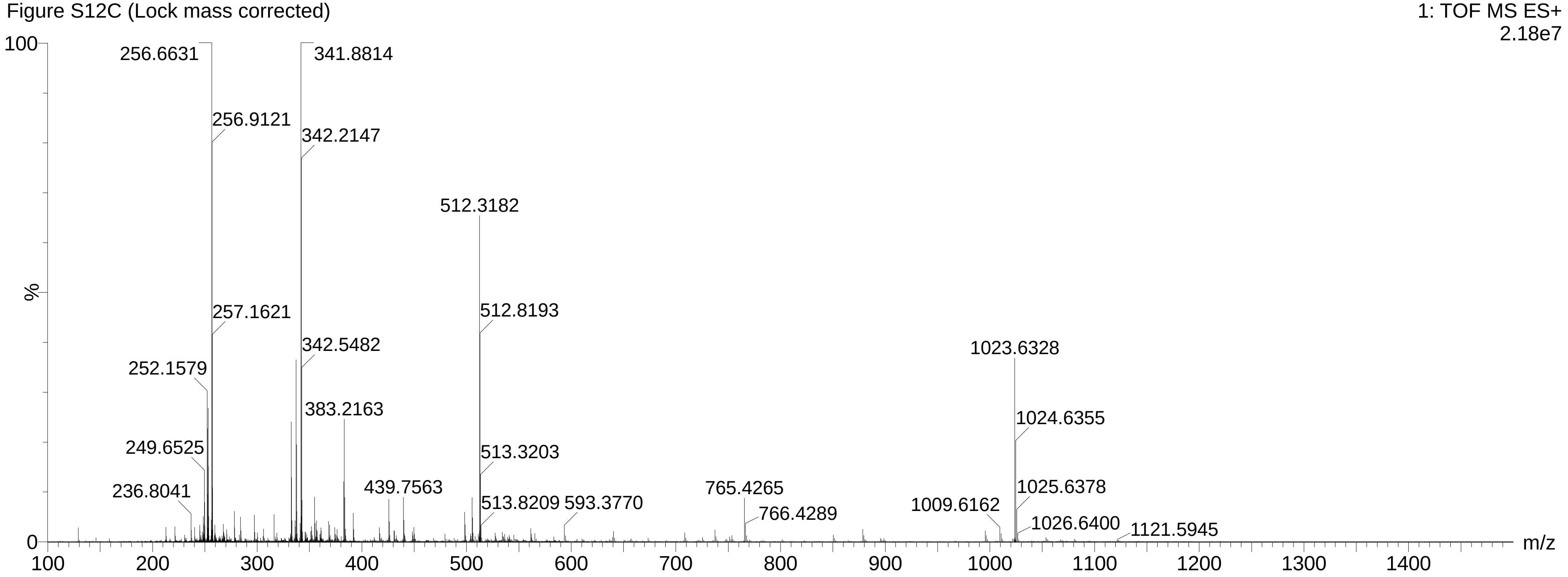
Figure S12C (Lock mass corrected)
1: TOF MS ES+
2.18e7
100
%
0
256.6631
341.8814
256.9121
342.2147
512.3182
257.1621
512.8193
342.5482
252.1579
1023.6328
383.2163
1024.6355
249.6525
513.3203
236.8041
439.7563
765.4265
1025.6378
513.8209
593.3770
1009.6162
766.4289
1026.6400
1121.5945
m/z
100
200
300
400
500
600
700
800
900
1000
1100
1200
1300
1400

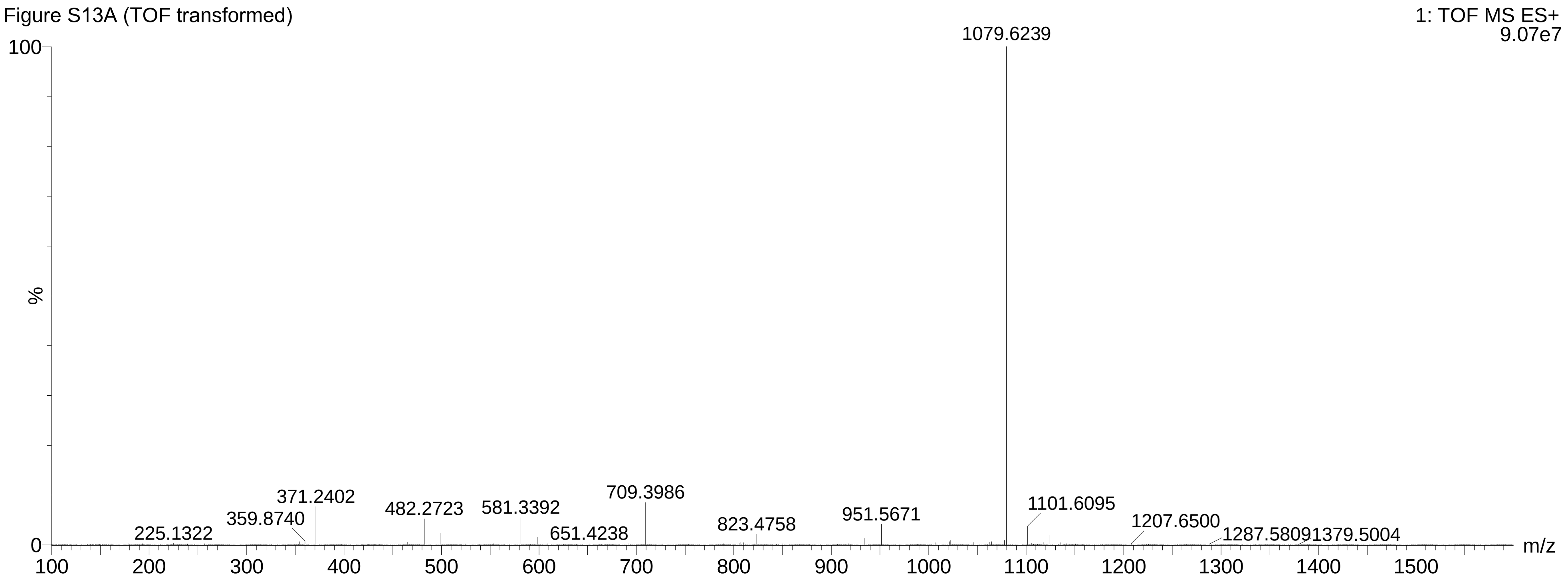

Figure S13A (TOF transformed)
1: TOF MS ES+
9.07e7
1079.6239
371.2402
359.8740
225.1322
482.2723
581.3392
651.4238
709.3986
823.4758
951.5671
1101.6095
1207.6500
1287.5809
1379.5004
100
%
0
m/z
100
200
300
400
500
600
700
800
900
1000
1100
1200
1300
1400
1500

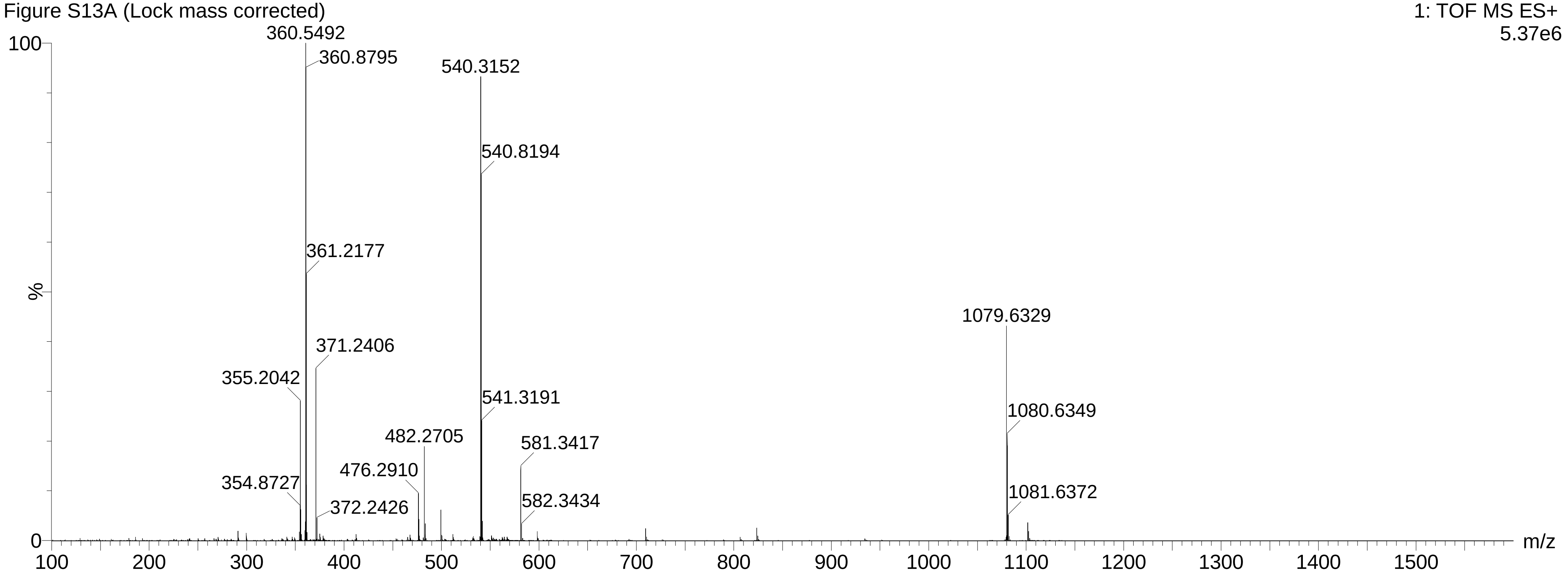

Figure S13A (Lock mass corrected)
1: TOF MS ES+
5.37e6
360.5492
360.8795
540.3152
540.8194
361.2177
1079.6329
371.2406
355.2042
541.3191
1080.6349
482.2705
581.3417
476.2910
354.8727
372.2426
582.3434
1081.6372
100
%
0
m/z
100
200
300
400
500
600
700
800
900
1000
1100
1200
1300
1400
1500

Figure S13B (TOF transformed)

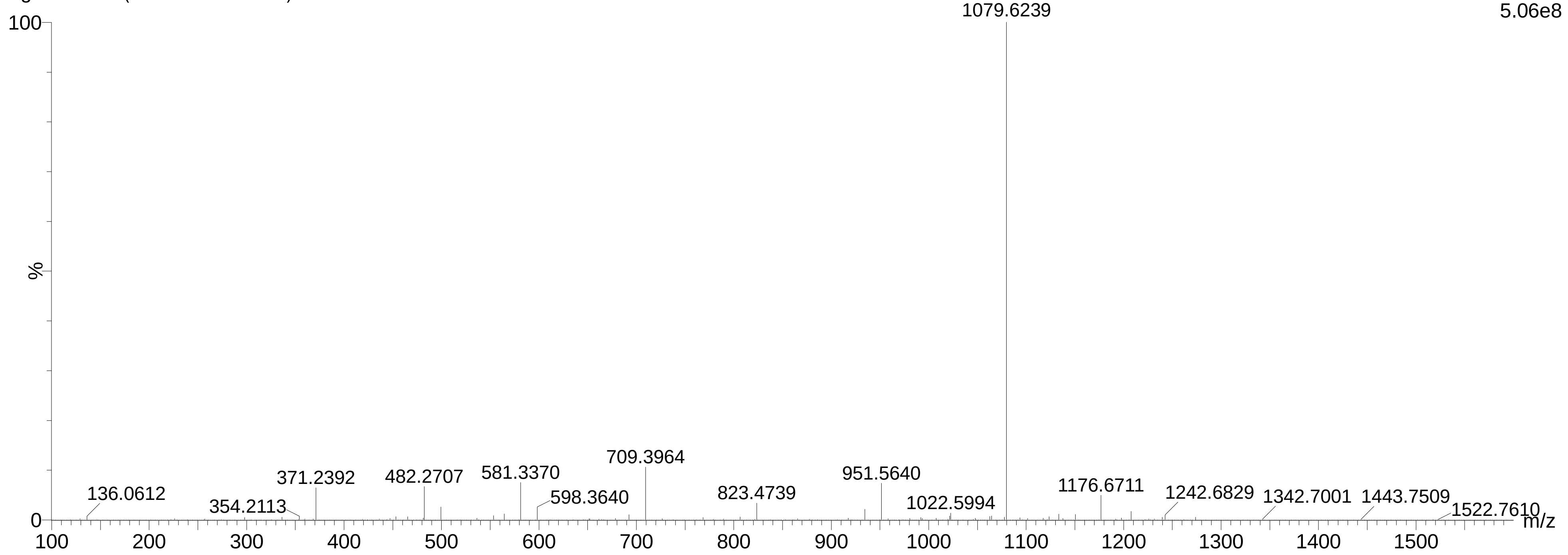

Figure S13B (Lock mass corrected)

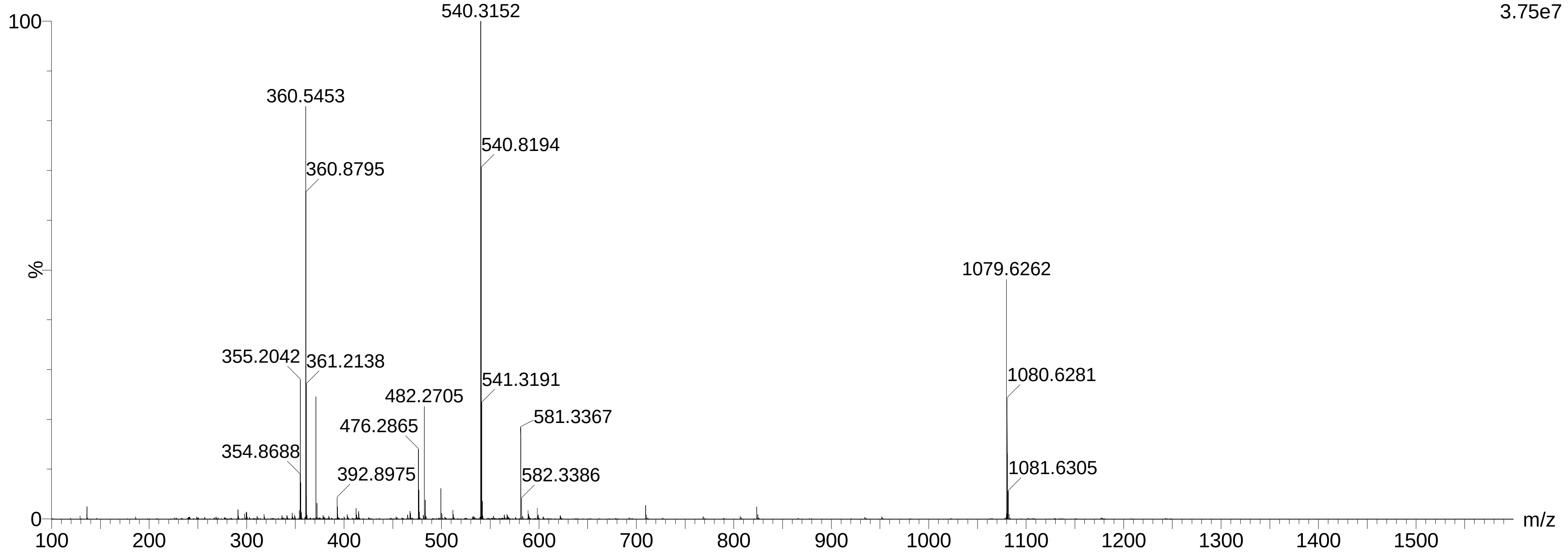

Figure S13C (TOF transformed)

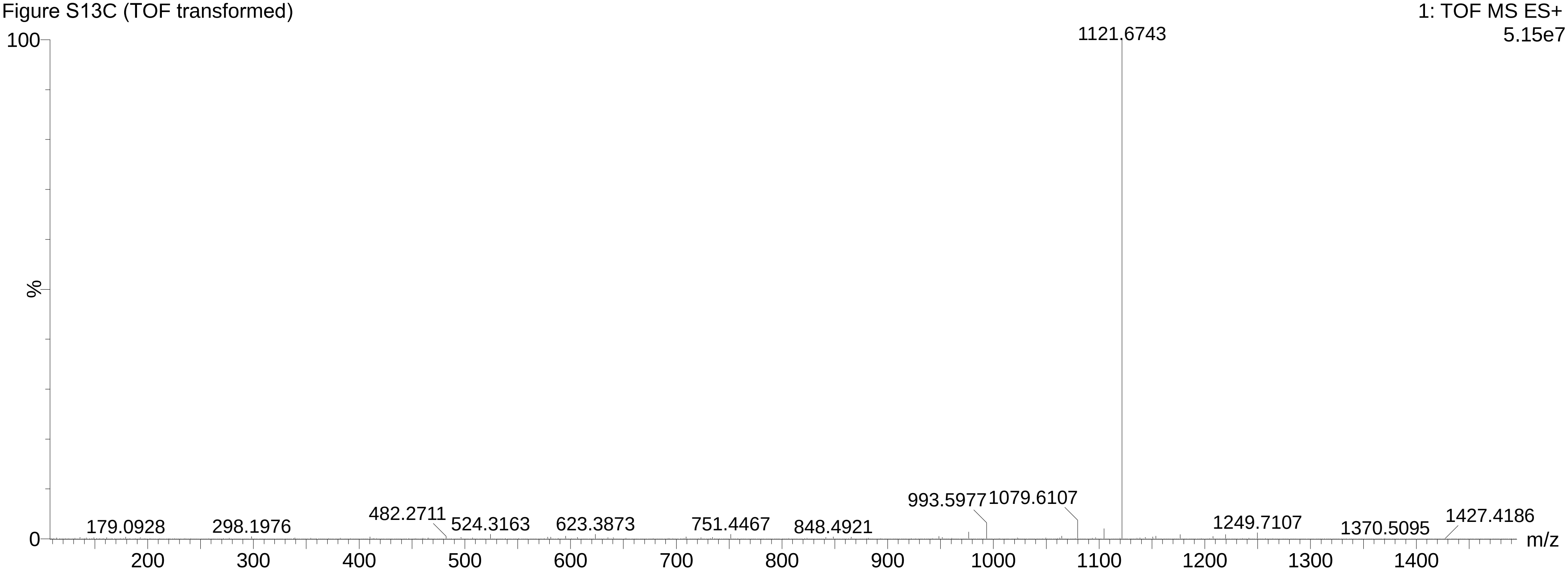

Figure S13C (Lock mass corrected)

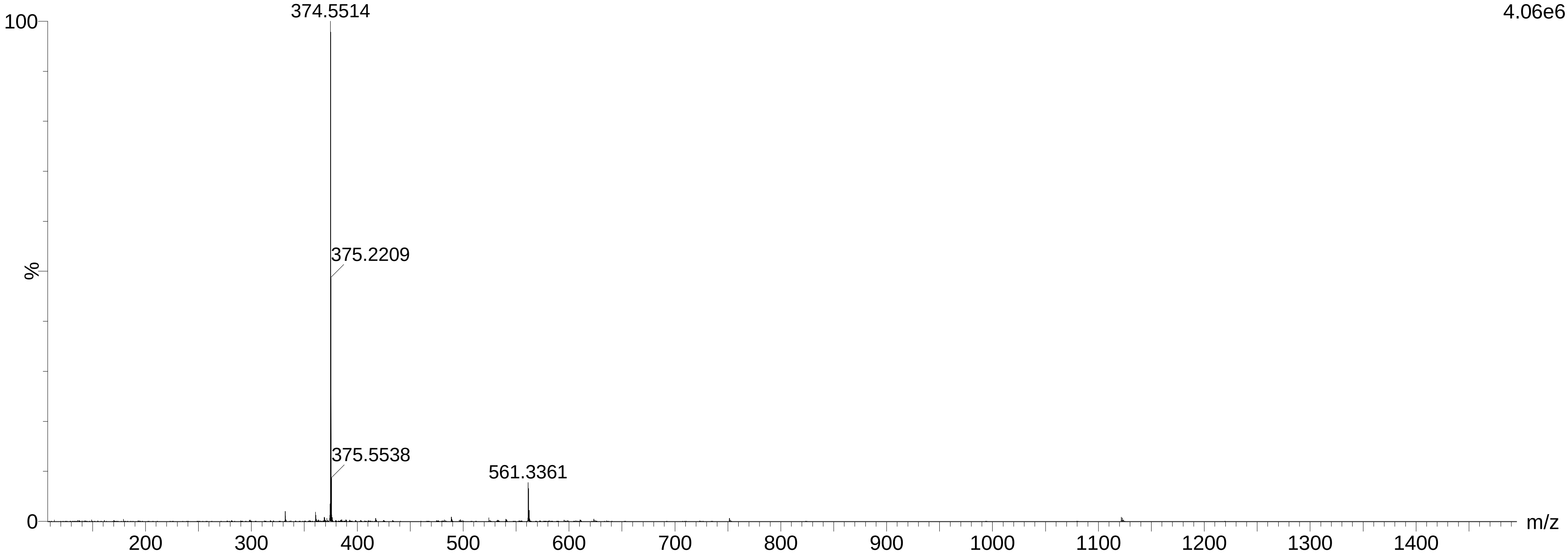

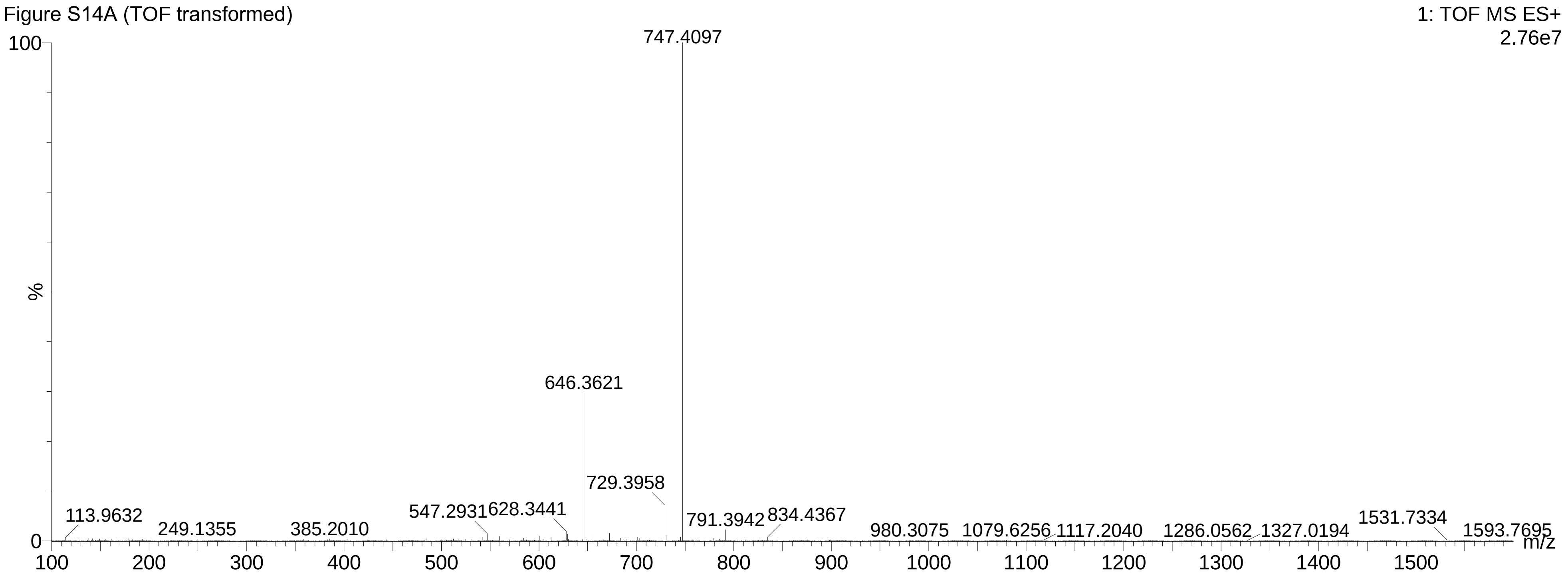

Figure S14A (TOF transformed)
1: TOF MS ES+
2.76e7
747.4097
646.3621
729.3958
113.9632
249.1355
385.2010
547.2931
628.3441
791.3942
834.4367
980.3075
1079.6256
1117.2040
1286.0562
1327.0194
1531.7334
1593.7695
%
m/z

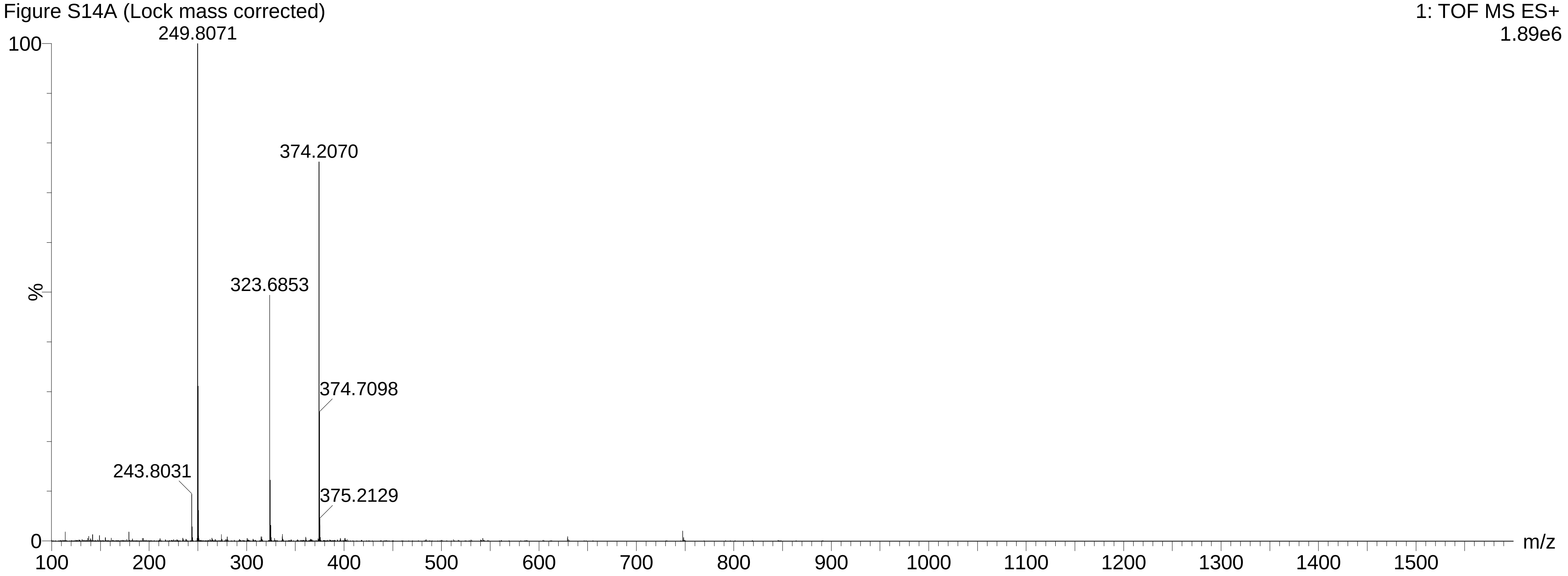

Figure S14A (Lock mass corrected)
1: TOF MS ES+
1.89e6
249.8071
374.2070
323.6853
374.7098
243.8031
375.2129
%
m/z

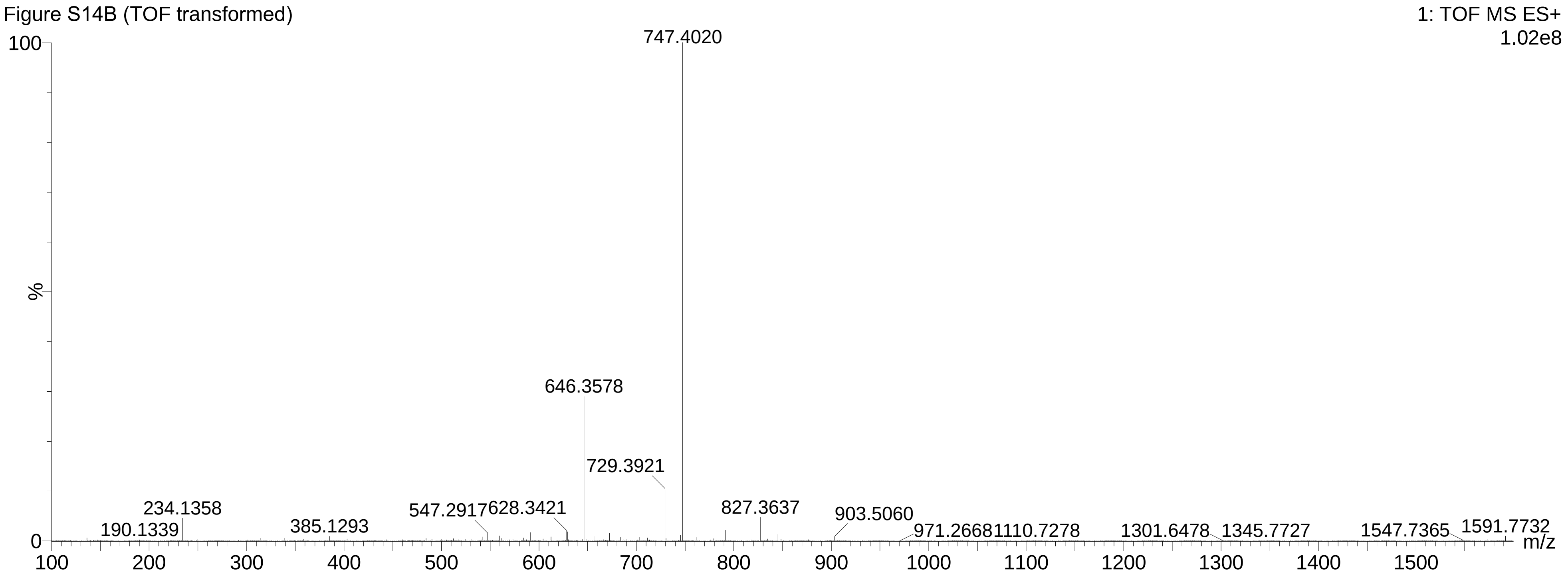
Figure S14B (TOF transformed)
1: TOF MS ES+
1.02e8
747.4020
646.3578
729.3921
234.1358
190.1339
385.1293
547.2917
628.3421
827.3637
903.5060
971.2668
1110.7278
1301.6478
1345.7727
1547.7365
1591.7732
%
m/z

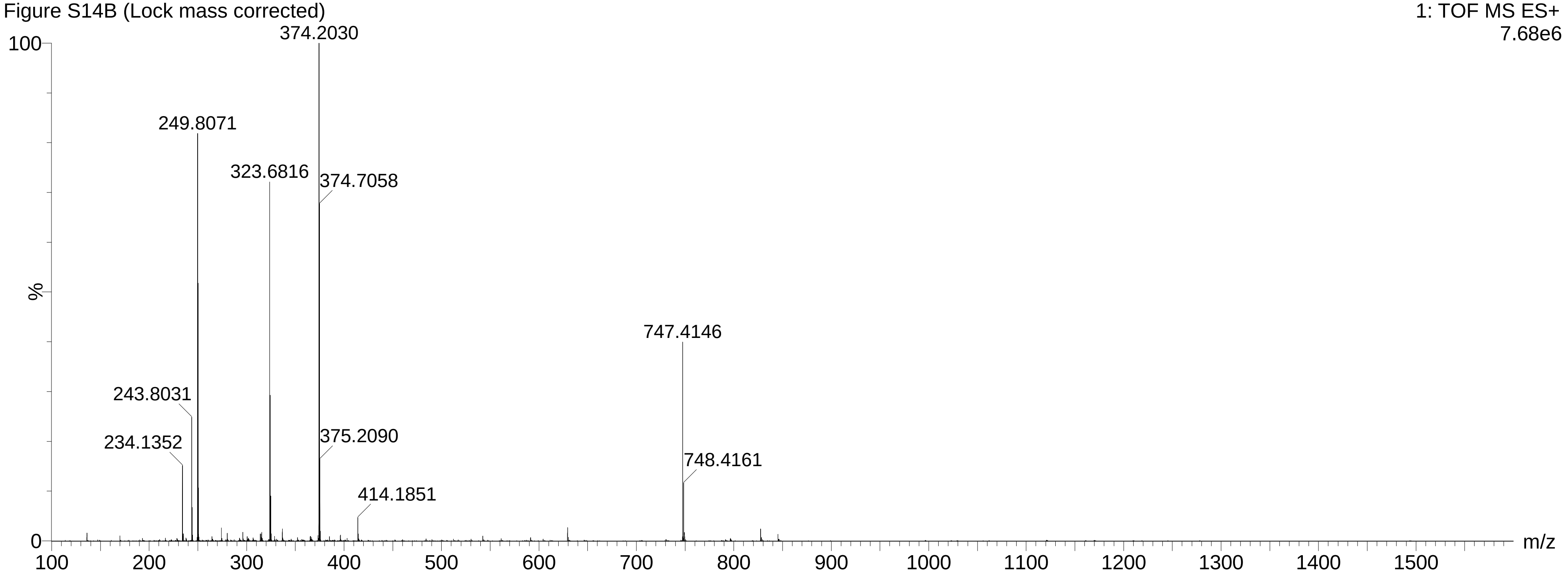
Figure S14B (Lock mass corrected)
1: TOF MS ES+
7.68e6
374.2030
249.8071
323.6816
374.7058
747.4146
243.8031
234.1352
375.2090
748.4161
414.1851
%
m/z

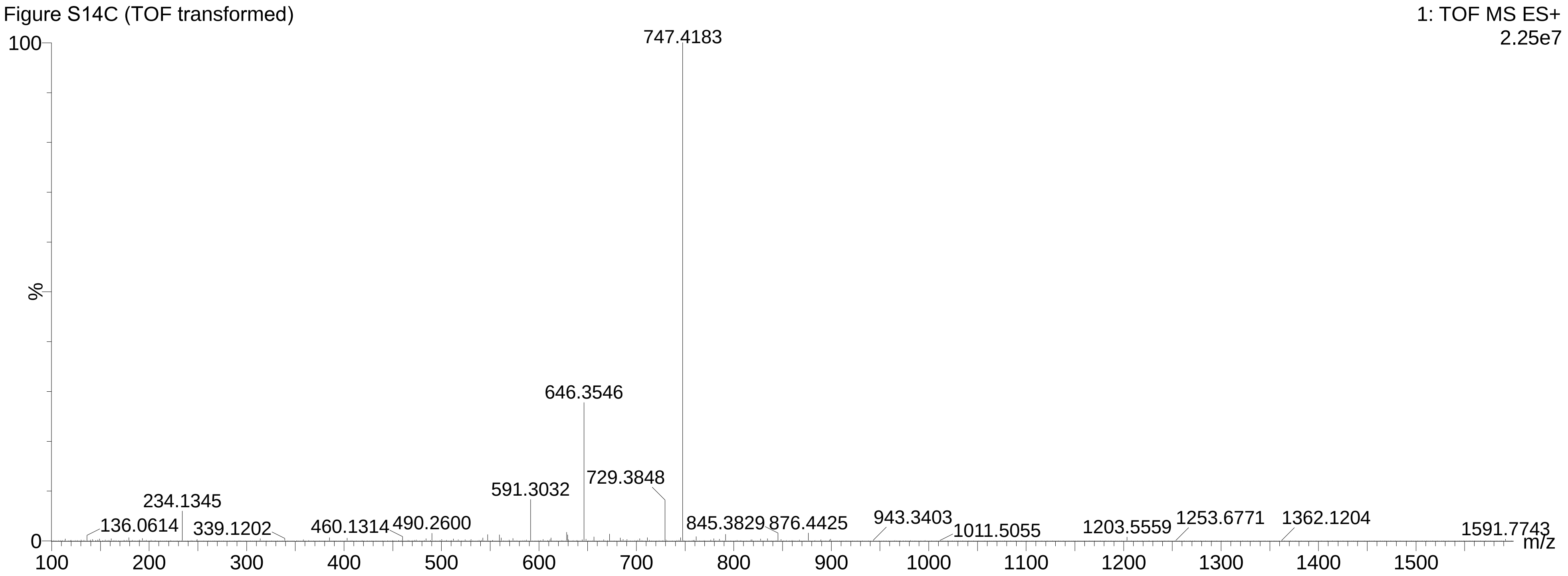
Figure S14C (TOF transformed)
1: TOF MS ES+
2.25e7
747.4183
646.3546
729.3848
591.3032
234.1345
136.0614
339.1202
460.1314
490.2600
845.3829
876.4425
943.3403
1011.5055
1203.5559
1253.6771
1362.1204
1591.7743
%
m/z

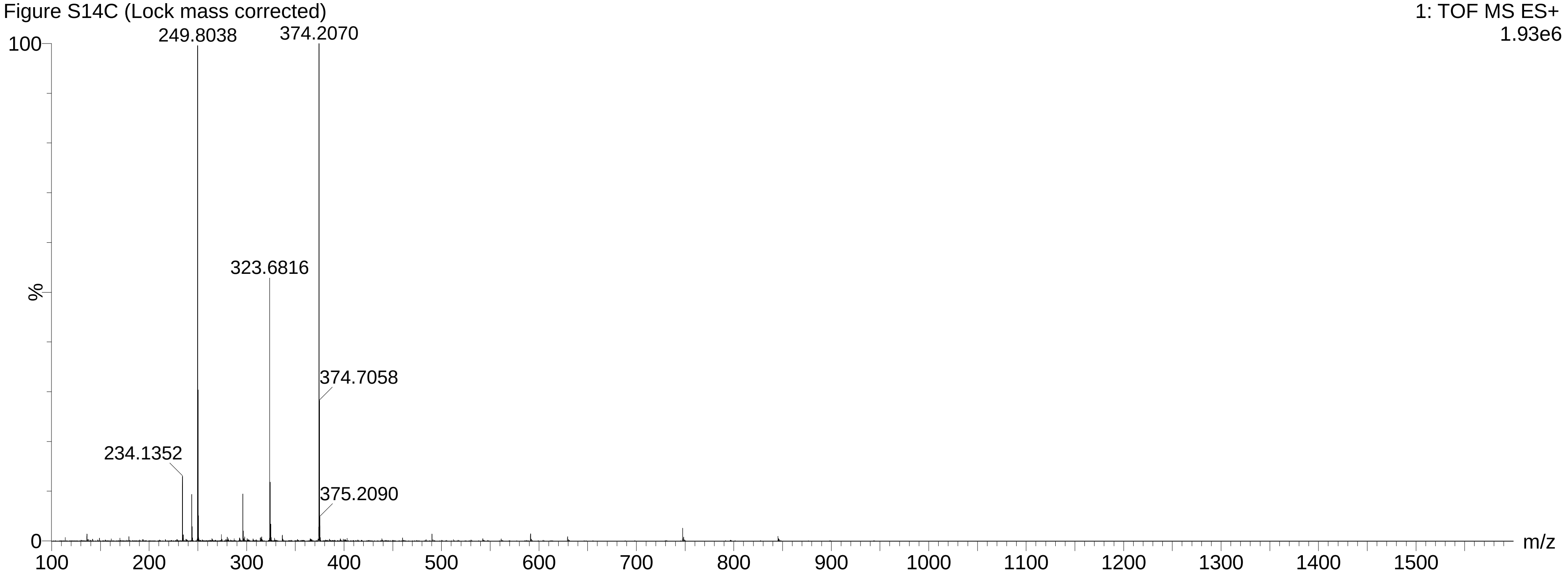
Figure S14C (Lock mass corrected)
1: TOF MS ES+
1.93e6
249.8038
374.2070
323.6816
374.7058
234.1352
375.2090
%
m/z

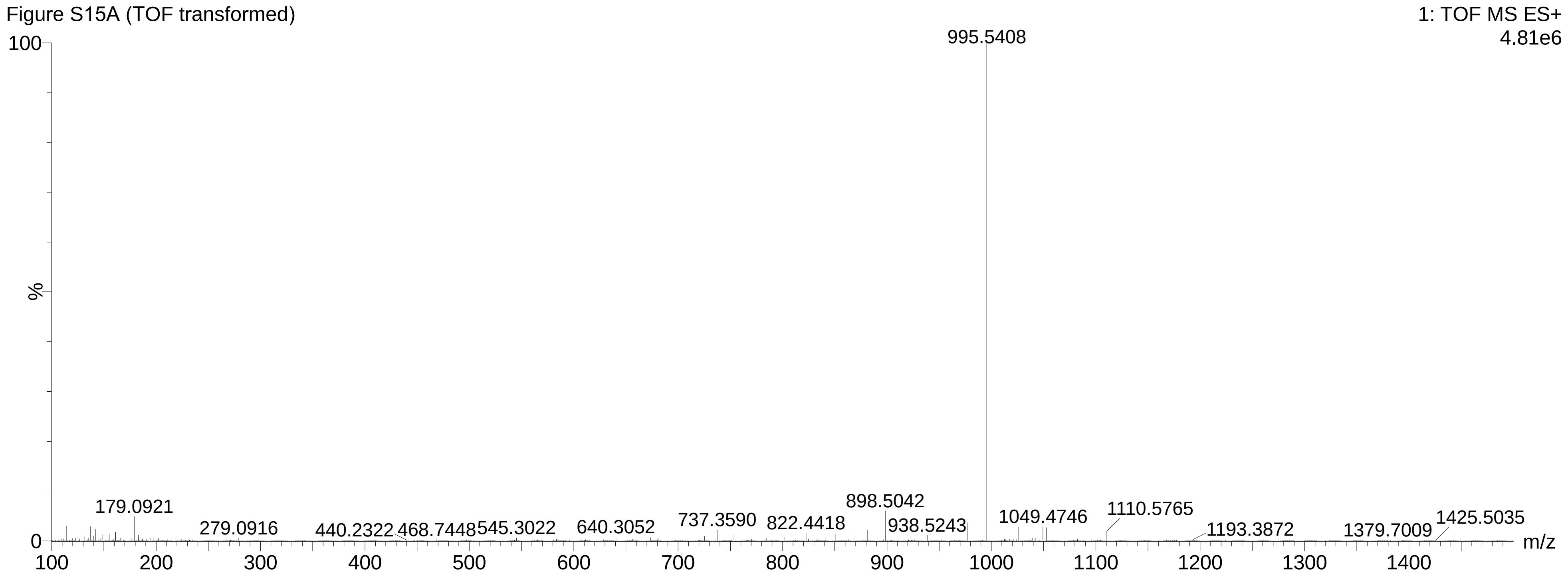
Figure S15A (TOF transformed)
1: TOF MS ES+
4.81e6
995.5408
179.0921
279.0916
440.2322
468.7448
545.3022
640.3052
737.3590
822.4418
898.5042
938.5243
1049.4746
1110.5765
1193.3872
1379.7009
1425.5035
%
m/z

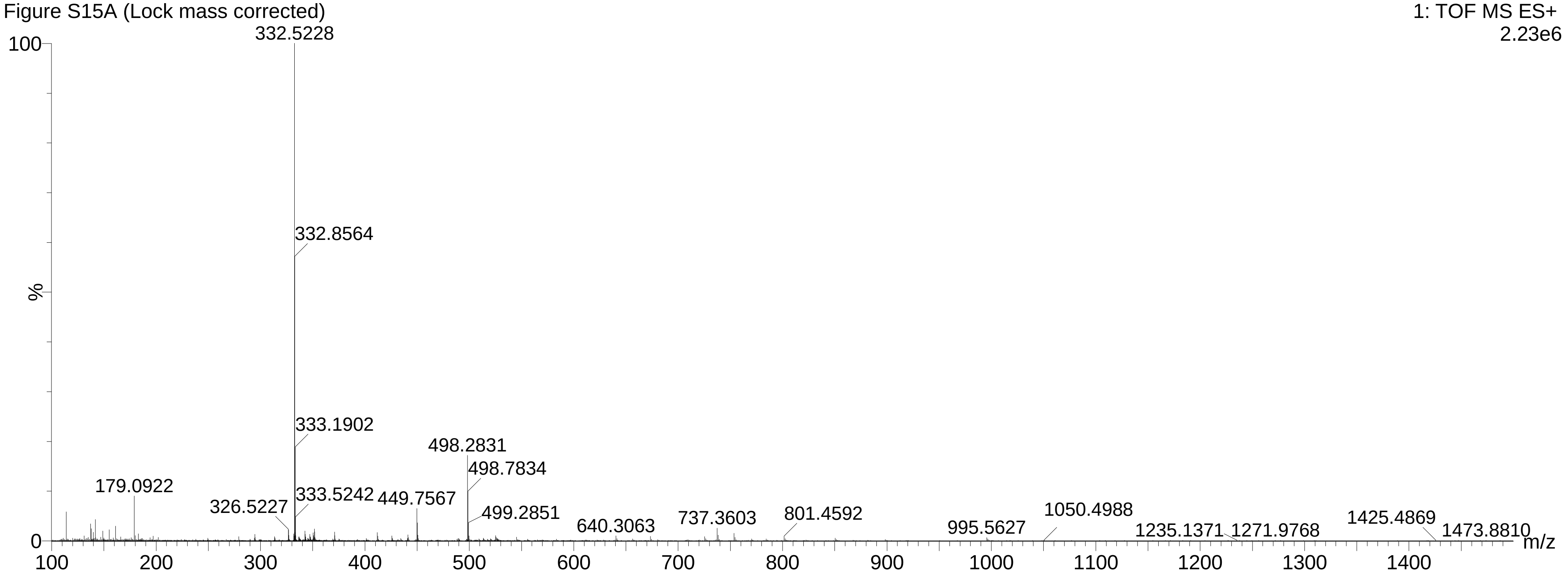
Figure S15A (Lock mass corrected)
1: TOF MS ES+
2.23e6
332.5228
332.8564
333.1902
498.2831
498.7834
179.0922
326.5227
333.5242
449.7567
499.2851
640.3063
737.3603
801.4592
995.5627
1050.4988
1235.1371
1271.9768
1425.4869
1473.8810
%
m/z

Figure S15B (TOF transformed)

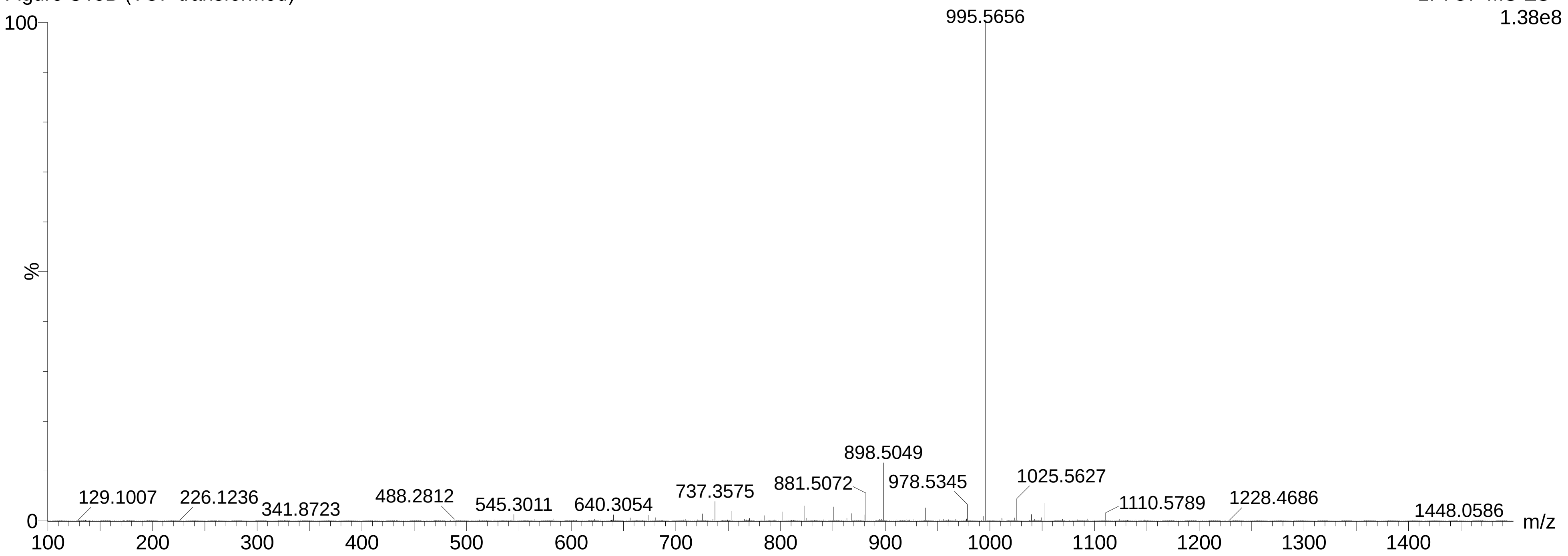

Figure S15B (Lock mass corrected)

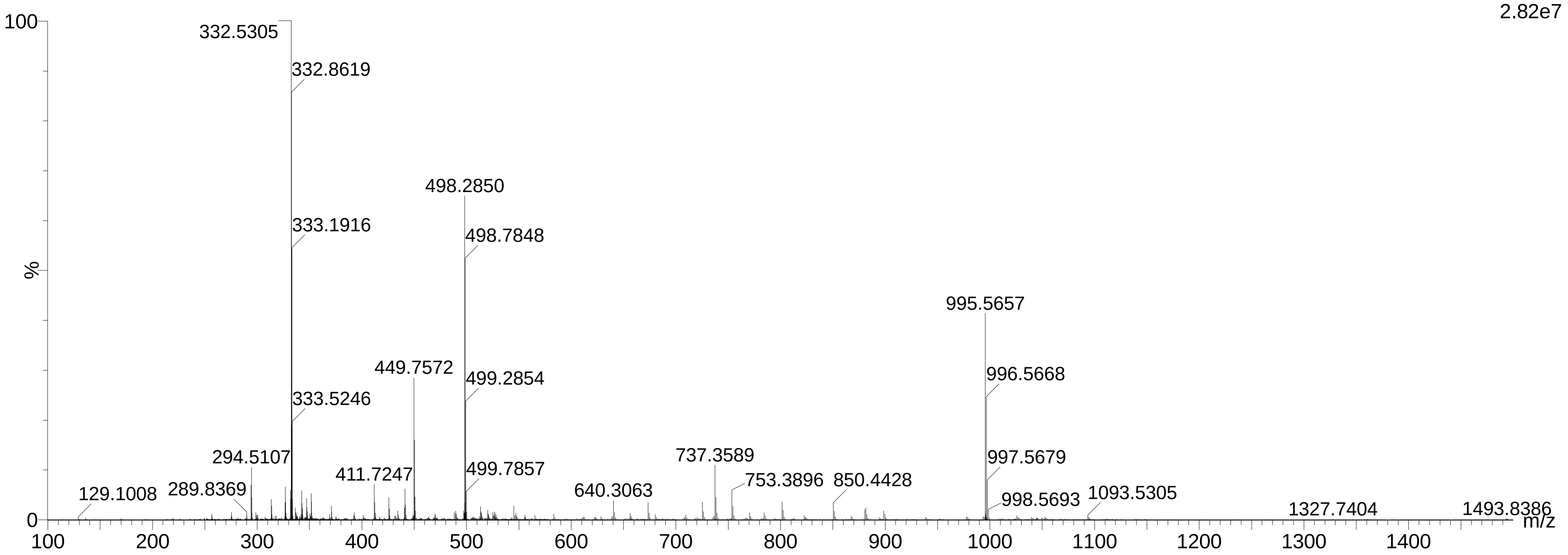

Figure S15C (TOF transformed)

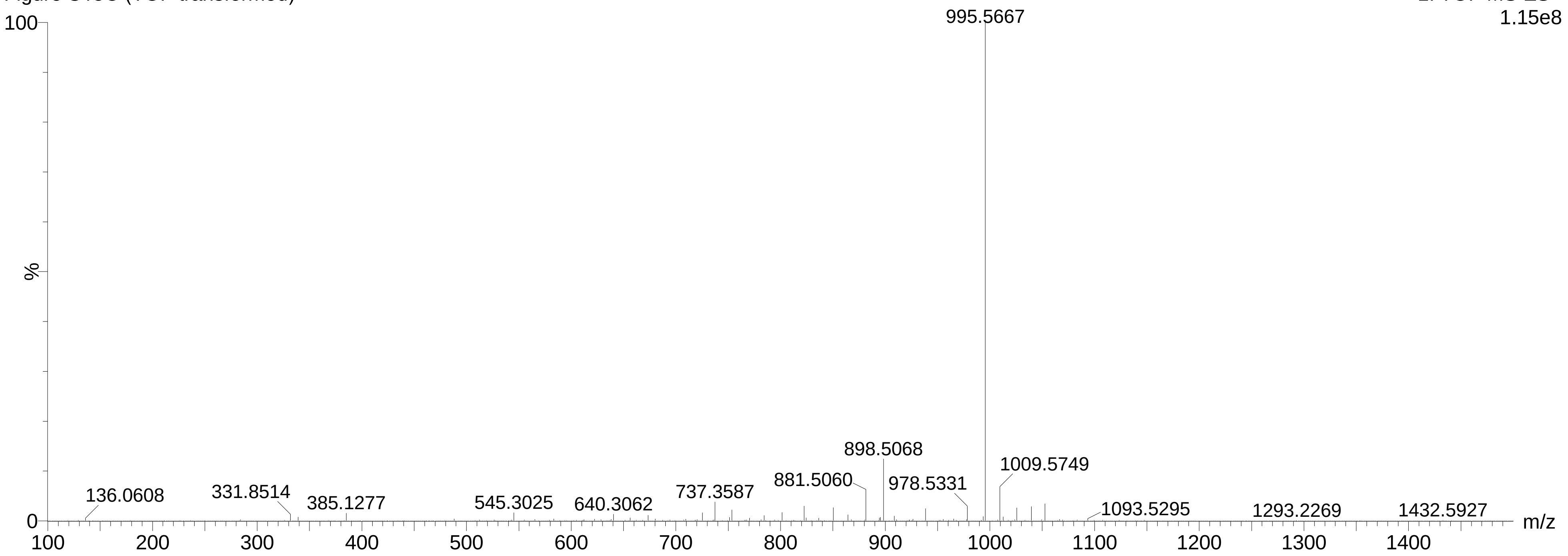

Figure S15C (Lock mass corrected)

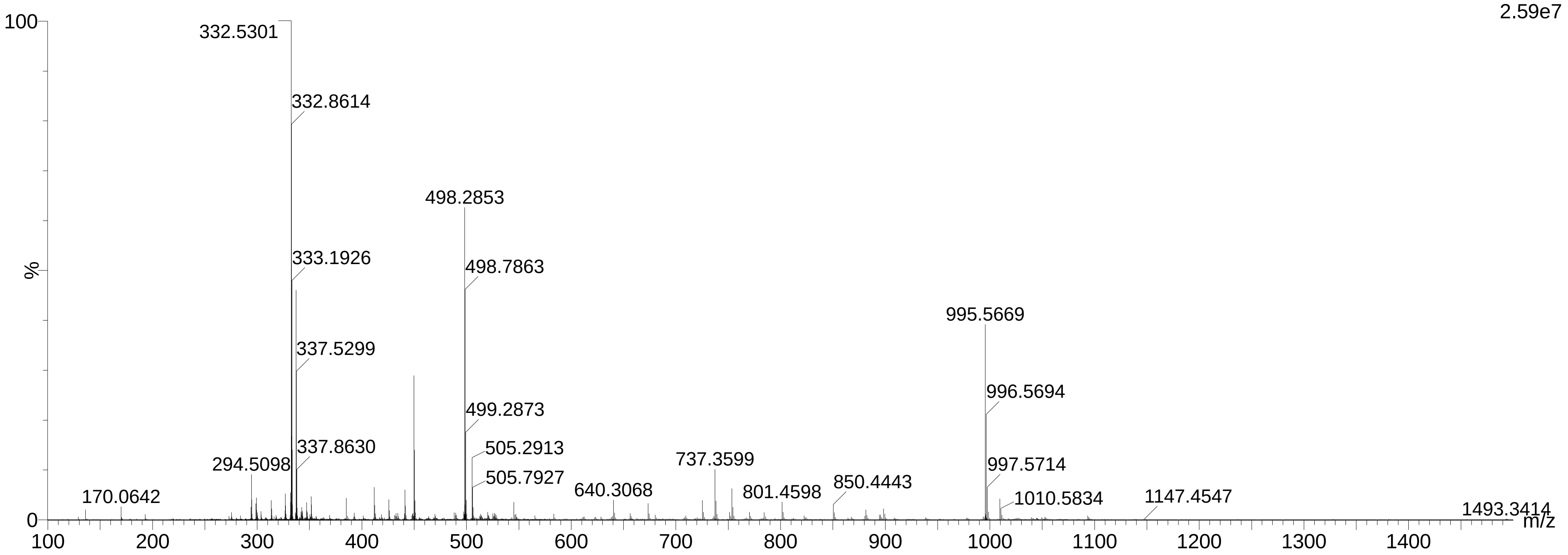

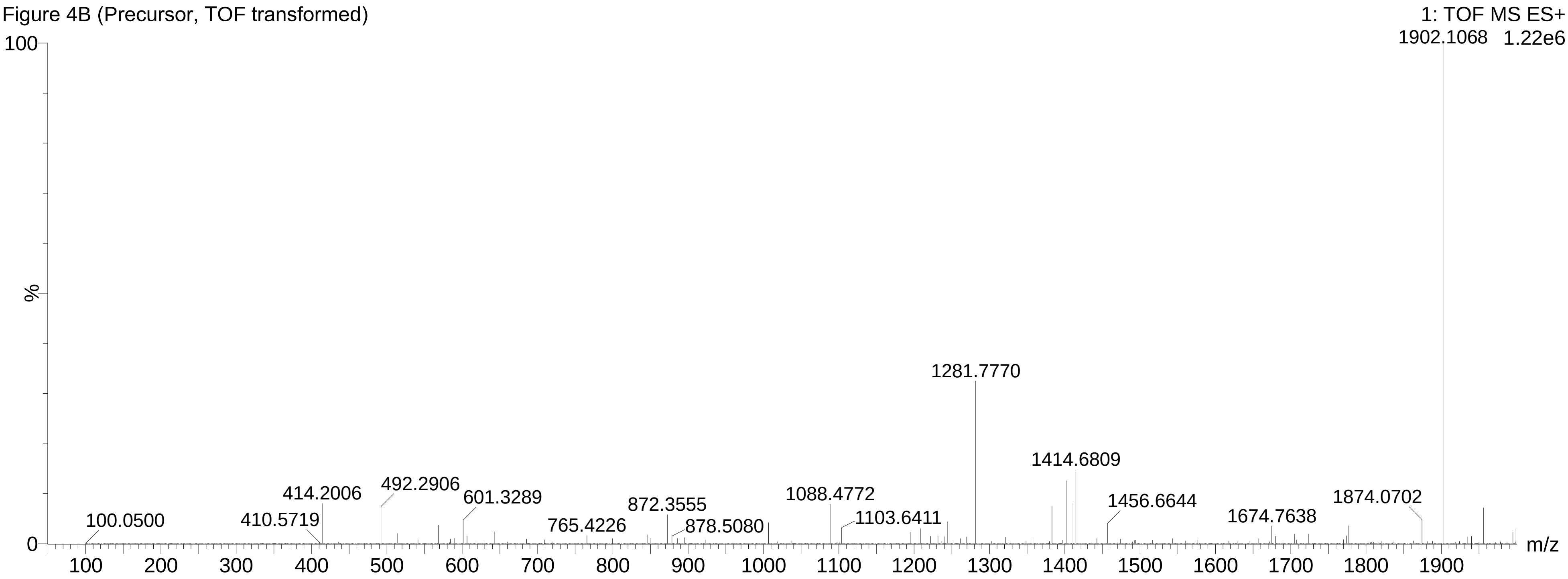
Figure 4B (Precursor, TOF transformed)
1: TOF MS ES+
1902.1068
1.22e6
100
%
0
1281.7770
1414.6809
1456.6644
1674.7638
1874.0702
1088.4772
1103.6411
872.3555
878.5080
765.4226
601.3289
492.2906
414.2006
410.5719
100.0500
m/z
100
200
300
400
500
600
700
800
900
1000
1100
1200
1300
1400
1500
1600
1700
1800
1900

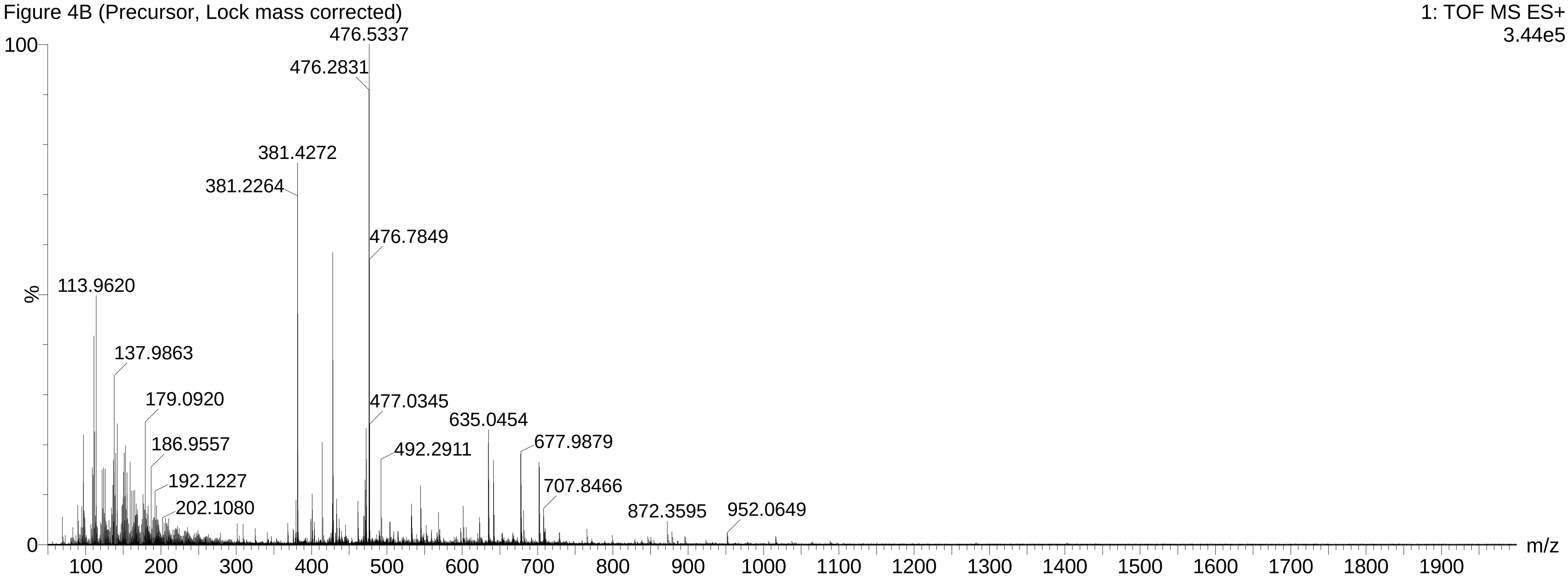
Figure 4B (Precursor, Lock mass corrected)
1: TOF MS ES+
3.44e5
100
%
0
476.5337
476.2831
381.4272
381.2264
476.7849
113.9620
137.9863
179.0920
477.0345
186.9557
635.0454
677.9879
492.2911
192.1227
707.8466
202.1080
872.3595
952.0649
m/z
100
200
300
400
500
600
700
800
900
1000
1100
1200
1300
1400
1500
1600
1700
1800
1900

Figure 4B (TOF transformed)

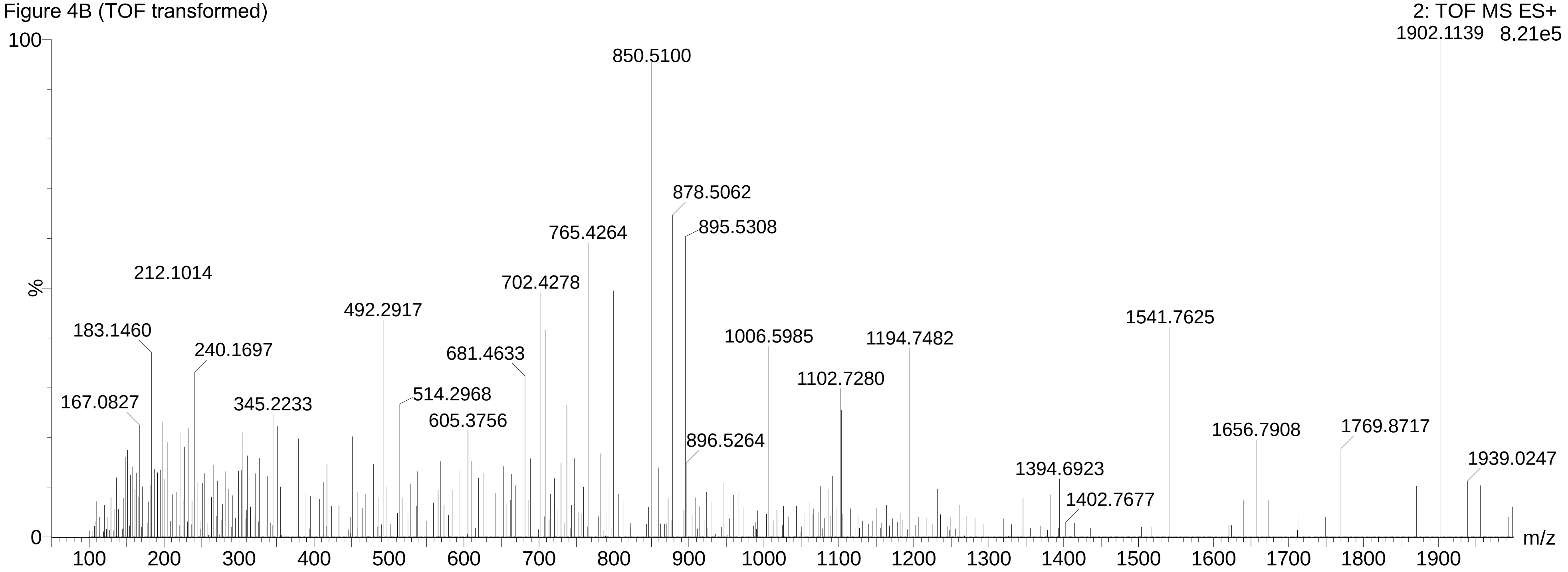

Figure 4B (Lock mass corrected)

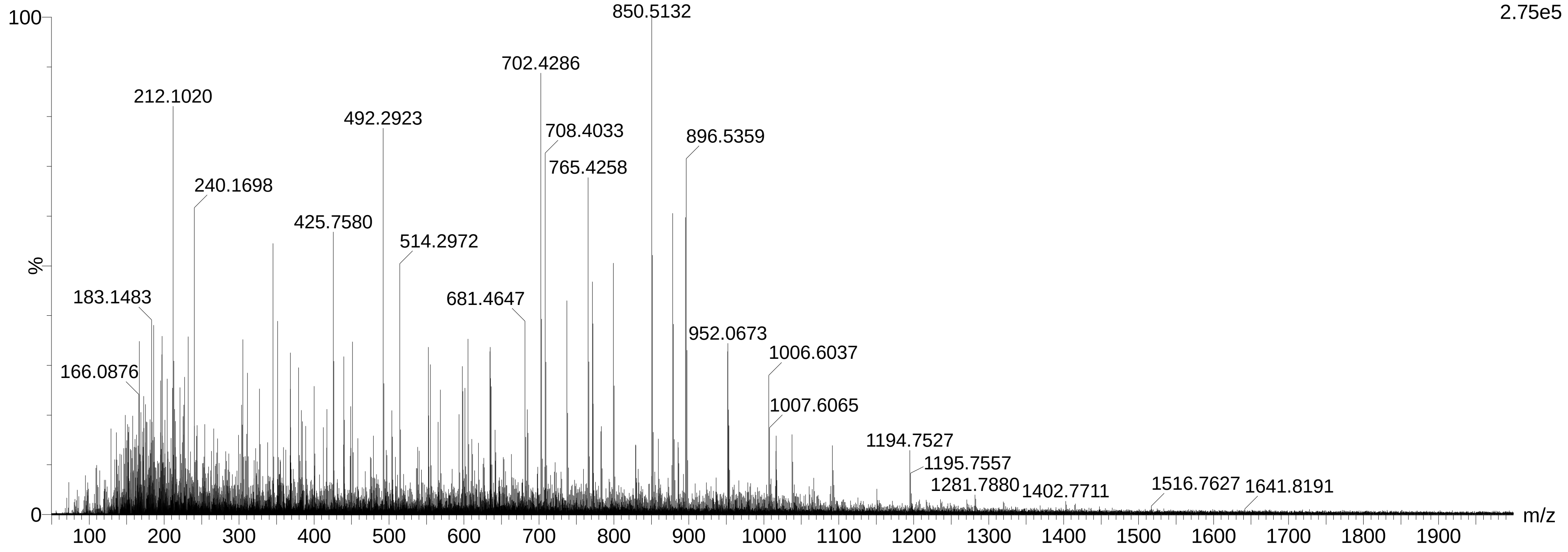

Figure 4C (Precursor, TOF transformed)

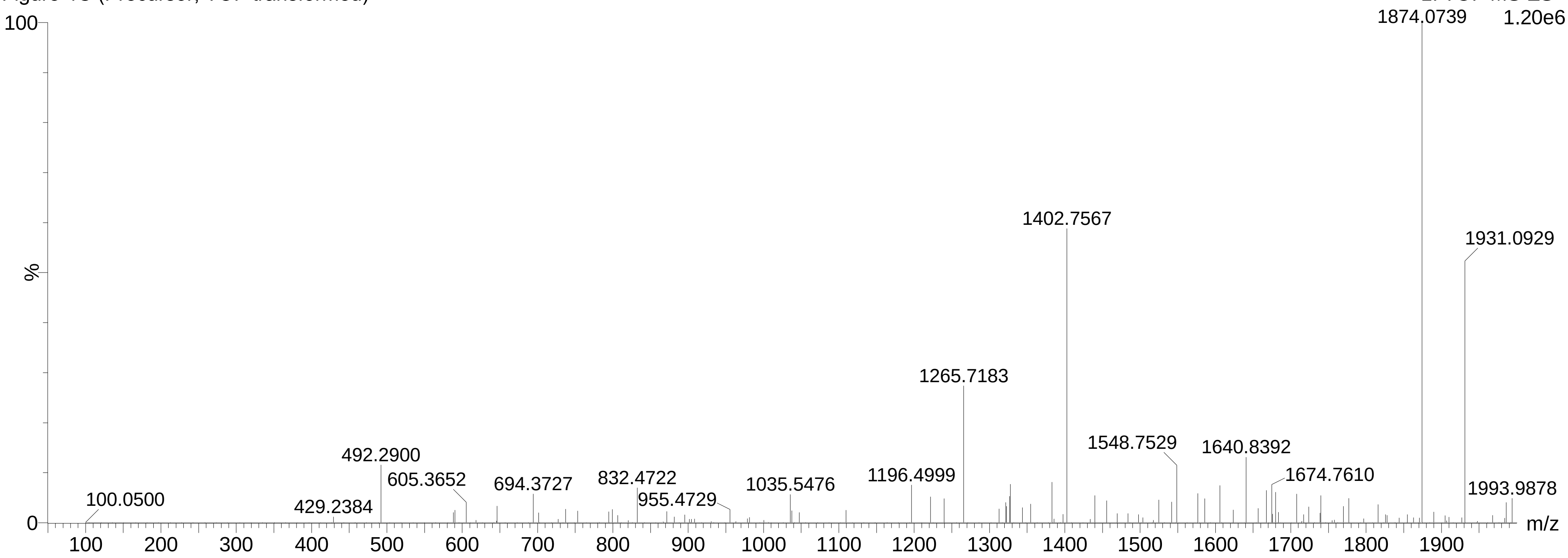

Figure 4C (Precursor, Lock mass corrected)

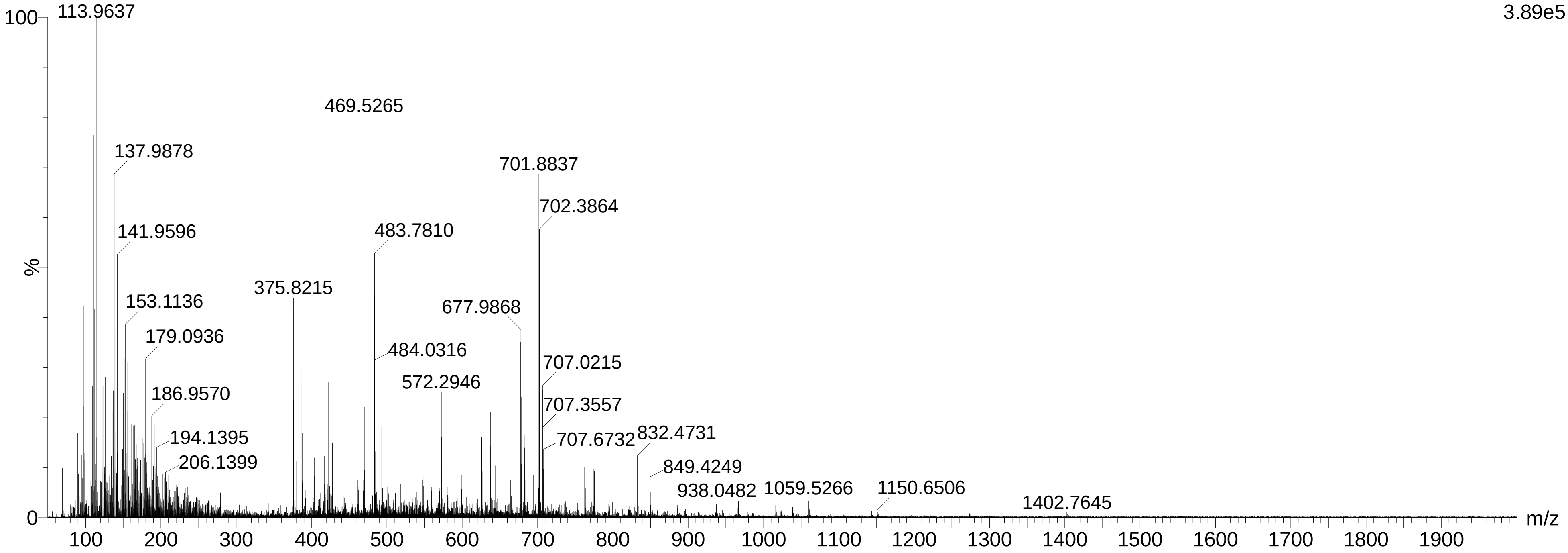

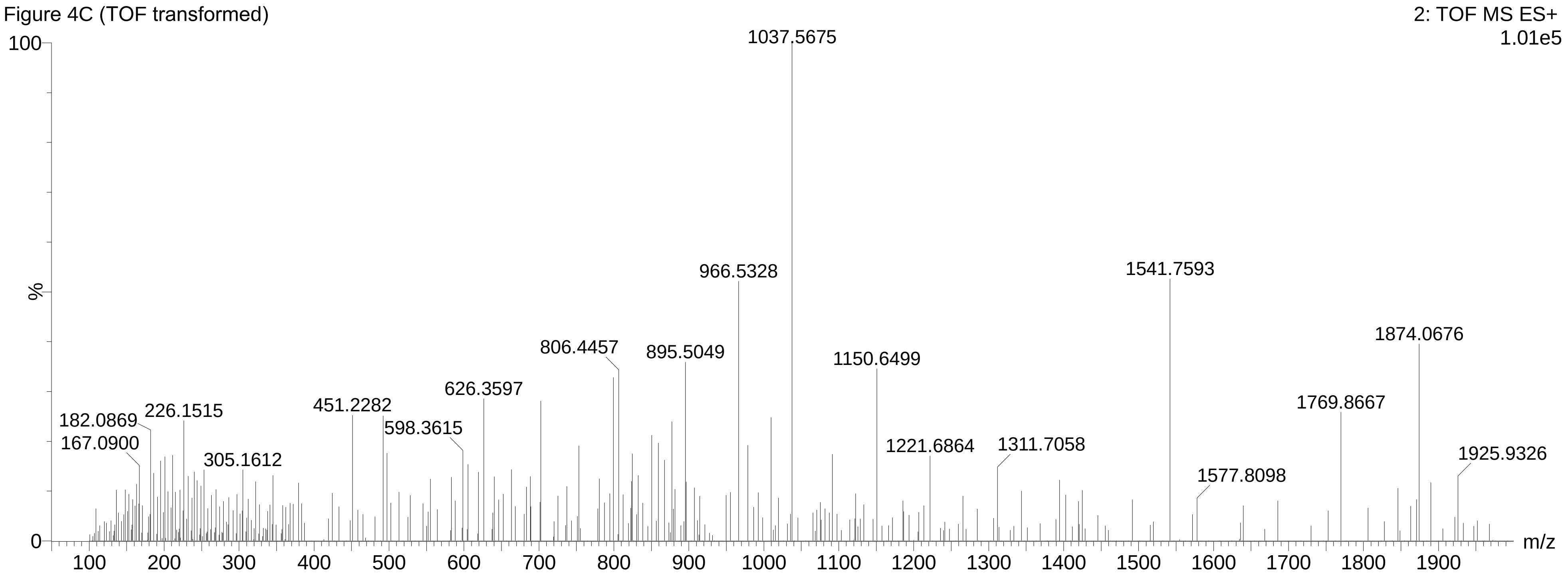

Figure 4C (TOF transformed)
2: TOF MS ES+
1.01e5
1037.5675
966.5328
1541.7593
1874.0676
806.4457
895.5049
1150.6499
626.3597
1769.8667
451.2282
182.0869
226.1515
598.3615
167.0900
1221.6864
1311.7058
1925.9326
305.1612
1577.8098
100
%
0
m/z

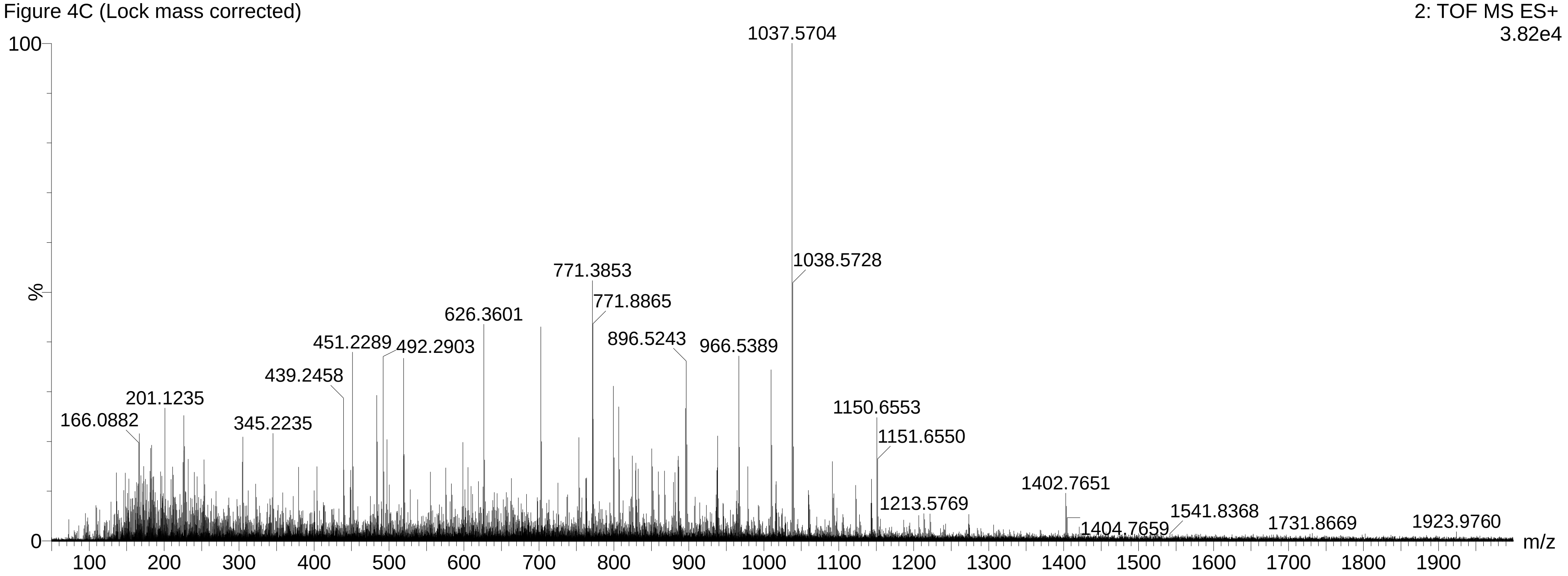

Figure 4C (Lock mass corrected)
2: TOF MS ES+
3.82e4
1037.5704
1038.5728
771.3853
771.8865
626.3601
896.5243
451.2289
492.2903
966.5389
439.2458
201.1235
166.0882
345.2235
1150.6553
1151.6550
1402.7651
1213.5769
1541.8368
1404.7659
1731.8669
1923.9760
100
%
0
m/z

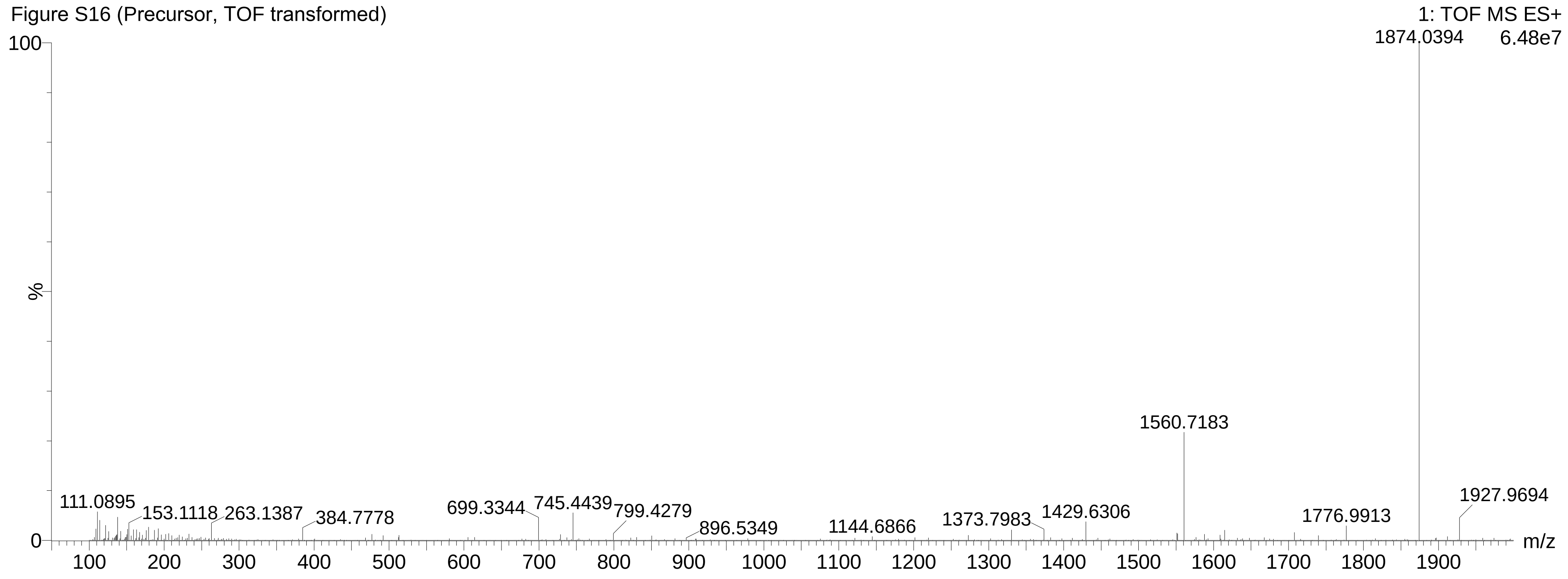
Figure S16 (Precursor, TOF transformed)
1: TOF MS ES+
6.48e7
1874.0394
1560.7183
111.0895
153.1118
263.1387
384.7778
699.3344
745.4439
799.4279
896.5349
1144.6866
1373.7983
1429.6306
1776.9913
1927.9694
%
m/z

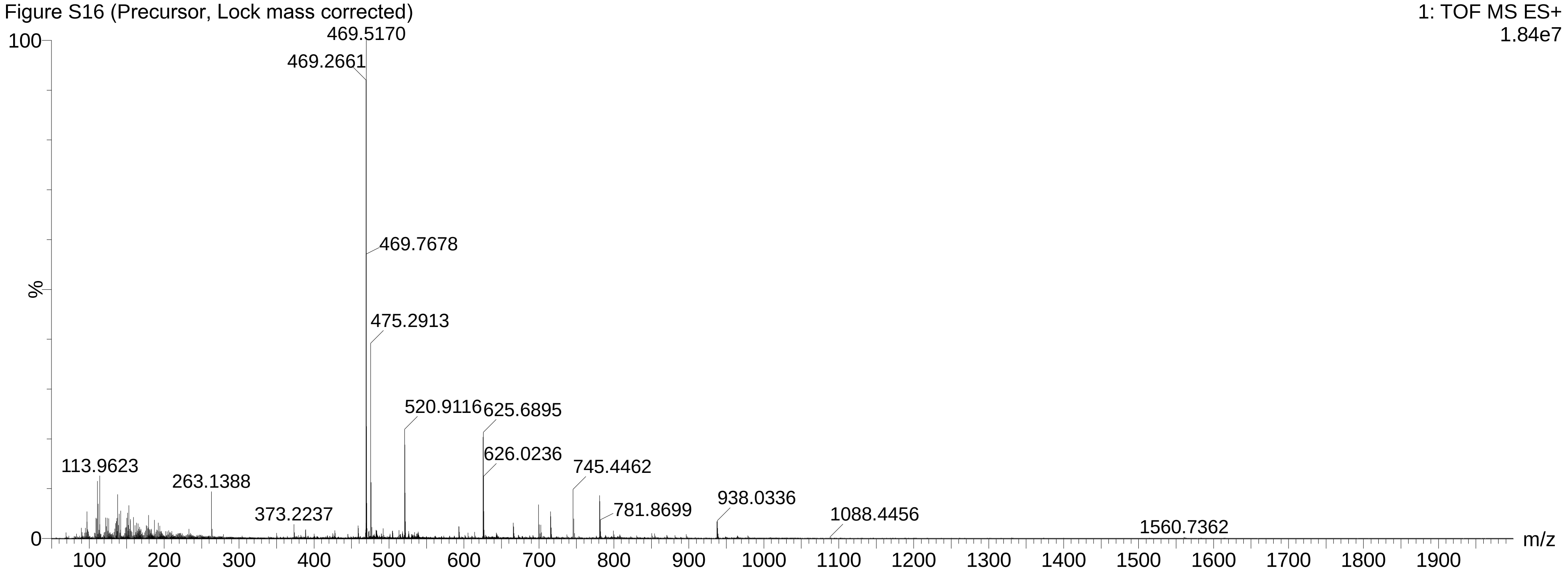
Figure S16 (Precursor, Lock mass corrected)
1: TOF MS ES+
1.84e7
469.5170
469.2661
469.7678
475.2913
520.9116
625.6895
626.0236
745.4462
781.8699
938.0336
1088.4456
1560.7362
113.9623
263.1388
373.2237
%
m/z

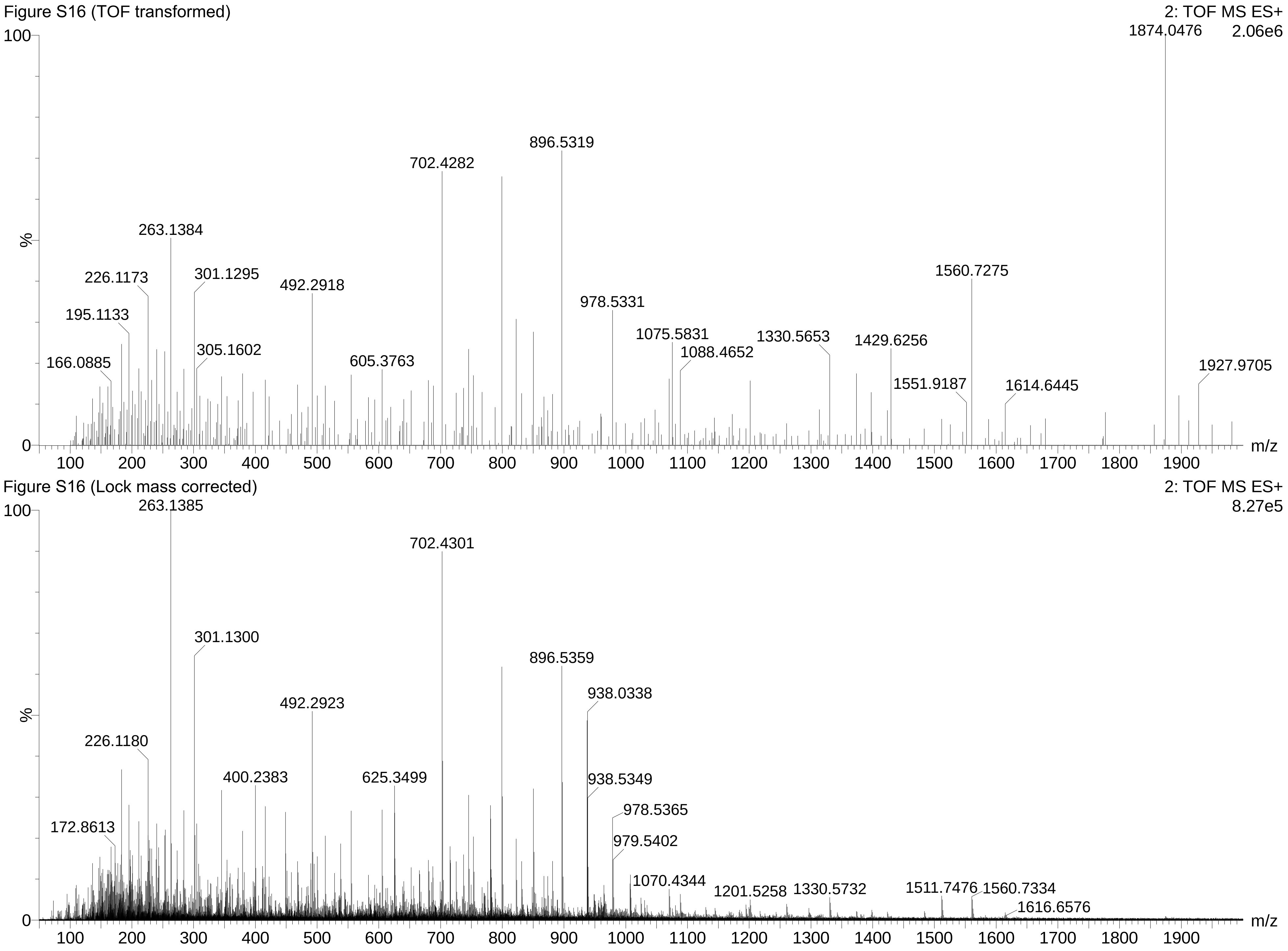
Figure S16 (TOF transformed)
2: TOF MS ES+
2.06e6
1874.0476
896.5319
702.4282
263.1384
226.1173
301.1295
492.2918
195.1133
978.5331
1560.7275
1075.5831
1330.5653
1429.6256
1088.4652
305.1602
166.0885
605.3763
1927.9705
1551.9187
1614.6445
%
m/z
Figure S16 (Lock mass corrected)
2: TOF MS ES+
8.27e5
263.1385
702.4301
301.1300
896.5359
938.0338
492.2923
226.1180
400.2383
625.3499
938.5349
978.5365
172.8613
979.5402
1070.4344
1201.5258
1330.5732
1511.7476
1560.7334
1616.6576
%
m/z
